# Jumping plant lice (Hemiptera, Psylloidea) of Bulgaria – an annotated checklist

**DOI:** 10.3897/BDJ.13.e147277

**Published:** 2025-03-24

**Authors:** Monika Pramatarova, Igor Malenovský, Ilia Gjonov

**Affiliations:** 1 Sofia University, Faculty of Biology, Sofia, Bulgaria Sofia University, Faculty of Biology Sofia Bulgaria; 2 National Museum of Natural History - Bulgarian Academy of Sciences, Sofia, Bulgaria National Museum of Natural History - Bulgarian Academy of Sciences Sofia Bulgaria; 3 Department of Botany and Zoology, Faculty of Science, Masaryk University, Brno, Czech Republic Department of Botany and Zoology, Faculty of Science, Masaryk University Brno Czech Republic; 4 Department of Entomology, Moravian Museum, Brno, Czech Republic Department of Entomology, Moravian Museum Brno Czech Republic

**Keywords:** Balkan Peninsula, biodiversity, Europe, faunistics, new records, new synonymy, new host plant records, phytophagous insects, psyllids, Sternorrhyncha, taxonomy, West Palaearctic

## Abstract

**Background:**

Knowledge of the fauna of jumping plant lice or psyllids in Bulgaria is rather scattered. So far, 113 species of psyllids have been recorded from Bulgaria in 51 publications. The aim of this study is to provide an up-to-date checklist of the Psylloidea species from Bulgaria, based on extensive fieldwork by the authors and examination of specimens mainly from the Zoological Collection of Sofia University (BFUS) and the Moravian Museum in Brno (MMBC). In addition, a thorough review of all relevant literature was undertaken to consolidate the existing records.

**New information:**

Twenty-five species of jumping plant lice are recorded here from Bulgaria for the first time. Of these, two species represent new records for Europe: *Dyspersakantshavelii* (Gegechkori, 1977) and *Heterotriozakochiae* (Gegechkori, 1975); and three additional species represent new records for the Balkan Peninsula: *Arytainillaspartiicola* (Šulc, 1912), *Craspedoleptaaraneosa* Loginova, 1962 and *Eryngiofagababugani* (Loginova, 1964). A new synonymy is proposed: *Colposceniaosmanica* Vondráček, 1953 = *Colposceniakiritshenkoi* Loginova, 1960, syn. nov. A lectotype is designated for *C.osmanica* to stabilise the nomenclature. Original drawings or photographs of diagnostic characters on male and female terminalia are provided for *C.osmanica*, *D.kantshavelii*, *Heterotriozaeurotiae* (Loginova, 1960) and *H.kochiae*. Distributional maps summarising all known records from Bulgaria are provided for each species. Where available, photographs of live or mounted specimens and information on host plants are also provided, including new host plant records for 10 species. The previously published records of *Aphalarasauteri* Burckhardt, 1983, *Bactericeraacutipennis* (Zetterstedt, 1828), *B.reuteri* (Šulc, 1913), *Dyspersaapicalis* (Foerster, 1848), *D.viridula* (Zetterstedt, 1828), *Eryngiofagamesomela* (Flor, 1861) and *Triozadispar* Löw, 1878 from Bulgaria are considered doubtful and these species are deleted from the list of the Bulgarian fauna, which now comprises 130 species from 33 genera and six families of jumping plant lice. This diversity is compared with the known data on Psylloidea in other countries of the Balkan Peninsula and Turkey and the Bulgarian psyllid fauna is discussed from the perspective of biogeography.

## Introduction

Jumping plant lice or psyllids (Hemiptera, Psylloidea) are small plant sap-feeding insects. They are usually narrowly host-specific and some species are of increasing economic importance as vectors of bacterial plant pathogens such as *Candidatus* *Phytoplasma* and *Liberibacter* spp. (Burckhardt et al. 2021, Moreno et al. 2021, Mauck et al. 2024). The superfamily Psylloidea comprises about 4,000 species worldwide, of which about 400 species occur in Europe (Ouvrard 2020). Although it is generally acknowledged that the Palaearctic fauna of the Psylloidea is comparatively well known, knowledge of the psyllids of the Balkans is still incomplete. For some countries, for example, Albania, Bosnia and Herzegovina, Kosovo, Montenegro and North Macedonia, information is scarce, scattered or even missing (Ouvrard 2020).

Bulgaria is a medium-sized country (app. 110,000 km^2^) located in the south-eastern part of the European continent and in the central and eastern part of the Balkan Peninsula. The country borders Romania to the north, Serbia and North Macedonia to the west, Greece and Turkey to the south and the Black Sea to the east. A significant part of the Bulgarian territory belongs to the Alpine-Himalayan Belt, which is characterised by the typical alternation of chain-like mountain ranges with old, highly fractured mountain massifs. The diverse relief is complemented by the flat Danubian Plain, which is connected to the vast Eastern European Plain. In terms of hydroclimate, the country occupies a transitional position between the southern parts of the temperate and subtropical climate, with the southern regions being influenced by the Mediterranean. Together with the physical-geographical position of Bulgaria, the combination of diverse relief, climate and water regime determines the high biological diversity of the country (Hubenov 1997, Kopralev et al. 2002).

The first data on the Bulgarian psyllids were published by Dimitar Joakimov as part of a pioneering list of the Hemiptera fauna of Bulgaria (Joakimov 1909), which included records of 17 species of psyllids. Although some of the psyllids collected by Joakimov have recently been found in the collections of the National Museum of Natural History in Sofia, the voucher specimens for the records published in Joakimov (1909) have probably been lost and cannot be revised (Pramatarova et al. 2023).

Thereafter, the psyllids were neglected by Bulgarian entomologists for a long time, until in the 1960s when Konov (1961), Tsalev (1961), Harizanov (1963), Harizanov (1964), Harizanov (1966a), Harizanov (1966b), Harizanov (1966c), Harizanov (1966d), Harizanov (1966e), Harizanov (1970) dealt with the biology and ecology of several economically important species: *Cacopsyllapyri* (Linnaeus, 1758), *C.pyrisuga* (Foerster, 1848), *C.picta* (Foerster, 1848), *C.pruni* (Scopoli, 1763), *Homotomaficus* (Linnaeus, 1758) and *Bactericeranigricornis* (Foerster, 1848).

At the same time, the Polish expert on Psylloidea, Sędzimir Maciej Klimaszewski, reported in a series of faunistic and taxonomic papers (Klimaszewski 1961a, Klimaszewski 1961b, Klimaszewski 1963, Klimaszewski 1964, Klimaszewski 1965, Klimaszewski 1970), which are summarised in the checklist of psyllids of the Palaearctic Region (Klimaszewski 1973), a total of 41 species from Bulgaria, including formal descriptions of four new species, two of which are still valid – *Craspedoleptabulgarica* Klimaszewski, 1961 and *Colposceniatraciana* (Klimaszewski, 1970). *Craspedoleptasalicorniae* Klimaszewski, 1961 was synonymised with *Rhodochlanisbicolor* (Scott, 1880) and *Homotomaviridis* Klimaszewski, 1961 was synonymised with *H.ficus* (Linnaeus, 1758) by Burckhardt (1989).

Another important contribution was made by the Czech hemipterologist, Pavel Lauterer. Mainly on the basis of material he had collected during several field trips to Bulgaria in the 1970–1980s and, in collaboration with other colleagues, he presented 14 species from Bulgaria, including *Agonoscenapistaciae* Burckhardt & Lauterer, 1989 and *Megagonoscenagallicola* Burckhardt & Lauterer, 1989, which were described as new to science (Harizanov and Lauterer 1968, Lauterer 1979, Lauterer 1982, Burckhardt and Lauterer 1989, Lauterer and Janíček 1990, Lauterer 1991, Lauterer 1993, Burckhardt and Lauterer 1997a, Lauterer and Malenovský 2002). In addition, Loginova (1978) described *Diaphorinalycii* Loginova, 1978, partly on the basis of material collected by P. Lauterer in Bulgaria. However, a larger part of P. Lauterer’s material from Bulgaria has remained unpublished to this day.

In the 1980s, Elżbieta Głowacka, another Polish entomologist, visited Bulgaria several times and published comprehensive lists of psyllids collected in the Western Rhodopes and the Pirin Mountains, which represented another major step in the knowledge of the Bulgarian fauna, as 26 species were recorded from the country for the first time (Głowacka and Harizanov 1983, Głowacka 1989).

Further faunistic data on psyllids from Bulgaria were scattered in the publications of Schaefer (1949), Vondráček (1953), Loginova (1974a), Gegechkori and Loginova (1990), Maryańska-Nadachowska et al. (1992), Kuznetsova et al. (1995), Burckhardt and Lauterer (1997b), Hodkinson and Bird (2000), Maryańska-Nadachowska (2002), Labina (2007), Labina et al. (2009), Labina et al. (2014), Vétek and Rédei (2009), Etropolska et al. (2011), Etropolska et al. (2015), Wonglersak et al. (2017), Nakabachi et al. (2020), Percy and Cronk (2020), Shapoval et al. (2021) and Nokkala et al. (2022).

Recently, Pramatarova et al. (2021) provided a list of psyllid species collected in the Sarnena Sredna Gora Mountains, Pramatarova et al. (2023) revised the historical material of the Psylloidea in the National Museum of Natural History in Sofia and Pramatarova et al. (2024) published DNA barcodes for 24 species of Aphalaridae from Bulgaria, four of which were recorded for the first time from the country.

Here, we summarise and critically re-evaluate all previously published data on psyllids from Bulgaria. We add many new data, based on a complete revision of the material of psyllids collected by Pavel Lauterer in the 1970–1980s and our own extensive fieldwork in Bulgaria in recent years. The main aim of this work is to provide an up-to-date checklist of the Psylloidea for Bulgaria. We also provide maps summarising the distribution of individual psyllid species in Bulgaria. Where available or relevant, we provide habitus photographs, illustrations of diagnostic characters and information on the nomenclature and host plants of the psyllids.

## Materials and methods

A large part of the specimens included in this paper were collected by us during faunistic surveys in different regions of Bulgaria in the years 1997–2023 by sweeping or direct search on host plants. This material is mainly deposited in the Zoological Collection of Sofia University in Sofia, Bulgaria (BFUS) and partly in the Moravian Museum in Brno, Czechia (MMBC). We have also reviewed and identified all specimens collected by Pavel Lauterer and his colleagues and students (R. Janíček, A. Merta and others) in Bulgaria in the 1970–1980s – this material is deposited in the MMBC. A small number of specimens of psyllids from Bulgaria deposited in the National Museum in Prague, Czechia (NMPC), have also been added to the dataset. In total, 10838 specimens of psyllids were directly examined by us. In view of the considerable size of the dataset, detailed information on the material examined is not given in the main text of the paper, but can be found together with previously published records of psyllids from Bulgaria in a GBIF dataset published in parallel (Pramatarova et al. 2025). The material from BFUS has also been included in an internal database prepared on the Specify collection management platform (Specify Collections Consortium 2021).

**Figure 1.**
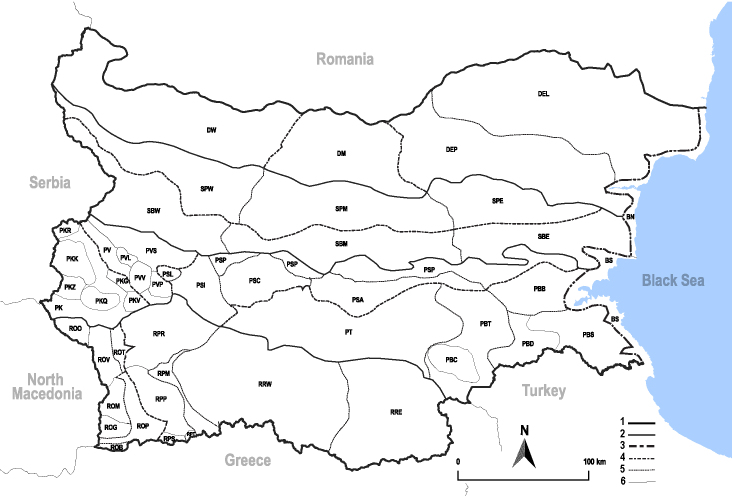
Map of the geographical regions of Bulgaria (Hubenov 1997). **1** - Bulgarian state border; **2** - border of regions; **3** - Black Sea border; **4** - border of subregions; **5** - border of smaller territorial units; **6** - border of mountains or basins. Abbreviations: **Danubian Plain (D)**: Western Danubian Plain (**DW**), Middle Danubian Plain (**DM**), Eastern Danubian Plain (**DE**) - Ludogorie-Dobrudzha District (**DEL**), Popovo-Provadiya District (**DEP**); **Stara Planina Range system (S)**: Predbalkan (**SP**) - Western Predbalkan (**SPW**), Middle Predbalkan (**SPM**), Eastern Predbalkan (**SPE**), Balkan (**SB**) - Western Balkan (**SBW**), Middle Balkan (**SBM**), Eastern Balkan (**SBE**); **Transitional region (P)**: Kraishte-Konyavo District (**PK**) - Rouy Mt. (**PKR**), Golo Burdo Mt. Verila (**PKG**), Verila Mt. (**PKV**), Kraishte (**PKK**), Zemenska Planina Mt. (**PKZ**), Konyavska Planina Mt. (**PKQ**), Vitosha District (**PV**) - Sofia Basin (**PVS**), Lyulin Mt. (**PVL**), Vitosha Mt. (**PVV**), Plana Mt. (**PVP**), Srednogorie-Podbalkan subregion (**PS**) - Podbalkan Basins (**PSP**), Sredna Gora Mts. (**PSS**), lhtimanska Sredna Gora (**PSI**), Lozenska Planina (**PSL**), Sushtinska Sredna Gora (**PSC**), Surnena Sredna Gora (**PSA**), Thracian Lowland (**PT**), Toundzha-Strandzha subregion (**PB**) - Sakar-Toundzha District (**PBT**), Sakar Mt. (**PBC**), Bakadzhik-Bourgas District (**PBB**), Strandzha-Dervent District (**PBD**), Strandzha Mt. (**PBS**); **Rila-Rhodopi Massif (R)**: Osogovo-Belasitsa group (**RO**) - Osogovska Planina Mt. (**ROO**), Vlahina Planina Mt. (**ROV**), Maleshevska Planina Mt. (**ROM**), Ograzhden Mt. (**ROG**), Belasitsa Mt. (**ROB**), Srednostrumska Valley (**ROS**), Boboshevo-Simitli Valley (**ROT**), Krupnik-Sandanski-Petrich Valley (**ROP**), Rila-Pirin group (**RP**) - Rila Mt. (**RPR**), Pirin Mt. (**RPP**), Slavianka Mt. (**RPS**), Sturgach Mt. (**RPT**), Mesta Valley (**RPM**), Rhodopi Mts (**RR**) - Western Rhodopi Mts (**RPW**), Eastern Rhodopi Mts (**RPE**); **Black Sea coast (B)**: Northern Black Sea coast (**BN**), Southern Black Sea coast (**BS**).

In order to give a concise overview of the distribution of the species in the main text, we give a list of the geomorphological units where the species have been recorded, based on the classification of Hubenov (1997), which is widely used in literature on the fauna of Bulgaria. For each species, we also provide a distribution map with these units (Fig. 1). In the absence of precise GPS coordinates for a record, estimated coordinates were used for georeferencing. If a locality was only given as a town or village, the centre was taken as the reference point. When additional information was provided (e.g. elevation, slope orientation etc.), these details were included in the process and points that corresponded to this information were selected. Published records that did not contain sufficient information, such as specimen deposition details or those that referred to species whose original identification we considered uncertain in light of current taxonomy, were classified in our distribution maps as 'published, requiring revision', meaning that these data require further verification or revision. The maps were created using QGIS 3.26.3 software (http://www.qgis.org).

The psyllid taxa are listed in alphabetical order according to the classification proposed by Burckhardt et al. (2021), the nomenclature following Ouvrard (2020) and Cho et al. (2022). For each species, a reference is given to its morphological diagnosis and illustrations in literature that can be used for identification. Unless otherwise stated, the general distribution is summarised from Ouvrard (2020). The distribution in Bulgaria is listed separately for published records and newly-studied material using the abbreviations of the districts from Hubenov (1997) (see the legend to Fig. 1 for the list of abbreviations). We explicitly list host plants only if they are associated with data from Bulgaria (for more details on host plants throughout the species' range, see Ouvrard (2020)). We use the term 'host plant' or 'confirmed host plant' only for plant taxa where a psyllid species is known to complete its life cycle from immature to adult (Burckhardt et al. 2014). In cases where only adults were collected, but no immatures, on a plant taxon that we nevertheless believe to be a potential host plant, we use the term 'probable host plant'. The nomenclature of the host plants is taken from POWO (2024). The morphological terminology in the taxonomic notes follows Ossiannilsson (1992). Photographs of live specimens of psyllids were taken with a Canon 70D DSLR camera with a Canon MP-E 65 mm macro lens and a Yongnuo YN-24EX macro flash or with a Nikon 5200 DSLR camera with a Laowa ultra macro lens and a Laowa Twin Flash KX-800. Most photographs of dry-mounted specimens were taken with a Keyence VHX-5000 digital microscope with VH-Z20T objective. Line drawings were made of slide-mounted specimens under a Leica DM5500B compound microscope with drawing tube. The latter instrument with Leica DFC320 camera and Leica Application Suite software was also used for detailed images of terminalia, temporarily slide-mounted in glycerol.

## Checklists

### Annotated checklist of Psylloidea in Bulgaria

#### Aphalaridae

Löw, 1879

9F6DD742-3D95-5FDB-873B-240920856451

#### Aphalarinae

Löw, 1879

1E4F9ACC-3507-5F9A-8B2D-36E59C48EFAB

#### Aphalara

Foerster, 1848

6FB7918B-529C-5B52-91C6-3FE836CBAAF9

#### Aphalara affinis

(Zetterstedt, 1828)

42FA4BA3-BA81-545C-B3F6-8220BB1BAC04

##### Distribution

**General distribution.** Northern, Central and Eastern Europe, Russia (European part, Siberia and Far East). **Distribution in Bulgaria** (Fig. 2b). Published records: RRW (Pramatarova et al. 2024). Material examined: RRW.

##### Diagnosis

**Adult** (Fig. 2a). Loginova (1961), Ossiannilsson (1992). **Fifth instar immature.** Ossiannilsson (1992).

#### Aphalara avicularis

Ossiannilsson in Ossiannilsson & Jansson, 1981

05EF6E22-4D24-59BD-97B7-FB4EB5CD5644

##### Feeds on

In Bulgaria, adults were collected on *Polygonumaviculare* L. (Głowacka 1989, Pramatarova et al. 2024), which is a confirmed host plant (Ossiannilsson 1992).

##### Distribution

**General distribution.** Europe (except Mediterranean), South Korea, Russian Far East (Cho et al. 2020). **Distribution in Bulgaria** (Fig. 3b). Published records: ROP, SBW (Głowacka 1989, Pramatarova et al. 2024). Material examined: BN, PSL, ROP, SBW.

##### Diagnosis

**Adult** (Fig. 3a) **and fifth-instar immature.** Ossiannilsson (1992).

#### Aphalara borealis

Heslop-Harrison, 1949

16F70D16-F796-5BD5-9B8B-24893684776B

##### Distribution

**Figure 4.**
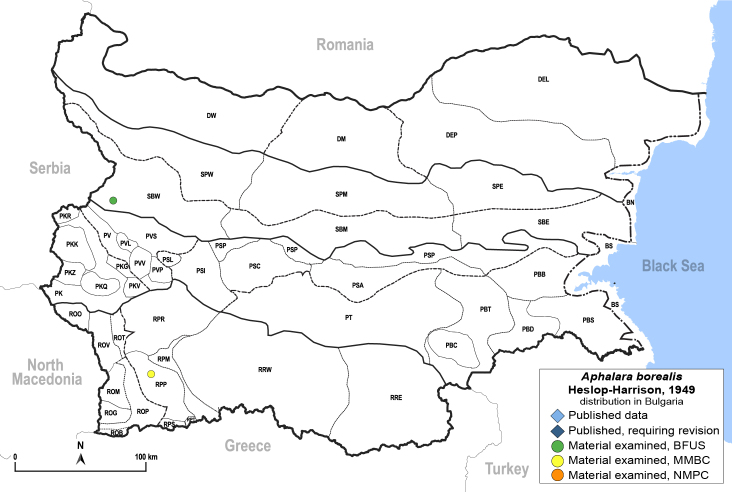
Distribution of *Aphalaraborealis* Heslop-Harrison, 1949 in Bulgaria.

**General distribution.** Northern, Central and Eastern Europe, Great Britain, Kazakhstan, Mongolia, Russia (European part, Siberia and Far East). **Distribution in Bulgaria** (Fig. 4). Material examined: RPP, SBW.

##### Notes

New record for Bulgaria.

##### Diagnosis

**Adult and fifth-instar immature.** Ossiannilsson (1992).

#### Aphalara exilis

(Weber & Mohr, 1804)

A4EB2746-6017-55D8-B516-32294C7D0739

##### Distribution

**Figure 5.**
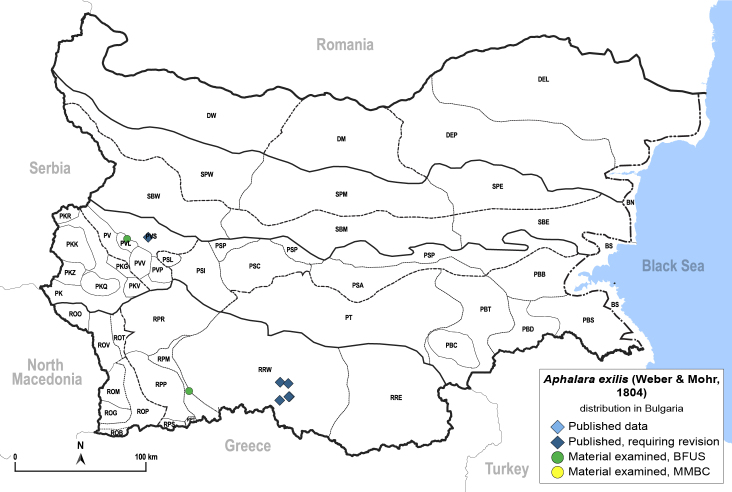
Distribution of *Aphalaraexilis* (Weber & Mohr, 1804) in Bulgaria.

**General distribution.** Western Palaearctic (Burckhardt and Lauterer 1997a). **Distribution in Bulgaria** (Fig. 5). Published records: PVS, RRW (Joakimov 1909, Klimaszewski 1965, Głowacka and Harizanov 1983). Material examined: PVL, RPM.

##### Notes

The species is quite rare in Bulgaria and only a few specimens have been collected recently. It is very likely that the previously published records from Bulgaria refer in fact to *A.nigrimaculosa* (Pramatarova et al. 2023).

##### Diagnosis

**Adult and fifth-instar immature.** Ossiannilsson (1992), Burckhardt and Lauterer (1997a).

#### Aphalara freji

Burckhardt & Lauterer, 1997

D20F1B90-119D-52B5-B3F3-FB95548A4A86

##### Distribution

**Figure 6.**
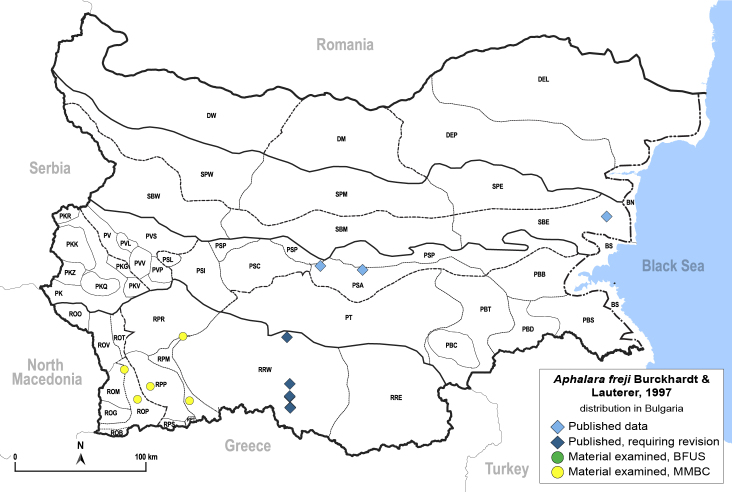
Distribution of *Aphalarafreji* Burckhardt & Lauterer, 1997 in Bulgaria.

**General distribution.** Western Palaearctic, Iran, South Korea. **Distribution in Bulgaria** (Fig. 6). Published records: ROP, RRW (Klimaszewski 1965, Głowacka and Harizanov 1983, Głowacka 1989; as *A.polygoni*); PSA, SBE (Pramatarova et al. 2021, Pramatarova et al. 2024). Material examined: PSA, ROM, ROP, RPM, RPP, RPR, SBE.

##### Notes

Before the revision by Burckhardt and Lauterer (1997a), *A.freji* was usually misinterpreted as *A.polygoni* auct. nec Foerster, 1848. Therefore, we assign here the data from Bulgaria published by Klimaszewski (1965), Głowacka and Harizanov (1983) and Głowacka (1989) as '*A.polygoni*' to *A.freji*. This is partly supported by the fact that the specimens of Głowacka and Harizanov (1983) and Głowacka (1989) were collected on *Polygonum* sp., while the host plants of *A.polygoni* Foerster, 1848 are *Rumex* spp. (Burckhardt and Lauterer 1997a).

##### Diagnosis

**Adult.** Burckhardt and Lauterer (1997a); Ossiannilsson (1992) as *A.polygoni*. **Fifth-instar immature.** Ossiannilsson (1992), as *A.polygoni*.

#### Aphalara maculipennis

Löw, 1886

EF6F2685-0313-53D0-9F08-25A81E5F9C5A

##### Distribution

**General distribution.** Palaearctic. **Distribution in Bulgaria** (Fig. 7b). Published records: without locality data (Pramatarova et al. 2023); SBW (Pramatarova et al. 2024). Material examined: SBW.

##### Diagnosis

**Adult** (Fig. 7a) **and fifth-instar immature.** Ossiannilsson (1992).

#### Aphalara nigrimaculosa

Gegechkori, 1981

0D523943-3B35-5E8C-9CAE-B35B71238E07

##### Feeds on

So far, the only known host plant of *Aphalaranigrimaculosa* was *Rumexalpinus* L. (Gegechkori 1981, Burckhardt and Lauterer 1997a). In Bulgaria, we collected adults and immatures on *Rumexacetosella* L., which represents a new host plant record for the species.

##### Distribution

**General distribution.** Bulgaria, Greece, Caucasus (Burckhardt and Lauterer 1997a). **Distribution in Bulgaria** (Fig. 8c). Published records: RPP (Głowacka 1989, as *A.sauteri*); RRW (Harizanov and Lauterer 1968, as *Aphalaraexilis*; *Pramatarova et al. 2024*); without locality data (Burckhardt and Lauterer 1997a, Pramatarova et al. 2023). Material examined: RPP, RPR, RRW, SBM.

##### Notes

The records of *Aphalarasauteri* Burckhardt, 1983 from th*e* Pirin Mts. published by Głowacka (1989) are assigned here to *A.nigrimaculosa*. This is based on other specimens from the same area from MMBC and BFUS, which were examined by us and all belong to *A.nigrimaculosa*. See also the comment under *A.sauteri* in the section 'Doubtful records' below.

##### Diagnosis

**Adult** (Fig. 8a). Gegechkori (1981), Burckhardt and Lauterer (1997a). **Fifth instar immature** (Fig. 8b).

#### Aphalara polygoni

Foerster, 1848

521738F2-9F0A-5618-8B8D-F5AC835439DA

##### Feeds on

*Rumex* sp. according to Głowacka and Harizanov (1983) and Głowacka (1989). The confirmed host plants are *Rumexacetosa* L., *R.acetosella* L. and *R.scutatus* L. (Ossiannilsson 1992, Burckhardt and Lauterer 1997a).

##### Distribution

**Figure 9.**
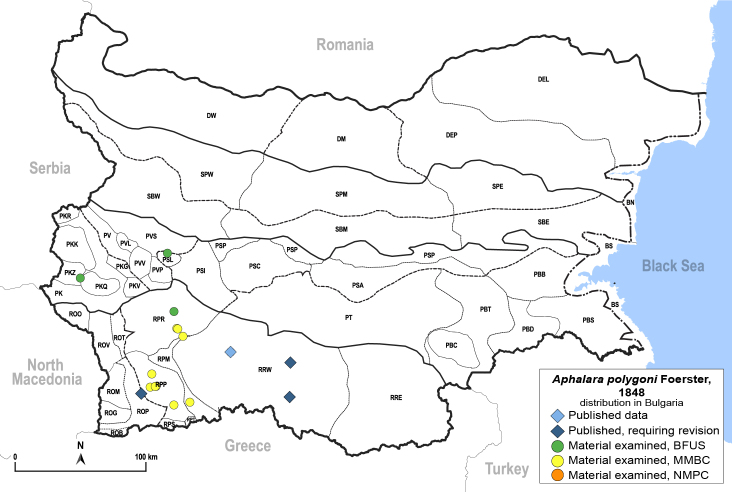
Distribution of *Aphalarapolygoni* Foerster, 1848 in Bulgaria.

**General distribution.** Northern, Central and Eastern Europe (Burckhardt and Lauterer 1997a). **Distribution in Bulgaria** (Fig. 9). Published records: RRW, ROP (Głowacka and Harizanov 1983, Głowacka 1989, as *A.rumicicola*); without locality data (Pramatarova et al. 2023); RRW (Pramatarova et al. 2024). Material examined: PKZ, PSL, RPM, RPP, RPR, RRW.

##### Diagnosis

**Adult and fifth-instar immature.** Ossiannilsson (1992), as *A.rumicicola*. According to Burckhardt and Lauterer (1997a), *A.rumicicola* Klimaszewski, 1966 is a junior synonym of *A.polygoni*.

#### Colposcenia

Enderlein, 1929

46CAF199-ACA6-58BD-8A90-A7D48CF1CDE1

#### Colposcenia aliena

(Löw, 1881)

573DE9B8-D9F8-537C-95AB-135F70BB3825

##### Feeds on

Oligophagous on *Tamarix* spp. (Burckhardt 1989, Spodek et al. 2017). In Bulgaria, we collected adults and immatures on *Tamarixramosissima* Ledeb., which is thus one of the confirmed host plants.

##### Distribution

**General distribution.** Southern Europe, North Africa, Ethiopia, Middle East, Caucasus, Central Asia, China. **Distribution in Bulgaria** (Fig. 10c). Published records: DEP (Pramatarova et al. 2024). Material examined: DEP.

##### Diagnosis

**Adult** (Fig. 10a). Loginova (1960); Tamanini (1977), as *C.italica*. **Fifth instar immature** (Fig. 10b).

#### Colposcenia bidentata

Burckhardt, 1988

4D125506-2548-5459-ADB5-E1591A034EA5

##### Feeds on

The genus *Tamarix* was indicated as the host plant of *Colposceniabidentata* (Burckhardt 1988). In Bulgaria, we collected adults on *Tamarixramosissima* Ledeb., a probable host plant species. It is possible that *C.bidentata* also develops on *T.tetrandra* Pall. ex. M.B., as mixed communities of both *Tamarix* species occur in Bulgaria.

##### Distribution

**General distribution.** Bulgaria and Turkey. **Distribution in Bulgaria** (Fig. 11b). Published records: RRE (Pramatarova et al. 2024). Material examined: ROM, ROP, RRE.

##### Diagnosis

**Adult** (Fig. 11a). Burckhardt (1988).

#### Colposcenia osmanica

Vondráček, 1953

EA7EC767-4CE5-538E-9076-487C9EDDAE63

Colposcenia kiritshenkoi Loginova, 1960: 57, **syn. nov.**

##### Feeds on

*Colposceniaosmanica* (as *C.kiritshenkoi*) has been recorded from *Tamarixnegevensis* Zohary, *T.parviflora* DC., *T.ramosissima* Ledeb., *T.senegalensis* DC. and *T.smyrnensis* Bunge (Gegechkori and Loginova 1990, Spodek et al. 2017). In Bulgaria, we collected adults on *T.ramosissima* and perhaps also *T.tetrandra* Pall. ex. M.B., which often grows together with the first species in mixed stands.

##### Distribution

**General distribution.** South-eastern Europe (Bulgaria, Ukraine, southern Russia), Middle East, Caucasus, Central Asia. **Distribution in Bulgaria** (Fig. 12d). Published records: ROP (Klimaszewski 1963, Klimaszewski 1965, Głowacka 1989), Bulgaria, without locality data (Gegechkori and Loginova 1990; as *C.kiritshenkoi*, *Maryańska-Nadachowska (2002)*); ROV (Pramatarova et al. 2024). Material examined: BN, BS, DW, PBT, ROM, ROP, ROV, RPP, RRE, SPE.

##### Diagnosis

**Adult** (Fig. 12a, b, c). Vondráček (1953), Loginova (1974b); Loginova (1960), Loginova (1974b), as *C.kiritshenkoi*. Based on numerous specimens collected at different times at several places in Bulgaria, we conclude here that *C.osmanica* and *C.kiritshenkoi* are seasonal forms of one and the same species, differing only in their colouration (*C.osmanica* - summer form, *C.kiritshenkoi* - overwintering form). Both colour forms were collected by us in Bulgaria (Fig. 12a, b). The male and female terminalia of specimens from Bulgaria are illustrated in Fig. 12c. Further details on the proposed synonymy can be found in the 'Discussion' section below.

#### Colposcenia traciana

(Klimaszewski, 1970)

124BE59B-4FD2-560F-A4F3-65901DC02B9C

##### Feeds on

As with all other *Colposcenia* species, the host plants of *C.traciana* belong to the plant genus *Tamarix* (Klimaszewski 1970). Seljak (2020) found it on *Tamarixgallica* L. in Slovenia. In Bulgaria, we collected adults on *Tamarixramosissima* Ledeb., which is another probable host species.

##### Distribution

**General distribution.** Slovenia (Seljak 2020), Bulgaria, Greece. **Distribution in Bulgaria** (Fig. 13b). Published records: BS (Klimaszewski 1965, as *Stigmaphalaratamaricis*), BS, PBS (Klimaszewski 1970, Pramatarova et al. 2024). Material examined: BS, ROP.

##### Notes

The type locality of *C.traciana* is in Bulgaria: Primorsko (Klimaszewski 1970).

##### Diagnosis

**Adult** (Fig. 13a). Klimaszewski (1970), as *Stigmaphalaratamaricistraciana*; Loginova (1974b).

#### Craspedolepta

Enderlein, 1921

C14F0B4A-37DB-5699-9D6D-144F9260A99F

#### Craspedolepta araneosa

Loginova, 1962

71CE6ECB-7C98-5DF7-B775-22EBD6DA021D

##### Feeds on

In Bulgaria, we collected adults on *Artemisiapedemontana* Balb., which represents a new probable host plant for the species. In Russia and Central Asia, *C.araneosa* was collected on *Artemisiaaustriaca* Jacq., *A.lercheana* Weber ex Stechm., *A.nitrosa* Weber ex Stechm., *A.schrenkiana* Ledeb. and *A.sublessingiana* (B. Keler) Krasch. ex Poljakov (Loginova 1962).

##### Distribution

**General distribution.** Russia (south-east of the European part: Orenburg Region), Central Asia (Kazakhstan, Uzbekistan, Tajikistan) (Loginova 1962, Gegechkori and Loginova 1990). **Distribution in Bulgaria** (Fig. 14b). Material examined: BN.

##### Notes

New record for Bulgaria and the Balkan Peninsula.

##### Diagnosis

**Adult** (Fig. 14a). Loginova (1962), Loginova (1963a), Loginova (1964). The Bulgarian specimens agree well with the original description (Loginova 1962) as regards the presence of long setae on the body (especially on the dorsal surface of the head and thorax), the pattern of the forewings and the details of the male paramere and the female terminalia. They differ in the denser and larger surface spinules on the forewing membrane, which are more similar to the condition in *C.artemisiae* than the illustration in the original description. More material is needed to assess the taxonomic significance of this character.

#### Craspedolepta artemisiae

(Foerster, 1848)

518354E7-C239-5307-B2DF-B1D9BB039842

##### Distribution

**Figure 15.**
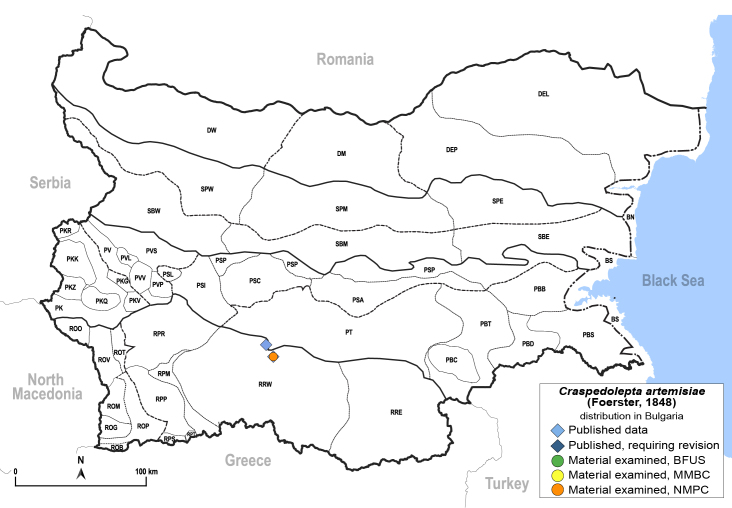
Distribution of *Craspedoleptaartemisiae* (Foerster, 1848) in Bulgaria.

**General distribution.** Central, South and Eastern Europe, Russia (European part, Siberia and Far East), Kazakhstan (Gegechkori and Loginova 1990). **Distribution in Bulgaria** (Fig. 15). Published records: RRW (Lauterer and Malenovský 2002). Material examined: RRW.

##### Diagnosis

**Adult.** Loginova (1963a), Klimaszewski (1975).

#### Craspedolepta bulgarica

Klimaszewski, 1961

91ED282A-37E4-5C3C-891A-1AC722CE0BDE

##### Feeds on

Collected on *Achillea* sp. in Bulgaria (Głowacka and Harizanov 1983, Pramatarova et al. 2024). Several *Achillea* spp. have been reported as confirmed or probable host plans in other countries (Burckhardt and Lauterer 1993, Conci et al. 1993, Lauterer 1993, AI-Khawaldeh et al. 1997, Seljak et al. 2008).

##### Distribution

**General distribution.** Central, South and Eastern Europe, Caucasus, Middle East and Central Asia (Gegechkori and Loginova 1990). **Distribution in Bulgaria** (Fig. 16b). Published records: PVS, RRW, SBW (Klimaszewski 1961a, Głowacka and Harizanov 1983, Pramatarova et al. 2024). Material examined: DEP, PKZ, PVS, ROM, ROP, RPP, RRE, RRW, SBW, SPW.

##### Notes

The type locality of *C.bulgarica* is Smoljan in the Rhodopi Mts. (Klimaszewski 1961a).

##### Diagnosis

**Adult** (Fig. 16a). Klimaszewski (1961a), Loginova (1963a), Klimaszewski (1975).

#### Craspedolepta conspersa

(Löw, 1888)

00309865-71F7-512A-B1F3-D848E1E51109

##### Distribution

**Figure 17.**
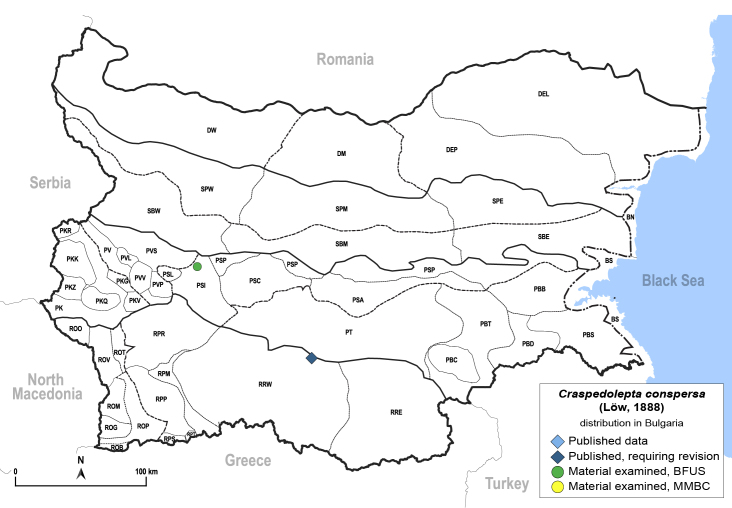
Distribution of *Craspedoleptaconspersa* (Löw, 1888) in Bulgaria.

**General distribution.** Central, South and Eastern Europe (Conci and Tamanini 1983, Cho et al. 2017a). **Distribution in Bulgaria** (Fig. 17). Published records: Bulgaria, without locality data (Klimaszewski 1973), RRW (Głowacka and Harizanov 1983). Material examined: PSI.

##### Notes

In his checklist of Palaearctic psyllids, Klimaszewski (1973) listed *C.conspersa* from Bulgaria, referring to his earlier work (Klimaszewski 1961a).⁠ However, this was probably a mistake, as Klimaszewski (1961a) studied specimens of *C.conspersa* only from Hungary. The species was again reported from Bulgaria by *Głowacka and Harizanov (1983)* and its occurrence in the country was also confirmed on the basis of our samples.

##### Diagnosis

**Adult.** Klimaszewski (1961a), Loginova (1963a), Loginova (1966), Conci and Tamanini (1983).

#### Craspedolepta innoxia

(Foerster, 1848)

6E31EFE8-8775-5A8E-AD08-BC5EF779D68C

##### Feeds on

Collected on *Daucuscarota* L. (Głowacka and Harizanov 1983), which is known as a host plant (Lauterer 1965).

##### Distribution

**General distribution.** Central, South and Eastern Europe, North Africa, Caucasus, Middle East, Central Asia (Gegechkori and Loginova 1990). **Distribution in Bulgaria** (Fig. 18b). Published records: ROM, RRW, SBW (Głowacka and Harizanov 1983, Lauterer 1991, Pramatarova et al. 2024). Material examined: PBB, PT, ROM, ROP, RRE, SBW, SPW.

##### Diagnosis

**Adult** (Fig. 18a). Vondráček (1957a), Loginova (1963a).

#### Craspedolepta latior

Wagner, 1944

D25927EE-C099-56D1-8132-9D3FB2945F5C

##### Feeds on

Collected on *Artemisia* sp. in Bulgaria (Głowacka and Harizanov 1983). Monophagous on *Artemisiavulgaris* L. in Europe (Ossiannilsson 1992, Burckhardt and Lauterer 2009).

##### Distribution

**Figure 19.**
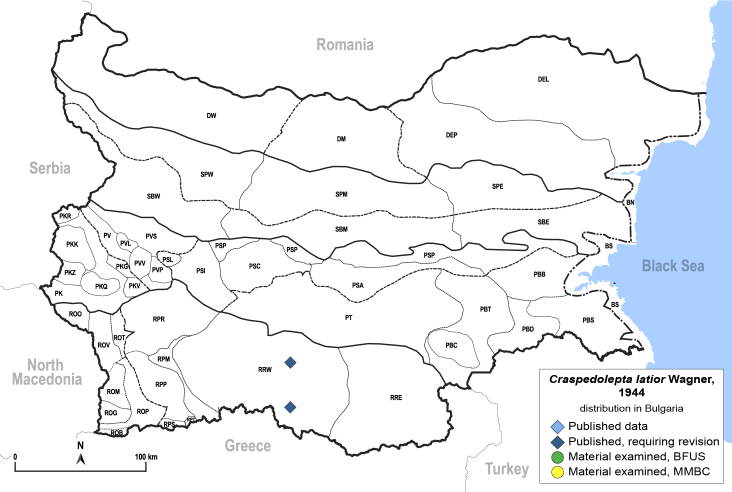
Distribution of *Craspedoleptalatior* Wagner, 1944 in Bulgaria.

**General distribution.** Central, Northern and Eastern Europe, Russia (European part, Siberia and Far East), Central Asia, Mongolia, Japan (Gegechkori and Loginova 1990). **Distribution in Bulgaria** (Fig. 19). Published records: RRW (Głowacka and Harizanov 1983).

##### Notes

The only record of *C.latior* from Bulgaria as published by Głowacka and Harizanov (1983) from the Western Rhodope Mts. requires a revision, as *Craspedolepta* spp. associated with *Artemisia* spp. have often been misidentified in the past. We have not been able to confirm the occurrence of *C.latior* in the country from our own collections or from P. Lauterer's material in MMBC.

##### Diagnosis

**Adult.** Loginova (1963a), Klimaszewski (1975), Ossiannilsson (1992), Burckhardt and Lauterer (2009). **Fifth-instar immature.** Ossiannilsson (1992).

#### Craspedolepta malachitica

(Dahlbom, 1851)

83D5F378-F912-5210-9672-63042620FA4D

##### Feeds on

In Bulgaria, adults were collected on *Artemisiaabsinthium* L. (Maryańska-Nadachowska et al. 1992, Pramatarova et al. 2024), a confirmed host plant (Ossiannilsson 1992, Burckhardt and Lauterer 2009).

##### Distribution

**General distribution.** Europe, Turkey, Caucasus, Central Asia, Russia (European part, Siberia, Far East), Mongolia. **Distribution in Bulgaria** (Fig. 20b). Published records: PKQ, PVS (Maryańska-Nadachowska et al. 1992, Pramatarova et al. 2024). Material examined: BN, PKQ, PVS, ROP, ROB.

##### Notes

*Craspedoleptamalachitica* was first published from Bulgaria by Maryańska-Nadachowska et al. (1992), which was overlooked by Pramatarova et al. (2024).

##### Diagnosis

**Adult** (Fig. 20a). Loginova (1963a), Klimaszewski (1975), Ossiannilsson (1992), Burckhardt and Lauterer (2009). **Fifth-instar immature.** Ossiannilsson (1992).

#### Craspedolepta nebulosa

(Zetterstedt, 1828)

FE7B4EEE-4E96-5BDA-9171-4FF6F07714EF

##### Feeds on

In Bulgaria, adults were collected on *Epilobiumangustifolium* L. (Głowacka and Harizanov 1983, Pramatarova et al. 2024), the only known host plant (Ossiannilsson 1992).

##### Distribution

**Figure 21.**
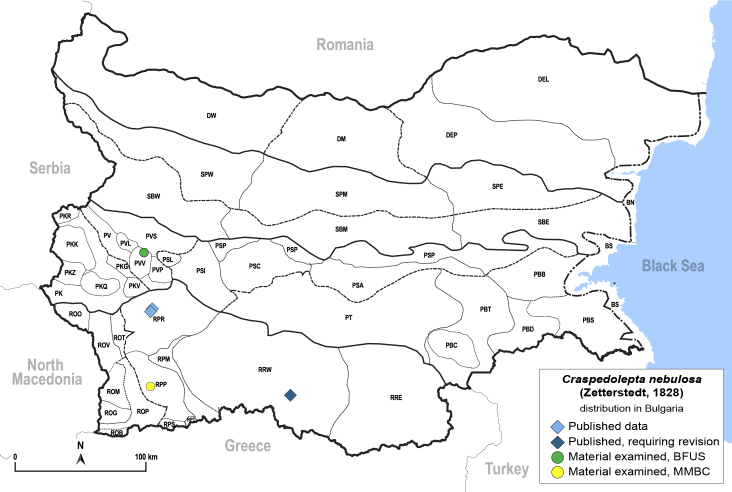
Distribution of *Craspedoleptanebulosa* (Zetterstedt, 1828) in Bulgaria.

**General distribution.** Holarctic. **Distribution in Bulgaria** (Fig. 21). Published records: RPR, RPW (Głowacka and Harizanov 1983, Pramatarova et al. 2024). Material examined: PVV, RPP, RPR.

##### Diagnosis

**Adult and fifth-instar immature.** Ossiannilsson (1992).

#### Craspedolepta nervosa

(Foerster, 1848)

D800454A-FFA4-5E64-AF3A-C580528A916E

##### Feeds on

We collected adults on *Achillea* sp. in Bulgaria (Pramatarova et al. 2024). Mainly confirmed from *Achilleamillefolium* L. in literature (Conci et al. 1993, Ossiannilsson 1992), but probably more widely oligophagous, as it was reported from *Achillea*, *Anthemis* and *Tanacetum* spp. (Gegechkori and Loginova 1990) and reared on *Cirsiumarvense* (Ossiannilsson 1992).

##### Distribution

**General distribution.** Palaearctic. **Distribution in Bulgaria**: (Fig. 22b). Published records: PVS, ROO, ROP, RRW, SBW (Joakimov 1909, Klimaszewski 1965, Głowacka and Harizanov 1983, Pramatarova et al. 2024). Material examined: DM, PVV, ROO, ROV, RPR, RRE, SBM, SBW.

##### Diagnosis

**Adult** (Fig. 22a) **and fifth-instar immature.** Ossiannilsson (1992).

#### Craspedolepta omissa

Wagner, 1944

893B4313-223E-5F45-B5B0-5523BA72C6C4

##### Feeds on

Adults were collected on *Artemisiavulgaris* L. in Bulgaria (Głowacka and Harizanov 1983, Pramatarova et al. 2024), which is a confirmed host plant (Conci et al. 1993).

##### Distribution

**General distribution.** Central and Eastern Europe, Turkey, Russia (European part, Siberia and Far East), Central Asia, Mongolia (Gegechkori and Loginova 1990). **Distribution in Bulgaria**: (Fig. 23b). Published records: RPR, RRW (Głowacka and Harizanov 1983, Pramatarova et al. 2024). Material examined: RPR, ROP.

##### Diagnosis

**Adult** (Fig. 23a). Loginova (1963a), Klimaszewski (1975).

#### Craspedolepta pontica

Dobreanu & Manolache, 1962

EAFA11C9-4F7A-5BD2-84EE-792806E00E07

##### Feeds on

According to Lauterer (1965) and Baeva (1985), the host plant of *C.pontica* is *Achilleamillefolium* L. According to Gegechkori and Loginova (1990), the host plants belong to the plant genera *Achillea*, *Anthemis* and *Tanacetum*. In Bulgaria, we collected adults of *C.pontica* on *Achilleaclypeolata* Sm. (Pramatarova et al. 2024), which is a probable host plant.

##### Distribution

**General distribution.** Central and South-eastern Europe, Caucasus, Middle East, Central Asia. **Distribution in Bulgaria** (Fig. 24b). Published records: ROM, RRW (Głowacka and Harizanov 1983, Pramatarova et al. 2024). Material examined: DEP, DM, PBT, ROM, ROP, ROV.

##### Diagnosis

**Adult** (Fig. 24a). Dobreanu and Manolache (1962), as *C.nervosapontica*; Loginova (1962), as *C.inarticulata* Loginova, 1962; Loginova (1964).

#### Craspedolepta subpunctata

(Foerster, 1848)

4C2B9BE8-879D-56D5-B57F-EB8987B38175

##### Feeds on

Adults were collected on *Epilobiumangustifolium* L. in Bulgaria (Głowacka and Harizanov 1983, Pramatarova et al. 2024), which is the only confirmed host plant (Ossiannilsson 1992).

##### Distribution

**Figure 25.**
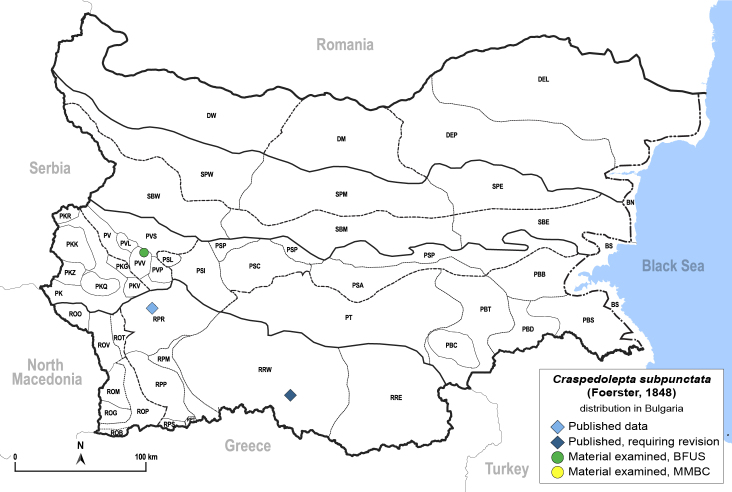
Distribution of *Craspedoleptasubpunctata* (Foerster, 1848) in Bulgaria.

**General distribution.** Holarctic. **Distribution in Bulgaria** (Fig. 25). Published records: RPR, RRW (Głowacka and Harizanov 1983, Pramatarova et al. 2024). Material examined: PVV, RPR.

##### Diagnosis

**Adult and fifth-instar immature.** Ossiannilsson (1992).

#### Eumetoecus

Loginova, 1961

0F477423-0493-5304-B4B6-3ED5602A985A

#### Eumetoecus kochiae

(Horváth, 1897)

6C9B6783-37B6-5ABB-9318-C17E4D84ACB9

##### Feeds on

In Bulgaria, adults were collected by P. Lauterer on *Bassialaniflora* (S.G.Gmel.) A.J.Scott (= *Kochiaarenaria* (Maerkl.) Roth), which was reported as a host plant in the original description (Horváth 1897). Loginova (1961) and Gegechkori and Loginova (1990) listed *Bassiaprostrata* (L.) Beck and *Camphorosmamonspeliaca* L. as host plants.

##### Distribution

**General distribution.** South-eastern Europe, Caucasus, Russia (European part, western Siberia), Middle East, Central Asia, Mongolia. **Distribution in Bulgaria** (Fig. 26b). Published records: Bulgaria, without locality data (Gegechkori and Loginova 1990). Material examined: BN.

##### Diagnosis

**Adult** (Fig. 26a). Loginova (1961).

#### Rhodochlanis

Loginova, 1964

8EE2342A-B160-5F1C-87BA-9F4DCAD6E825

#### Rhodochlanis bicolor

(Scott, 1880)

0D3204C3-C41A-5FD5-9697-8851AB5489C7

##### Feeds on

Oligophagous on *Petrosimonia*, *Salicornia*, *Salsola* and *Suaeda* spp. (Conci and Tamanini 1984b, Burckhardt 1989). In Bulgaria, adults were collected on *Salicorniaeuropaea* L. (Klimaszewski 1961a, Pramatarova et al. 2024), which is a probable host plant.

##### Distribution

**General distribution.** Southern and Eastern Europe, Caucasus, Middle East, Central Asia, Mongolia. **Distribution in Bulgaria** (Fig. 27b). Published records: BS (Klimaszewski 1961a), as *Craspedoleptasalicorniae*; (*Pramatarova et al. 2024*). Material examined: BS.

##### Notes

Klimaszewski (1961a) described *Craspedoleptasalicorniae* Klimaszewski, 1961 as a new species from the Bulgarian Black Sea Coast (Burgas). According to Burckhardt (1989), *C.salicorniae* is a junior synonym of *R.bicolor*.

##### Diagnosis

**Adult** (Fig. 27a). Burckhardt (1989); Klimaszewski (1961a), as *Craspedoleptasalicorniae*; Conci and Tamanini (1984b), as *R.salicorniae*. **Fifth-instar immature.** Conci and Tamanini (1984b), as *R.salicorniae*.

#### Rhinocolinae

Vondráček, 1957

51E9A80A-B21C-5222-9086-7804A3C1140D

#### Agonoscena

Enderlein, 1914

D16C1BFB-20A4-5419-ADE5-DE769208CA81

#### Agonoscena pistaciae

Burckhardt & Lauterer, 1989

8911C86E-CE01-55B6-A6F6-2054E2B30E9F

##### Feeds on

In Bulgaria, we collected adults on *Pistaciaterebinthus* L., which is a host plant confirmed by Rodrigo‐Gómez and Burckhardt (2023). In the Middle East, the preferred host plant of *A.pistaciae* is *Pistaciavera* L. (Burckhardt and Lauterer 1989, Rodrigo‐Gómez and Burckhardt 2023).

##### Distribution

**General distribution.** Spain, south-eastern Europe, Caucasus, Middle East, Central Asia (Rodrigo‐Gómez and Burckhardt 2023). **Distribution in Bulgaria** (Fig. 28b). Published records: ROM, RRE (Burckhardt and Lauterer 1989, Pramatarova et al. 2024). Material examined: ROM, RRE.

##### Notes

The type locality of *A.pistaciae* is in Bulgaria: Kresna Gorge (Burckhardt and Lauterer 1989).

##### Diagnosis

**Adult** (Fig. 28a) **and fifth-instar immature.** Burckhardt and Lauterer (1989), Rodrigo‐Gómez and Burckhardt (2023).

#### Agonoscena targionii

(Lichtenstein, 1874)

D493DE3C-6783-52AF-9CFD-135C56F79A9F

##### Feeds on

In Bulgaria, we collected adults on *Pistaciaterebinthus* L., which is one of the known host plants (Spodek et al. 2017, Seljak 2020). Throughout the Mediterranean Region, *A.targionii* is mainly found on *P.lentiscus* L. (Burckhardt and Lauterer 1989, Rodrigo‐Gómez and Burckhardt 2023), which does not occur in the Bulgarian flora.

##### Distribution

**General distribution.** Mediterranean, Middle East. **Distribution in Bulgaria** (Fig. 29b). Published records: ROM (Pramatarova et al. 2024). Material examined: ROM.

##### Diagnosis

**Adult** (Fig. 29a) **and fifth-instar immature.** Burckhardt and Lauterer (1989), Rodrigo‐Gómez and Burckhardt (2023).

#### Megagonoscena

Burckhardt & Lauterer, 1989

6A440918-74B4-5434-846A-89071BC82F7D

#### Megagonoscena gallicola

Burckhardt & Lauterer, 1989

5EF6F10B-C8DF-571B-9B94-3639307BD1D3

##### Feeds on

Inducing galls on *Pistaciaterebinthus* L. (= *P.palaestina* Boiss.) and *P.vera* L. (Burckhardt and Lauterer 1989). In Bulgaria, we collected adults and immatures on *P.terebinthus*, one of the confirmed host plants.

##### Distribution

**General distribution.** South-eastern Europe, Middle East. **Distribution in Bulgaria** (Fig. 30c). Published records: ROM, RRW (Burckhardt and Lauterer 1989, Pramatarova et al. 2024). Material examined: ROM, RRW.

##### Notes

The type locality of *M.gallicola* is in Bulgaria: Kresna (Burckhardt and Lauterer 1989).

##### Diagnosis

**Adult and fifth-instar immature** (Fig. 30a, b). Burckhardt and Lauterer (1989).

#### Megagonoscena viridis

(Baeva, 1963)

B366C642-0738-547F-9846-695ECE89B652

##### Feeds on

Oligophagous on *Pistaciaatlantica* Desf., *P.terebinthus* L. (= *P.palaestina* Boiss.) and *P.vera* L. (Burckhardt and Lauterer 1989). In Bulgaria, adults were collected on *P.terebinthus*.

##### Distribution

**General distribution.** Bulgaria, Caucasus, Middle East, Central Asia. **Distribution in Bulgaria** (Fig. 31b). Published records: ROM (Burckhardt and Lauterer 1989). Material examined: ROM.

##### Diagnosis

**Adult** (Fig. 31a) **and fifth-instar immature.** Burckhardt and Lauterer (1989).

#### Rhinocola

Foerster, 1848

6D9BA29F-C450-5BEA-9737-AD2BC57173DF

#### Rhinocola aceris

(Linnaeus, 1758)

C2462393-60C3-5A71-9F2D-8882333A066F

##### Feeds on

Adults were collected on *Acer* sp. in Bulgaria (Głowacka and Harizanov 1983, Pramatarova et al. 2024); *Acer* spp. are confirmed host plants (Ossiannilsson 1992).

##### Distribution

**General distribution.** Europe, Caucasus, Middle East. **Distribution in Bulgaria** (Fig. 32b). Published records: Bulgaria, without locality data (Gegechkori and Loginova 1990); PSL, RRW, SBW (Harizanov and Lauterer 1968, Głowacka and Harizanov 1983, Pramatarova et al. 2024). Material examined: BS, PSL, RRW, SBW, SPW.

##### Diagnosis

**Adult** (Fig. 32a) **and fifth-instar immature.** Ossiannilsson (1992).

#### Calophyidae

Vondráček, 1957

969869EE-1CA8-560F-90B9-409082B60DD7

#### Calophyinae

Vondráček, 1957

38B31092-CF43-5B65-A830-16E9FF283AC0

#### Calophya

Löw, 1879

CA1506B1-D774-50AE-8707-8F3C1BCD23DC

#### Calophya rhois

(Löw, 1877)

5E9B5A6A-2CE2-57C8-A090-A5C64228ABAC

##### Feeds on

In Bulgaria, adults and immatures were collected on *Cotinuscoggygria* Scop. (Klimaszewski 1964, Harizanov and Lauterer 1968, our data), which is the confirmed host plant (Burckhardt and Basset 2000, Burckhardt and Mühlethaler 2003).

##### Distribution

**General distribution.** Europe, Turkey, Caucasus, China. **Distribution in Bulgaria** (Fig. 33b). Published records: BN, RRW (Klimaszewski 1964, Harizanov and Lauterer 1968). Material examined: BS, ROP, RRW, SPW.

##### Notes

*Calophyarhois* was apparently introduced into several countries of central and western Europe together with its host plant, *Cotinuscogyggria*, which is often planted as an ornamental in parks and gardens (Burckhardt and Mühlethaler 2003, Mifsud et al. 2010). Burckhardt and Basset (2000) and Burckhardt and Mühlethaler (2003) hypothesised that *C.rhois* is native to East Asia and was introduced to Europe and the Caucasus. However, *Cotinuscogyggria* has a very wide range extending from the southern parts of Central Europe to central and southern China and is also considered native to the Bulgarian flora (Assyov et al. 2012, POWO 2024). *Calophyarhois* is common in southern Europe and has been listed amongst the native species of psyllids in Italy (Conci et al. 1996), Slovenia (Seljak 2020) and north-eastern Turkey (Burckhardt 1988). Therefore, it is possible that *C.rhois* is an autochthonous species occurring spontaneously in natural habitats at least in the southern parts of Bulgaria. On the other hand, *C.cogyggria* is also often planted as an ornamental plant in Bulgaria. Our record from SPW was made along a road near an agricultural field, where *C.cogyggria* and *C.rhois* were apparently introduced.

##### Diagnosis

**Adult** (Fig. 33a). Vondráček (1957a), Hodkinson and White (1979). **Fifth-instar immature.** Loginova (1968), White and Hodkinson (1982).

#### Carsidaridae

Crawford, 1911

B9FB27DA-6109-5732-BF84-BF692B2567B8

#### Homotominae

Heslop-Harrison, 1958

D285F876-68EE-5FC8-99BB-D997F87EADBF

#### Homotoma

Guérin-Méneville, 1844

0AB16104-F032-5C47-A60C-3FC4DC6BEA5B

#### Homotoma ficus

(Linnaeus, 1758)

01677807-4476-5119-8B81-03443192C9E5

##### Feeds on

In Bulgaria, Klimaszewski (1961b), Harizanov (1963) and we collected adults and immatures on *Ficuscarica* L., which is a confirmed host plant (Rapisarda 1989, Burckhardt 1989, Jerinić-Prodanović 2011).

##### Distribution

**General distribution.** Native to Mediterranean parts of Europe, North Africa, Ukraine, Caucasus, Middle East and Iran; alien in Great Britain, Central and south-eastern Europe, Serbia and USA. **Distribution in Bulgaria** (Fig. 34b). Published records: BS, PBB, PSP, ROP, ROT (Klimaszewski 1961b, Tsalev 1961, Harizanov 1963, Głowacka 1989). Material examined: BN, BS, ROM, ROP.

##### Notes

Klimaszewski (1961b) described *Homotomaviridis* Klimaszewski, 1961 partly based on material from Bulgaria (paratypes from Petrich). This taxon is considered a synonym of *H.ficus* (Burckhardt 1989). *Ficuscarica* L. is not native to the Bulgarian flora (POWO 2024), but it is cultivated as a fruit crop in yards and farms and naturalised in southern parts of the country and along the Black Sea coast (Assyov et al. 2012). *Homotomaficus* is consequently an alien species in Bulgaria.

##### Diagnosis

**Adult** (Fig. 34b). Loginova (1964), Tamanini (1965), Jerinić-Prodanović (2011). **Fifth-instar immature.** Rapisarda (1989), as *H.viridis*; Jerinić-Prodanović (2011).

#### Liviidae

Löw, 1879

FE2503E9-145D-5FB0-B4EE-99208E98DC2A

#### Euphyllurinae

Crawford, 1914

C817A86C-2DFD-51B0-85AE-E9DB930A9595

#### Euphyllura

Foerster, 1848

CDF16882-FD26-58E8-BACF-6156B6D5D914

#### Euphyllura phillyreae

Foerster, 1848

79046B90-159A-57CD-96C4-FBF735491313

##### Feeds on

According to Lauterer et al. (1986), *E.phillyreae* is oligophagous on the plant genera *Olea*, *Osmanthus* and *Phillyrea*. In Bulgaria, we collected adults and immatures on *Phillyrealatifolia* L., which is one of the confirmed host plants.

##### Distribution

**General distribution.** Mediterranean, Ukraine, south-western Russia, Caucasus, Middle East. **Distribution in Bulgaria** (Fig. 35b). Published records: Bulgaria, without locality data (Gegechkori and Loginova 1990). Material examined: BS, PBS, ROM, ROP, RRE.

##### Diagnosis

**Adult** (Fig. 35a). Lauterer et al. (1986). **Fifth-instar immature.** Loginova (1973).

#### Psyllopsis

Löw, 1879

250D2491-2D14-5052-AE5F-1D143A0797D0

#### Psyllopsis discrepans

(Flor, 1861)

EE42B6E3-F482-5443-860E-8972AF878890

##### Feeds on

Oligophagous on *Fraxinus* spp. (Conci and Tamanini 1990, Ossiannilsson 1992). In Bulgaria, adults and immatures were collected on *Fraxinus* sp. (Głowacka and Harizanov 1983).

##### Distribution

**Figure 36.**
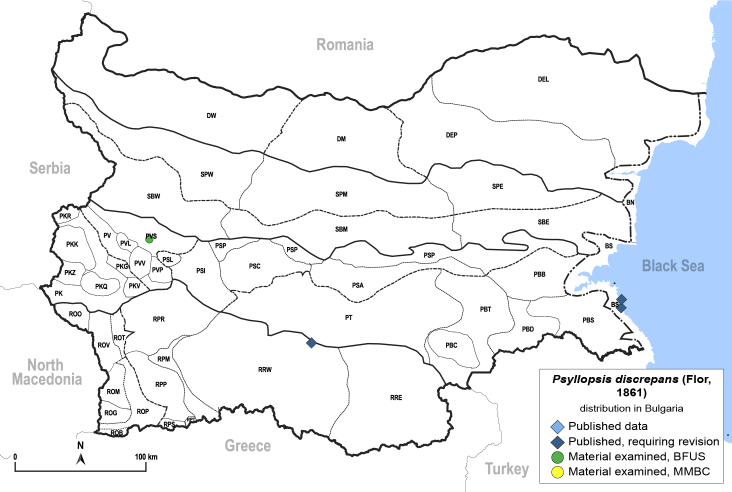
Distribution of *Psyllopsisdiscrepans* (Flor, 1861) in Bulgaria.

**General distribution.** Europe, Caucasus, alien in North America. **Distribution in Bulgaria** (Fig. 36). Published records: BS, RRW (Klimaszewski 1965, Głowacka and Harizanov 1983, Głowacka 1989). Material examined: PVS.

##### Diagnosis

**Adult and fifth-instar immature.** Loginova (1954), Ossiannilsson (1992).

#### Psyllopsis distinguenda

Edwards, 1913

84FB85C2-563F-53D3-B9C3-1242D1B0D58C

##### Feeds on

Oligophagous on *Fraxinusangustifolia* Vahl (including subsp. oxycarpa (M.Bieb. ex Willd.) Franco & Rocha Afonso) and *F.excelsior* L. (Conci and Tamanini 1990). In Bulgaria, we collected adults on *Fraxinus* sp.

##### Distribution

**Figure 37.**
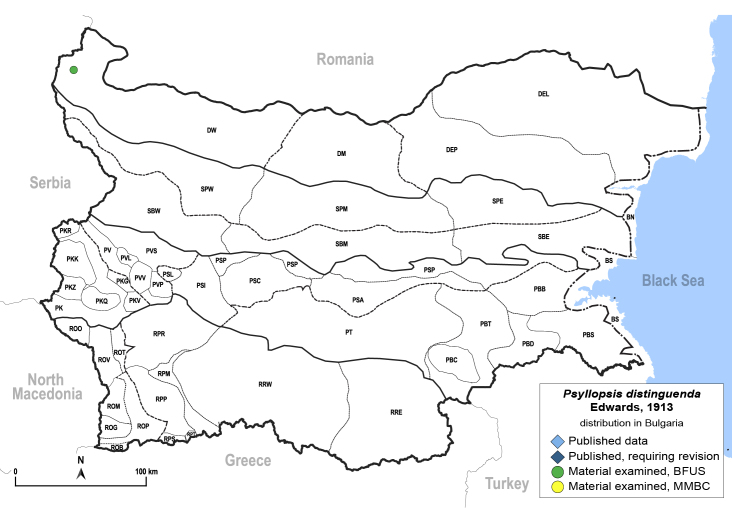
Distribution of *Psyllopsisdistinguenda* Edwards, 1913 in Bulgaria.

**General distribution.** Europe, Caucasus. **Distribution in Bulgaria** (Fig. 37). Material examined: DW.

##### Notes

New record for Bulgaria.

##### Diagnosis

**Adult.** Hodkinson and White (1979), Conci and Tamanini (1990). **Fifth-instar immature.** White and Hodkinson (1982).

#### Psyllopsis dobreanuae

Loginova, 1971

11FB15CF-2963-5419-95F2-E0F2CB140BBB

##### Feeds on

The only known host plant so far is *Fraxinusexcelsior* L. (Dobreanu and Manolache 1962, Conci and Tamanini 1990). In Bulgaria, we collected adults and immatures on *Fraxinus* sp.

##### Distribution

**General distribution.** Romania, North Macedonia, Moldova (Loginova 1971, Malenovský and Jerinić-Prodanović 2011). **Distribution in Bulgaria** (Fig. 38c). Material examined: PBS.

##### Notes

New record for Bulgaria.

##### Diagnosis

**Adult** (Fig. 38a). Dobreanu and Manolache (1962), as *P.meliphila*; Loginova (1971), Conci and Tamanini (1990). **Fifth-instar immature** (Fig. 38b).

#### Psyllopsis fraxini

(Linnaeus, 1758)

6E745C67-05A2-5C27-882E-734DB352DC46

##### Feeds on

Oligophagous on *Fraxinus* spp. (Conci and Tamanini 1990), on which we also collected adults in Bulgaria.

##### Distribution

**General distribution.** Europe (Conci and Tamanini 1990), alien in Australia, New Zealand and North America. **Distribution in Bulgaria** (Fig. 39b). Material examined: SPE.

##### Notes

New record for Bulgaria.

##### Diagnosis

**Adult** (Fig. 39a). Conci and Tamanini (1990), Ossiannilsson (1992). **Fifth-instar immature.** Ossiannilsson (1992), White and Hodkinson (1982).

#### Psyllopsis fraxinicola

(Foerster, 1848)

474A225B-53F0-5A36-A3D6-11B5AE42BFF9

##### Feeds on

Oligophagous on *Fraxinus* spp. (Conci and Tamanini 1990). In Bulgaria, adults and immatures were collected on *Fraxinusexcelsior* L. (Głowacka and Harizanov 1983, our data).

##### Distribution

**Figure 40.**
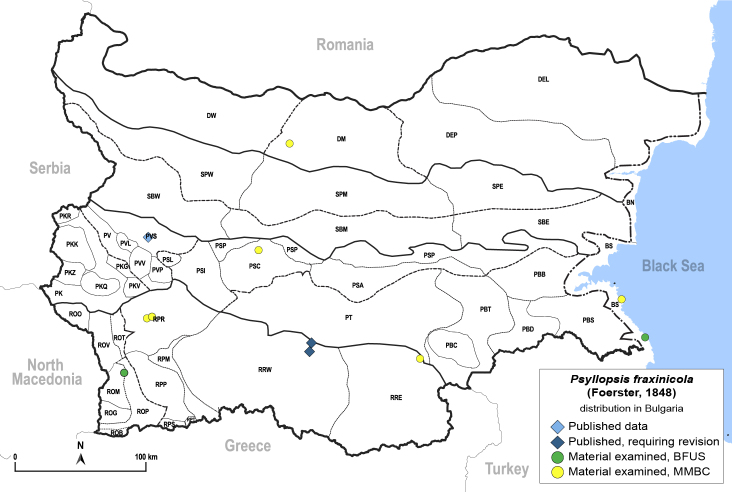
Distribution of *Psyllopsisfraxinicola* (Foerster, 1848) in Bulgaria.

Europe, North Africa, Caucasus, Middle East, Kazakhstan; alien in Australia, New Zealand, North and South America. **Distribution in Bulgaria** (Fig. 40). Published records: RRW, PVS (Głowacka and Harizanov 1983, Pramatarova et al. 2023). Material examined: BS, DM, PSC, PVS, ROM, RPR, RRE.

##### Diagnosis

**Adult and fifth-instar immature.** Conci and Tamanini (1990), Ossiannilsson (1992).

#### Psyllopsis machinosa

Loginova, 1963

0634E1BD-495B-573A-BB61-C03256D9F708

##### Feeds on

The known host plants are Fraxinusangustifoliasubsp.oxycarpa (M.Bieb. ex Willd.) Franco & Rocha Afonso (Gegechkori and Loginova 1990) and F.a.subsp.syriaca (Boiss.) Yalt. (Spodek et al. 2017). In Bulgaria, adults were collected on *Fraxinusexcelsior* L. (Lauterer 1993), which is another probable host plant species.

##### Distribution

**Figure 41.**
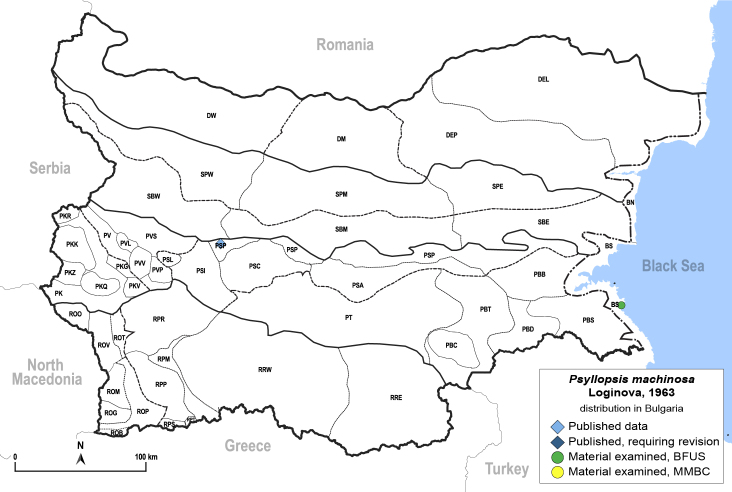
Distribution of *Psyllopsismachinosa* Loginova, 1963 in Bulgaria.

**General distribution.** South-eastern Europe, Middle East, Central Asia. **Distribution in Bulgaria** (Fig. 41). Published records: PSP (Lauterer 1993). Material examined: BS, PSP.

##### Diagnosis

**Adult.** Loginova (1963b).

#### Psyllopsis meliphila

Löw, 1881

85C4EE44-94D9-51F8-B411-05B84C875830

##### Feeds on

In Bulgaria, adults and immatures were collected on *Fraxinusornus* L. (Lauterer 1979), which is the only confirmed host plant species for *P.meliphila* (Conci and Tamanini 1990).

##### Distribution

**Figure 42.**
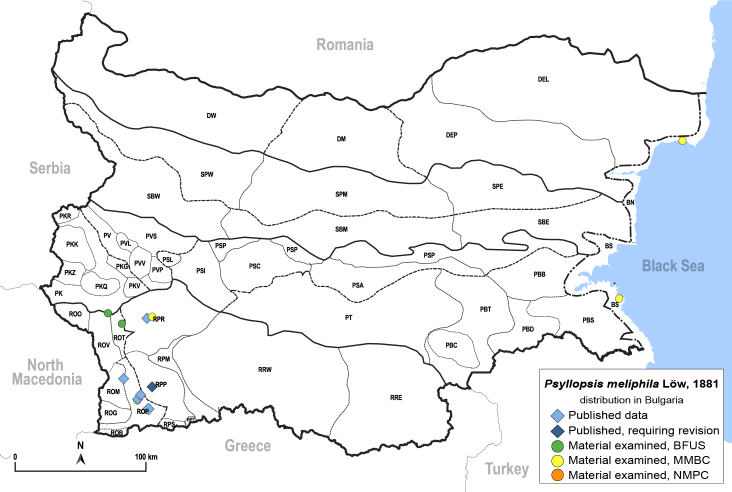
Distribution of *Psyllopsismeliphila* Löw, 1881 in Bulgaria.

**General distribution.** Southern parts of Central and south-eastern Europe (Conci and Tamanini 1990). **Distribution in Bulgaria** (Fig. 42). Published records: Bulgaria, without locality data (Maryańska-Nadachowska 2002), ROM, ROP, RPP, RPR (Lauterer 1979, Głowacka 1989). Material examined: BN, BS, ROM, ROP, ROT, ROV, RPP, RPR.

##### Diagnosis

**Adult.** Loginova (1971), Conci and Tamanini (1990). **Fifth-instar immature.** Rapisarda (1998).

#### Strophingia

Enderlein, 1914

71159AA1-AB4D-534B-8041-098808808A6F

#### Strophingia cinereae

Hodkinson, 1971

571E1764-1B60-5084-A83E-EA0662BCA221

##### Feeds on

In Bulgaria, we collected adults on *Ericaarborea* L., which is one of the confirmed host plants (Hodkinson 1981).

##### Distribution

**Figure 43.**
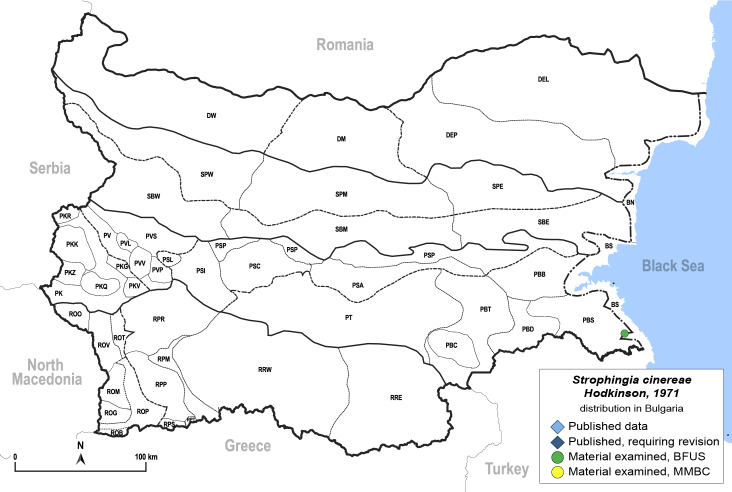
Distribution of *Strophingiacinereae* Hodkinson, 1971 in Bulgaria.

**General distribution.** Great Britain, Mediterranean parts of Europe and North Africa. **Distribution in Bulgaria** (Fig. 43). Material examined: PBS.

##### Notes

New record for Bulgaria. *Strophingiacinereae* has so far only been collected in the Strandzha Mountains near the Black Sea coast in the south-eastern part of the country in a habitat of nature conservation importance 'Strandzha heaths of tree heather (*Ericaarborea*) and common heather (*Callunavulgaris*)' (Gussev 2015).

##### Diagnosis

**Adult and fifth-instar immature.** Hodkinson (1981), Ossiannilsson (1992).

#### Strophingia ericae

(Curtis, 1835)

0163CC2D-B18C-588B-9195-3630C01098C9

##### Feeds on

In Bulgaria, we collected adults on *Callunavulgaris* (L.) Hull., which is a confirmed host plant (Hodkinson 1981).

##### Distribution

**General distribution.** Europe. **Distribution in Bulgaria** (Fig. 44b). Material examined: PBS.

##### Notes

New record for Bulgaria. *Strophingiaericae* is widespread in western, northern and central Europe (Lauterer 1976, Hodkinson 1981, Ossiannilsson 1992, Serbina et al. 2020, Seljak 2020). In Bulgaria, however, it is restricted to the Strandzha Mountains in the south-east of the country, the only region in the country where its host plant, *Callunavulgaris*, occurs (Assyov et al. 2012). The Strandzha heaths are a habitat of conservation importance (Gussev 2015), where *Callunavulgaris* occurs together with *Ericaarborea*, the host plant of *Strophingiacinereae*, which has also been recorded there (see above).

##### Diagnosis

**Adult** (Fig. 44a) **and fifth-instar immature.** Hodkinson (1981), Ossiannilsson (1992).

#### Liviinae

Löw, 1879

B0FA5E85-15B7-5F34-9A5D-AF8241ADE86C

#### Aphorma

Hodkinson, 1974

108DC27F-DA3E-5DDA-8E7F-089372420CC9

#### Aphorma lichenoides

(Puton, 1898)

B59DFBEE-D314-5E37-B786-55B692363523

##### Distribution

**Figure 45.**
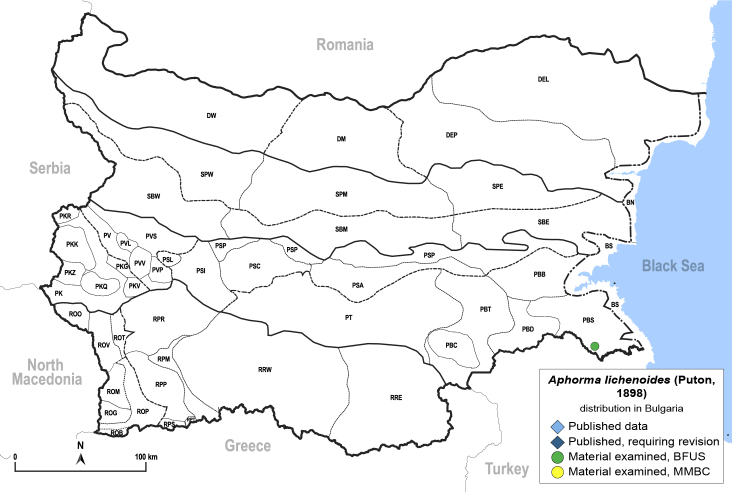
Distribution of *Aphormalichenoides* (Puton, 1898) in Bulgaria.

**General distribution.** Great Britain, southern parts of Europe, North Africa, Turkey. **Distribution in Bulgaria** (Fig. 45). Material examined: PBS.

##### Notes

New record for Bulgaria.

##### Diagnosis

**Adult.** Conci et al. (1983). **Fifth-instar immature.** Burckhardt and Mifsud (2003).

#### Camarotoscena

Haupt, 1935

5440500B-07DC-5320-90D4-81154D69B5D4

#### Camarotoscena speciosa

(Flor, 1861)

A17A6951-E327-57D3-B791-1C7948F9B6B0

##### Feeds on

Oligophagous on *Populus* spp. (Burckhardt et al. 2023). In Bulgaria, we collected adults on *Populusnigra* L., which is one of the confirmed host plants.

##### Distribution

**General distribution.** Europe, Caucasus, Middle East, Central Asia, China, Mongolia. **Distribution in Bulgaria** (Fig. 46b). Published records: RRW (Głowacka and Harizanov 1983). Material examined: PKZ, RPM.

##### Diagnosis

**Adult** (Fig. 46a) **and fifth-instar immature.** Ossiannilsson (1992).

#### Camarotoscena subrubescens

(Flor, 1861)

6B8B2893-4D0E-58EF-A3F0-8556D92938A9

##### Feeds on

In Bulgaria, adults were collected on *Populusnigra* L. by P. Lauterer. This is one of the confirmed host plant*s* (Burckhardt et al. 2023).

##### Distribution

**Figure 47.**
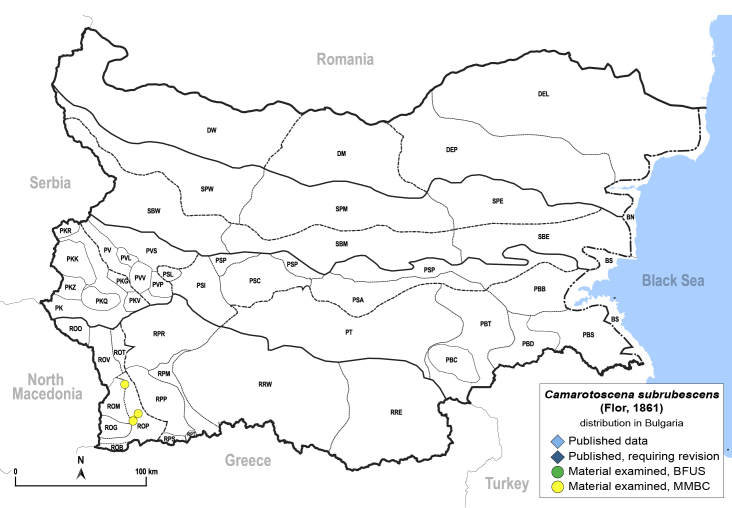
Distribution of *Camarotoscenasubrubescens* (Flor, 1861). in Bulgaria.

**General distribution.** Southern Europe, Turkey. **Distribution in Bulgaria** (Fig. 47). Material examined: ROM, ROP.

##### Notes

New record for Bulgaria.

##### Diagnosis

**Adult.** Loginova (1975).

#### Livia

Latreille, 1802

E38CD727-CD35-5739-BEDF-FBA881745A2F

#### Livia junci

(Schrank, 1789)

50F70E7F-88AE-5550-93CD-3285594CC0F6

##### Feeds on

Collected on *Juncus* sp. in Bulgaria (Głowacka and Harizanov 1983, Głowacka 1989). Oligophagous on a number of *Juncus* spp. (Hodkinson and Bird 2000).

##### Distribution

**General distribution.** Palaearctic. **Distribution in Bulgaria** (Fig. 48b). Published records: BS, PBT, PVS, PVV, ROP, RRW (Joakimov 1909, Vondráček 1953, Klimaszewski 1965, Głowacka and Harizanov 1983, Głowacka 1989, Pramatarova et al. 2023). Material examined: BS, PVV, ROM, ROP, RPM, RPR, RRE, SBE, SBM, SBW, SPE.

##### Diagnosis

**Adult** (Fig. 48a) **and fifth-instar immature.** Ossiannilsson (1992), as *L.juncorum*; Hodkinson and Bird (2000).

#### Livia mediterranea

Loginova, 1974

DBC1DE74-52DC-5A89-BF5B-295A3FB041CD

##### Distribution

**Figure 49.**
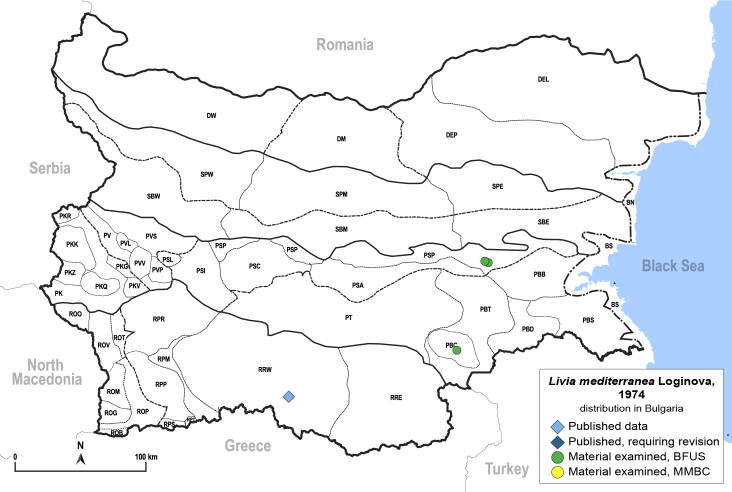
Distribution of *Liviamediterranea* Loginova, 1974 in Bulgaria.

**General distribution.** Southern Europe, North Africa, Caucasus, Middle East. **Distribution in Bulgaria** (Fig. 49). Published records: RRW (Harizanov and Lauterer 1968, as *L.crefeldensis*); Bulgaria, without locality data (Loginova 1974a, Hodkinson and Bird 2000). Material examined: PBC, PSP.

##### Notes

The type locality of *L.mediterranea* is in Georgia, but paratypes from Bulgaria (collected by Harizanov, without exact locality data) were also used for the species description (Loginova 1974a, Hodkinson and Bird 2000). The species seems to be rare in Bulgaria, as only a few specimens have been collected so far.

##### Diagnosis

**Adult.** Loginova (1974a), Hodkinson and Bird (2000).

#### Psyllidae

Latreille, 1807

F99E19F7-C59C-53CB-AFE5-77E4C042700D

#### Acizziinae

White & Hodkinson, 1985

D6E35E00-51D1-594D-A4F9-598861872F5D

#### Acizzia

Heslop-Harrison, 1961

386EE413-4590-5839-B97F-39D9F76EBAF7

#### Acizzia jamatonica

(Kuwayama, 1908)

01B3659B-5A09-5CA2-9CB4-09C96231CC5A

##### Feeds on

In Bulgaria, adults and immatures were collected on *Albiziajulibrissin* Durazz., which is the only confirmed host plant (Vétek and Rédei 2009, Harizanova et al. 2012).

##### Distribution

**General distribution.** Native to Eastern Asia, alien in Southern and Central Europe, Iran and North America. **Distribution in Bulgaria** (Fig. 50b). Published records: BS, PT, RRE (Vétek and Rédei 2009, Harizanova et al. 2012). Material examined: BN, BS.

##### Notes

*Acizziajamatonica* is now widespread and causing cosmetic damage on *Albiziajulibrissin*, planted as an ornamental in parks and private gardens in southern Bulgaria and along the Black Sea coast (Harizanova et al. 2012). The first evidence of this alien psyllid species in Bulgaria dates back to 2009 (Vétek and Rédei 2009).

##### Diagnosis

**Adult** (Fig. 50a) **and fifth-instar immature.** Burckhardt and Mühlethaler (2003).

#### Diaphorininae

Vondráček, 1951

600F1DAF-8CFC-5C80-A5F0-281119A3A0CC

#### Diaphorina

Löw, 1880

4F598517-EE2E-56F5-BAAD-62F1252A4140

#### Diaphorina lycii

Loginova, 1978

74681071-9B13-5016-81CE-EC6CAD6367ED

##### Feeds on

Oligophagous on several *Lycium* spp. (Burckhardt 1984). In Bulgaria, numerous adults were collected on *Lyciumbarbarum* L. (= *L.halimifolium* Mill.) (Loginova 1978, Nakabachi et al. 2020), which is one of the confirmed host plants.

##### Distribution

**General distribution.** Southern Europe, North Africa, Caucasus, Middle East, Central Asia, Mongolia. **Distribution in Bulgaria** (Fig. 51b). Published records: BN, BS, PSA, RRW (Loginova 1978, Nakabachi et al. 2020, Pramatarova et al. 2021). Material examined: BN, BS, PSA, RRW.

##### Notes

The type locality of *D.lycii* is in Turkmenistan (Burckhardt 1984). Paratypes from four localities in Bulgaria (collected by P. Lauterer and A. Merta, MMBC) were listed in the species description by Loginova (1978). In Bulgaria, *D.lycii* is monophagous on *Lyciumbarbarum*, which was imported to Bulgaria as an ornamental shrub, cultivated in parks and gardens for hedges, naturalised in 1900 and has since spread and become invasive in many parts of the country (Petrova et al. 2013). *Diaphorinalycii* should also be considered as an alien species in the Bulgarian fauna. The oldest documented specimens from Bulgaria were collected in 1973 (Loginova 1978).

##### Diagnosis

**Adult** (Fig. 51a). Loginova (1978), Burckhardt (1984). **Fifth-instar immature.** Burckhardt (1984).

#### Psyllinae

Latreille, 1807

85539A33-70A6-5BFD-97E1-9383540CCB42

#### Arytaina

Foerster, 1848

14CE4A5A-C8A1-54CA-B9AA-CF251C3CB756

#### Arytaina genistae

(Latreille, 1804)

5E8BBDD5-552F-5100-A80A-44DE4FEF5207

##### Distribution

**Figure 52.**
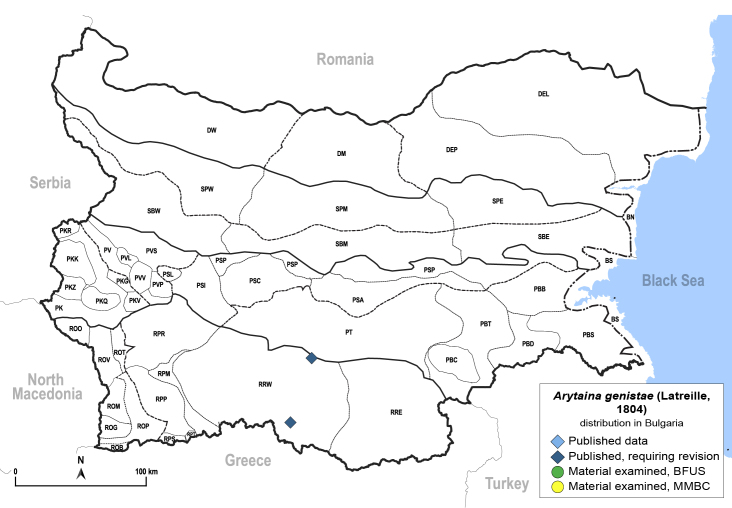
Distribution of *Arytainagenistae* (Latreille, 1804) in Bulgaria.

**General distribution.** Europe, alien in North America. **Distribution in Bulgaria** (Fig. 52). Published records: RRW (Klimaszewski 1965, Głowacka and Harizanov 1983).

##### Notes

*Arytainagenistae* is particularly widespread in the western parts of Europe. The only records for Bulgaria were published by Klimaszewski (1965) from the western Rhodope Mountains. These specimens should be re-examined to exclude the possibility that they do not belong to the similar *A.maculata*, which is common in the south-western part of the country.

##### Diagnosis

**Adult and fifth-instar immature.** Hodkinson and Hollis (1987), Ossiannilsson (1992).

#### Arytaina maculata

(Löw, 1886)

37B90BF3-C573-5838-AF66-4ECDAA2141DB

##### Feeds on

*Arytainamaculata* is narrowly oligophagous on *Chamaecytisus* spp., a taxonomically difficult group in the Balkans. *Arytainamaculata* was recorded by Klimaszewski (1970) on *Ch.ratisbonensis* (Schaeff.) Rotm. from Bulgaria (Yakoruda), by Andrianova and Klimaszewski (1983) on *Ch.borysthenicus* (Gruner) from south-western Russia, by Conci et al. (1993) from Italy on *Ch.spinescens* Rothm. and by *Malenovský et al. (2011)* on *Ch.austriacus* (L.) Link from Czechia. We collected many adult and immature specimens of *A.maculata* in south-western Bulgaria on Chamaecytisusaustriacussubsp.stefanoffii (Stoj.) Kuzmanov and many adults also on *Chamaecytisuseriocarpus* (Boiss.) Rothm. - the former is thus a newly-confirmed and the latter a probable host plant.

##### Distribution

**General distribution.** Central and south-eastern Europe. **Distribution in Bulgaria** (Fig. 53b). Published records: ROP, RPM, RPP, RRW, SBW (Klimaszewski 1965, Klimaszewski 1970, Głowacka and Harizanov 1983, Głowacka 1989, Lauterer 1993, Pramatarova et al. 2021, Pramatarova et al. 2023). Material examined: ROB, ROM, ROP, RPM, RPP, RPR, RPS, RRE, RRW, SBM, SBW.

##### Diagnosis

**Adult** (Fig. 53a). Hodkinson and Hollis (1987). **Fifth-instar immature.** Loginova (1976).

#### Arytainilla

Loginova, 1972

697BF621-7F83-5B72-80AB-B4AB2680E792

#### Arytainilla spartiicola

(Šulc, 1907)

AB13AF79-4211-545E-B702-01FEA3A684DD

##### Feeds on

The host plants known to date are *Cytisusscoparius* (L.) Link (Šulc 1907, Burckhardt 1983) and *C.decumbens* (Durande) Spach (Conci and Tamanini 1985a). Here, we report *Cytisusagnipilus* Velen. as a new host plant species confirmed by the presence of immatures in Bulgaria.

##### Distribution

**General distribution.** France, Germany, Switzerland, Italy. **Distribution in Bulgaria** (Fig. 54b). Material examined: SBW.

##### Notes

New record for Bulgaria and the Balkan Peninsula. *Arytainillaspartiicola* is a rare species, previously known only from a few localities in Europe (Šulc 1907, Burckhardt 1983, Conci and Tamanini 1985a).

##### Diagnosis

**Adult** (Fig. 54a) **and fifth-instar immature.** Conci and Tamanini (1985a).

#### Cacopsylla

Ossiannilsson, 1970

10D2460C-DBE5-56C2-B103-29863BA5927B

#### Cacopsylla abdominalis

(Meyer-Dür, 1871)

D2F75597-47D7-5713-A1D9-E97A36A1CF98

##### Distribution

**General distribution.** Europe, Caucasus, Central Asia, Mongolia, Russian Far East, South Korea (Cho et al. 2020). **Distribution in Bulgaria** (Fig. 55b). Material examined: PBS, RPM, RPP, RPR.

##### Notes

New record for Bulgaria.

##### Diagnosis

**Adult** (Fig. 55a). Loginova (1967), Lauterer and Burckhardt (1997).

#### Cacopsylla affinis

(Löw, 1880)

93470BB0-8F46-5EFC-8B8B-E4E984F328F2

##### Feeds on

Collected on *Crataegus* spp. in Bulgaria (Etropolska et al. 2015), which are confirmed host plants (Ossiannilsson 1992).

##### Distribution

**Figure 56.**
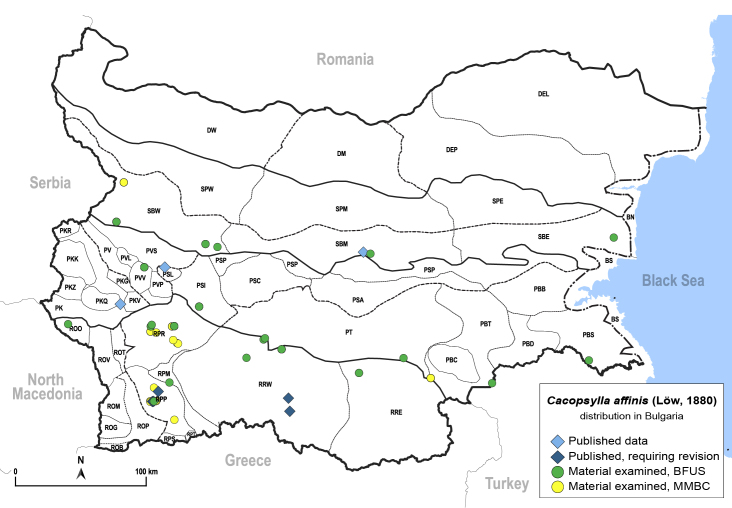
Distribution of *Cacopsyllaaffinis* (Löw, 1880) in Bulgaria.

**General distribution.** Europe, Turkey, Caucasus. **Distribution in Bulgaria** (Fig. 56). Published records: PKQ, PSL, RPP, RRW, SBM (Lauterer 1982, Głowacka and Harizanov 1983, Głowacka 1989, Etropolska et al. 2015). Material examined: PBS, PBT, PSI, PT, PVV, RPM, RPP, RPR, RRE, RRW, SBE, SBM, SBW.

##### Diagnosis

**Adult and fifth-instar immature.** Ossiannilsson (1992), Burckhardt (2010).

#### Cacopsylla albipes

(Flor, 1861)

344DED06-4D91-5AAB-B62B-43A3DAB592AF

##### Distribution

**Figure 57.**
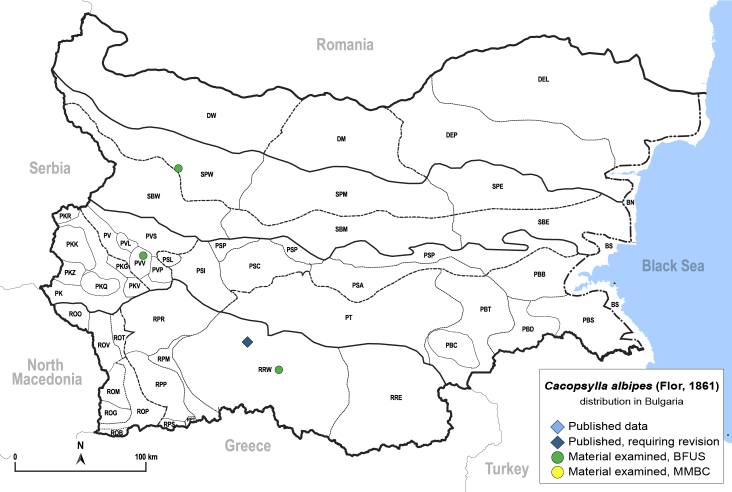
Distribution of *Cacopsyllaalbipes* (Flor, 1861) in Bulgaria.

**General distribution.** Europe (except north), Turkey, Caucasus. **Distribution in Bulgaria** (Fig. 57). Published records: RRW (Harizanov and Lauterer 1968, Głowacka and Harizanov 1983). Material examined: PVV, RRW, SPW.

##### Diagnosis

**Adult.** Vondráček (1957a), Hodkinson and White (1979), Burckhardt (2010). **Fifth-instar immature.** Burckhardt (2010).

#### Cacopsylla ambigua

(Foerster, 1848)

1B3D664F-7162-5B33-A643-0F48583DF63F

##### Feeds on

Collected on *Salix* sp. in Bulgaria (Głowacka and Harizanov 1983). Oligophagous on several *Salix* spp. (Ossiannilsson 1992, Lauterer and Burckhardt 1997, Percy and Cronk 2020).

##### Distribution

**Figure 58.**
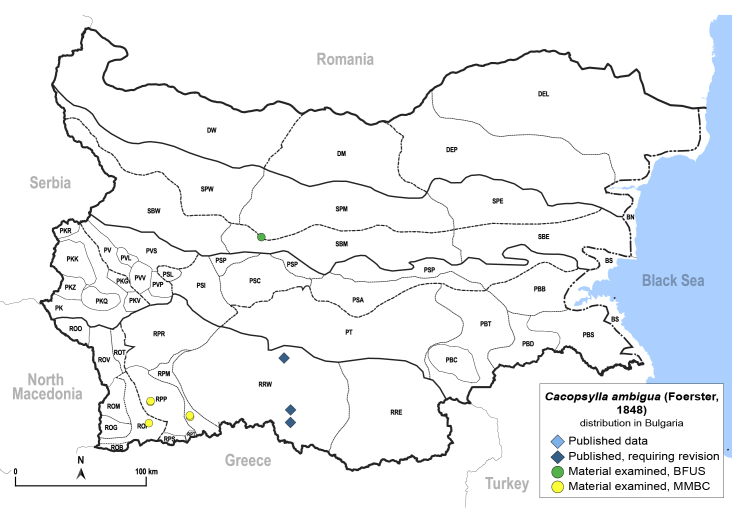
Distribution of *Cacopsyllaambigua* (Foerster, 1848) in Bulgaria.

**General distribution.** Palaearctic. **Distribution in Bulgaria** (Fig. 58). Published records: RRW (Klimaszewski 1965, Głowacka and Harizanov 1983). Material examined: ROP, RPM, RPP, SBM.

##### Diagnosis

**Adult.** Ossiannilsson (1992), Lauterer and Burckhardt (1997). **Fifth-instar immature.** Ossiannilsson (1992).

#### Cacopsylla bidens

(Šulc, 1907)

042408F8-0F87-5776-B358-8699E012A389

##### Feeds on

Oligophagous on *Pyrus*; the previously known hosts were *P.communis* L., *P.pyraster* (L.) Burgsd. and *P.syriaca* Boiss. (Burckhardt and Hodkinson 1986, Cho et al. 2017b). In Bulgaria, adults were collected on *P.communis* by Głowacka (1989) and Etropolska et al. (2015) and on *P.spinosa* Forrsk. by us; the latter is also a probable host.

##### Distribution

**Figure 59.**
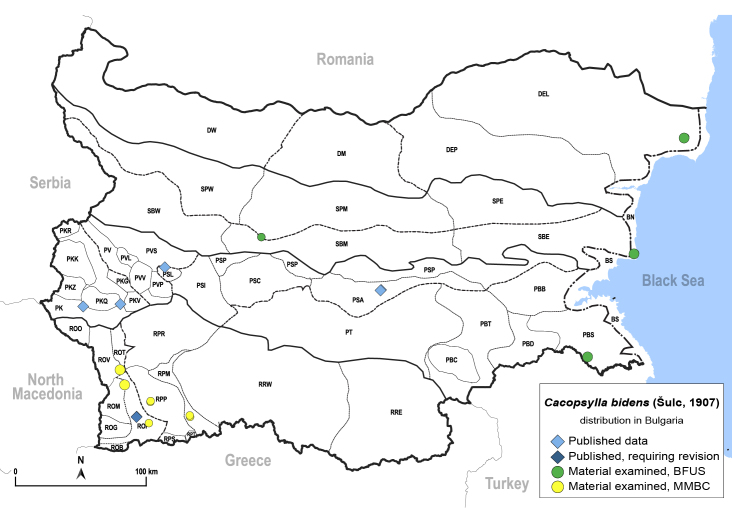
Distribution of *Cacopsyllabidens* (Šulc, 1907) in Bulgaria.

**General distribution.** Central and southern Europe, North Africa, Middle East, Central Asia, India, Mongolia, China; alien in South America. **Distribution in Bulgaria** (Fig. 59). Published records: PK, PKQ, PSA, PSL, ROP (Głowacka 1989, Etropolska et al. 2015, Pramatarova et al. 2023). Material examined: BN, DEL, PBS, PSA, ROM, ROV.

##### Diagnosis

**Adult.** Burckhardt and Hodkinson (1986), Burckhardt (2010), Valle et al. (2017). **Fifth-instar immature.** Burckhardt and Hodkinson (1986), Burckhardt (2010).

#### Cacopsylla breviantennata

(Flor, 1861)

0DB84C4D-2932-576C-BAFD-17DC17B83749

##### Distribution

**Figure 60.**
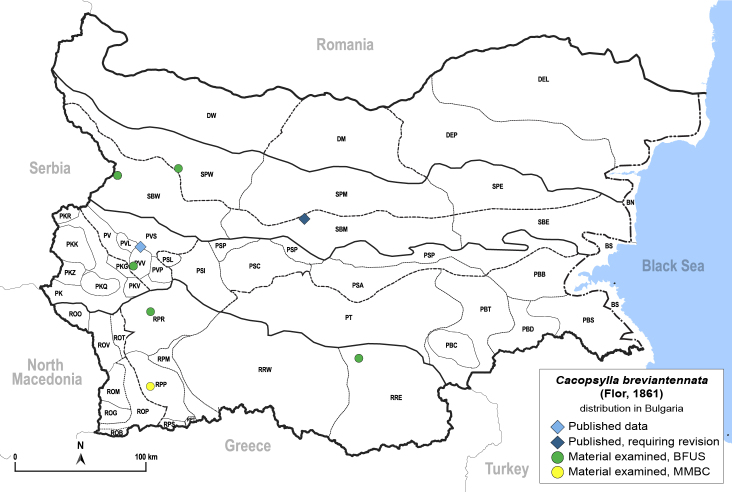
Distribution of *Cacopsyllabreviantennata* (Flor, 1861) in Bulgaria.

**General distribution.** Central and Southern Europe, North Africa, Turkey, Caucasus. **Distribution in Bulgaria** (Fig. 60). Published records: PVV, SBM (Klimaszewski 1970, Pramatarova et al. 2023). Material examined: PVV, RPP, RPR, RRE, SBW, SPW.

##### Diagnosis

**Adult.** Vondráček (1957a), Burckhardt (2010). **Fifth-instar immature.** Burckhardt (2010).

#### Cacopsylla brunneipennis

(Edwards, 1896)

2DA02349-FB50-5412-B56D-CBBF7E2CB052

##### Feeds on

Reported from *Salix* sp. in Bulgaria (Głowacka and Harizanov 1983). Oligophagous on several *Salix* spp. (Ossiannilsson 1992, Lauterer and Burckhardt 1997, Percy and Cronk 2020).

##### Distribution

**Figure 61.**
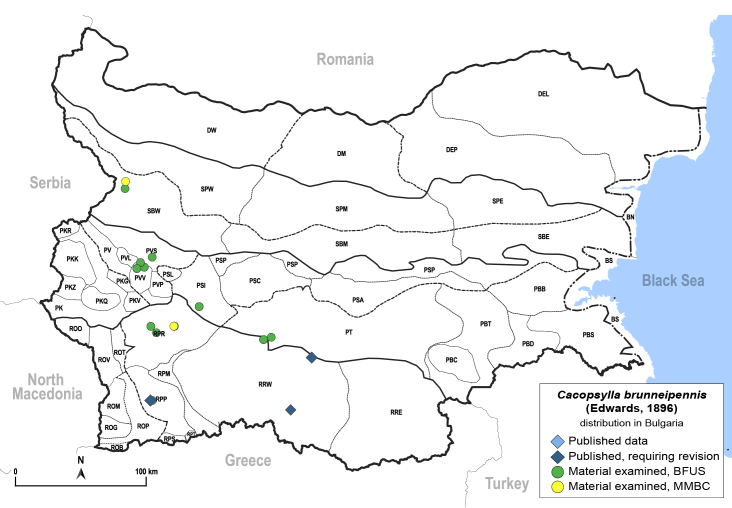
Distribution of *Cacopsyllabrunneipennis* (Edwards, 1896) in Bulgaria.

**General distribution.** Europe, Caucasus, Middle East, Central Asia. **Distribution in Bulgaria** (Fig. 61). Published records: RPP, RRW (Głowacka and Harizanov 1983, as *Cacopsyllaklapaleki*; Głowacka 1989). Material examined: PT, PVS, PVV, RPP, RPR, RRW, SBW.

##### Diagnosis

**Adult.** Ossiannilsson (1992), Lauterer and Burckhardt (1997). **Fifth-instar immature.** Ossiannilsson (1992).

#### Cacopsylla corcontum

(Šulc, 1909)

4489CC7C-CDD2-5E74-80BB-DC9A163C661C

##### Feeds on

Collected on *Sorbusaucuparia* L. in Bulgaria (Głowacka and Harizanov 1983), which is a confirmed host plant (Ossiannilsson 1992).

##### Distribution

**Figure 62.**
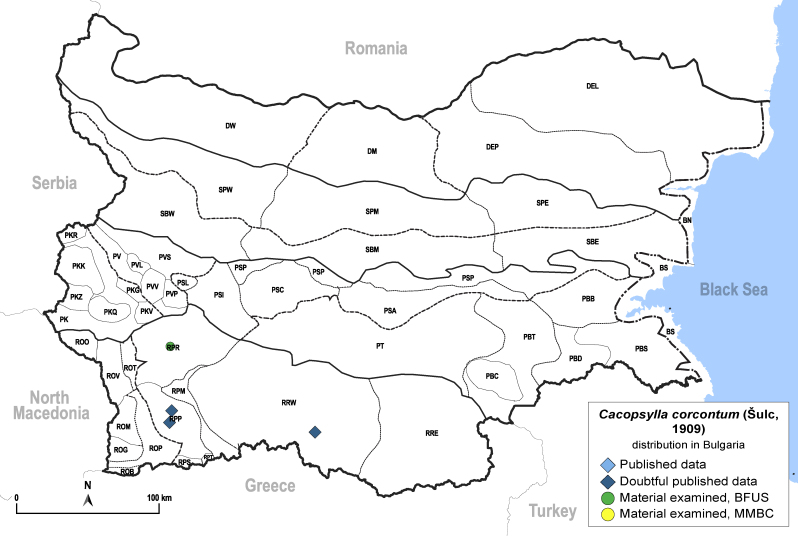
Distribution of *Cacopsyllacorcontum* (Šulc, 1909) in Bulgaria.

**General distribution.** Central and northern Europe. **Distribution in Bulgaria** (Fig. 62). Published records: RPP, RRW (Głowacka and Harizanov 1983, Głowacka 1989). Material examined: RPR.

##### Diagnosis

**Adult and fifth-instar immature.** Ossiannilsson (1992), Burckhardt (2010).

#### Cacopsylla crataegi

(Schrank, 1801)

1ECF955F-E6B9-5643-BEAD-B8BC18EF8BD4

##### Distribution

**Figure 63.**
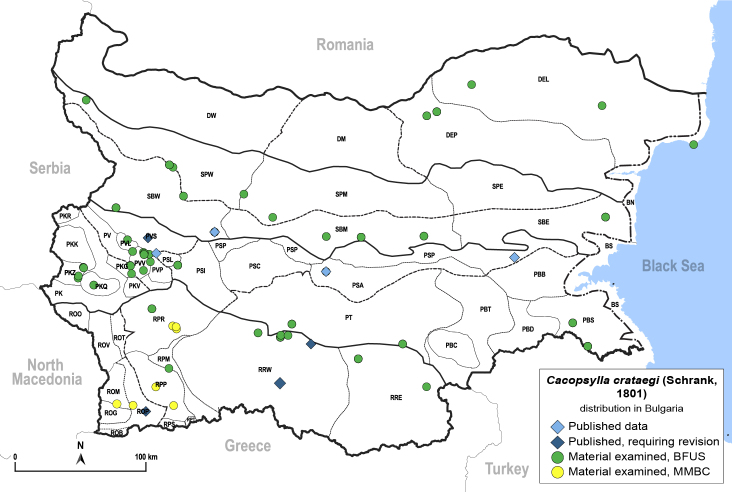
Distribution of *Cacopsyllacrataegi* (Schrank, 1801) in Bulgaria.

**General distribution.** Europe, North Africa, Caucasus, Middle East, India. **Distribution in Bulgaria** (Fig. 63). Published records: PSI, PSP, PVS, ROP, RRW, SBW (Joakimov 1909, Klimaszewski 1965, Harizanov and Lauterer 1968, Głowacka and Harizanov 1983, Pramatarova et al. 2021, Pramatarova et al. 2023). Material examined: BN, DEL, DEP, PBS, PKQ, PKZ, PSA, PSI, PSP, PT, PVL, PVV, ROM, ROP, RPM, RPP, RPR, RRE, RRW, SBE, SBM, SBW, SPM, SPW.

##### Diagnosis

**Adult and fifth-instar immature.** Ossiannilsson (1992), Burckhardt (2010).

#### Cacopsylla mali

(Schmidberger, 1836)

830A5C27-4335-5203-A392-F9CBCCADBA99

##### Feeds on

Collected on *Malusdomestica* (Suckow) Borkh. in Bulgaria (Głowacka and Harizanov 1983, Etropolska et al. 2015), which is a confirmed host plant (Ossiannilsson 1992).

##### Distribution

**Figure 64.**
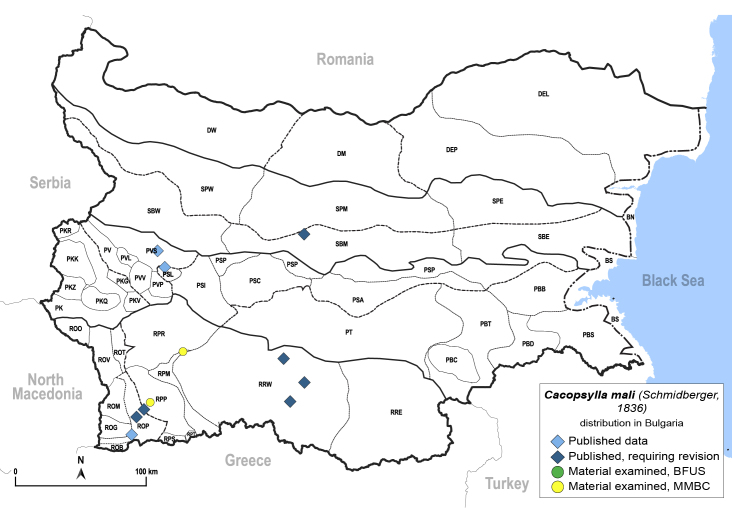
Distribution of *Cacopsyllamali* (Schmidberger, 1836) in Bulgaria.

**General distribution.** Palaearctic, alien in North America, South Africa and Australia. **Distribution in Bulgaria** (Fig. 64). Published records: PSL, PVS, ROP, RRW, SBM (Klimaszewski 1970, Głowacka and Harizanov 1983, Głowacka 1989, Etropolska et al. 2015). Material examined: RPP, RPR.

##### Diagnosis

**Adult and fifth-instar immature.** Ossiannilsson (1992), Burckhardt (2010).

#### Cacopsylla melanoneura

(Foerster, 1848)

AAB937E4-4B3B-51B4-8704-C0441CB07445

##### Feeds on

In Bulgaria, collected on *Crataegus* sp. (Głowacka and Harizanov 1983) and *Malusdomestica* (Suckow) Borkh. (Etropolska et al. 2015), which are confirmed host plants (Ossiannilsson 1992).

##### Distribution

**Figure 65.**
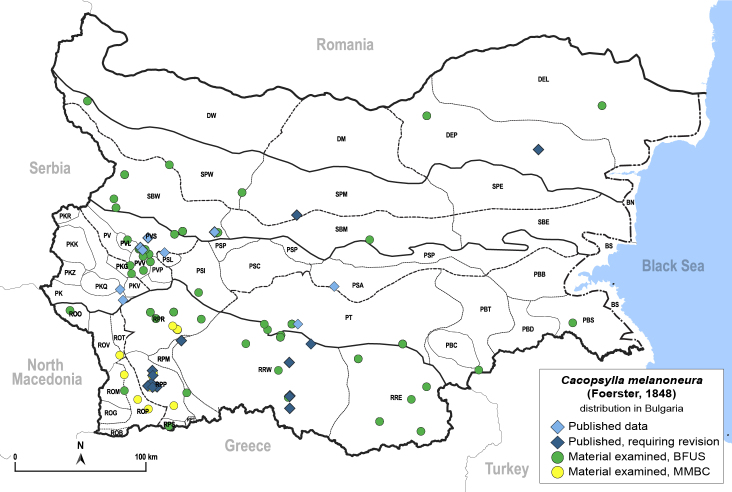
Distribution of *Cacopsyllamelanoneura* (Foerster, 1848) in Bulgaria.

**General distribution.** Palaearctic. **Distribution in Bulgaria** (Fig. 65). Published records: DEP, PK, PKQ, PSA, PSL, PT, PVL, PVS, PVV, RPM, RPP, RRW, SBM, SBW, SPM (Klimaszewski 1970, Głowacka and Harizanov 1983, Głowacka 1989, Etropolska et al. 2015, Pramatarova et al. 2021, Pramatarova et al. 2023). Material examined: DEL, DEP, PBS, PBT, PKQ, PSI, PVL, PVS, PVV, ROM, ROO, ROP, ROV, RPM, RPP, RPR, RPS, RRE, RRW, SBM, SBW, SPW.

##### Diagnosis

**Adult and fifth-instar immature.** Ossiannilsson (1992), Burckhardt (2010).

#### Cacopsylla myrtilli

(Wagner, 1947)

E073687F-A3C0-5DE1-9EF3-E563A91B09BD

##### Feeds on

In Bulgaria, collected on *Vacciniummyrtillus* L. (Głowacka 1989), which is a confirmed host plant (Ossiannilsson 1992).

##### Distribution

**Figure 66.**
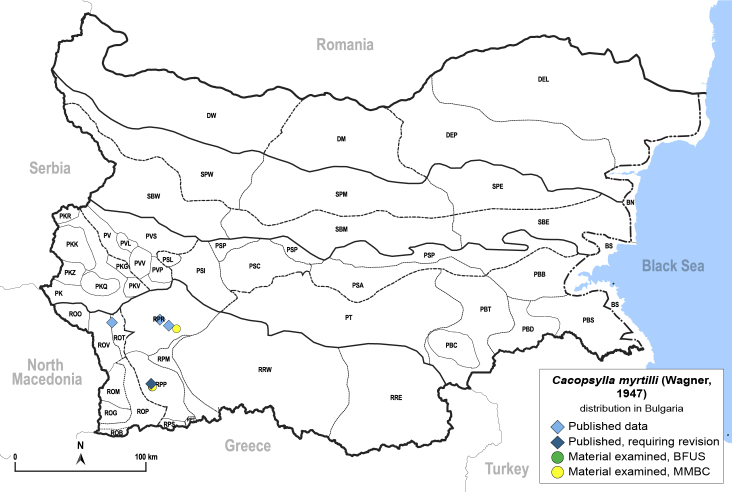
Distribution of *Cacopsyllamyrtilli* (Wagner, 1947) in Bulgaria.

**General distribution.** Central, northern and eastern Europe, Russia (European part, Siberia, Far East), Kazakhstan, North America. **Distribution in Bulgaria** (Fig. 66). Published records: ROV, RPP, RPR (Klimaszewski 1970, Głowacka 1989, Labina et al. 2009, Shapoval et al. 2021, Nokkala et al. 2022). Material examined: RPP, RPR.

##### Diagnosis

**Adult and fifth-instar immature.** Ossiannilsson (1992).

#### Cacopsylla nigrita

(Zetterstedt, 1828)

274C8A5B-78F7-5F08-8E62-9B401E445A1E

##### Distribution

**Figure 67.**
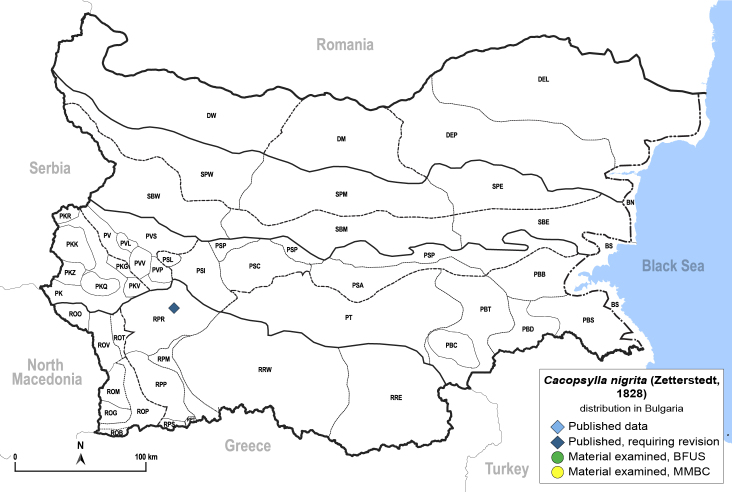
Distribution of *Cacopsyllanigrita* (Zetterstedt, 1828) in Bulgaria.

**General distribution.** Europe, Caucasus, Kazakhstan (Gegechkori and Loginova 1990). **Distribution in Bulgaria** (Fig. 67). Published records: RPR (Klimaszewski 1965).

##### Diagnosis

**Adult.** Ossiannilsson (1992), Lauterer and Burckhardt (1997). **Fifth-instar immature.** Ossiannilsson (1992).

#### Cacopsylla notata

(Flor, 1861)

9EB81898-C771-509B-9637-6782F13C76B1

##### Feeds on

In Bulgaria, Głowacka (1989) and the authors collected adults on *Pyrusspinosa* Forssk., which is one of the confirmed host plants (Burckhardt and Hodkinson 1986, Cho et al. 2017b).

##### Distribution

**Figure 68.**
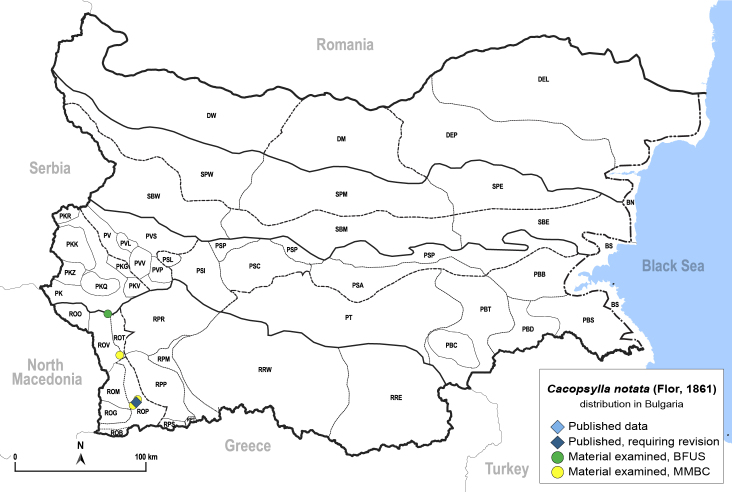
Distribution of *Cacopsyllanotata* (Flor, 1861) in Bulgaria.

**General distribution.** Southern Europe, Middle East. **Distribution in Bulgaria** (Fig. 68). Published records: ROP (Głowacka 1989). Material examined: ROP, ROV.

##### Diagnosis

**Adult and fifth-instar immature.** Burckhardt and Hodkinson (1986).

#### Cacopsylla peregrina

(Foerster, 1848)

473E1E1F-8518-5556-AF6D-76BF1191496B

##### Feeds on

Collected on *Crataegus* sp. in Bulgaria (Głowacka and Harizanov 1983); *Crataegus* spp. are confirmed host plants (Ossiannilsson 1992).

##### Distribution

**General distribution.** Palaearctic, alien in North America. **Distribution in Bulgaria** (Fig. 69b). Published records: PSA, PSC, PSP, PVS, ROP, RRW, SBW (Joakimov 1909, Głowacka and Harizanov 1983, Nokkala et al. 2003, Pramatarova et al. 2021). Material examined: BS, PBS, PSA, PSI, ROM, RPP, RPR, RRE, SBE, SBM, SBW, SPW.

##### Diagnosis

**Adult** (Fig. 69a) **and fifth-instar immature.** Ossiannilsson (1992), Burckhardt (2010).

#### Cacopsylla picta

(Foerster, 1848)

20B92440-C3CE-5E25-9C0A-6953D5F026B7

##### Feeds on

In Bulgaria, adults and immatures were collected on *Malusdomestica* (Suckow) Borkh. (Harizanov 1963, Harizanov 1966c, Głowacka and Harizanov 1983, Etropolska et al. 2015), which is a confirmed host plant (Ossiannilsson 1992).

##### Distribution

**Figure 70.**
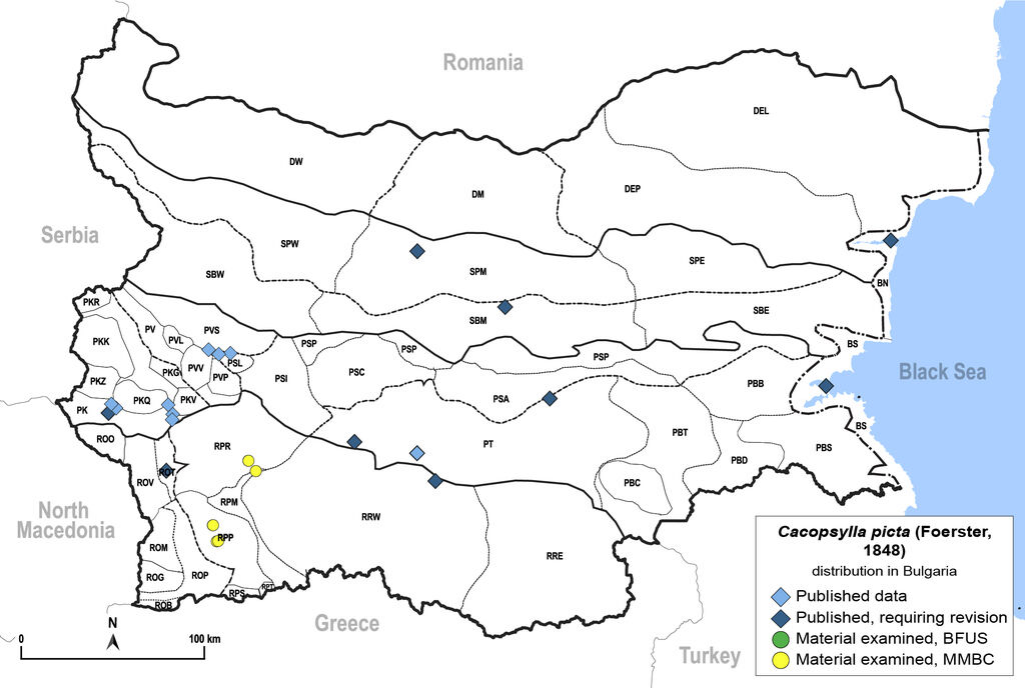
Distribution of *Cacopsyllapicta* (Foerster, 1848) in Bulgaria.

**General distribution.** Europe, Turkey. **Distribution in Bulgaria** (Fig. 70). Published records: BS, PKQ, PSL, PT, PVS, ROT, RRW, SBM, SPM (Harizanov 1963, Harizanov 1966c, Głowacka and Harizanov 1983, as *C.costalis*; Etropolska et al. 2011, Etropolska et al. 2015). Material examined: RPP, RPR.

##### Diagnosis

**Adult and fifth-instar immature.** Ossiannilsson (1992), as *C.costalis*; Burckhardt (2010).

#### Cacopsylla pruni

(Scopoli, 1763)

64ABE9C8-11E4-530E-95C9-703A4D88F4F7

##### Feeds on

Reported from *Prunuscerasifera* Ehrh., *P.insititia* L., *P.domestica* L. and *P.spinosa* L. in Bulgaria, which was also partially confirmed by immatures (Harizanov 1963, Harizanov 1966b, Etropolska et al. 2015).

##### Distribution

**General distribution.** Europe, North Africa, Caucasus, Middle East, Mongolia (Gegechkori and Loginova 1990, Sauvion et al. 2021). **Distribution in Bulgaria** (Fig. 71b). Published records: BS, PK, PKQ, PSL, PT, PVS, RPP, RRW (Harizanov 1963, Klimaszewski 1965, Harizanov 1966b, Głowacka and Harizanov 1983, Głowacka 1989, Etropolska et al. 2011, Etropolska et al. 2015, Pramatarova et al. 2023). Material examined: BN, PBS, PBT, PKQ, PKZ, PSC, PSI, PSP, PT, PVL, PVS, PVV, ROO, ROP, RPM, RPP, RPR, RPS, RRE, RRW, RRW, SBE, SBW, SPW.

##### Diagnosis

**Adult** (Fig. 71a) **and fifth-instar immature.** Ossiannilsson (1992), Burckhardt (2010). According to Sauvion et al. (2021), *C.pruni* is a complex of two cryptic species, informally referred to as '*C.pruni* A' and '*C.pruni* B', which can be distinguished by molecular characters. Specimens from Bulgaria have not yet been identified as either A or B. Only *C.pruni* B is known from the Balkans (Sauvion et al. 2021).

#### Cacopsylla pulchella

(Löw, 1877)

FF02D16A-1106-52EB-A3F1-16F22B683B5D

##### Feeds on

We collected adults and immatures on *Cercissiliquastrum* L., the only confirmed host plant (Burckhardt 1999).

##### Distribution

**General distribution.** Southern and south-eastern Europe, Middle East, alien in the Azores, Iberian Peninsula, Great Britain, Central Europe and Belarus. **Distribution in Bulgaria** (Fig. 72b). Material examined: BN, RRE.

##### Notes

New record for Bulgaria. The host plant of *C.pulchella*, *Cercissiliquastrum* is native to Bulgaria (POWO 2024). However, our specimens of *C.pulchella* were collected on ornamental trees in city gardens.

##### Diagnosis

**Adult** (Fig. 72a). Loginova (1964), Hodkinson and White (1979). **Fifth-instar immature.** White and Hodkinson (1982), Burckhardt (1999).

#### Cacopsylla pulchra

(Zetterstedt, 1840)

A2385B0D-BE0F-5D66-8A78-A6C43F3ABF14

##### Feeds on

Collected on *Salix* sp. in Bulgaria (Głowacka and Harizanov 1983), Oligophagous on several *Salix* spp. (Ossiannilsson 1992, Lauterer and Burckhardt 1997, Percy and Cronk 2020).

##### Distribution

**Figure 73.**
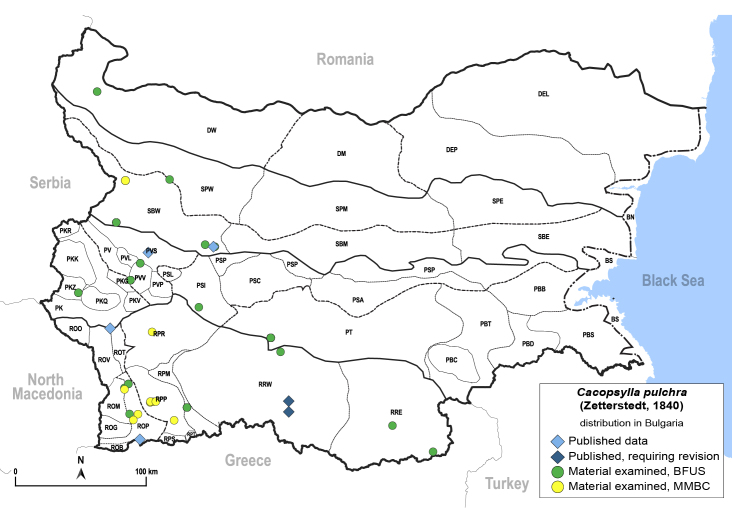
Distribution of *Cacopsyllapulchra* (Zetterstedt, 1840) in Bulgaria.

**General distribution.** Palaearctic**. Distribution in Bulgaria** (Fig. 73). Published records: PK, PVS, ROP, RRW, SBW (Głowacka and Harizanov 1983, Percy and Cronk 2020, Pramatarova et al. 2021, Pramatarova et al. 2023). Material examined: DW, PKZ, PSI, PT, PV, PVS, PVV, ROM, ROP, RPM, RPP, RPR, RRE, RRW, SBW.

##### Diagnosis

**Adult.** Lauterer and Burckhardt (1997), Ossiannilsson (1992). **Fifth-instar immature.** Ossiannilsson (1992).

#### Cacopsylla pyri

(Linnaeus, 1758)

853933FE-99D4-51A3-B7E6-7739251A46E9

##### Feeds on

In Bulgaria, adults and immatures were collected on *Pyruscommunis* L. (Harizanov 1963, Harizanov 1966a, Harizanov 1966d, Etropolska et al. 2015), which is a confirmed host plant (Burckhardt and Hodkinson 1986, Cho et al. 2017b).

##### Distribution

**Figure 74.**
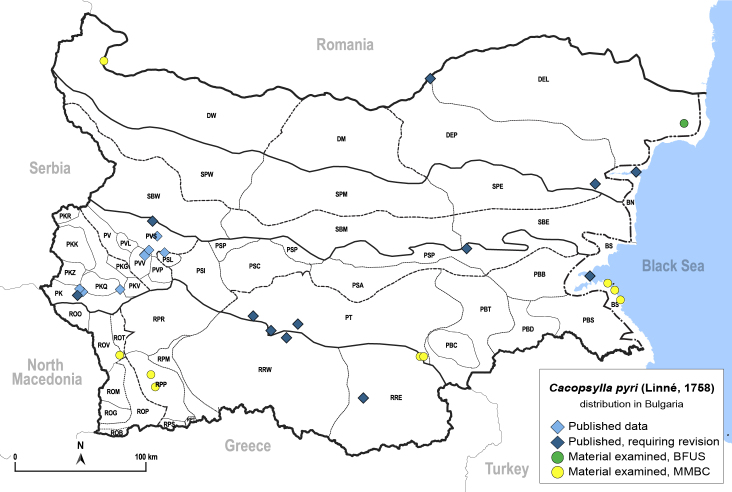
Distribution of *Cacopsyllapyri* (Linnaeus, 1758) in Bulgaria.

**General distribution.** Europe, Caucasus, Middle East, Central Asia. **Distribution in Bulgaria** (Fig. 74). Published records: BN, BS, DEP, PK, PKQ, PSL, PT, PVS, PVV, RRE, RRW (Harizanov 1963, Klimaszewski 1965, Harizanov 1966a, Głowacka and Harizanov 1983, Etropolska et al. 2015, Pramatarova et al. 2023). Material examined: BS, DEL, DW, PT, PVV, ROV, RPP, RRE.

##### Diagnosis

**Adult and fifth-instar immature.** Burckhardt and Hodkinson (1986), Burckhardt (2010).

#### Cacopsylla pyricola

(Foerster, 1848)

23AEAD2B-AA88-583E-957A-0C4B76A2732D

##### Feeds on

In Bulgaria, collected from *Pyruscommunis* L. (Etropolska et al. 2015), which is a confirmed host plant (Burckhardt and Hodkinson 1986, Cho et al. 2017b).

##### Distribution

**Figure 75.**
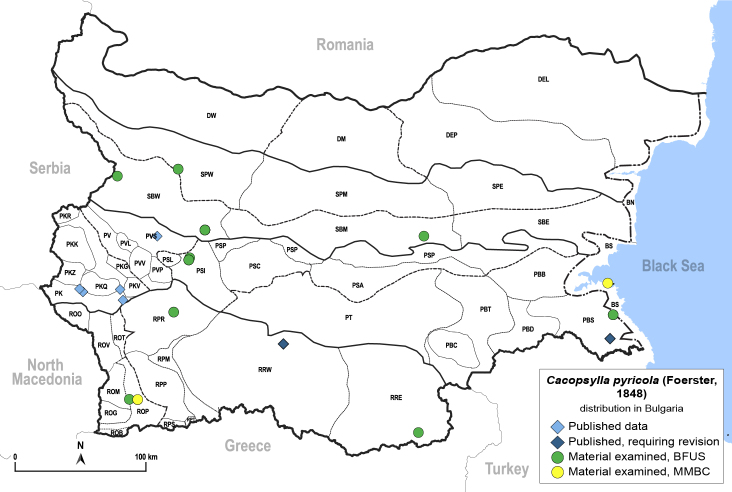
Distribution of *Cacopsyllapyricola* (Foerster, 1848) in Bulgaria.

**General distribution.** Europe, Turkey, Caucasus, alien in North America. **Distribution in Bulgaria** (Fig. 75). Published records: PBS, PK, PKQ, PVS, RRW (Klimaszewski 1965, Głowacka and Harizanov 1983, Etropolska et al. 2011, Etropolska et al. 2015). Material examined: BS, PBS, PSI, ROP, RPR, RRE, SBM, SBW, SPW.

##### Diagnosis

**Adult and fifth-instar immature.** Burckhardt and Hodkinson (1986), Burckhardt (2010).

#### Cacopsylla pyrisuga

(Foerster, 1848)

D2D7943B-FC4D-5460-8030-034E6DFF8913

##### Feeds on

In Bulgaria, adults and immatures were collected on *Pyruscommunis* L. (Harizanov 1963, Harizanov 1966e, Etropolska et al. 2015), which is a confirmed host plant (Burckhardt and Hodkinson 1986, Cho et al. 2017b).

##### Distribution

**Figure 76.**
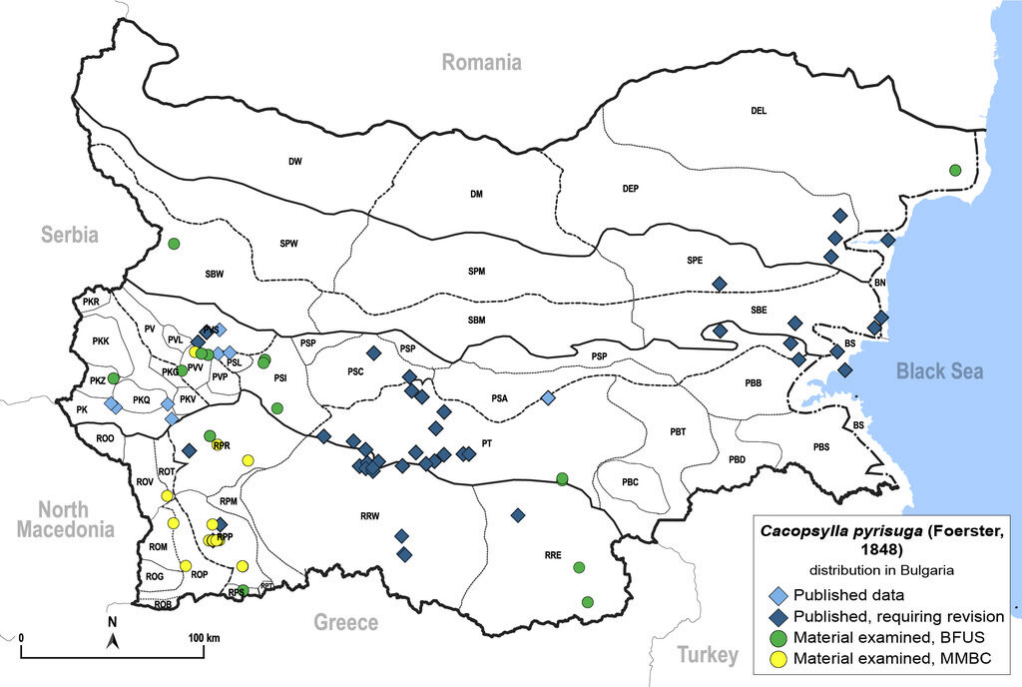
Distribution of *Cacopsyllapyrisuga* (Foerster, 1848) in Bulgaria.

**General distribution.** Europe, Caucasus, Middle East. **Distribution in Bulgaria** (*Fig. 76*). Published records: BN, BS, DEP, PBB, PK, PSC, PSL, PSP, PT, PVS, RPP, RRE, RRW, SBE (Joakimov 1909, Harizanov 1963, Harizanov 1964, Harizanov and Lauterer 1968, Klimaszewski 1965, Głowacka and Harizanov 1983, Etropolska et al. 2011, Etropolska et al. 2015, Pramatarova et al. 2023). Material examined: DEL, PKG, PKZ, PSI, PT, PVV, ROM, ROP, ROV, RPP, RPR, RPS, SBW.

##### Diagnosis

**Adult and fifth-instar immature.** Burckhardt and Hodkinson (1986), Burckhardt (2010).

#### Cacopsylla rhamnicola

(Scott, 1876)

A2751BC2-3653-5F76-B91A-AF524F96377F

##### Feeds on

In Bulgaria, we collected adults on *Rhamnuscathartica* L., which is a confirmed host plant (Ossiannilsson 1992).

##### Distribution

**General distribution.** Europe, Turkey, Caucasus, Central Asia, Mongolia. **Distribution in Bulgaria** (Fig. 77b). Material examined: PVV, RRW, SBW.

##### Notes

New record for Bulgaria.

##### Diagnosis

**Adult** (Fig. 77a) **and fifth-instar immature.** Ossiannilsson (1992).

#### Cacopsylla saliceti

(Foerster, 1848)

BBA6B434-DC6A-58B6-A5BB-EF6ABAAD8348

##### Feeds on

Collected on *Salix* spp. in Bulgaria (Głowacka and Harizanov 1983). Oligophagous on several *Salix* spp. (Lauterer and Burckhardt 1997, Percy and Cronk 2020).

##### Distribution

**Figure 78.**
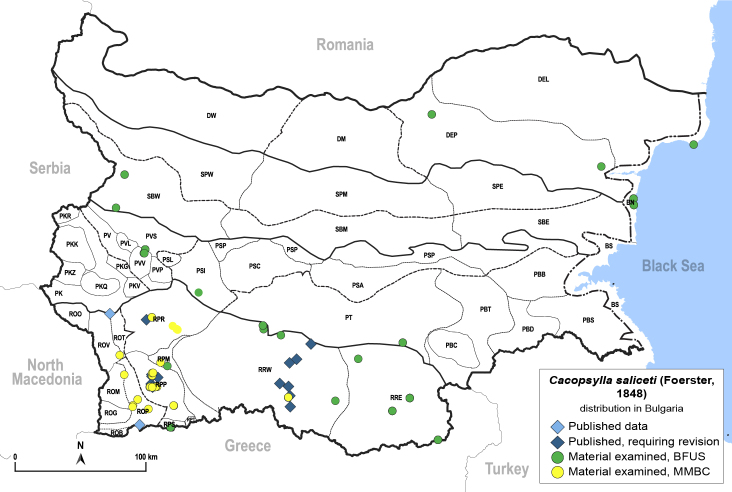
Distribution of *Cacopsyllasaliceti* (Foerster, 1848) in Bulgaria.

**General distribution.** Europe (except north), Caucasus, Middle East. **Distribution in Bulgaria** (Fig. 78). Published records: PK, ROP, RPP, RPR, RRW (Klimaszewski 1965, Harizanov and Lauterer 1968, Głowacka and Harizanov 1983, Głowacka 1989, Percy and Cronk 2020). Material examined: BN, DEP, PSI, PT, PVV, ROM, ROP, ROV, RPM, RPP, RPR, RPS, RRE, RRW, SBW.

##### Diagnosis

**Adult.** Loginova (1967), Lauterer and Burckhardt (1997).

#### Cacopsylla sorbi

(Linnaeus, 1767)

CF0CFA8E-89F9-5CD5-9EF3-76DC7ACD45D5

##### Feeds on

In Bulgaria, collected on *Sorbusaucuparia* L. (Głowacka and Harizanov 1983), which is a confirmed host plant (Ossiannilsson 1992).

##### Distribution

**Figure 79.**
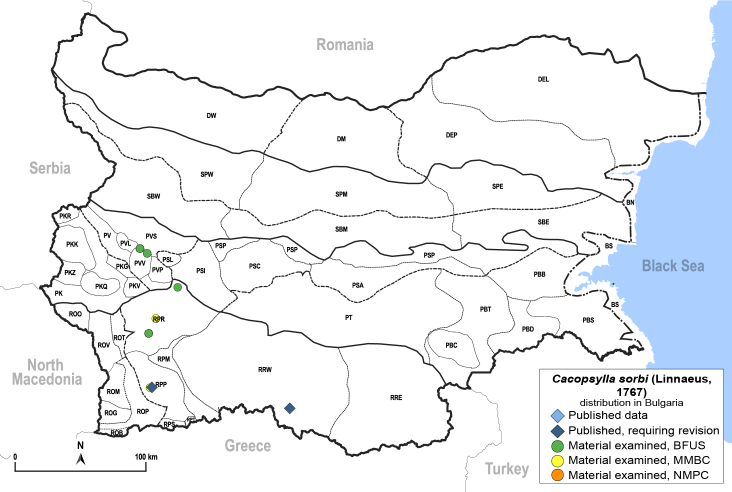
Distribution of *Cacopsyllasorbi* (Linnaeus, 1767) in Bulgaria.

**General distribution.** Europe, alien in North America. **Distribution in Bulgaria** (Fig. 79). Published records: RPP, RRW (Głowacka and Harizanov 1983, Głowacka 1989). Material examined: PVV, RPP, RPR.

##### Diagnosis

**Adult and fifth-instar immature.** Ossiannilsson (1992).

#### Cacopsylla ulmi

(Foerster, 1848)

BEF48AB7-659E-5411-9A95-00245B927826

##### Distribution

**Figure 80.**
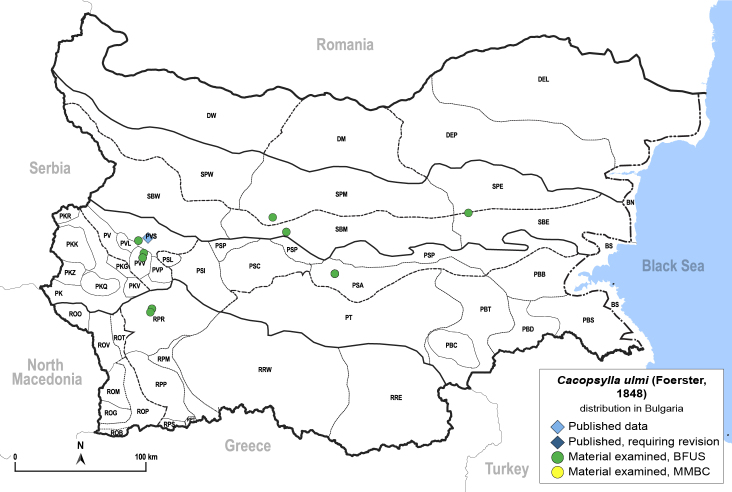
Distribution of *Cacopsyllaulmi* (Foerster, 1848) in Bulgaria.

**General distribution.** Europe, Caucasus, Central Asia, Russia (European part, Siberia and Far East) (Gegechkori and Loginova 1990). **Distribution in Bulgaria** (Fig. 80). Published records: PVS (Pramatarova et al. 2023). Material examined: PSA, PVS, PVV, RPR, SBM, SPE, SPM.

##### Diagnosis

**Adult and fifth-instar immature.** Ossiannilsson (1992).

#### Cacopsylla visci

(Curtis, 1835)

03902A35-A45C-5C27-8487-6943C6A2F311

##### Feeds on

We collected adults on *Viscumalbum* L., which is one of the confirmed host plants (Burckhardt et al. 2017).

##### Distribution

**General distribution.** Palaearctic. **Distribution in Bulgaria** (Fig. 81b). Published records: RPR (Lauterer 1979). Material examined: BS, PBS, RPR.

##### Diagnosis

**Adult** (Fig. 81a). Vondráček (1957a), Hodkinson and White (1979). **Fifth-instar immature.** White and Hodkinson (1982).

#### Livilla

Curtis, 1836

D37A1664-75D5-587B-B76C-BF39F1B36A3D

#### Livilla cognata

(Löw, 1881)

49E03CF6-8674-5B94-B906-2A822C5D7066

##### Feeds on

Oligophagous on *Chamaecytisus* spp. and probably also *Cytisus* and *Genista* spp. It was recorded from *Chamaecytisusratisbonensis* (Schaeff.) Rothm. in Austria (Löw 1888) and Czechia (Lauterer 1965; I. Malenovský, personnal observation), on *Genistagermanica* in Czechia (Lauterer 1965), on *Ch.hirsutus* (L.) Link and *Cytisusnigricans* L. in Italy (Conci et al. 1993) and on *Ch.heuffelii* (Wierzb.) Rothm. in Serbia (Jerinić-Prodanović 2010). In Bulgaria, we collected adults and immatures on *Chamaecytisuselongatus* (Waldst. & Kit.) Link., which is a newly-confirmed host species. We also repeatedly collected adults on Ch.austriacussubsp.stefanofii (Stoj.) Kuzmanov, which is another probable host.

##### Distribution

**General distribution.** Central and south-eastern Europe, Caucasus. **Distribution in Bulgaria** (Fig. 82b). Published records: RPP, RPR, RRW (Klimaszewski 1970, Głowacka and Harizanov 1983, Głowacka 1989). Material examined: ROB, ROO, RPP, RPR, RPS, RRE, SBW.

##### Diagnosis

**Adult** (Fig. 82a). Vondráček (1957a), Hodkinson and Hollis (1987).

#### Livilla horvathi

(Scott, 1879)

2EAB5A94-4C26-542D-82BF-BB4FFE7E8C75

##### Feeds on

*Livillahorvathi* was collected from *Chamaecytisusaustriacus* (L.) Link in Slovenia, Hungary and Slovakia (Löw 1888), *Genistatinctoria* L. in Romania (Dobreanu and Manolache 1962) and *G.sericea* Wulfen and *G.tinctoria* in Italy (Conci et al. 1993). In Bulgaria, we collected adults and one immature on *Genistalydia* Boiss. and *Chamaecytisuselongatus* (Waldst. & Kit.) Link, which are also probable host plants.

##### Distribution

**General distribution.** Central and south-eastern Europe, Turkey. **Distribution in Bulgaria** (Fig. 83c). Published records: PSA, RPM, RRW (Klimaszewski 1965, Głowacka and Harizanov 1983, Głowacka 1989, Pramatarova et al. 2021). Material examined: DEP, PBS, PBT, PSA, ROM, RPP, RPR, RPS, RRE, SBW.

##### Diagnosis

**Adult** (Fig. 83a). Vondráček (1957a), Hodkinson and Hollis (1987). **Fifth-instar immature** (Fig. 83b).

#### Livilla radiata

(Foerster, 1848)

3C4F211A-1714-546F-AAD2-A303C75AEBAD

##### Feeds on

Oligophagous on *Chamaecytisusaustriacus* (L.) Link., *Ch.borysthenicus* (Gruner) Klásk., *Ch.heuffelii* (Wierzb.) Rothm., *Ch.hirsutus* (L.) Link, *Ch.ratisbonensis* (Schaeff.) Rothm., *Cytisusnigricans* (L.) and *Cy.villosus* Pourr. (Löw 1888, Klimaszewski 1975, Andrianova and Klimaszewski 1983, Głowacka and Harizanov 1983, Conci et al. 1993, Malenovský et al. 2011). In south-western Bulgaria, we repeatedly collected adults on Ch.austriacussubsp.stefanoffii (Stoj.) Kuzmanov, syntopic with *Arytainamaculata* and *Livillacognata*.

##### Distribution

**General distribution.** Central and south-eastern Europe. **Distribution in Bulgaria** (Fig. 84b). Published records: RRW (Klimaszewski 1965, Głowacka and Harizanov 1983). Material examined: ROB, ROP, RPP, RPR, RPS, RRE, RRW, SPM.

##### Diagnosis

**Adult** (Fig. 84a). Vondráček (1957a), Hodkinson and Hollis (1987).

#### Livilla ulicis

Curtis, 1836

E5E2E78E-3A33-5F32-B5D6-B4598BE972E8

##### Distribution

**General distribution.** Europe (except north). **Distribution in Bulgaria** (Fig. 85b). Published records: PVV, RPR (Joakimov 1909, Klimaszewski 1965). Material examined: PVV, RPP, RPR, RRE, RRW.

##### Diagnosis

**Adult** (Fig. 85a). Vondráček (1957a), Hodkinson and Hollis (1987). **Fifth-instar immature.** Loginova (1976), White and Hodkinson (1982).

#### Livilla variegata

(Löw, 1881)

74D800B6-3D50-5B44-BF93-1C203287F8D5

##### Feeds on

Both known host plants of *L.variegata*, *Laburnumanagyroides* Medik. and *L.alpinum* (Mill.) Bercht. & J.Presl, are not native to Bulgaria. Our specimens (adults and immatures) were collected on *L.anagyroides* in ornamental greenery in the Sofia agglomeration. *Laburnumanagyroides* is frequently planted as an ornamental tree in Bulgaria and spreads in both natural and disturbed habitats (Petrova et al. 2013).

##### Distribution

**General distribution.** Native to southern and some parts of central Europe (e.g. the Alps), alien in western and other parts of central Europe and North America. **Distribution in Bulgaria** (Fig. 86b). Material examined: PVS.

##### Notes

New record for Bulgaria.

##### Diagnosis

**Adult** (Fig. 86a) Hodkinson and Hollis (1987). **Fifth-instar immature.** White and Hodkinson (1982).

#### Psylla

Geoffroy, 1762

BBE5A1BF-03B6-513A-B526-61B6FAE95B2C

#### Psylla alni

(Linnaeus, 1758)

BFAB7379-92E6-577D-A53E-5698C1CA1B5D

##### Feeds on

Collected on *Alnusglutinosa* (L.) Gaertn. (Głowacka and Harizanov 1983), which is one of the confirmed host plants (Ossiannilsson 1992).

##### Distribution

**General distribution.** Holarctic. **Distribution in Bulgaria** (Fig. 87b). Published records: ROP, RPP, RRW (Klimaszewski 1965, Głowacka and Harizanov 1983, Głowacka 1989, Pramatarova et al. 2023). Material examined: BN, PKZ, PVS, ROM, ROP, RPM, RPP, RPR.

##### Diagnosis

**Adult** (Fig. 87a) **and fifth-instar immature.** Ossiannilsson (1992).

#### Psylla alpina

Foerster, 1848

9EC89FB8-EF60-514D-9BE4-452BBFACCB6D

##### Feeds on

We collected adults and immatures on *Alnusalnobetula* (Ehrh.) K.Koch. (= *A.viridis* (Chaix) DC.), which is the only confirmed host plant (Lauterer 1979, Seljak 2020).

##### Distribution

**General distribution.** Mountains in central and south-eastern Europe. **Distribution in Bulgaria** (Fig. 88b). Published records: RPR (Lauterer 1979). Material examined: RPR.

##### Diagnosis

**Adult** (Fig. 88a). Vondráček (1957a).

#### Psylla colorata

Löw, 1888

6157781A-22C7-51A6-9C88-7D6331E634AD

##### Feeds on

Adults were collected by *Głowacka and Harizanov (1983)* and the authors on *Ostryacarpinifolia* Scop., which is the only confirmed host plant (Burckhardt 1979, Seljak 2020).

##### Distribution

**Figure 89.**
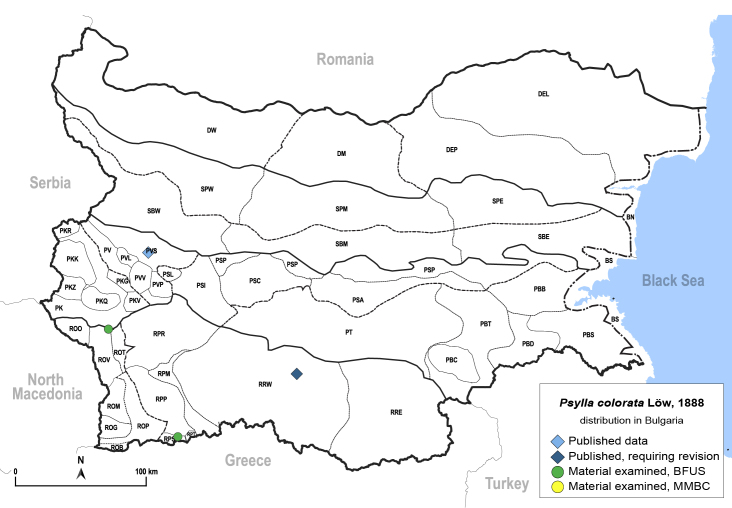
Distribution of *Psyllacolorata* Löw, 1888 in Bulgaria.

**General distribution.** Southern parts of western, central and eastern Europe, Middle East. **Distribution in Bulgaria** (Fig. 89). Published records: PVS, RRW (Głowacka and Harizanov 1983, Labina 2007, Labina et al. 2014). Material examined: ROV, RPS.

##### Diagnosis

**Adult and fifth instar immature.** Burckhardt (1979).

#### Psylla foersteri

Flor, 1861

3D8ABD55-F19F-594B-9751-F51532A414D0

##### Feeds on

*Alnus* spp. (Głowacka 1989); we collected adults on *Alnusglutinosa* (L.) Gaertn., which is one of the confirmed host plants (Ossiannilsson 1992).

##### Distribution

**General distribution.** Europe, North Africa, Caucasus, Middle East, alien in New Zealand. **Distribution in Bulgaria** (Fig. 90b). Published records: PSA, PSP, PVL, PVS, ROP, RRW (Joakimov 1909, Klimaszewski 1965, Głowacka and Harizanov 1983, Głowacka 1989, Pramatarova et al. 2021, Pramatarova et al. 2023). Material examined: BN, PKG, PKZ, PSA, PSP, PVV, ROM, ROP, RPM, RPR.

##### Diagnosis

**Adult** (Fig. 90a) **and fifth-instar immature.** Ossiannilsson (1992).

#### Psylla fusca

(Zetterstedt, 1828)

DDBAB863-1686-587F-AE5B-C4D5D643F364

##### Distribution

**Figure 91.**
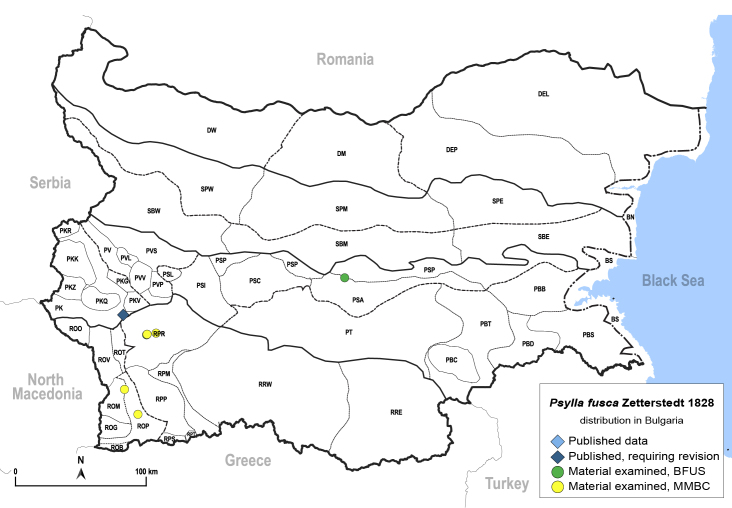
Distribution of *Psyllafusca* Zetterstedt 1828 in Bulgaria.

**General distribution.** Europe. **Distribution in Bulgaria** (Fig. 91). Published records: PK (Schaefer 1949). Material examined: PSP, ROM, ROP, RPR.

##### Diagnosis

**Adult and fifth-instar immature.** Ossiannilsson (1992).

#### Psylla hartigii

Flor, 1861

C1BDDDD3-E09B-5FC2-93F0-51C2CBFCEC63

##### Feeds on

In Bulgaria, collected on *Betula* sp. (Głowacka and Harizanov 1983). Oligophagous on *Betula* spp. (Ossiannilsson 1992).

##### Distribution

**Figure 92.**
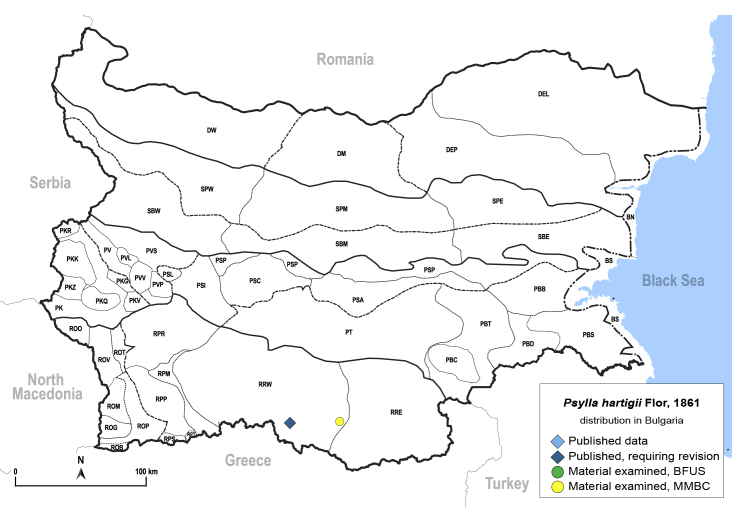
Distribution of *Psyllahartigii* Flor, 1861 in Bulgaria.

**General distribution.** Holarctic. **Distribution in Bulgaria** (Fig. 92). Published records: RRW (Głowacka and Harizanov 1983). Material examined: RRW.

##### Diagnosis

**Adult and fifth-instar immature.** Ossiannilsson (1992).

#### Spanioneura

Foerster, 1848

8C9BEAEE-43F2-5799-9BCA-4400EC3CD01C

#### Spanioneura buxi

(Linnaeus, 1758)

7622637C-0F78-52DF-AE99-430CAF2CEE0B

##### Feeds on

Oligophagous on *Buxus* spp. (Hodkinson and White 1979). In Bulgaria, Głowacka and Harizanov (1983), Głowacka (1989) and the authors collected adults on *B.sempervirens* L., which is one of the confirmed host species. *Buxussempervirens* is not native to Bulgaria (POWO 2024), but is widely cultivated as an ornamental plant.

##### Distribution

**General distribution.** Western and southern Europe, Caucasus, alien in Central, northern and eastern Europe, North America and Hawaii. **Distribution in Bulgaria** (Fig. 93b). Published records: ROP, RPP, RRW (Głowacka and Harizanov 1983, Głowacka 1989). Material examined: ROM, PVS.

**Notes**: *Spanioneurabuxi* is an alien species in Bulgaria.

##### Diagnosis

**Adult** (Fig. 93a) **and fifth-instar immature.** Ossiannilsson (1992).

#### Spanioneura fonscolombii

Foerster, 1848

E239B35D-847C-5CB1-991B-E61B3039BF7C

##### Feeds on

We collected adults on *Buxussempervirens* L., which is the only confirmed host plant (Hodkinson and White 1979, Burckhardt and Mühlethaler 2003).

##### Distribution

**Figure 94.**
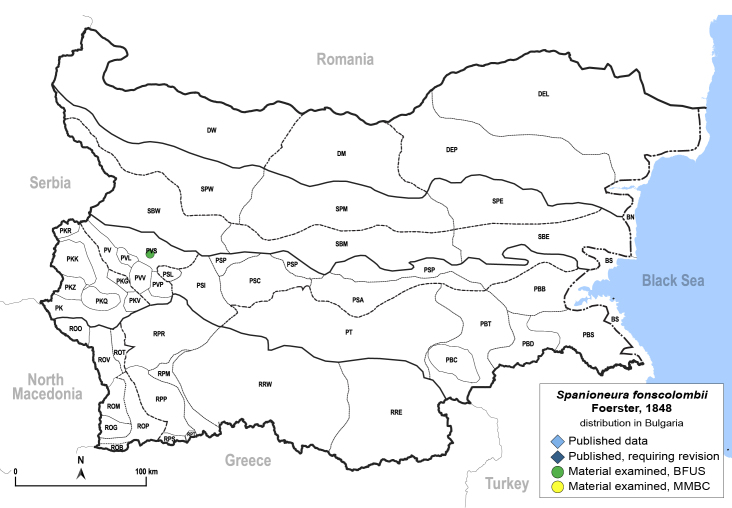
Distribution of *Spanioneurafonscolombii* Foerster, 1848 in Bulgaria.

**General distribution.** Western and southern Europe, Caucasus (Gegechkori and Loginova 1990), alien in central, northern and eastern Europe, North America and Australia (Oswald 2022, Prunar et al. 2023). **Distribution in Bulgaria** (Fig. 94). Material examined: PVS.

##### Notes

New record for Bulgaria. *Spanioneurafonscolombii* has spread in Europe on cultivated box trees in the last two decades. Our record from Bulgaria (from ornamental greenery in a city park) is further evidence of its expansion after the recent records in Romania, Austria and other countries (Oswald 2022, Prunar et al. 2023).

##### Diagnosis

**Adult.** Hodkinson and White (1979)**. Fifth-instar immature.** White and Hodkinson (1982).

#### Triozidae

Löw, 1879

78240868-B941-53FD-A4E7-C1A0F6E3F0EA

#### Bactericera

Puton, 1876

7DB25062-5482-589F-80E3-B2832938FEEB

#### Bactericera albiventris

(Foerster, 1848)

3BB7D45B-1456-5D7B-91BA-D4B5D6988255

##### Feeds on

Oligophagous on several *Salix* spp. (Głowacka 1989, Ossiannilsson 1992, Burckhardt and Lauterer 1997b, Percy and Cronk 2020). We collected adults on *Salixalba* L., which is one of the confirmed host plants.

##### Distribution

**General distribution.** Palaearctic. **Distribution in Bulgaria** (Fig. 95b). Published records: PK, PVS, PVV, ROP, RPM, RPP, RRW, SBW (Joakimov 1909, Głowacka and Harizanov 1983, Głowacka 1989, Percy and Cronk 2020, Pramatarova et al. 2023). Material examined: BN, PKG, PKZ, PSI, PSP, PT, PVV, ROM, ROP, RPM, RPR, RRE, RRW, SBW.

##### Diagnosis

**Adult** (Fig. 95a) **and fifth-instar immature.** Ossiannilsson (1992), Burckhardt and Lauterer (1997b).

#### Bactericera bohemica

(Šulc, 1913)

34DE9532-598E-58D3-B2D5-E177C8BDD712

##### Feeds on

*Geummontanum* L. (Głowacka 1989), which is one of the confirmed host plants (Ossiannilsson 1992).

##### Distribution

**Figure 96.**
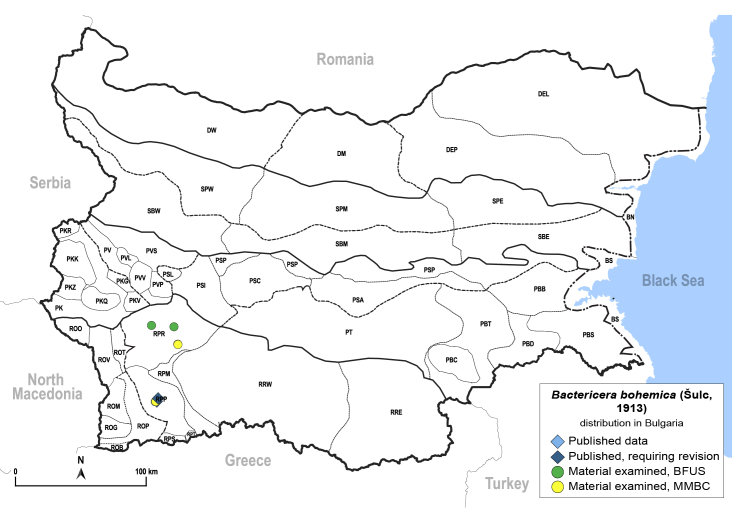
Distribution of *Bactericerabohemica* (Šulc, 1913) in Bulgaria.

**General distribution.** Northern Europe, mountains in central and south-eastern Europe, Caucasus, southern Siberia and Mongolia (Gegechkori and Loginova 1990). **Distribution in Bulgaria** (Fig. 96). Published records: RPP (Głowacka 1989), without locality data (Burckhardt and Lauterer 1997b). Material examined: RPP, RPR.

##### Diagnosis

**Adult and fifth-instar immature.** Conci and Tamanini (1985c), Ossiannilsson (1992).

#### Bactericera curvatinervis

(Foerster, 1848)

CC2926AE-AC77-56DB-8F54-543F8B667691

##### Feeds on

Reported from *Salix* spp. in Bulgaria (Głowacka and Harizanov 1983, Głowacka 1989). Oligophagous on several *Salix* spp. (Ossiannilsson 1992, Burckhardt and Lauterer 1997b, Percy and Cronk 2020).

##### Distribution

**Figure 97.**
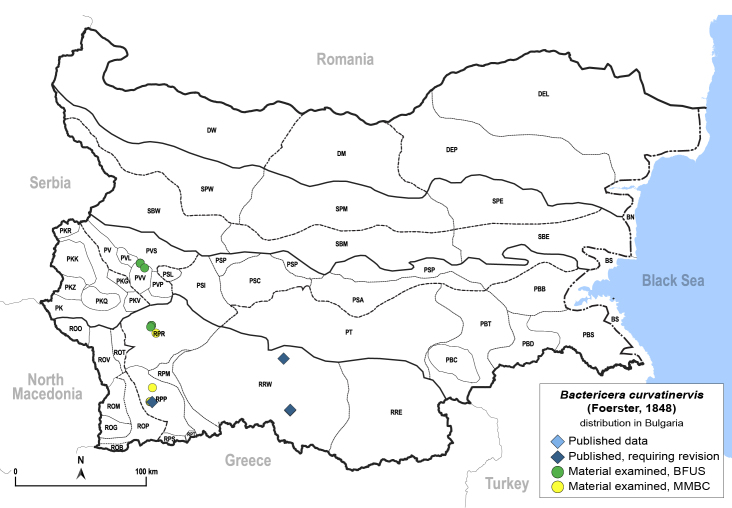
Distribution of *Bactericeracurvatinervis* (Foerster, 1848) in Bulgaria.

**General distribution.** Palaearctic. **Distribution in Bulgaria** (Fig. 97). Published records: RPP, RRW (Głowacka and Harizanov 1983, Głowacka 1989). Material examined: PVV, RPP, RPR.

##### Diagnosis

**Adult and fifth-instar immature.** Ossiannilsson (1992), Burckhardt and Lauterer (1997b).

#### Bactericera femoralis

(Foerster, 1848)

651B883A-E8CD-5879-AB76-C6E39B3FEC45

##### Feeds on

Oligophagous on *Alchemilla* spp. (Ossiannilsson 1992, Burckhardt and Lauterer 1997b), on which we also collected adults in Bulgaria.

##### Distribution

**General distribution.** Palaearctic. **Distribution in Bulgaria** (Fig. 98b). Published records: PVV (Klimaszewski 1965). Material examined: RPP, RPR, SBM.

##### Diagnosis

**Adult** (Fig. 98a) **and fifth-instar immature.** Ossiannilsson (1992).

#### Bactericera harrisoni

(Wagner, 1955)

46FF61A6-D547-5359-A77C-11CFE1ACBA66

##### Distribution

**Figure 99.**
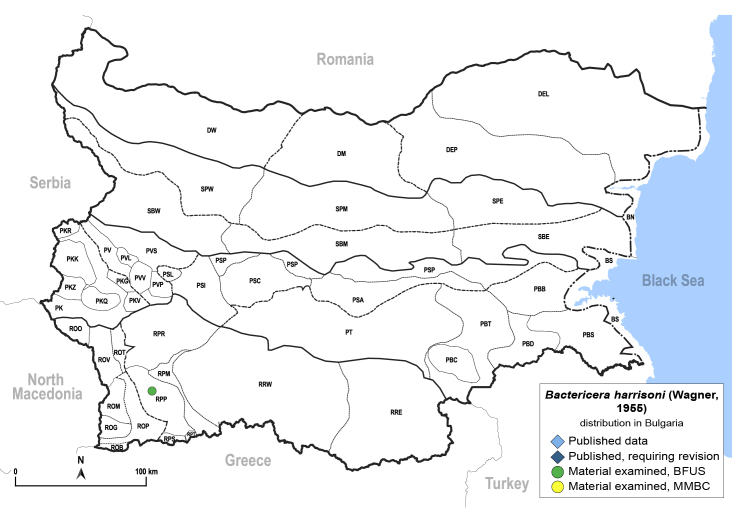
Distribution of *Bactericeraharrisoni* (Wagner, 1955) in Bulgaria.

**General distribution.** Mountains in central and south-eastern Europe (Austria, Czechia, Italy, Romania, Slovakia, Slovenia, Switzerland). **Distribution in Bulgaria** (Fig. 99). Material examined: RPP.

##### Notes

New record for Bulgaria. The record of the similar and closely-related *B.reuteri* (Šulc, 1913) from the Western Rhodopes (RRW) published by Głowacka and Harizanov (1983) could indeed concern *B.harrisoni* (see the comment under Doubtful records below).

##### Diagnosis

**Adult.** Conci and Tamanini (1985c).

#### Bactericera lyrata

Seljak, Malenovský & Lauterer, 2008

E56DD5EE-1D2A-5A42-A268-195C7D676E85

##### Distribution

**General distribution.** Central and south-eastern Europe (Czechia, Hungary, Slovenia). **Distribution in Bulgaria** (Fig. 100b). Material examined: PSI, ROP, SBW.

##### Notes

New record for Bulgaria. The record of the similar and closely related *B.reuteri* (Šulc, 1913) from the Western Rhodopes (RRW) published by Głowacka and Harizanov (1983) could indeed concern *B.lyrata* or *B.harrisoni* (see the comment under 'Doubtful records' below).

##### Diagnosis

**Adult** (Fig. 100a). Seljak et al. (2008). **Fifth-instar immature.** Seljak and Malenovský (2014).

#### Bactericera modesta

(Foerster, 1848)

BDA309AF-20C3-58B1-A8A9-733BAC1B0C42

##### Feeds on

In Bulgaria, we collected adults on *Sanguisorbaminor* Scop., which is one of the confirmed host plants (Burckhardt and Lauterer 1997b).

##### Distribution

**General distribution.** South-western, central and south-eastern Europe, Central Asia and Mongolia. **Distribution in Bulgaria** (Fig. 101b). Published records: Bulgaria, without locality data (Burckhardt and Lauterer 1997b), PSA (Pramatarova et al. 2021), RRW (Głowacka and Harizanov 1983). Material examined: BS, PBS, PSA, PT, ROP, RPS, RRE.

##### Diagnosis

**Adult** (Fig. 101a) Burckhardt and Lauterer (1997b), Seljak et al. (2008)**. Fifth-instar immature.** Burckhardt and Lauterer (1997b).

#### Bactericera nigricornis

(Foerster, 1848)

AD4A41F8-1324-5AC4-8FEF-599AF5C04583

##### Feeds on

Polyphagous (Ossiannilsson 1992). In Bulgaria, reported from *Alliumcepa* L. (Konov 1961), *Brassicaoleracea* L., *Daucuscarota* L. (Harizanov and Lauterer 1968) and *Solanumtuberosum* L. (Harizanov 1970).

##### Distribution

**General distribution.** Palaearctic. **Distribution in Bulgaria** (Fig. 102b). Published records: DEP, DW, PT, RPP, RRW (Konov 1961, Harizanov and Lauterer 1968, Głowacka and Harizanov 1983, Głowacka 1989). Material examined: ROP, ROV, RPP, RPR.

##### Notes

The report by Konov (1961) on *B.nigricornis* from *Alliumcepa* could concern the similar *Bactericeratremblayi* (Wagner, 1961), which is known from onion and leek crops in neighbouring countries (Serbia, Greece and Turkey: Jerinić-Prodanović (2010), Ouvrard and Burckhardt (2012)), but has not been documented from Bulgaria.

##### Diagnosis

**Adult** (Fig. 102a) **and fifth-instar immature.** Ossiannilsson (1992), Burckhardt and Freuler (2000).

#### Bactericera perrisii

Puton, 1876

79FFF290-B798-5547-85A1-FA0A8B99BCD7

##### Feeds on

In Bulgaria, we collected adults on *Artemisiacampestris* L., which is a confirmed host plant (Lauterer 1982, Burckhardt and Lauterer 1997b).

##### Distribution

**General distribution.** Southern, central and south-eastern Europe, Caucasus, Middle East, Central Asia, southern Siberia, Mongolia. **Distribution in Bulgaria** (Fig. 103b). Published records: ROP (Głowacka 1989). Material examined: BN, ROM, ROP, ROV.

##### Diagnosis

**Adult** (Fig. 103a) **and fifth-instar immature.** Burckhardt and Lauterer (1997b).

#### Bactericera striola

(Flor, 1861)

71C04C85-8F90-5273-AF12-DE88D60A4ABB

##### Feeds on

In Bulgaria, collected on *Salix* sp. (Głowacka and Harizanov 1983). Oligophagous on several *Salix* spp. (Ossiannilsson 1992, Burckhardt and Lauterer 1997b, Percy and Cronk 2020).

##### Distribution

**General distribution.** Europe, Caucasus, Central Asia (Gegechkori and Loginova 1990), southern Siberia, Mongolia. **Distribution in Bulgaria** (Fig. 104b). Published records: PVS, RRW (Joakimov 1909, Głowacka and Harizanov 1983). Material examined: RPP, RPR.

##### Diagnosis

**Adult** (Fig. 104a) **and fifth-instar immature.** Ossiannilsson (1992).

#### Bactericera trigonica

Hodkinson, 1981

C89A5C8C-2790-59E2-85CE-BE5F12A91E84

##### Distribution

**General distribution.** Southern and Central Europe, North Africa, Middle East. **Distribution in Bulgaria** (Fig. 105b). Material examined: BN, ROP

##### Notes

New record for Bulgaria.

##### Diagnosis

**Adult** (Fig. 105a) **and fifth-instar immature.** Burckhardt and Freuler (2000).

#### Dyspersa

Klimaszewski, 1968

E2E7EC9D-837E-5360-8BC3-F214FFADD03E

#### Dyspersa abdominalis

(Flor, 1861)

03ADE25B-6A03-5356-A7B4-B91348A654A8

##### Distribution

**Figure 106.**
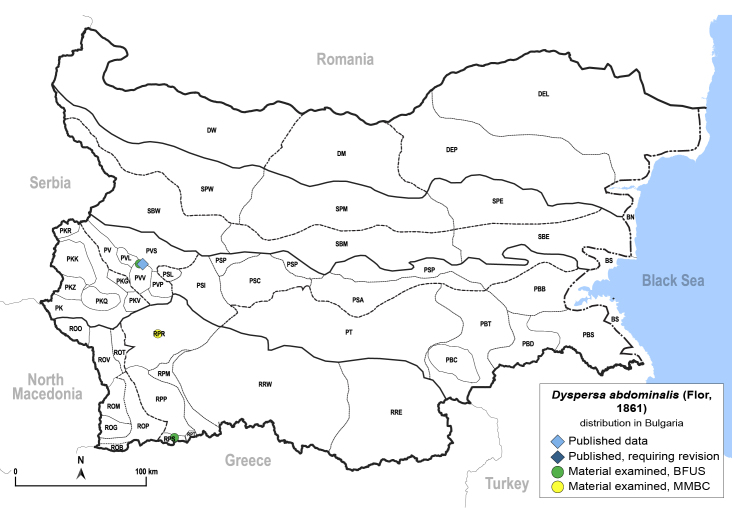
Distribution of *Dyspersaabdominalis* (Flor, 1861) in Bulgaria.

**General distribution.** Palaearctic. **Distribution in Bulgaria** (Fig. 106). Published records: PVV (Pramatarova et al. 2023). Material examined: PVV, RPR, RPS.

##### Diagnosis

**Adult and fifth-instar immature.** Ossiannilsson (1992).

#### Dyspersa cirsii

(Löw, 1881)

BD8F4A64-3EEF-5A20-A9EC-56037BD93B91

##### Feeds on

Oligophagous on *Cirsium* spp. (Ossiannilsson 1992). In Bulgaria, we collected adults on *Cirsiumappendiculatum* Griseb., which is a probable host plant.

##### Distribution

**General distribution.** Europe. **Distribution in Bulgaria** (Fig. 107b). Published records: RPP, RRW (Harizanov and Lauterer 1968, Klimaszewski 1970, Głowacka and Harizanov 1983: all as *Triozaviridula* auct. nec Zetterstedt, 1828; Głowacka (1989)). Material examined: PKQ, ROV, RPP, RPR.

##### Diagnosis

**Adult** (Fig. 107a) **and fifth-instar immature.** Ossiannilsson (1992).

#### Dyspersa kantshavelii

(Gegechkori, 1977)

2CFD2085-D0BA-5CCA-A4FA-D29189FC4EAE

##### Feeds on

The host plant of *D.kantshavelii* is unknown. Based on its morphology, it belongs to a complex of species that are associated with thistles (*Cirsium* spp.) (Ossiannilsson 1972, Burckhardt and Lauterer 2002b).

##### Distribution

**General distribution.** Caucasus (Armenia, Azerbaijan, Georgia), Turkey. **Distribution in Bulgaria** (Fig. 108f). Material examined: ROP, RPP, RRW.

##### Notes

New record for Bulgaria and Europe. *Dyspersakantshavelii* is a little-known species that was previously only known from the mountains of the Caucasus and north-eastern Turkey (Gegechkori 1977, Gegechkori 1984, Burckhardt 1988). In Bulgaria, the species was collected by P. Lauterer in several places in the Pirin mountains , mostly at high altitudes (1600–2500 m a.s.l.); there is also a record from the Western Rhodopi.

##### Diagnosis

**Adult** (Fig. 108a, b, c, d, e). We provide here photos and drawings of the diagnostic characters, based on specimens collected in Bulgaria. The specimens correspond well with the original description (Gegechkori 1977). *Dyspersakantshavelii* resembles *D.agrophila* (Löw, 1888), *D.cirsii*, *D.flixiana* (Burckhardt & Lauterer, 2002) and *D.viridula* (Zetterstedt, 1828) in the size, colouration, genal processes, forewing venation and general structure of the male and female terminalia, but differs from them in the shape of the paramere (its posterior margin is almost evenly convex and the anterior margin is more shallowly incised subapically in lateral view and the apical process is shorter than in all other species), the distal segment of the aedeagus (its apical dilation is subglobular and bearing a small tooth and incision anteriorly/ventrally in lateral view, whereas this tooth and incision is larger in the other species) and the female terminalia (the apical processes of the proctiger and the subgenital plate is similar to *D.viridula*, but slightly shorter than in *D.agrophila* and longer than in *D.cirsii* and *D.flixiana*) (Fig. 108e, see also Ossiannilsson (1992), Burckhardt and Lauterer (2002b)).

#### Dyspersa mesembrina

(Burckhardt, 1986)

C7F53558-B812-5599-BC69-3B41B1912AAB

##### Distribution

**Figure 109.**
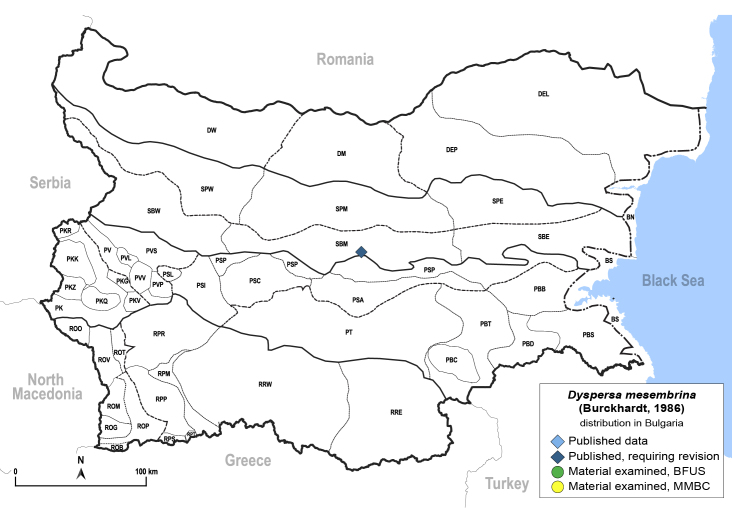
Distribution of *Dyspersamesembrina* (Burckhardt, 1986) in Bulgaria.

**General distribution.** Mountains in Central and south-eastern Europe (Switzerland, Italy, Slovakia and Bulgaria). **Distribution in Bulgaria** (Fig. 109). Published records: SBM (Klimaszewski (1970), as *Triozapallida*; Burckhardt 1986).

##### Notes

According to Burckhardt (1986), the specimens recorded by Klimaszewski (1970) from Bulgaria as "*Triozapallida*" are *T.mesembrina*.

##### Diagnosis

**Adult and fifth-instar immature.** Burckhardt (1986).

#### Dyspersa munda

(Foerster, 1848)

5E5D5637-AA76-5420-AB7A-881D8559FF7B

##### Distribution

**Figure 110.**
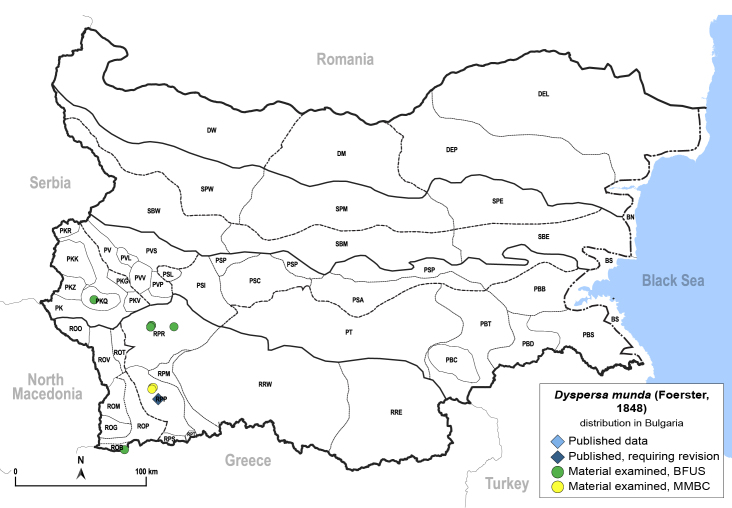
Distribution of *Dyspersamunda* (Foerster, 1848) in Bulgaria.

**General distribution.** Palaearctic. **Distribution in Bulgaria** (Fig. 110). Published records: RPP (Głowacka 1989). Material examined: PKQ, ROB, RPP, RPR.

##### Diagnosis

**Adult and fifth-instar immature.** Ossiannilsson (1992).

#### Dyspersa pallida

(Haupt, 1935)

631194D2-1302-5C14-81C8-9716E3C7DA2A

##### Distribution

**Figure 111.**
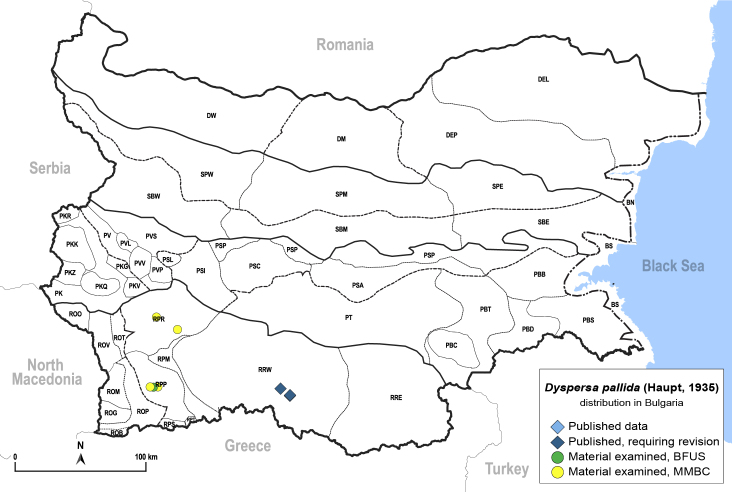
Distribution of *Dyspersapallida* (Haupt, 1935) in Bulgaria.

**General distribution.** Europe, Turkey, Russia (European part, Siberia). **Distribution in Bulgaria** (Fig. 111). Published records: RRW (Głowacka and Harizanov 1983). Material examined: RPP, RPR.

##### Diagnosis

**Adult and fifth-instar immature.** Burckhardt (1986), Ossiannilsson (1992), Burckhardt and Freuler (2000), as *Triozaanthrisci* Burckhardt, 1986.

#### Eryngiofaga

Klimaszewski, 1968

B9A2A411-02FA-5118-8A41-B60B891F398A

#### Eryngiofaga babugani

(Loginova, 1964)

906B9E92-4077-54A0-82C7-BFAC89EB3CC7

##### Distribution

**General distribution.** South-eastern Europe (Ukraine: Crimea), Caucasus (Russia: Dagestan) (Loginova 1977). The species identity of specimens from China (Li 2011) is doubtful. **Distribution in Bulgaria** (Fig. 112b). Material examined: BN.

##### Notes

New record for Bulgaria and the Balkan Peninsula. Only a single female was collected by P. Lauterer on 23 July 1987 in a dry grassland vegetation on limestone on the Black Sea coast (10–50 m a.s.l.). This specimen has an ochreous vertex and thorax, extensive dark brown pattern on thorax and almost completely dark brown to black abdomen, legs and antennae (except the antennal segment 3 which is dark yellow), a small body size (total length including forewings folded over the body 2.3 mm), short antennae (0.83 mm), a high ratio of the length of the antennal segments 3 and 4 (4.3), relatively long genal processes (almost as long as the vertex in frontal view), forewing with a quite short and convex vein Rs, clear or only slightly whitish membrane and a small dark spot and a darkened anal vein in the middle of the clavus and a slightly concave female proctiger posterior to the circumanal pore ring. It resembles *E.babugani* in these characters, but the measurements do not match the description by Loginova (1977) perfectly and more specimens, especially males, are needed to confirm the identification.

##### Diagnosis

**Adult** (Fig. 112a). Loginova (1977).

#### Eryngiofaga dlabolai

(Vondráček 1957)

4321B4B3-44C3-55D9-A5E2-033DFA7D6463

##### Feeds on

We collected adults and immatures on *Eryngiumcampestre* L., which is a confirmed host plant (Loginova 1977).

##### Distribution

**General distribution.** Central and south-eastern Europe, Caucasus. **Distribution in Bulgaria** (Fig. 113b). Published records: PSA, PSP (Joakimov (1909), as "*Triozamesotela*"; Pramatarova et al. 2021, Pramatarova et al. 2023). Material examined: BN, BS, DEP, DM, PBC, PBT, PKZ, PSA, PVS, ROM, ROV, RPS, RRE, SBW, SPW.

##### Notes

The record of *Eryngiofagamesomela* (Flor, 1861) (cited as '*Triozamesotela* Flor') from Bulgaria by Joakimov (1909) is here attributed to *E.dlabolai*. On the basis of numerous recently collected material from different regions, *E.dlabolai* seems to be the only *Eryngiofagas*pecies associated with *Eryngium* in Bulgaria.

##### Diagnosis

**Adult** (Fig. 113a). Vondráček (1957b), Loginova (1977).

#### Heterotrioza

Dobreanu & Manolache, 1960

A40E6D47-59DF-58A3-B5BA-022A3D50F1A9

#### Heterotrioza chenopodii

(Reuter, 1876)

6E2AE50F-2E22-5DC8-BEB4-8C373C5344F8

##### Feeds on

Oligophagous on Amaranthaceae (Lauterer 1982, Ossiannilsson 1992). In Bulgaria, adults were collected on *Atriplex* spp. by Głowacka and Harizanov (1983) and *Głowacka (1989)*, on *Chenopodium* sp. by Harizanov and Lauterer (1968) and on *Chenopodiumalbum* L. by the authors.

##### Distribution

**General distribution.** Palaearctic, India, alien in North and South America and Hawaii. **Distribution in Bulgaria** (Fig. 114c). Published records: BS, PT, PVV, ROP, RPP, RRW (Klimaszewski (1965), as *Triozaobliquahorvathi*; Harizanov and Lauterer (1968), as *Triozachenopodii*; Głowacka and Harizanov (1983), as *Heterotriozaobliquahorvathi*; Głowacka (1989), Pramatarova et al. (2023)). Material examined: BS, ROM, ROV, ROB, ROP.

##### Diagnosis

**Adult** (Fig. 114a, b) **and fifth-instar immature.** Ossiannilsson (1992).

#### Heterotrioza dichroa

(Scott, 1879)

4891D26B-A6A8-5E19-8BEE-BE86F592DC0B

##### Distribution

**Figure 115.**
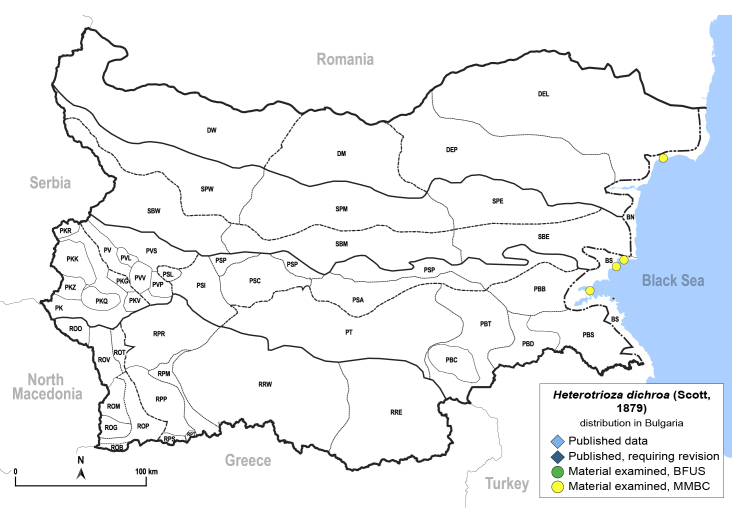
Distribution of *Heterotriozadichroa* (Scott, 1879) in Bulgaria.

**General distribution.** Central and south-eastern Europe, Caucasus, Middle East, Central Asia. **Distribution in Bulgaria** (Fig. 115). Published records: Bulgaria, without locality data (Lauterer 1991). Material examined: BN, BS.

##### Notes

Lauterer (1991) mentioned only briefly that he collected *H.dichroa* in Bulgaria on *Atripextatarica*. Here, we give more details on his material and confirm the presence of *H.dichroa* in Bulgaria.

##### Diagnosis

**Adult.** Loginova (1964), partly as *Triozaatriplicina*; Lauterer (1965).

**Feeds on**: In Bulgaria, adults were collected on *Atriplextatarica* L., which is a confirmed host plant (Lauterer 1991).

#### Heterotrioza kochiae

(Gegechkori, 1975)

B5FB351F-19AF-50DA-81ED-7717A50A5CDC

##### Feeds on

In Bulgaria, adults were collected on *Bassiaprostrata* (L.) Beck (= *Kochiaprostrata* (L.) Schrad., Amaranthaceae), which has been listed as a host plant (Gegechkori 1984, Gegechkori and Loginova 1990).

##### Distribution

**General distribution.** Bulgaria, Caucasus (Azerbaijan, Armenia, Georgia, Russia: Stavropol Region) (Gegechkori and Loginova 1990). **Distribution in Bulgaria** (Fig. 116b). Material examined: BN.

##### Notes

New record for Bulgaria and Europe.

##### Diagnosis

**Adult** (Fig. 116a, c, d, e). Originally described (as *Triozakochiae*) by Gegechkori (1975) from Georgia and later also recorded from other countries of the Caucasus. Gegechkori (1975) considered *H.kochiae* to be similar and closely related to *Triozaeurotiae* Loginova, 1960 (now *Heterotriozaeurotiae*), which occurs sympatrically with *H.kochiae* in the Caucasus, but has also been recorded from Kazakhstan, Central Asia, Mongolia and Iran (Loginova 1960, Gegechkori and Loginova 1990, Burckhardt and Lauterer 1993). However, Gegechkori (1975) did not give any characters to distinguish the two species and his illustrations are not detailed enough, which makes the identification of *H.kochiae* difficult. Here, we provide habitus photographs and illustrations of the male and female terminalia of specimens of *H.kochiae* from Bulgaria, collected from *Bassiaprostrata* on the Black Sea coast in summer (15 July 1973, P. Lauterer leg.) (Fig. 116a, c, d, e), which agree well with the original description, except for a smaller size (forewing length, in mm: 1.38–1.44 in males (n = 3) and 1.46–1.56 in females (n = 3) from Bulgaria, while Gegechkori (1975) reported 1.59–1.67 in males and 1.62–1.70 in females for the type series from Georgia, collected on 1 June). We consider this size difference as intraspecific variation. We compared the Bulgarian specimens of *H.kochiae* with the original description of *H.eurotiae* and with material from Kazakhstan, identified as *H.eurotiae* by Loginova and deposited in the MMBC (1 male, 2 females, Kokterek sands, 10 km Karatal, Zaisan District, 9 July 1962, *Eurotiaceratoides*, leg. Loginova). On the basis of this comparison, we can confirm that *H.kochiae* and *H.eurotiae* are similar in body colouration and the general structure of the head, forewings, female and male terminalia and especially the aedeagus. *Heterotriozakochiae* is smaller than *H.eurotiae* (in the latter species, the forewing length in mm according to Loginova (1960) is 1.80–1.90 in males, 1.95–2.00 in females; 1.78 in the male and 1.88–2.08 in the females from MMBC). *Heterotriozakochiae* differs from *H.eurotiae* mainly by a relatively broader and shorter paramere (in lateral view) with a less pronounced postero-apical tooth (Fig. 116c) and by the female proctiger and subgenital plate, both of which are slightly broader apically (in lateral view) than in *H.eurotiae* and the female proctiger is also straight dorsally (in females of *H.eurotiae*, the proctiger is distinctly concave dorsally, Fig. 116d). Both species probably also differ in their host plants: while *H.kochiae* was only reported from *Bassiaprostrata*, *H.eurotiae* was described from a number of specimens found on *Krasheninnikoviaceratoides* (L.) Gueldenst. (= *Eurotiaceratoides* (L.) C.A. Mey, Amaranthaceae) and most of the later records are also from this plant species (Loginova 1960, Gegechkori and Loginova 1990), with the exception of a record from '*Kochia* sp.' (= *Bassia* sp.) from Mongolia (Loginova 1972) and a record from '*Kochiacana*' (= *Bassiastellaris* (Moq.) Bornm.) from Iran (Burckhardt and Lauterer 1993), which should be re-examined.

#### Lauritrioza

Conci & Tamanini, 1986

3CF04B2F-939A-5D19-BB32-F965C2E0D178

#### Lauritrioza alacris

(Flor, 1861)

ABA8CB34-78CE-5247-9CDC-E3BD27FD9840

##### Feeds on

*Laurusnobilis* L., the only host plant of *L.alacris* (Conci and Tamanini 1985b, Bastin et al. 2023), is not native to Bulgaria (POWO 2024), but it is a widespread ornamental plant that has escaped from cultivation and naturalised in south-eastern Bulgaria along the Black Sea coast (Fidan et al. 2019, Vladimirov and Petrova 2023). Our record of adults of *L.alacris* in south-western Bulgaria comes from a laurel plant cultivated in a private garden.

##### Distribution

**General distribution.** Native to the Mediterranean parts of southern Europe, North Africa and Middle East; introduced with the host plant to Central and northern Europe, Great Britain, Ireland, Ukraine (Crimea), Caucasus and North and South Americas. **Distribution in Bulgaria** (Fig. 117b). Material examined: ROM.

##### Notes

New record for Bulgaria.

##### Diagnosis

**Adult** (Fig. 117a) **and fifth instar immature.** Conci and Tamanini (1985b), Ossiannilsson (1992).

#### Phylloplecta

Riley, 1884

7A4F3591-B25F-51FE-87A3-C037F66FD5E6

#### Phylloplecta trisignata

(Löw, 1886)

F76AE009-8432-5355-89F6-7251F5A3FD13

##### Feeds on

Reported from *Rubus* spp. by Głowacka (1989); *Rubus* spp. are known as host plants (Conci and Tamanini 1984a, Spodek et al. 2017).

##### Distribution

**General distribution.** Southern Europe, Middle East. **Distribution in Bulgaria** (Fig. 118b). Published records: PSP, ROP (Joakimov 1909, Głowacka 1989, Pramatarova et al. 2023). Material examined: BN, BS, PSP, PVS.

##### Diagnosis

**Adult** (Fig. 118a). Conci and Tamanini (1984a). **Fifth-instar immature.** Conci and Tamanini (1986).

#### Spanioza

Enderlein, 1926

3C097826-CA69-523A-ABF1-337A8B739A1F

#### Spanioza galii

(Foerster, 1848)

4BED683E-036E-5498-AA0F-FA7B6133E628

##### Distribution

**General distribution.** West Palaearctic, Central Asia (Burckhardt and Lauterer 2006). **Distribution in Bulgaria** (Fig. 119b). Published records: PT, RRE (Klimaszewski 1965). Material examined: BN, BS, DEL, DEP, DW, PBB, PBT, PKZ, PSP, RRE, RRW, SBM, SBW.

##### Notes

The specimens of '*Triozagalii*' published by Klimaszewski (1965) should be revised as they may be *S.velutina*. The two species were only later distinguished by Burckhardt and Lauterer (2006).

##### Diagnosis

**Adult** (Fig. 119a) **and fifth-instar immature.** Burckhardt and Lauterer (2006).

#### Spanioza velutina

(Foerster, 1848)

850515F3-D258-59F5-B191-BE33B3B25BA3

##### Distribution

**General distribution.** Palaearctic (Burckhardt and Lauterer 2006, Cho et al. 2022). **Distribution in Bulgaria** (Fig. 120b). Material examined: BN, BS, DEL, DEP, DW, PBS, PBT, RPR, RRE, RRW, SBE, SBW.

##### Notes

New record for Bulgaria.

##### Diagnosis

**Adult** (Fig. 120a). Burckhardt and Lauterer (2006).

#### Trichochermes

Kirkaldy, 1904

7FBB75AC-AB55-56F3-BC9F-33AE767CB0C9

#### Trichochermes rhamni

(Schrank, 1801)

E3155F7D-4EA9-5959-A43D-AA941328587C

##### Distribution

**General distribution.** Europe, Caucasus, Central Asia (Gegechkori and Loginova 1990). **Distribution in Bulgaria** (Fig. 121b). Material examined: SBW.

##### Notes

New record for Bulgaria.

##### Diagnosis

**Adult** (Fig. 121a) **and fifth-instar immature.** Ossiannilsson (1992).

#### Trichochermes walkeri

(Foerster, 1848)

BA6A7A9D-C27C-5E58-8EC2-9AEB2DF7A0A3

##### Feeds on

In Bulgaria, adults were collected on *Rhamnuscathartica* L. (Kuznetsova et al. 1995, Labina et al. 2014), which is a confirmed host plant (Ossiannilsson 1992).

##### Distribution

**Figure 122.**
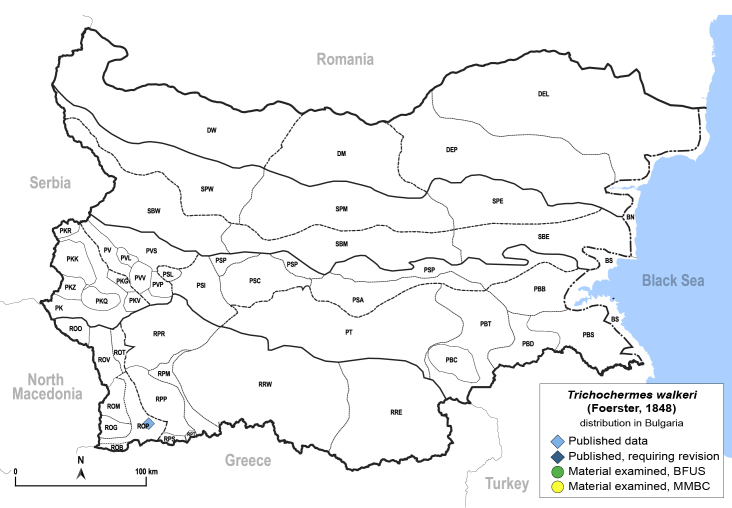
Distribution of *Trichochermeswalkeri* (Foerster, 1848) in Bulgaria.

**General distribution.** Europe, Caucasus, Central Asia (Gegechkori and Loginova 1990). **Distribution in Bulgaria** (Fig. 122). Published records: ROP (Labina et al. 2014).

##### Diagnosis

**Adult and fifth-instar immature.** Ossiannilsson (1992).

#### Trioza

Foerster, 1848

94F6A18D-691B-587D-B1A8-54119B9C93BE

#### Trioza cerastii

(Linnaeus, 1758)

9883DBD1-BCEF-5BA4-B863-FA0CFDE7B866

##### Distribution

**Figure 123.**
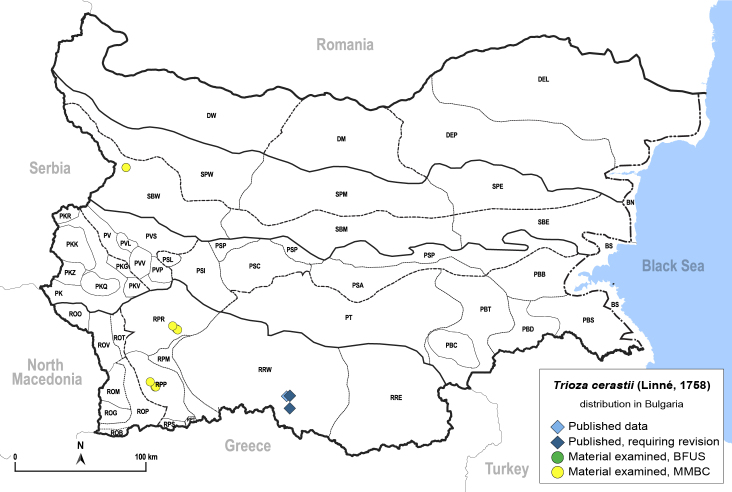
Distribution of *Triozacerastii* (Linnaeus, 1758) in Bulgaria.

**General distribution.** Europe. **Distribution in Bulgaria** (Fig. 123). Published records: RRW (Głowacka and Harizanov 1983). Material examined: RPP, RPR, SBW.

##### Diagnosis

**Adult and fifth-instar immature.** Ossiannilsson (1992).

#### Trioza flavipennis

Foerster, 1848

765200E8-0F27-5990-8F6C-FF1119DB8961

##### Distribution

**Figure 124.**
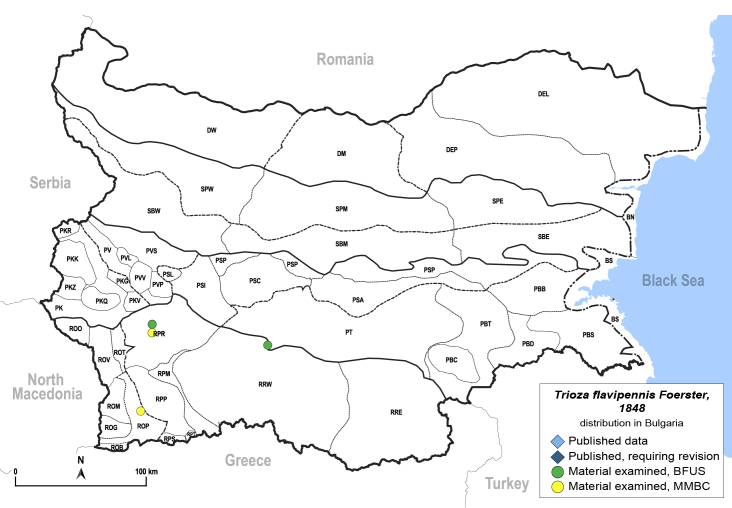
Distribution of *Triozaflavipennis* Foerster, 1848 in Bulgaria.

**General distribution.** Europe. **Distribution in Bulgaria** (Fig. 124). Material examined: RPR, RRW, ROP.

##### Notes

New record for Bulgaria.

##### Diagnosis

**Adult and fifth-instar immature.** Ossiannilsson (1992).

#### Trioza megacerca

Burckhardt, 1983

E6918919-B127-5BF8-A856-24B2612CAC3D

##### Distribution

**Figure 125.**
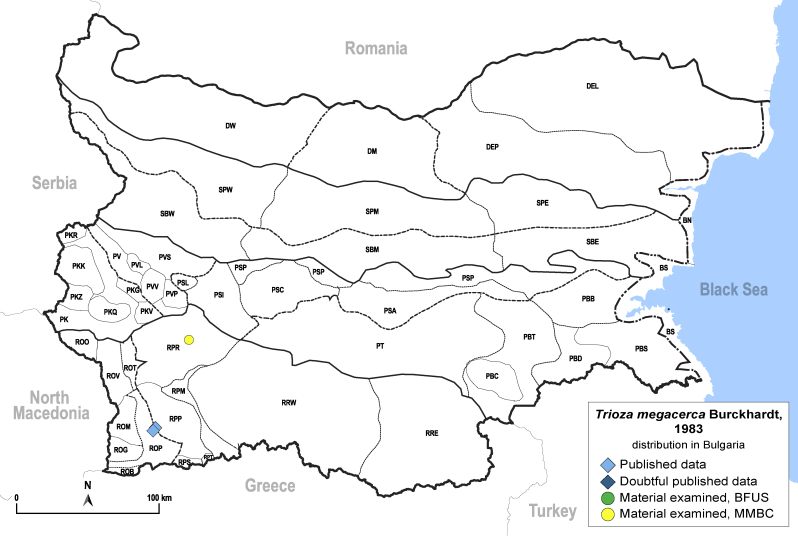
Distribution of *Triozamegacerca* Burckhardt, 1983 in Bulgaria.

**General distribution.** Southern parts of Central Europe and the Balkans. **Distribution in Bulgaria** (Fig. 125). Published records: ROP (Lauterer and Malenovský 2002). Material examined: RPR, ROP.

##### Diagnosis

**Adult.** Burckhardt (1983).

#### Trioza neglecta

Loginova, 1978

59DDA516-AF2C-5353-AE64-BF47D41E88F7

##### Feeds on

We collected adults and immatures on *Elaeagnusangustifolia* L., which is a confirmed host plant of *T.neglecta* (Loginova 1978, Lauterer and Janíček 1990). *Elaeagnusangustifolia* is not native to the Bulgarian flora. It is planted intentionally as an ornamental plant, to stabilise dry and eroded areas and in shelter belts. It has naturalised and become invasive in many regions of Bulgaria (Petrova et al. 2013).

##### Distribution

**General distribution.** Native to eastern parts of south-eastern Europe, Middle East and Caucasus, alien in Central Europe and the Balkans. **Distribution in Bulgaria** (Fig. 126b). Published records: SPE (Lauterer and Janíček 1990). Material examined: PVS, SPE.

##### Notes

*Triozaneglecta* is an alien species in the Bulgarian fauna. The first record from Bulgaria dates back to 1987 from the vicinity of a road between Antonovo and Kesarevo in the north-central part of the country (Lauterer and Janíček 1990).

##### Diagnosis

**Adult** (Fig. 126a). Dobreanu and Manolache (1962), as *Triozaelaeagni*; Loginova (1978).

#### Trioza proxima

Flor, 1861

E173FC1A-2192-599D-8173-B044DC5AA36B

##### Distribution

**General distribution.** Europe, Caucasus (Gegechkori and Loginova 1990). **Distribution in Bulgaria** (Fig. 127b). Published records: RPM, RPP, RRW (Klimaszewski 1970, Głowacka 1989). Material examined: BS, PSI, ROP, RRE.

##### Diagnosis

**Adult** (Fig. 127a). Burckhardt (1983).

#### Trioza remota

Foerster, 1848

AC8CA70E-E4E5-59B7-A516-E2D0B51C5C63

##### Feeds on

We collected adults on *Quercus* sp. in Bulgaria. The confirmed host plants from other countries are *Qu.petraea* (Matt.) Liebl., *Qu.pubescens* Willd. and *Qu.robur* L. (Ossiannilsson 1992, Seljak 2020).

##### Distribution

**Figure 128.**
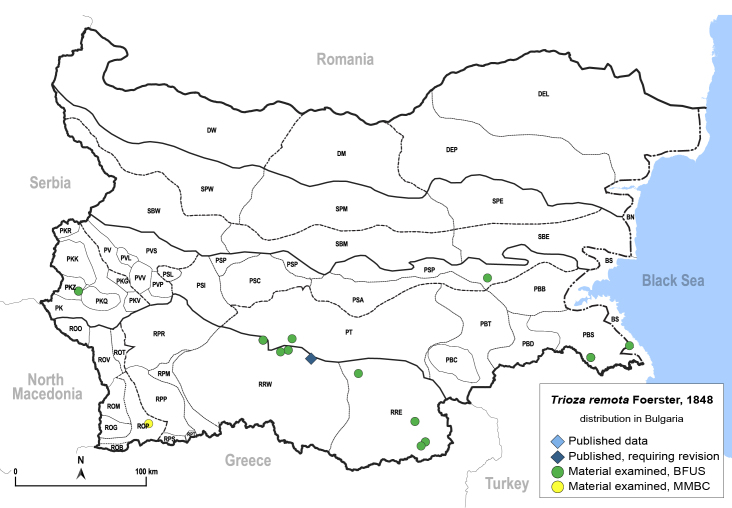
Distribution of *Triozaremota* Foerster, 1848 in Bulgaria.

**General distribution.** Europe, North Africa, Caucasus, Middle East. **Distribution in Bulgaria** (Fig. 128). Published records: RRW (Klimaszewski 1965). Material examined: BS, PBS, PKZ, PSP, PT, ROP, RRE, RRW.

##### Diagnosis

**Adult and fifth-instar immature.** Ossiannilsson (1992).

#### Trioza rotundata

Flor, 1861

53199CB4-F568-5DAB-A16E-B268F89C5D6D

##### Feeds on

Found on *Cardamineamara* L. in Bulgaria, which is a confirmed host plant (Burckhardt and Lauterer 2002a).

##### Distribution

**Figure 129.**
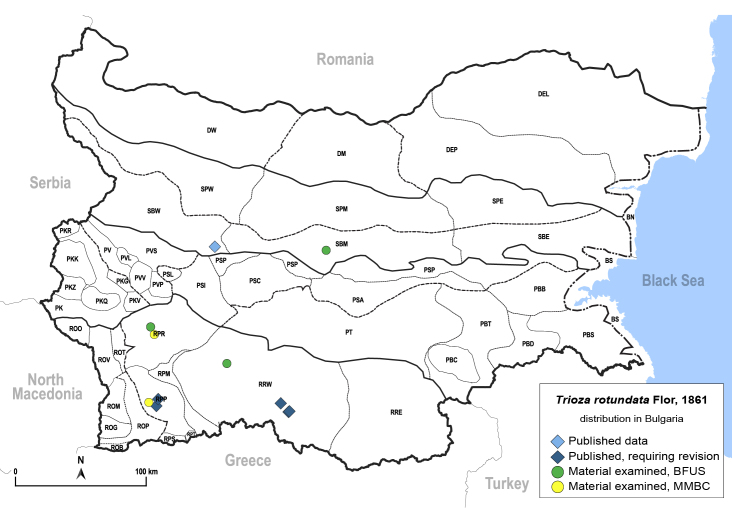
Distribution of *Triozarotundata* Flor, 1861 in Bulgaria.

**General distribution.** Europe, Caucasus. **Distribution in Bulgaria** (Fig. 129). Published records: RPP, RRW, SBW (Harizanov and Lauterer 1968, Głowacka and Harizanov 1983, Głowacka 1989, Burckhardt and Lauterer 2002a, Pramatarova et al. 2021). Material examined: RPP, RPR, RRW, SBM, SBW.

##### Diagnosis

**Adult and fifth-instar immature.** Burckhardt and Lauterer (2002a).

#### Trioza rumicis

Löw, 1880

CD8E9742-E4CD-5B5A-AFC1-F1BDF98D022E

##### Feeds on

Collected on *Rumex* sp. in Bulgaria (Głowacka and Harizanov 1983). Oligophagous and inducing galls on several *Rumex* spp. (Conci et al. 1996).

##### Distribution

**Figure 130.**
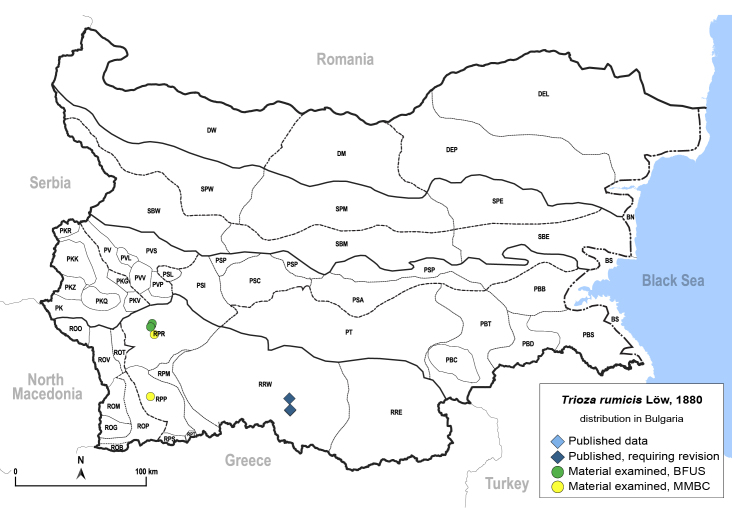
Distribution of *Triozarumicis* Löw, 1880 in Bulgaria.

**General distribution.** Central and south-eastern Europe, Caucasus, Iran (Gegechkori and Loginova 1990, Burckhardt and Lauterer 1993). **Distribution in Bulgaria** (Fig. 130). Published records: RRW (Głowacka and Harizanov 1983). Material examined: RPR, RPP.

##### Diagnosis

**Adult.** Dobreanu and Manolache (1962).

#### Trioza urticae

(Linnaeus, 1758)

C48EB531-E1E2-5371-A178-4DA660A09276

##### Feeds on

In Bulgaria, collected on *Urticadioica* L. (Głowacka and Harizanov 1983, Wonglersak et al. 2017), which is well known as a host plant (Ossiannilsson 1992).

##### Distribution

**General distribution.** Palaearctic. **Distribution in Bulgaria** (Fig. 131b). Published records: BS, PVV, ROP, RPP, RRW, SBW (Klimaszewski 1965, Głowacka and Harizanov 1983, Głowacka 1989, Wonglersak et al. 2017, Pramatarova et al. 2023). Material еxamined: BN, DEP, PBS, PKZ, PSI, PT, PVV, ROB, ROO, ROP, ROV, RPM, RPP, RPR, RPS, RRE, RRW, SBE, SBM, SBW, SPW.

##### Diagnosis

**Adult** (Fig. 131a) **and fifth-instar immature.** Ossiannilsson (1992).

### Doubtful records

#### Aphalara sauteri

Burckhardt, 1983

F0738037-3821-535D-9AA1-AF92E8D36739

##### Notes

The records of *A.sauteri* Burckhardt, 1983 published from the Pirin Mountains by Głowacka (1989) are attributed here to *A.nigrimaculosa*, based on the material collected in the same area and examined by the authors. Both *A.nigrimaculosa* and *A.sauteri* are similar in morphology and it is likely that Głowacka's material was misidentified. *Aphalarasauteri* is distributed in the Alps, Slovakia and Spain and its presence in Bulgaria is doubtful (Burckhardt and Lauterer 1997a).

#### Bactericera acutipennis

(Zetterstedt, 1828)

D72EE706-2BC5-5C48-9B6F-B13FBEB12D0E

##### Notes

*Bactericeraacutipennis* was recorded from Bulgaria (Rila Mountains) by Joakimov (1909) (as *Triozaacutipennis*). According to Klimaszewski (1965), this record probably refers to *Bactericerafemoralis*, for which the name *Triozaacutipennis* auct. nec Zetterstedt was erroneously and quite frequently used in literature (Ossiannilsson 1992, Burckhardt and Lauterer 1997b). We agree with Klimaszewski's opinion. The presence of *B.acutipennis* in Bulgaria cannot be excluded, as its host plant, *Comarumpalustre* L., occurs in the country, but further confirmation of *B.acutipennis* in Bulgaria is needed.

#### Bactericera reuteri

(Šulc, 1913)

21A6DF1D-289D-55AE-BE59-3B21DC40841F

##### Notes

Further confirmation is also needed for *B.reuteri*, which was recorded by Głowacka and Harizanov (1983) from conifers at high altitudes in the Rhodope Mountains. It is likely that this record concerns the closely-related, similar and recently-described *B.lyrata* (Seljak et al. 2008, Seljak and Malenovský 2014) or *B.harrisoni*, which was erroneously described and illustrated as '*Triozareuteri*' by Dobreanu and Manolache (1962) (see Conci and Tamanini (1985c)). The latter two species are confirmed in this paper on the basis of recently-collected material from Bulgaria. *Bactericerareuteri* is distributed in central and northern Europe, Russia and Mongolia and is monophagous on *Potentillaanserina* L. (Seljak et al. 2008). *Potentillaanserina* occurs in Bulgaria, but is restricted to the Danube Plain, with a maximum altitude of 300 m a.s.l. (Assyov et al. 2012), which makes the occurrence of *B.reuteri* at high altitude of the Rhodope Mountains unlikely.

#### Dyspersa apicalis

(Foerster, 1848)

728F82D7-C7B0-5309-995A-442365380110

##### Notes

The record of '*Triozaapicalis*' by Klimaszewski (1965) from Bulgaria (RRW) is doubtful according to Burckhardt (1986) as it could concern any species of the *Dyspersaapicalis* complex.

#### Dyspersa viridula

(Zetterstedt, 1828)

03FFDCAA-8EB3-5672-BDB9-C39EC4E90711

##### Notes

The name '*Triozaviridula*' was often misinterpreted in the past (Ossiannilsson 1972, Burckhardt 1986). The record of '*Triozaviridula*' by Joakimov (1909) from Bulgaria (PVV) could, according to Burckhardt (1986), concern any species of the *Dyspersaapicalis* complex. The records of '*Triozaviridula*' by Harizanov and Lauterer (1968), Klimaszewski (1970) and Głowacka and Harizanov (1983) are attributed here to *Dyspersacirsii*.

#### Eryngiofaga mesomela

(Flor, 1861)

2715C984-AD50-5F3A-AA02-553885E1370D

##### Notes

*Eryngiofagamesomela* was reported from Bulgaria by Joakimov (1909). However, this report probably refers to *E.dlabolai* (Vondráček, 1957), which was only later distinguished from *E.mesomela* (Pramatarova et al. 2021, Pramatarova et al. 2023). This is also supported by the lack of additional material of *E.mesomela* from Bulgaria, in contrast to numerous material of *E.dlabolai* (see above).

#### Trioza dispar

Löw, 1878

E84DDC86-E4C2-5479-8C4A-F009B862C072

##### Notes

Together with *T.foersteri* Meyer-Dür, 1871, *T.megacerca*, *T.proxima* and *T.tatrensis* Klimaszewski, 1965, *T.dispar* forms a group of morphologically and biologically similar species and the records of *T.dispar* published before the revision of the group by Burckhardt (1983) should be re-examined (see also Lauterer and Malenovský (2002)). This also applies to the only record of *T.dispar* from Bulgaria (Rila Mts.) by Joakimov (1909), as it could be, for example, *T.megacerca* or *T.proxima*, which have been confirmed more recently from this country. As the specimens were not available for revision (Pramatarova et al. 2023), we consider the occurrence of *T.dispar* in Bulgaria to be doubtful.

## Discussion

### Nomenclatural acts

The following new synonymy is proposed here:

*Colposceniaosmanica* Vondráček, 1953: 440 = *Colposceniakiritshenkoi* Loginova, 1960: 67, syn. nov.

*Colposceniaosmanica* was described by Vondráček (1953) on the basis of a large series of specimens collected in Turkey ('Ankara-Baraj, Anatolie, 3-4 July 1947, Feke, Toros, Anatolia, 12 August 1947, for many specimens of both sexes. Holotype and paratypes in the collections of National museum in Praha and those of author'). In the National Museum of Czechia in Prague (NMPC), we found 111 male and 85 female specimens, the labelling of which exactly corresponds with the published data on place and date of collection. All these specimens are dry-mounted (glued on pointed cardboard labels), but none of them was originally associated with an identification or type label, i.e. no holotype was actually fixed from the type series and labelled as such. There was only a label "Colposceniaosmanica Vond." pinned to the top of the box in which these specimens were kept. Due to the lack of labelling, the specimens were previously overlooked and omitted from the catalogue of the type specimens of Sternorrhyncha in the NMPC (Malenovský et al. 2016), but we have no doubt that they are part of the original type series of *C.osmanica*. The personal collection of Karel Vondráček in the Psylloidea collection in the Moravian Museum in Brno includes 10 microscopic slides with seven males, eight females and parts of other specimens of *C.osmanica* from 'Ankara - Baraj, 3-4.7.1947, Hoberlandt'; all slides were labelled as syntypes. In accordance with Article 74 of the ICZN (1999), we designate here a lectotype for *C.osmanica* in order to stabilise the nomenclature. The lectotype is a dry-mounted male deposited in the NMPC and labelled as follows: 'Ankara - Baraj, Anat. 3-4. VII 47, Exp. N Mus ČSR' and 'LECTOTYPUS, *Colposceniaosmanica* Vondráček, 1953, design. by I. Malenovský in Pramatarova et al. (2025)'. Six dry-mounted paralectotypes (3 males, 3 females) with the same locality data are newly deposited in MMBC, the rest of the dry-mounted paralectotypes are kept in NMPC.

*Colposceniakiritshenkoi* was described by Loginova (1960), based on specimens from Nakchivan in Azerbaijan. Although Loginova (1960) cited the work of Vondráček (1953), in the original description, she did not give any characters that distinguish *C.kiritshenkoi* from *C.osmanica*. In our opinion, the illustrations of the head, forewing and especially the male and female terminalia of *C.osmanica* by Vondráček (1953) and of *C.kiritshenkoi* by Loginova (1960) do not differ in any important details and show the same species. Loginova (1974b) later considered *C.osmanica* and *C.kiritshenkoi* as two valid species which she distinguished in her identification key of Palaearctic *Colposcenia* spp. mainly by the colouration of the body (green-yellow to yellow in *C.osmanica* vs. variegated, yellow-orange-crimson, with white spots on head and thorax in *C.kiritshenkoi*) and less clearly also by the forewing pattern (consisting of more or less well defined brownish spots on a yellowish background in *C.osmanica* vs. consisting of three brown stripes and spots in *C.kiritshenkoi*). Although Loginova (1974b) correctly stated that all *Colposcenia* species show a seasonal variation in colouration and indicated in the key that the above-mentioned differences in colouration between *C.osmanica* and *C.kiritshenkoi* should be compared in 'summer' specimens, she probably did not take into account that the type series of *C.osmanica* was collected in July and August, while the type specimens of *C.kiritshenkoi* were collected in April and, thus, could be different seasonal forms of the same species.

Both *C.osmanica* and *C.kiritschenkoi* have been reported from Bulgaria (Klimaszewski 1963, Klimaszewski 1965, Gegechkori and Loginova 1990). Many specimens of the two colour forms corresponding to *C.osmanica* (collected from late June to October) and *C.kiritshenkoi* (collected in April and May) were also examined by us from the collections of BFUS and MMBC. The two forms are sympatric in Bulgaria and we could not detect any differences in the morphology of the male and female terminalia (Fig. 12c). We also compared these specimens from Bulgaria with the type series of *C.osmanica* from Turkey (see above) and a specimen originally identified as *C.kiritshenkoi* by M. M. Loginova (deposited in the MMBC). We conclude that all these specimens are conspecific and propose to formally synonymise *C.kiritshenkoi* under *C.osmanica*. The synonymy of the two forms should be further supported by molecular data in the future. Pramatarova et al. (2024) published the partial sequences of *COI* and *cytb* gene fragments from specimens of both forms of *C.osmanica* collected in Bulgaria, but each of their sequences was unfortunately extracted from different specimens (COI - *C.kiritshenkoi*, cytb - *C.osmanica* *s.str.*) and the data cannot be directly compared between the two seasonal forms at the moment.

According to Spodek et al. (2017), further studies are needed to determine whether *C.kiritshenkoi* (and thus *C.osmanica*) is conspecific with *Colposcenialurida* (Scott, 1880). The identity of *C.lurida* is poorly known - it was described on the basis of a single male from the Caucasus (Scott 1880) and has never been re-described or illustrated. Recently, *C.lurida* was reported from Israel (Spodek et al. 2017).

### Diversity, distribution and host associations of Psylloidea in Bulgaria

Prior to this work, 113 species of psyllids have been recorded from Bulgaria in 51 publications. We consider the records of seven species (*Aphalarasauteri*, *Bactericeraacutipennis*, *B.reuteri*, *Dyspersaapicalis*, *D.viridula*, *Eryngiofagamesomela* and *Triozadispar*) as doubtful because they may have been misidentified. The published records from Bulgaria (Joakimov 1909, Klimaszewski 1965, Głowacka and Harizanov 1983, Głowacka 1989) precede taxonomic revisions in which relevant diagnostic characters for distinguishing closely-related and similar species were presented and we could not confirm the occurrence of these particular species in Bulgaria from the material we collected or examined. It is likely that some of these records were misidentified because similar, allopatric species are confirmed here from Bulgaria (*Aphalaranigrimaculosa*, *Eryngiofagadlabolai*) or the species names are known to have been frequently misinterpreted in the past (*B.acutipennis*, *D.viridula*). On the other hand, *B.acutipennis*, *B.reuteri*, *D.apicalis* and *T.dispar* (in their current taxonomic interpretations) are widely distributed in Europe (Burckhardt 1983, Burckhardt 1986, Ossiannilsson 1992, Seljak et al. 2008) and their host plants occur in Bulgaria (POWO 2024), so the occurrence of these psyllid species in the country is possible, but awaits proper documentation. *Arytainagenistae*, *Craspedoleptalatior*, *Cacopsyllanigrita*, *Dyspersamesembrina* and *Trichochermeswalkeri* are also known from Bulgaria only from previous publications (Klimaszewski 1965, Głowacka and Harizanov 1983, Burckhardt 1986, Labina et al. 2014) and are missing in the collections that were accessible to us. Although a revision of the relevant material and confirmation of these species by newly-collected material in the field would be desirable, we consider these data to be trustworthy. For 105 species previously published from Bulgaria, we were able to confirm their occurrence in the country on the basis of material that we personally examined. One species name, *Colposceniakiritshenkoi*, is newly synonymised and 25 species are recorded here for the first time from Bulgaria.

To summarise, a total of 130 species of jumping plant lice have been identified in Bulgaria to date. They belong to six families and 33 genera: Aphalaridae (8 genera, 31 species), Calophyidae (1 genus, 1 species), Carsidaridae (1 genus, 1 species), Liviidae (6 genera, 15 species), Psyllidae (8 genera, 45 species) and Triozidae (9 genera, 37 species). Compared to the numbers of psyllid species known from neighbouring countries and other countries of the Balkan Peninsula from where comprehensive faunistic data are available (Greece: 89 spp., Burckhardt (1987); Romania: 86 spp., Dobreanu and Manolache (1962); Serbia: 63 spp., Jerinić-Prodanović (2010); Turkey: 101 spp., Drohojowska and Burckhardt (2014); Croatia: 86 spp., Pintar (2023); Slovenia: 126 spp., Seljak (2020); see also Ouvrard (2020) and Suppl. material 1), the diversity of jumping plant lice in Bulgaria seems to be relatively high, although most of these countries have not been studied with the same intensity and, with the exception of Slovenia, a significantly higher number of species can be expected in them given their size and the diversity of natural conditions. To a certain extent, this also applies to Bulgaria, as 72 other species are known from at least one of the neighbouring countries (Greece, North Macedonia, Romania, Serbia or Turkey), but have not yet been found in Bulgaria (Suppl. material 1). Although our list is quite comprehensive, we expect that new taxa will be identified in the Bulgarian psyllid fauna in the future.

From a biogeographical point of view, the currently known psyllid fauna of Bulgaria is mainly composed of species that are widely distributed in the Palaearctic (36 spp.), the Western Palaearctic (22 spp.) or in Europe (10 spp.), some even having a Holarctic distribution (5 spp.).

On the other hand, many species have a more restricted distributional pattern. Some of them represent Mediterranean, sub-Mediterranean or Ponto-Mediterranean elements and they reach the northern limits of their general distribution (or at least their distribution in eastern Europe) in southern or central Bulgaria or on the Bulgarian Black Sea coast, often together with their host plants, such as *Agonoscenapistaciae*, *A.targionii*, *Megagonoscenagallicola* and *M.viridis* on *Pistaciaterebinthus*, *Colposceniaaliena* and *C.traciana* on *Tamarix* spp., *Euphylluraphillyreae* on *Phillyrealatifolia*, *Strophingiacinereae* on *Ericaarborea*, *Cacopsyllanotata* on *Pyrusspinosa* and *Psyllacolorata* on *Ostryacarpinifolia* or they seem to be limited by the climate, as their host plants are more widespread, such as *Aphormalichenoides* on *Ranunculusbulbosus*, *Camarotoscenasubrubescens* on *Populus* spp., *Liviamediterranea* on *Carexhumilis* and *C.pontica*, *Bactericeratrigonica* on Apiaceae and *Phylloplectatrisignata* on *Rubus* spp. (Spodek et al. 2017, Burckhardt et al. 2023).

*Homotomaficus*, *Diaphorinalycii*, *Spanioneurabuxi*, *S.fonscolombii* and *Lauritriozaalacris* are also widespread in the Mediterranean Region (and in the Caucasus and/or the Middle East, *D.lycii* also in Central Asia), but their host plants are not indigenous, i.e. they were introduced to Bulgaria and none of these psyllid species can be considered autochthonous in the Bulgarian fauna, although they reproduce outdoors in Bulgaria and some have even been found in natural habitats (*H.ficus*, *D.lycii*). The status of *Cacopsyllapulchella* is unclear, as its host plant, *Cercissiliquastrum*, is native to eastern Bulgaria (Assyov et al. 2012), but we only detected the psyllids on cultivated ornamental trees in an urban environment. *Calophyarhois*, associated with *Cotinuscoggygria*, is another psyllid species widely distributed in the Mediterranean, Middle East and Asia, relatively common in parks and gardens or along roads. While *C.rhois* is alien in several European countries (Burckhardt and Mühlethaler 2003, Mifsud et al. 2010), its host plant is hypothesised to be native to Bulgaria (Assyov et al. 2012, POWO 2024). In this case too, its status in Bulgaria should be confirmed by fieldwork in natural habitats. In addition to these species, *Livillavariegata* (native to southern Europe and the Alps), *Triozaneglecta* (native to eastern parts of south-eastern Europe, the Middle East and the Caucasus) and *Acizziajamatonica* (native to eastern Asia) have clearly been unintentionally introduced to Bulgaria with their host plants, which are used as ornamentals or for erosion control, but in the first two cases, naturalised and are invasive (Petrova et al. 2013). In total, therefore, at least eight species (6% of the Bulgarian psyllid fauna) can be regarded as alien (non-native, adventive) and naturalised in Bulgaria to date.

Several species reach the westernmost limits of their known natural distribution in Bulgaria. These species are either mainly distributed in the steppe vegetation of southern Ukraine, Russia and Central Asia and/or semi-arid regions of the Middle East and the northern Caucasus, such as *Colposceniaosmanica*, *Craspedoleptaaraneosa*, *Psyllopsismachinosa*, *Eryngiofagababugani* and *Heterotriozakochiae* (Gegechkori 1984, Gegechkori and Loginova 1990) or in the mountains of north-eastern Turkey and/or the Caucasus, such as *Aphalaranigrimaculosa*, *Colposceniabidentata* and *Dyspersakantshavelii* (Gegechkori 1984, Burckhardt 1988, Burckhardt and Lauterer 1997a).

Other "steppic" elements, mainly associated with open dry grasslands or saline habitats, extend from Central Asia, the Middle East or the Caucasus across the Balkans and reach their western distribution limit in central or south-western Europe: *Craspedoleptaartemisiae*, *C.bulgarica*, *C.innoxia*, *C.pontica*, *Eumetoecuskochiae*, *Rhodochlanisbicolor*, *Bactericeraperrisii*, *Heterotriozadichroa* and *Eryngiofagadlabolai* (Lauterer 1965, Loginova 1977, Conci et al. 1993, Lauterer and Malenovský 2002, Seljak 2020).

Another group of species is known to be restricted to south-eastern and central Europe - it includes species associated with open thermophilous woodlands and dry grasslands (*Psyllopsismeliphila*, *Arytainamaculata*, *Arytainillaspartiicola*, *Livillacognata*, *L.horvathi*, *L.radiata* and *Triozamegacerca*) and ruderal habitats (*Craspedoleptaconspersa*, *Bactericeralyrata*) (Conci and Tamanini 1983, Conci and Tamanini 1985a, Hodkinson and Hollis 1987, Conci and Tamanini 1990, Lauterer and Malenovský 2002, Malenovský et al. 2011, Seljak and Malenovský 2014). *Psyllopsisdobreanuae*, which was newly reported here for Bulgaria, was previously only known from eastern Romania, North Macedonia and Moldova (Loginova 1971, Malenovský and Jerinić-Prodanović 2011).

A large part of Bulgaria is covered by mountains and a significant part of the country's biodiversity is associated with montane and alpine habitats. A relatively large number of psyllid species in Bulgaria are also restricted to the mountains. These include: i) species with predominantly boreal distribution in the northern parts of Eurasia and rather isolated occurrences in the mountains or highlands of central and south-eastern Europe, such as *Aphalaraaffinis*, *Craspedoleptanebulosa*, *C.subpunctata*, *Cacopsyllacorcontum*, *C.myrtilli*, *C.sorbi*, *Bactericerabohemica*, *B.femoralis*, *Dyspersaabdominalis*, *D.cirsii*, *D.munda*, *D.pallida*, *Triozacerastii* and *T.rotundata* (Lauterer 1976, Ossiannilsson 1992, Conci et al. 1993, Conci et al. 1996, Labina et al. 2009, Malenovský et al. 2014); ii) species apparently endemic to the mountains of central and south-eastern Europe (Alps, Carpathians and Balkans), such as *Psyllaalpina*, *Bactericeraharrisoni* and *Dyspersamesembrina* (Conci and Tamanini 1985c, Burckhardt 1986, Conci et al. 1993, Conci et al. 1996); iii) species more widely distributed in the Alps, the Carpathians, the Balkans, the Caucasus and the Alborz in northern Iran, such as *Triozarumicis* (Conci et al. 1996, Burckhardt and Lauterer 1993) and iv) species restricted to the Balkans, the Pontic Mountains and the Caucasus, such as *Aphalaranigrimaculosa* and *Dyspersakantshavelii* mentioned above. A special area of nature conservation importance in Bulgaria is the Strandzha Mountains in the south-east of the country with its mixed stands of *Callunavulgaris* and *Ericaarborea* heaths (Gussev 2015), where only *Strophingiaericae* and *S.cinereae* have been recorded.

**Figure 132.**
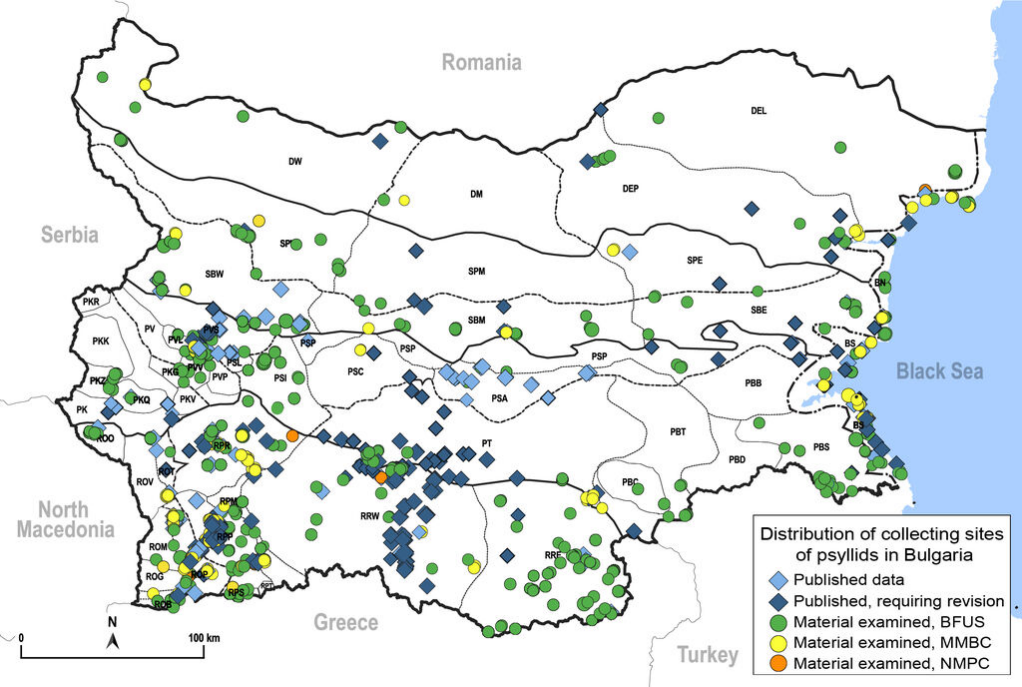
Distribution of collecting sites of psyllids in Bulgaria.

However, these conclusions should be regarded as provisional as the knowledge of the distribution of many psyllid species is incomplete and the knowledge of the Bulgarian psyllid fauna is also uneven at the regional level. Most of the data come from the south-western part of the country (Fig. 132). This explains why the Krupnik-Sandanski-Petrich Valley has the highest number of 58 recorded species, followed by the Pirin and Rila mountains, with 50 psyllid species each. In contrast, the regions of the Ruy, Verila, Plana, Ograzhden and Sturgach Mountains, Kraishte and Strandzha-Dervent Districts have been neglected and lack data, while the Danubian Plain and south-eastern Bulgaria remain poorly studied.

The knowledge of the host plants of the Bulgarian psyllids is also quite limited, as most records lack precise information on the host plants and, when this is present in the collections, it is rarely associated with material of psyllid immatures, so that these plant taxa can be considered as 'confirmed hosts' (Burckhardt et al. 2014). Nevertheless, we provide here new host plant information for ten psyllid species, some of which are also confirmed by the presence of immatures. These records indicate a regional specificity of the psyllid species in Bulgaria, as these species were previously known to be associated with other, albeit closely related, plant taxa in western, central and southern Europe (*Colposceniatraciana*, *Craspedoleptapontica*, *Arytainamaculata*, *Arytainillaspartiicola*, *Cacopsyllabidens*, *Livillacognata*, *L.horvathi*, *Dyspersacirsii*), Caucasus (*Aphalaranigrimaculosa*) or Central Asia (*Craspedoleptaaraneosa*).

Although there is still clearly much work to be done to fully understand the Bulgarian psyllid fauna, this study serves to illustrate the relatively high species diversity of psyllids in this country, especially due to its particular geographical location and its diverse natural habitats, despite a relatively small area.

## Supplementary Material

751EE30D-E0BD-5AC2-B622-F39C79205356 10.3897/BDJ.13.e147277.suppl1 Supplementary material 1 Comparison of psyllid diversity in Bulgaria and other Balkan countries and Turkey Data type review File: oo_1281180.pdf https://binary.pensoft.net/file/1281180 Pramatarova M., Malenovský I., Gjonov I.

## Figures and Tables

**Figure 2a.**
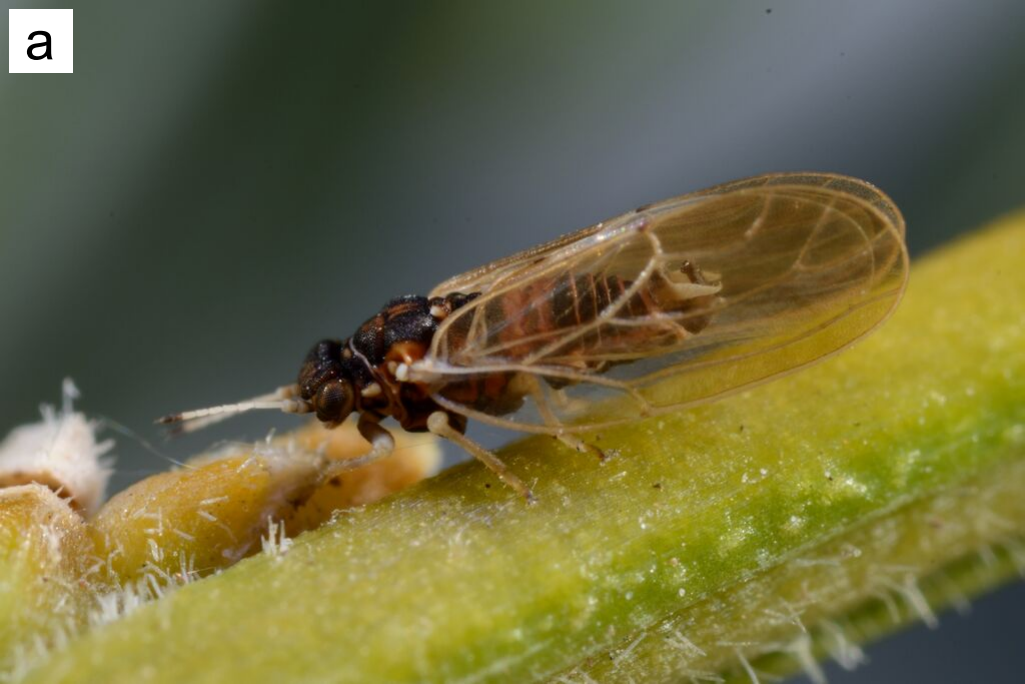
*Aphalaraaffinis*, adult male;

**Figure 2b.**
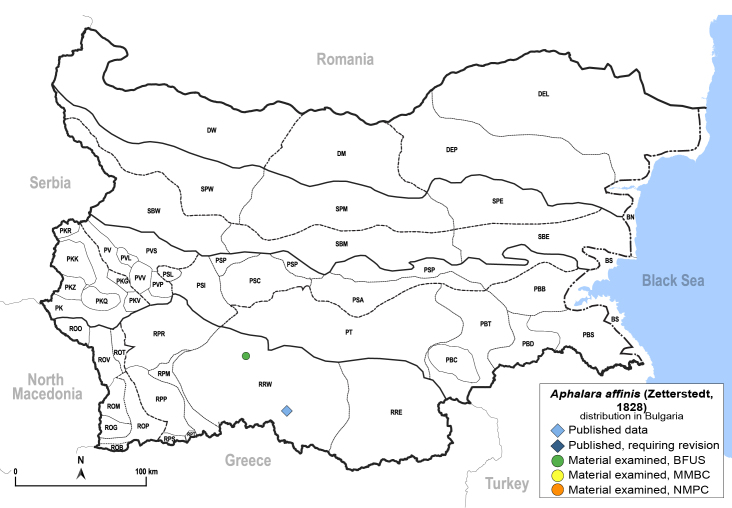
Distribution of *Aphalaraaffinis* in Bulgaria.

**Figure 3a.**
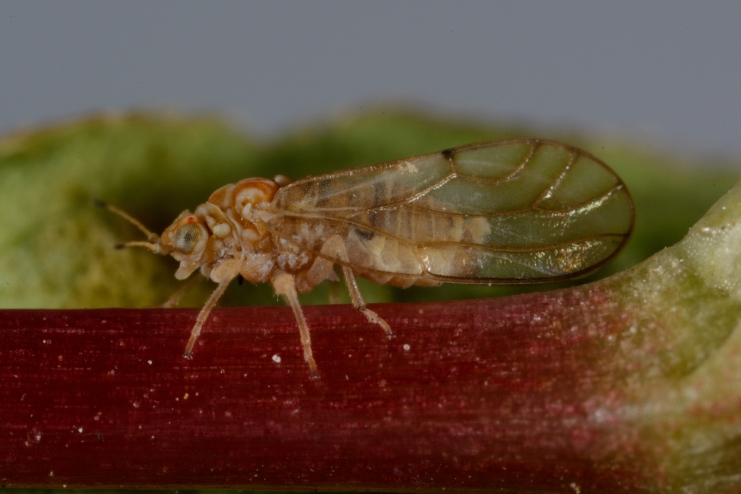
*Aphalaraavicularis*, adult male;

**Figure 3b.**
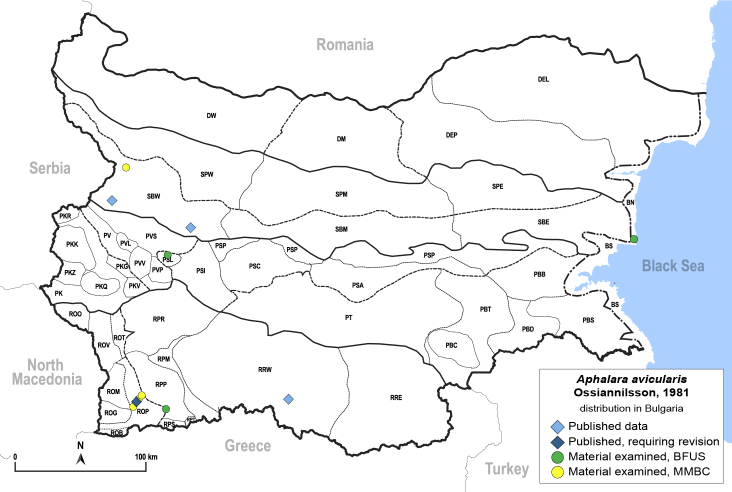
Distribution of *Aphalaraavicularis* in Bulgaria.

**Figure 7a.**
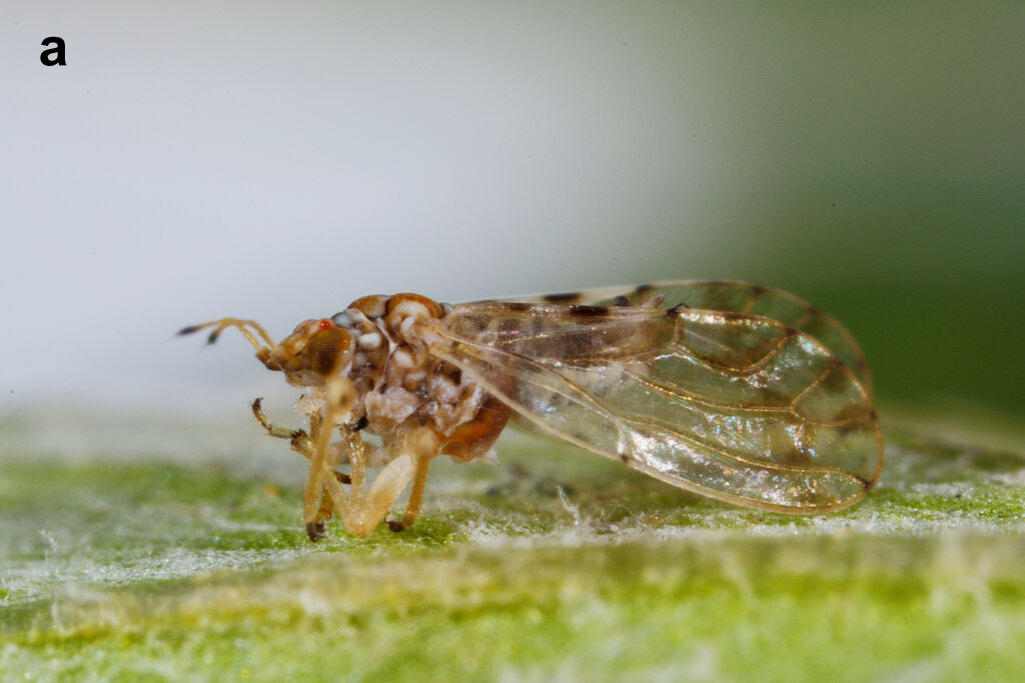
*Aphalaramaculipennis*, adult male (dead specimen);

**Figure 7b.**
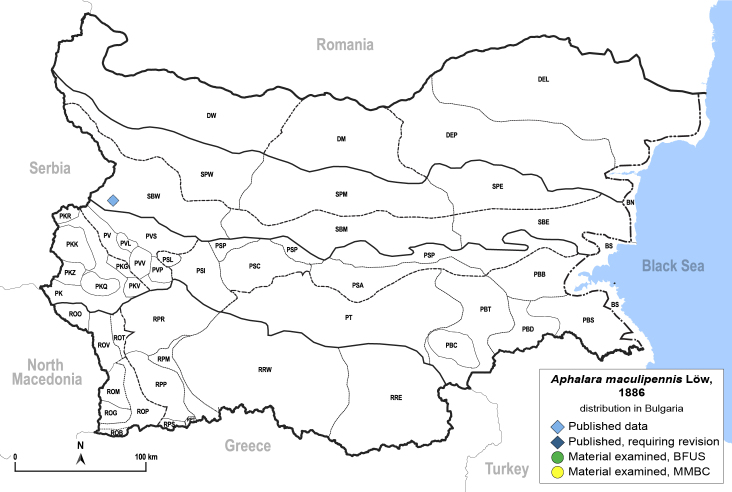
Distribution of *Aphalaramaculipennis* in Bulgaria.

**Figure 8a.**
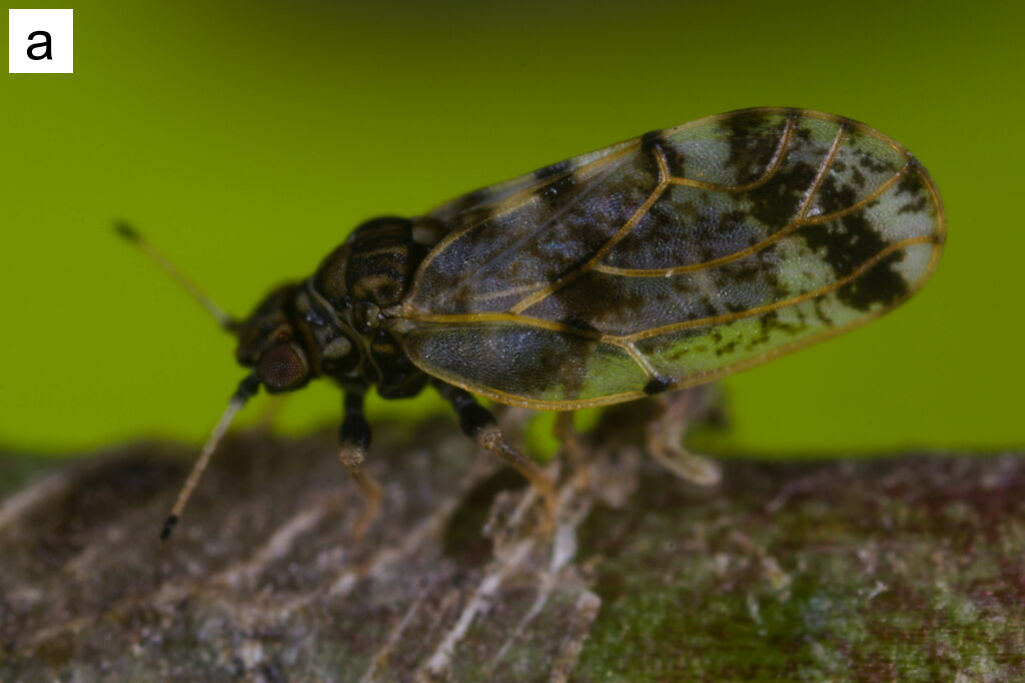
*Aphalaranigrimaculosa*, adult female;

**Figure 8b.**
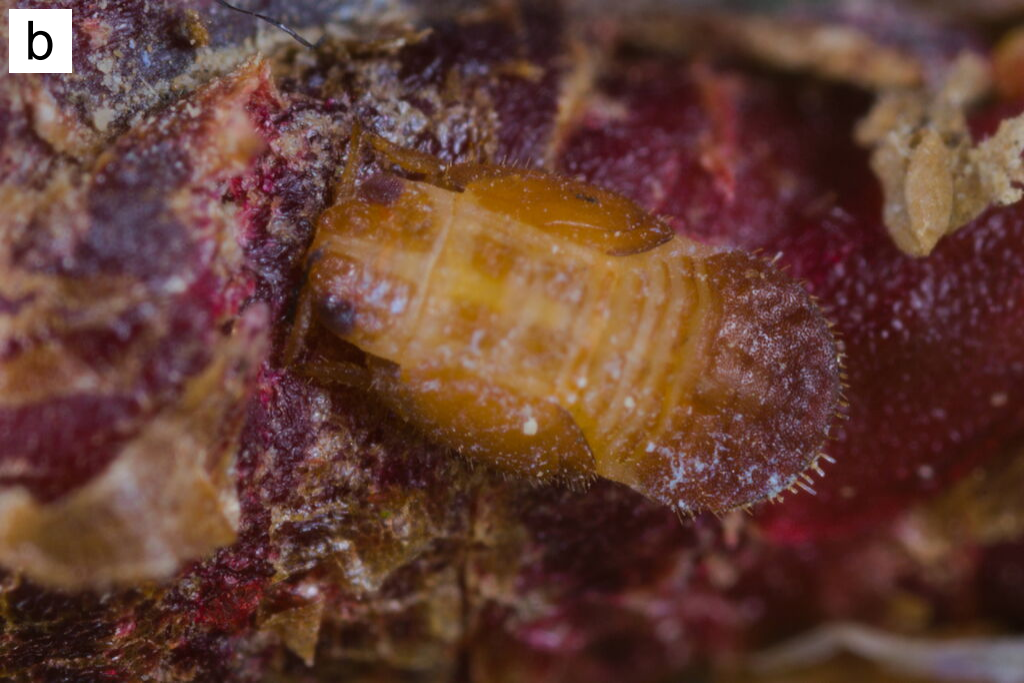
*Aphalaranigrimaculosa*, immature on *Rumexacetosella*;

**Figure 8c.**
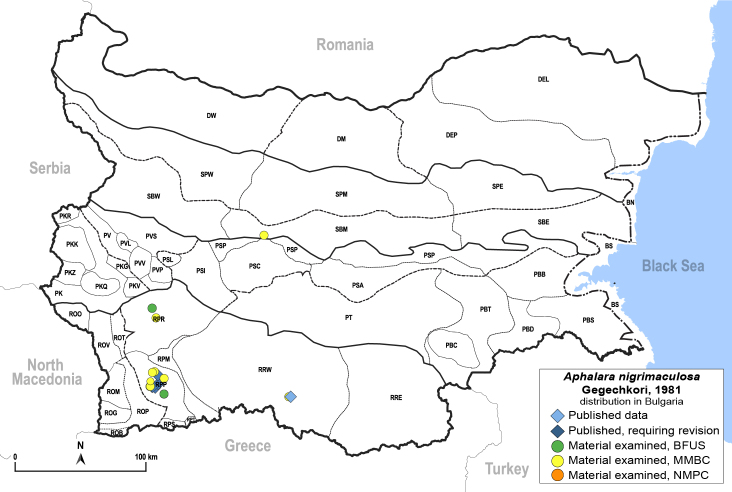
Distribution of *Aphalaranigrimaculosa* in Bulgaria.

**Figure 10a.**
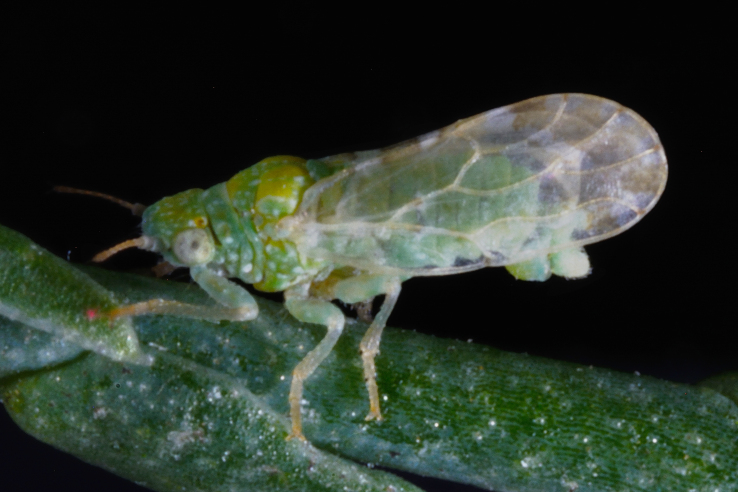
*Colposceniaaliena*, adult male;

**Figure 10b.**
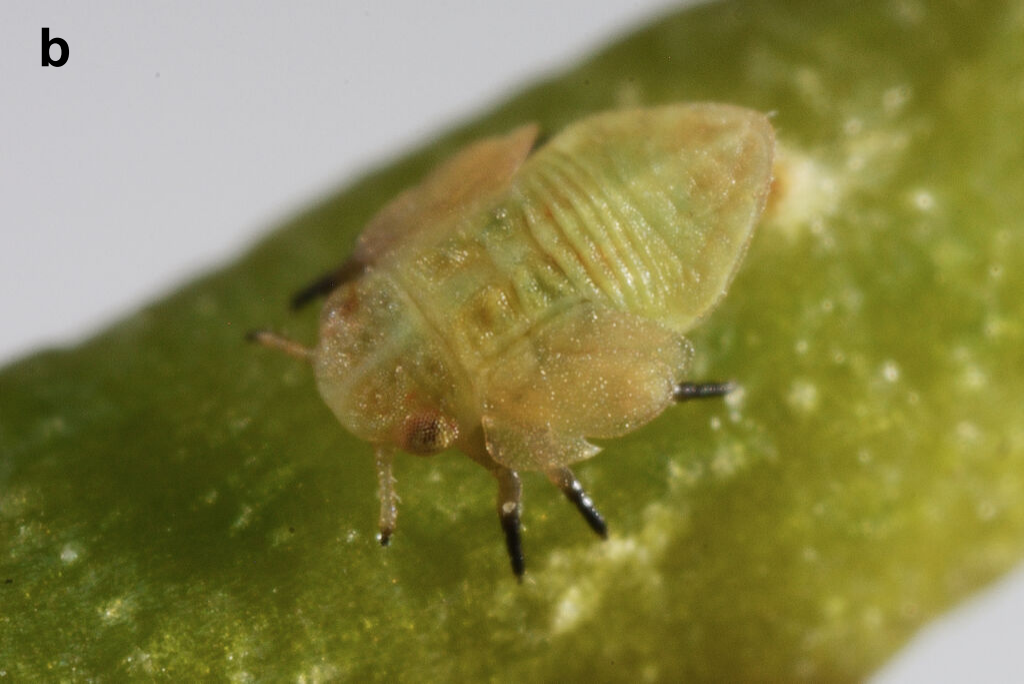
*Colposceniaaliena*, immature;

**Figure 10c.**
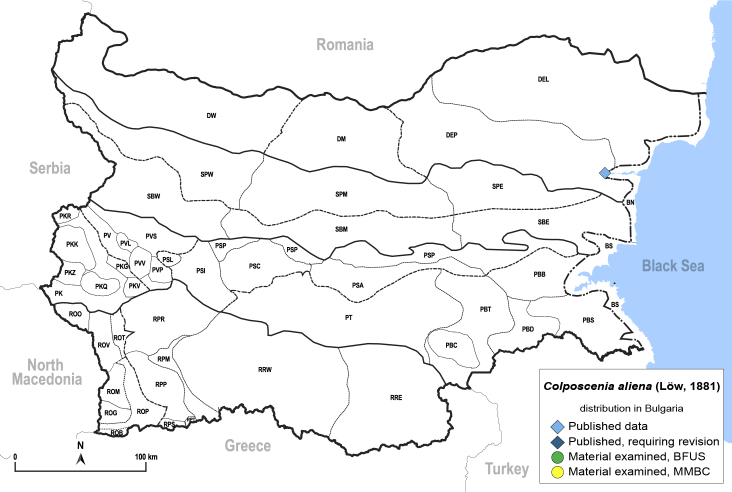
Distribution of *Colposceniaaliena* in Bulgaria.

**Figure 11a.**
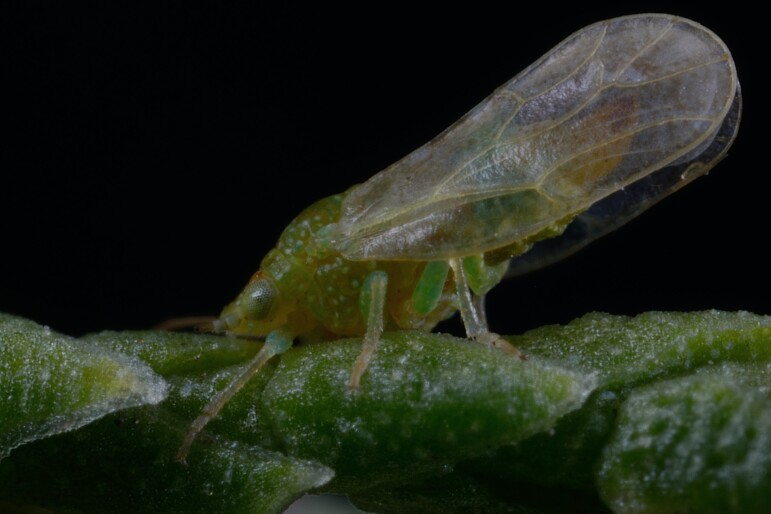
*Colposceniabidentata*, adult, female;

**Figure 11b.**
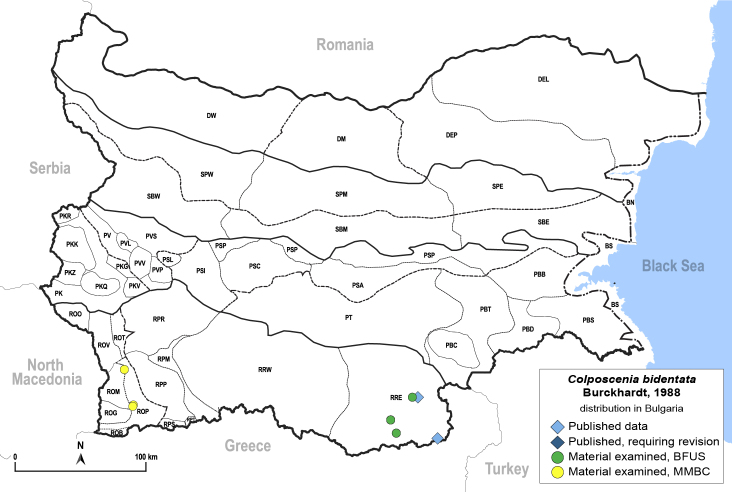
Distribution of *Colposceniabidentata* in Bulgaria.

**Figure 12a.**
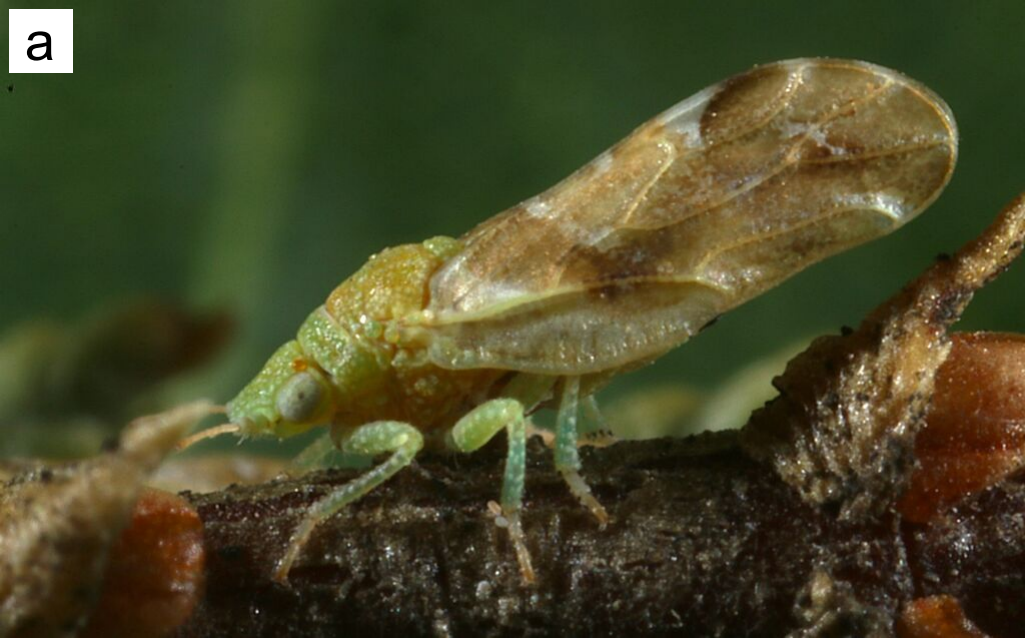
*Colposceniaosmanica*, adult female, summer specimen;

**Figure 12b.**
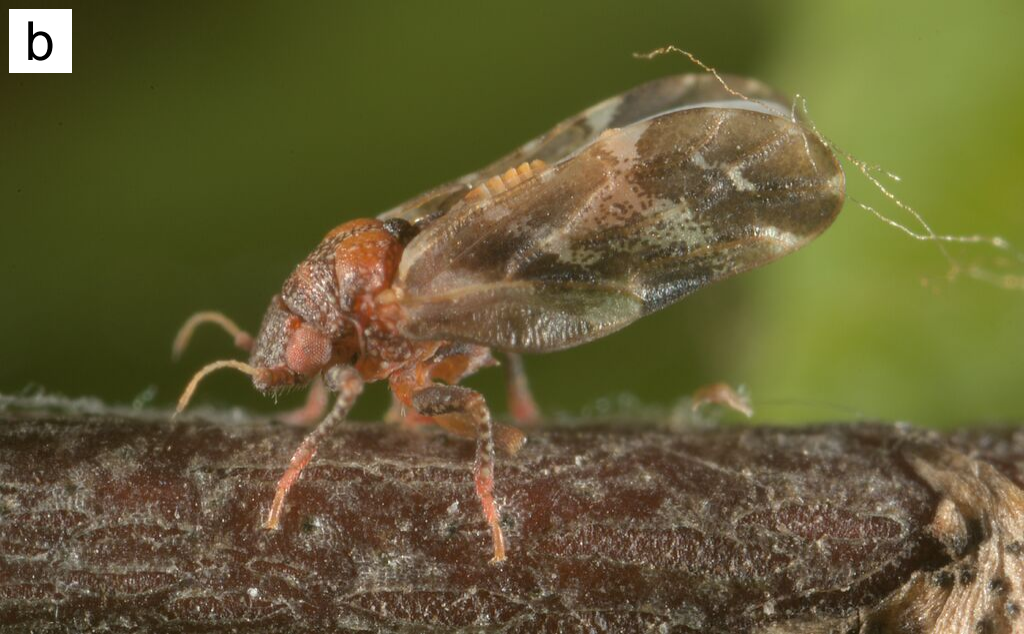
*Colposceniaosmanica*, adult male, overwintering specimen;

**Figure 12c.**
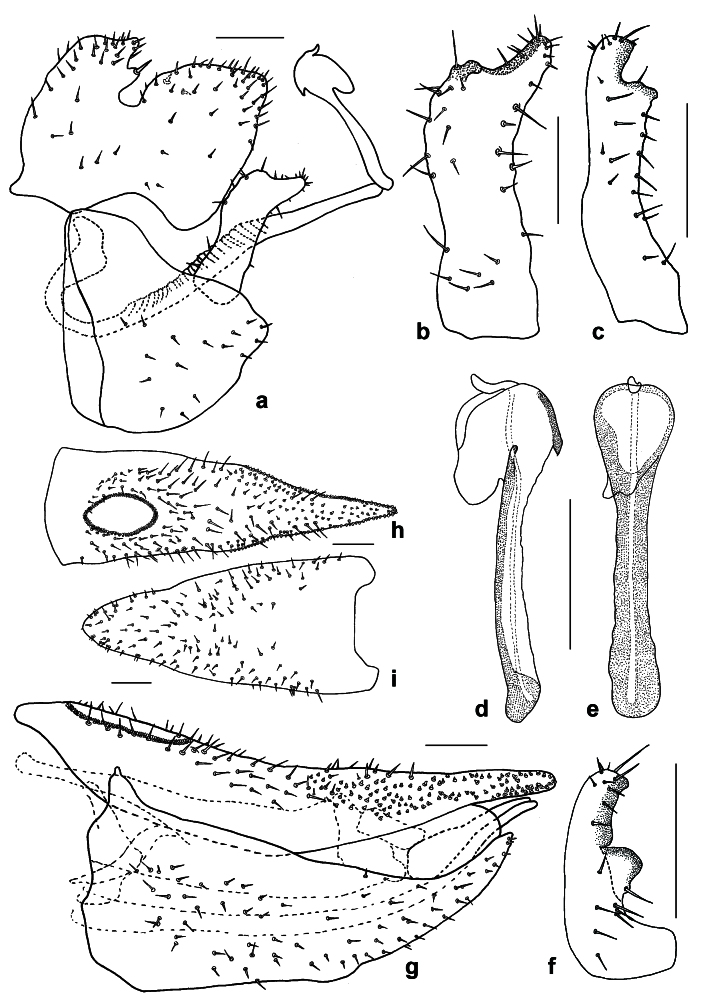
*Colposceniaosmanica*, male and female terminalia. **a** – male terminalia, lateral view; **b** – paramere, inner surface, lateral view; **c** – paramere, posterior view; **d** – distal segment of aedeagus, lateral view; **e** – distal segment of aedeagus, dorsal view; **f** – paramere, dorsal view; **g** – female terminalia, lateral view; **h** – female proctiger, dorsal view; **i** – female subgenital plate, ventral view. Scale bars: 0.1 mm;

**Figure 12d.**
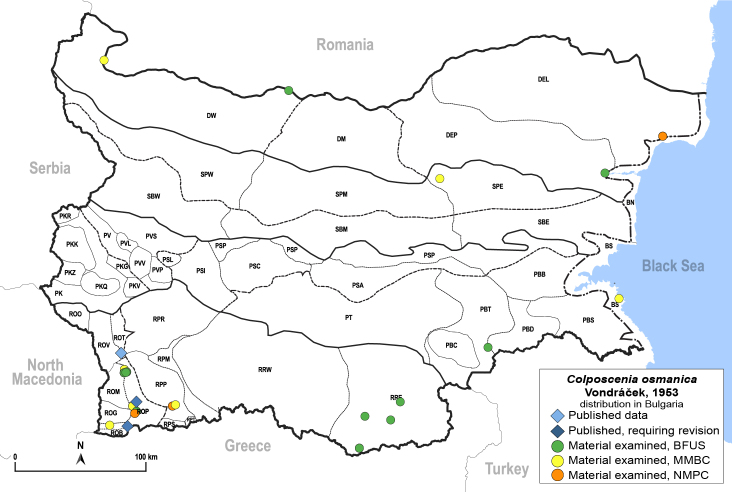
Distribution of *Colposceniaosmanica* in Bulgaria.

**Figure 13a.**
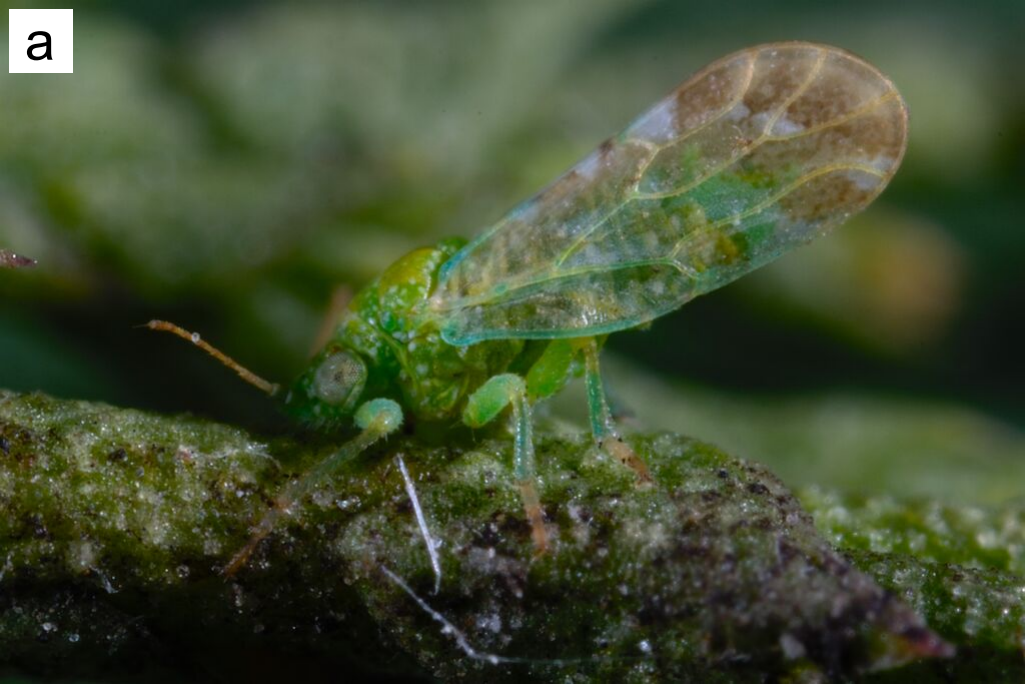
*Colposceniatraciana*, adult male;

**Figure 13b.**
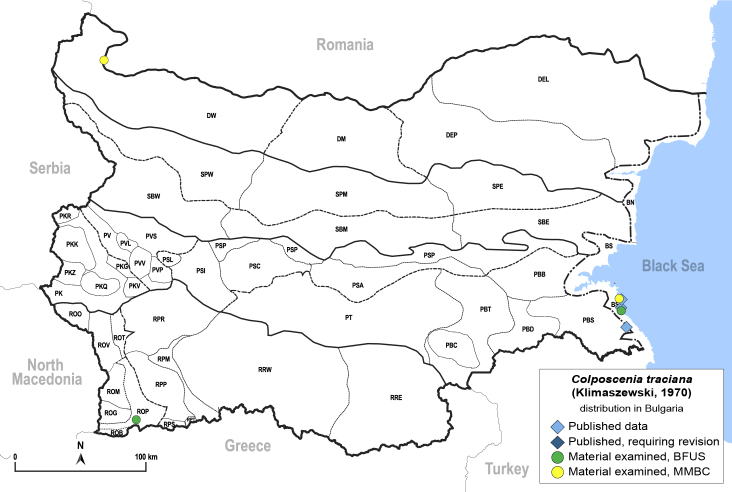
Distribution of *Colposceniatraciana* in Bulgaria.

**Figure 14a.**
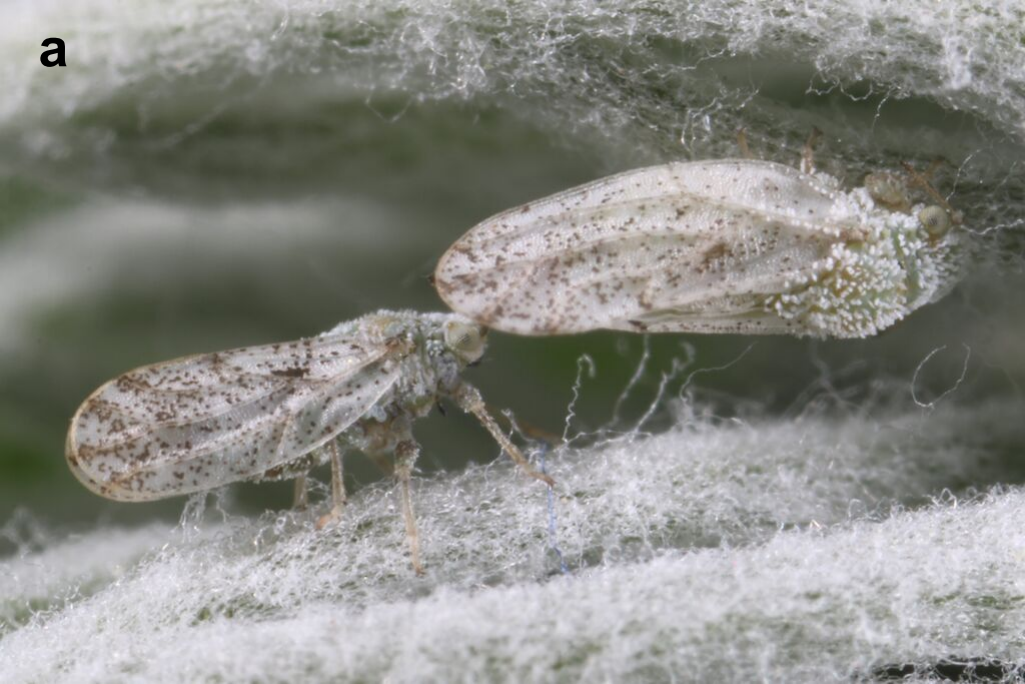
*Craspedoleptaaraneosa*, adult male and female;

**Figure 14b.**
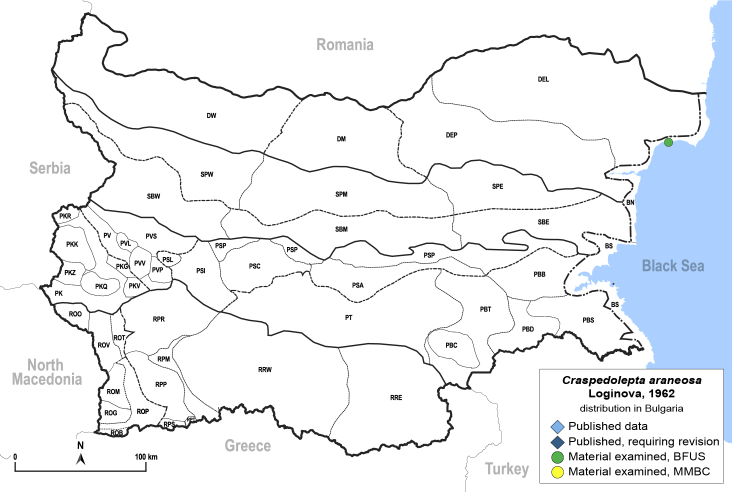
Distribution of *Craspedoleptaaraneosa* in Bulgaria.

**Figure 16a.**
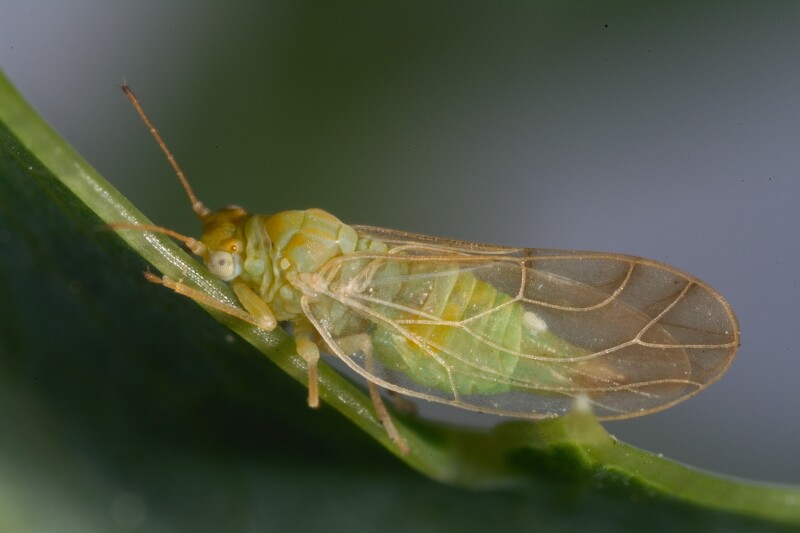
*Craspedoleptabulgarica*, adult female;

**Figure 16b.**
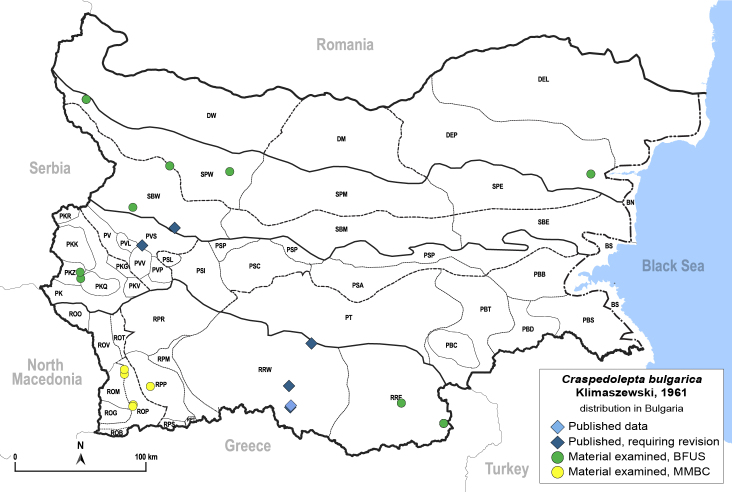
Distribution of *Craspedoleptabulgarica* in Bulgaria.

**Figure 18a.**
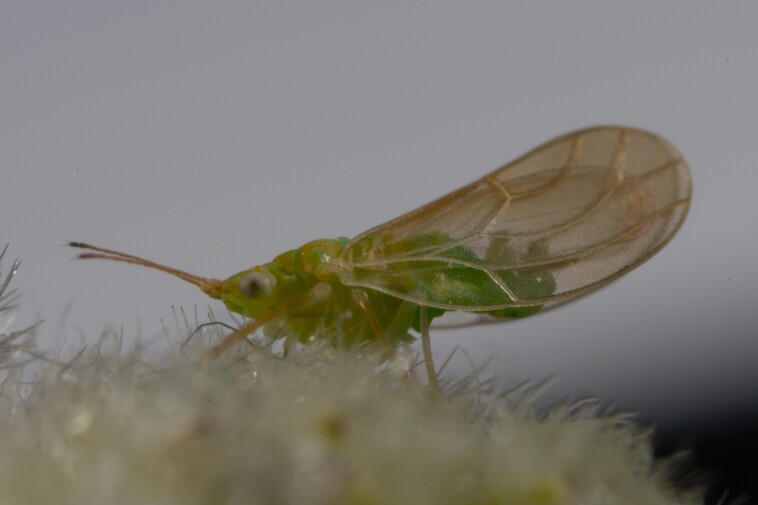
*Craspedoleptainnoxia*, adult male;

**Figure 18b.**
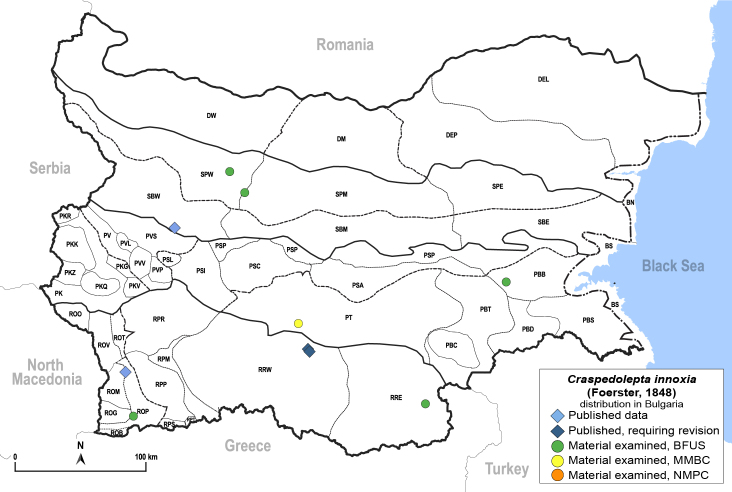
Distribution of *Craspedoleptainnoxia* in Bulgaria.

**Figure 20a.**
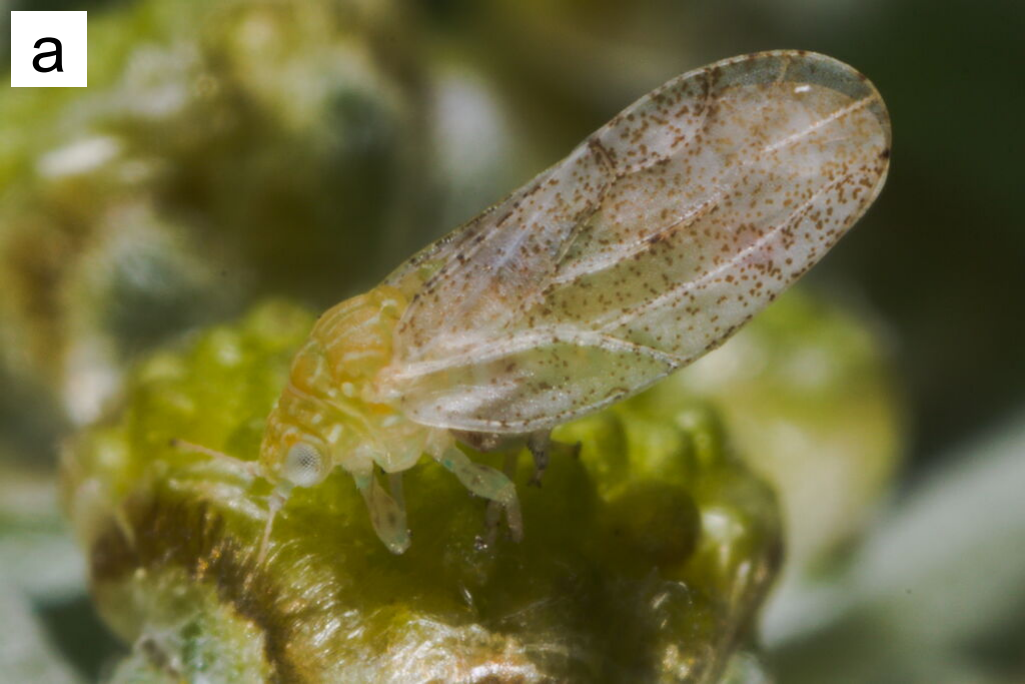
*Craspedoleptamalachitica*, adult;

**Figure 20b.**
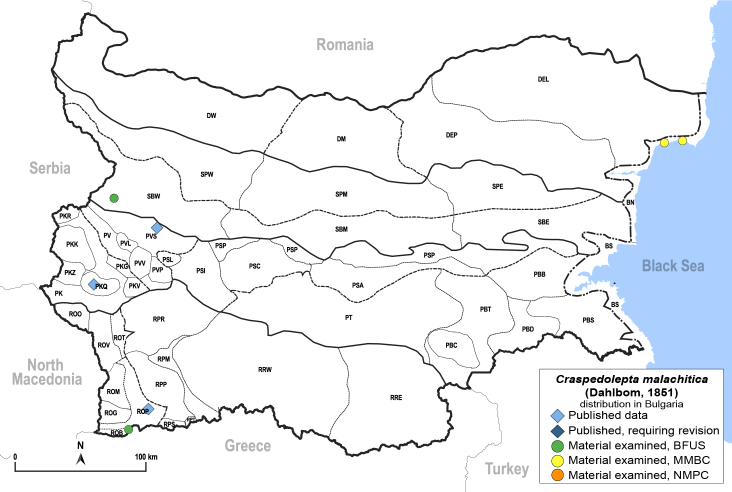
Distribution of *Craspedoleptamalachitica* in Bulgaria.

**Figure 22a.**
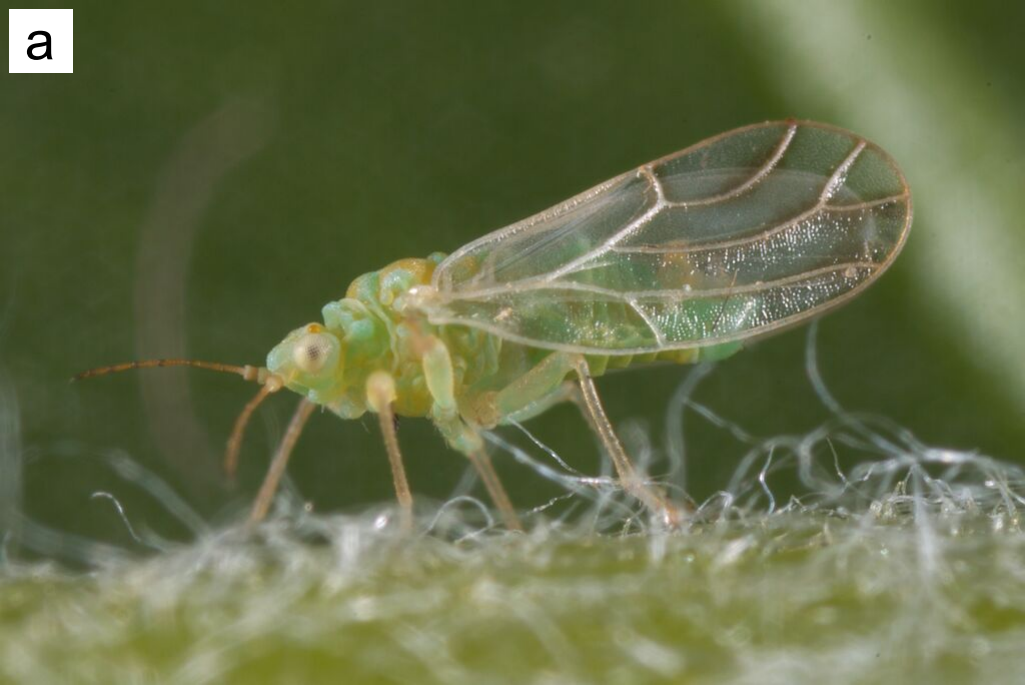
*Craspedoleptanervosa*, adult male;

**Figure 22b.**
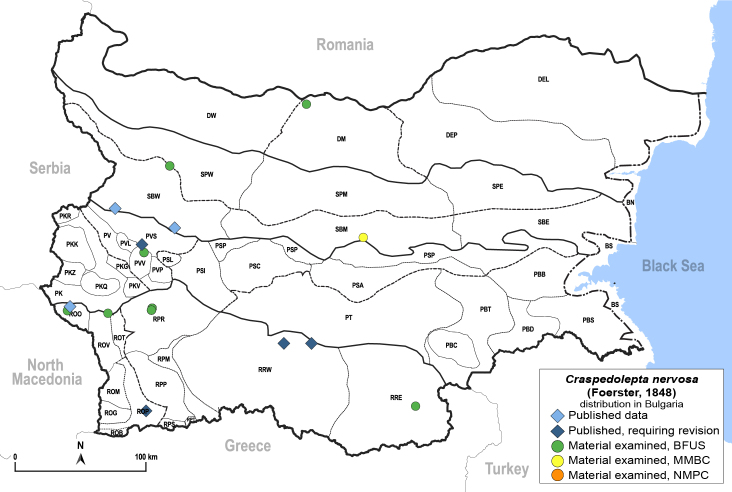
Distribution of *Craspedoleptanervosa* in Bulgaria.

**Figure 23a.**
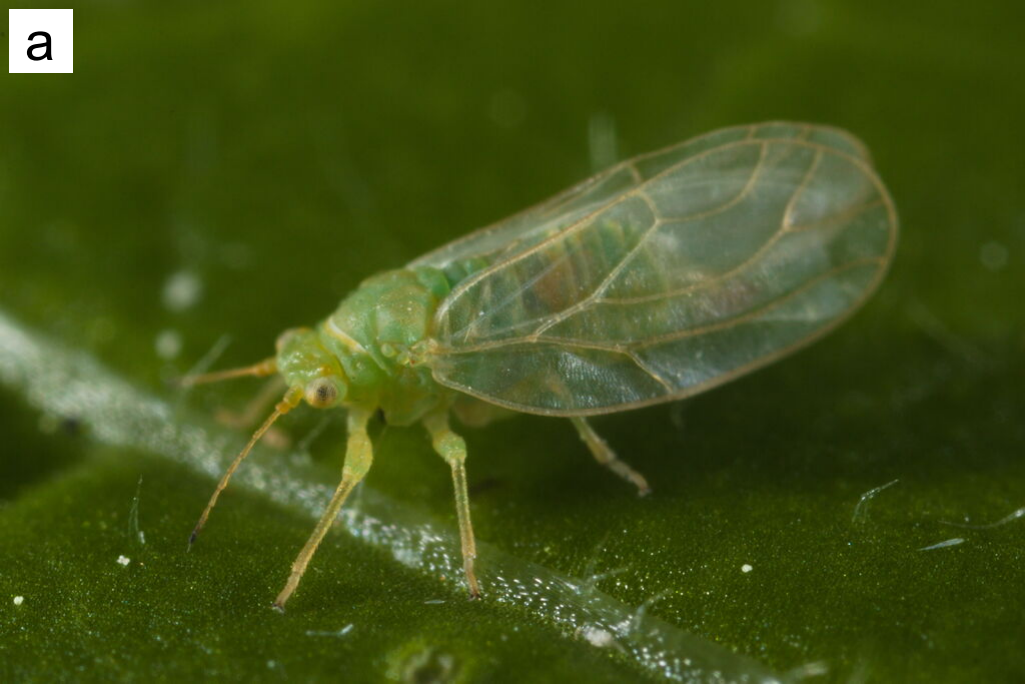
*Craspedoleptaomissa*, adult female;

**Figure 23b.**
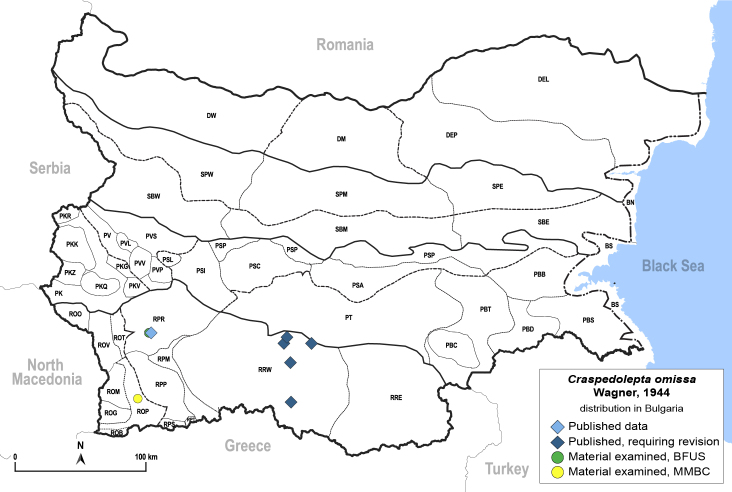
Distribution of *Craspedoleptaomissa* in Bulgaria.

**Figure 24a.**
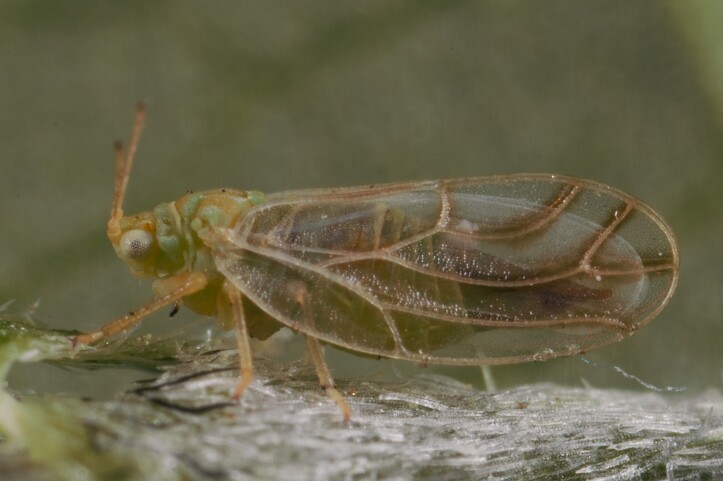
*Craspedoleptapontica*, adult female;

**Figure 24b.**
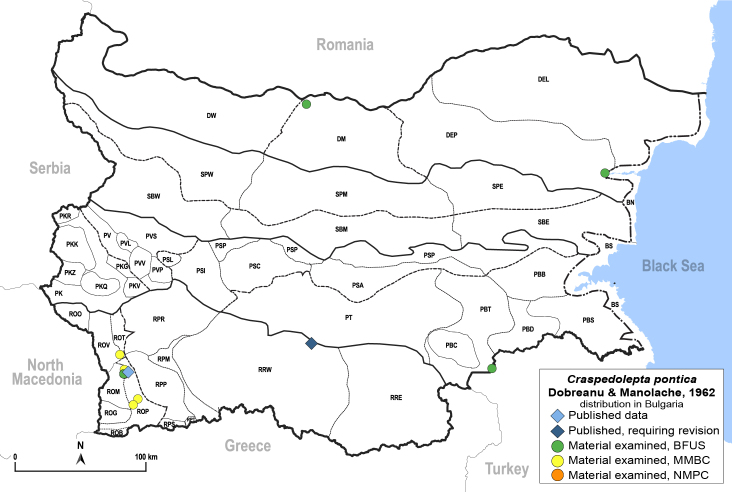
Distribution of *Craspedoleptapontica* in Bulgaria.

**Figure 26a.**
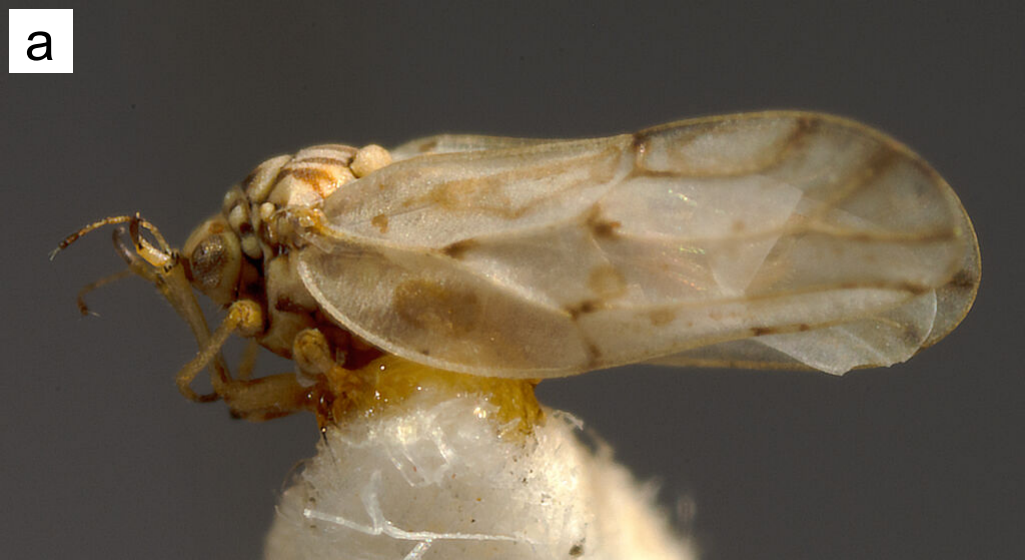
*Eumetoecuskochiae*, adult male;

**Figure 26b.**
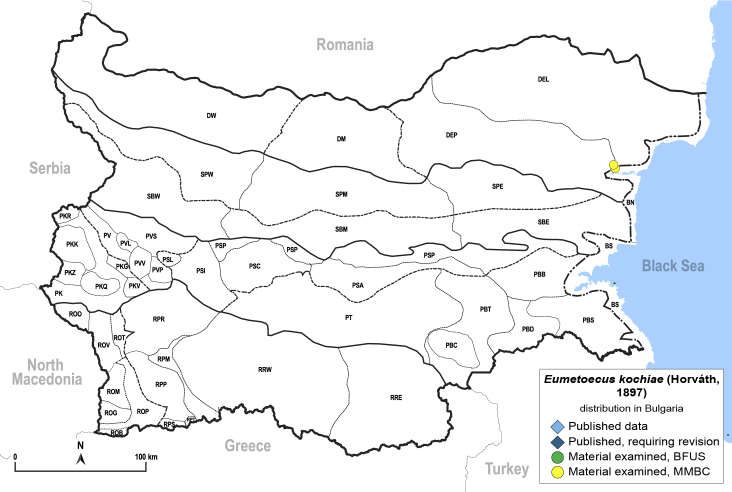
Distribution of *Eumetoecuskochiae* in Bulgaria.

**Figure 27a.**
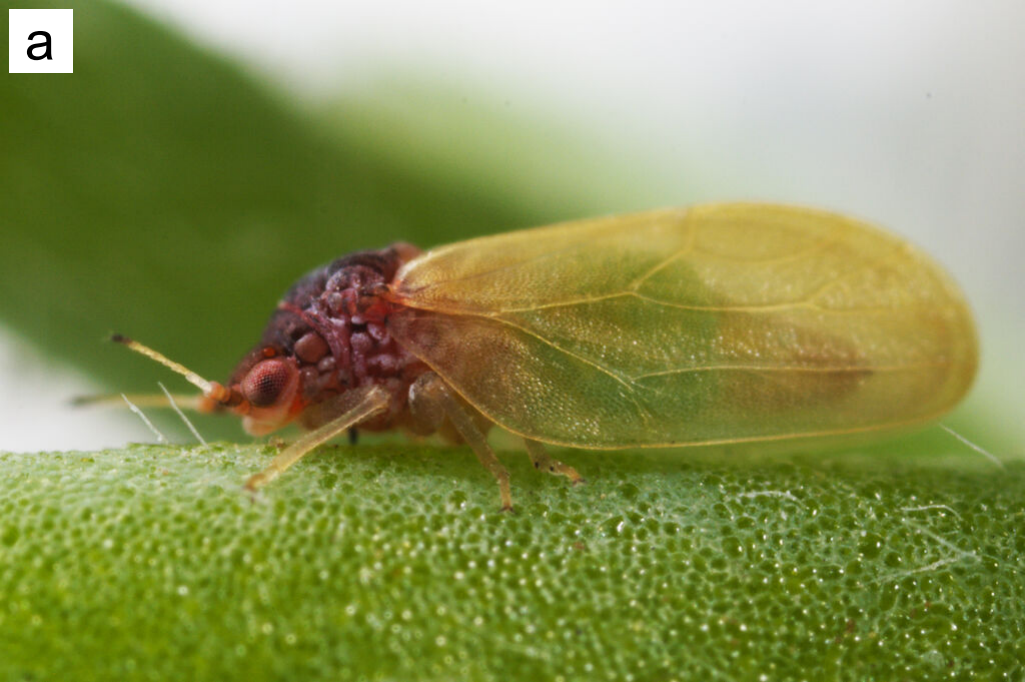
*Rhodochlanisbicolor*, adult female;

**Figure 27b.**
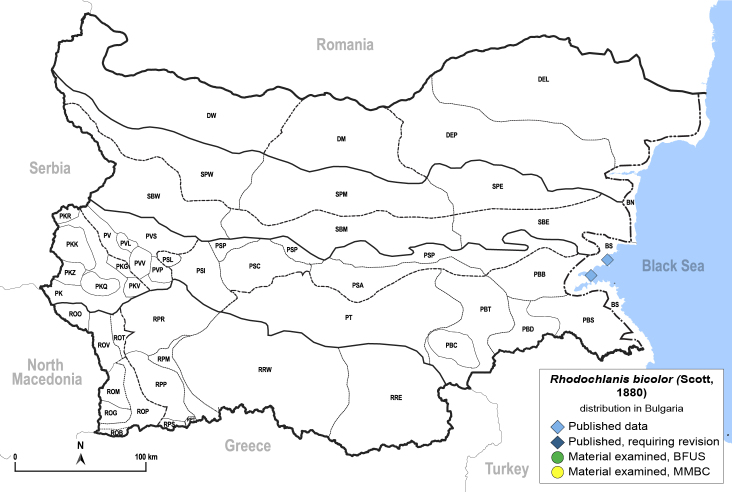
Distribution of *Rhodochlanisbicolor* in Bulgaria.

**Figure 28a.**
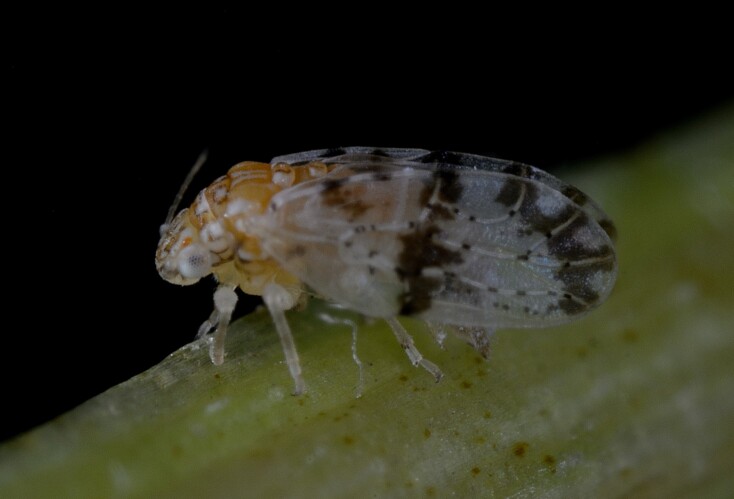
*Agonoscenapistaciae*, adult;

**Figure 28b.**
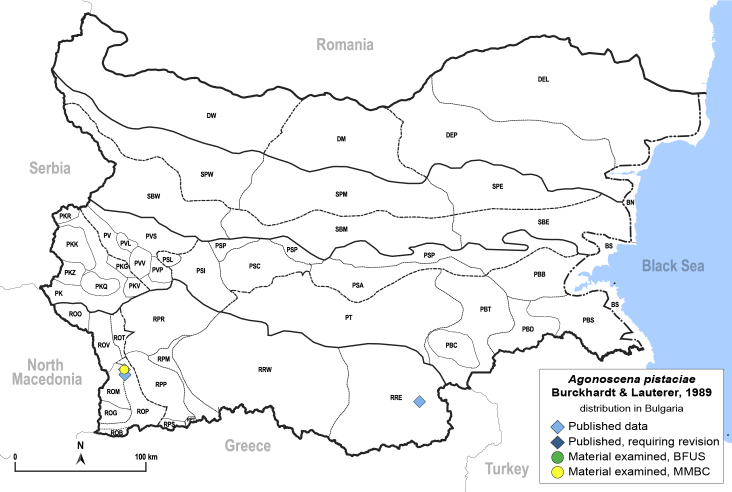
Distribution of *A.pistaciae* in Bulgaria.

**Figure 29a.**
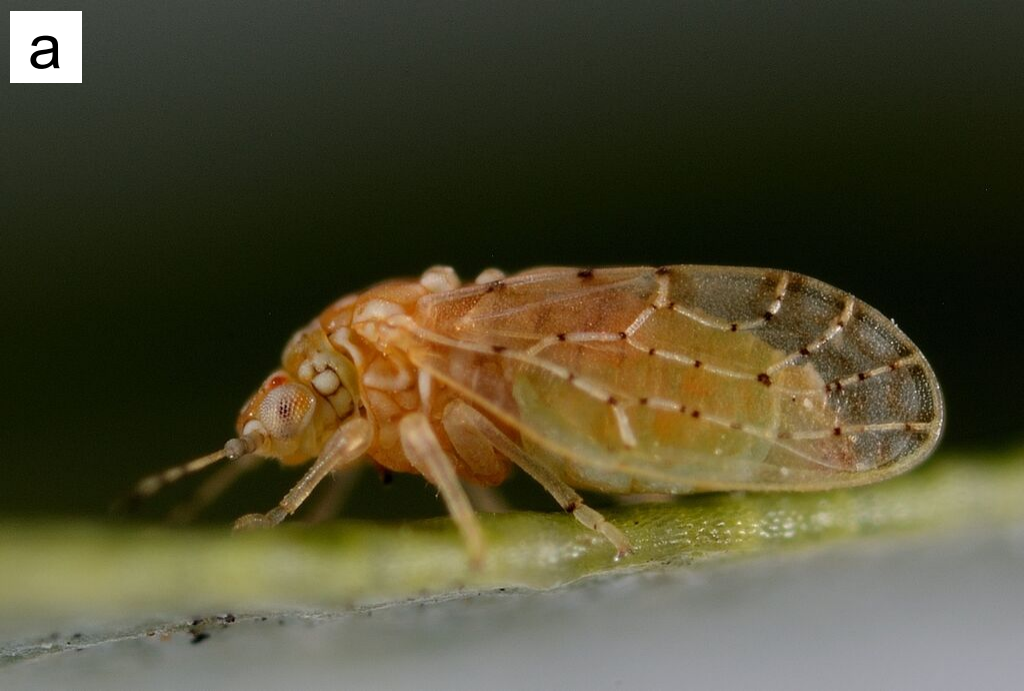
*Agonoscenatargionii*, adult female;

**Figure 29b.**
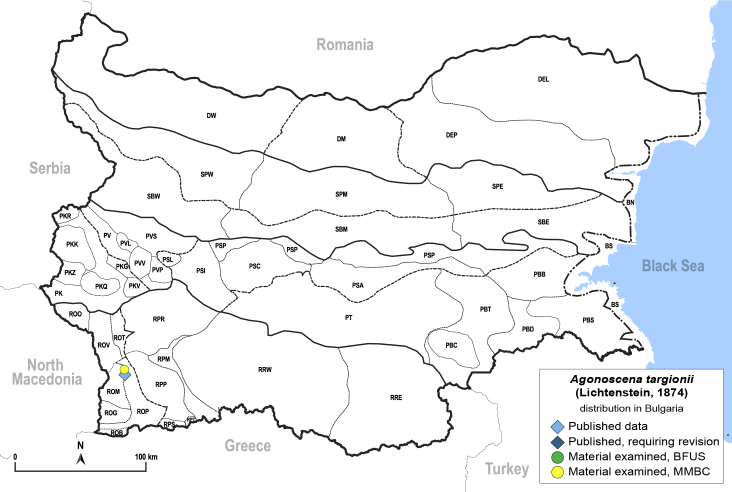
Distribution of *Agonoscenatargionii* in Bulgaria.

**Figure 30a.**
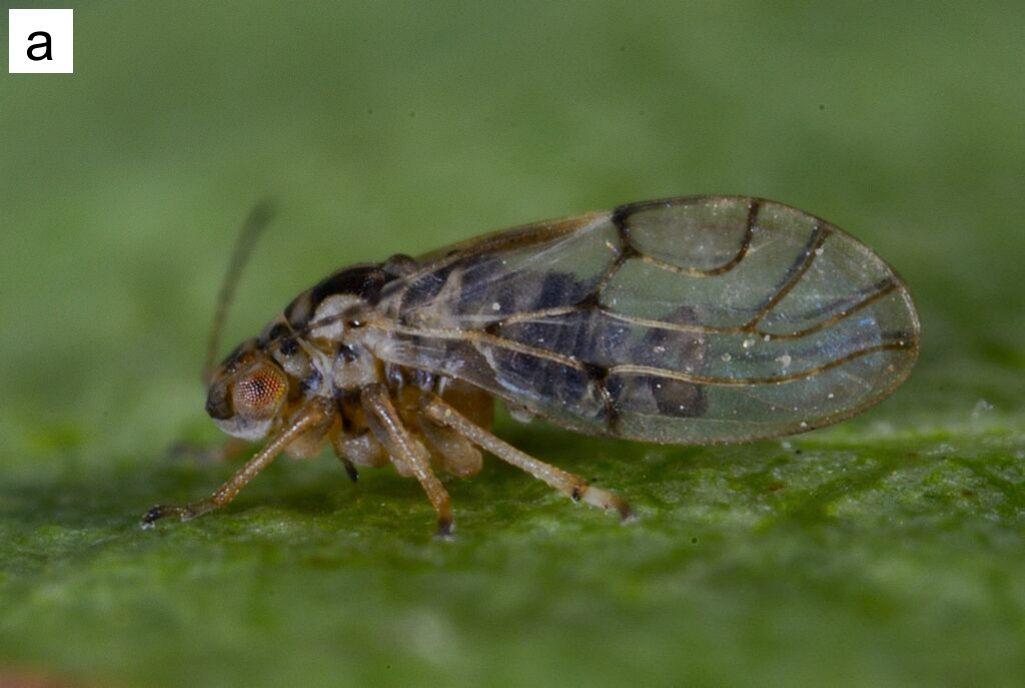
*Megagonoscenagallicola*, adult male;

**Figure 30b.**
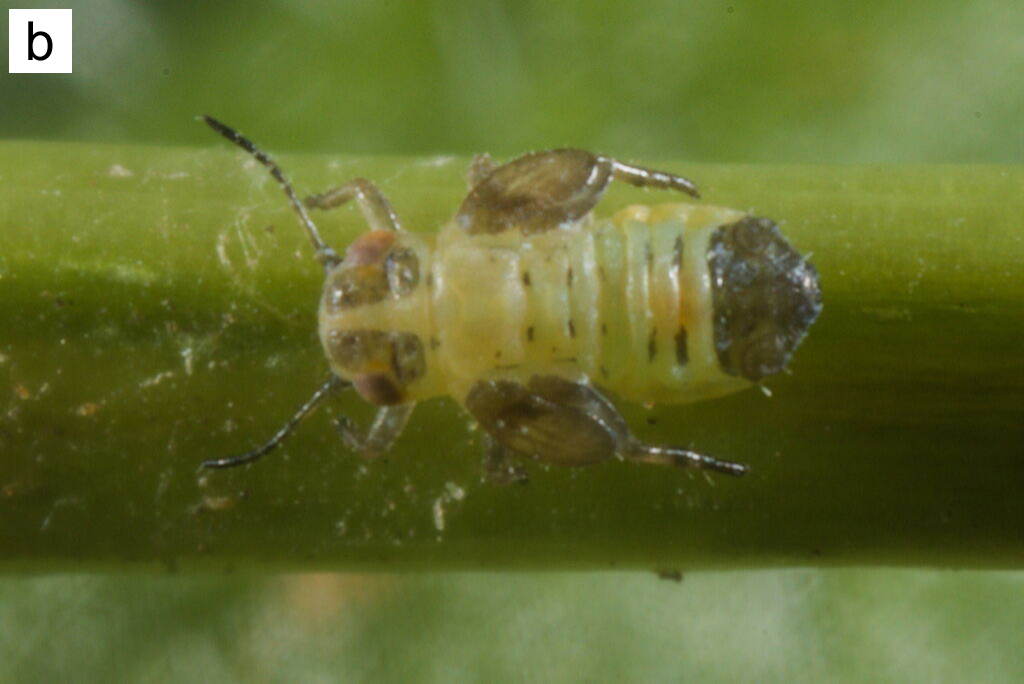
*Megagonoscenagallicola*, fifth instar immature;

**Figure 30c.**
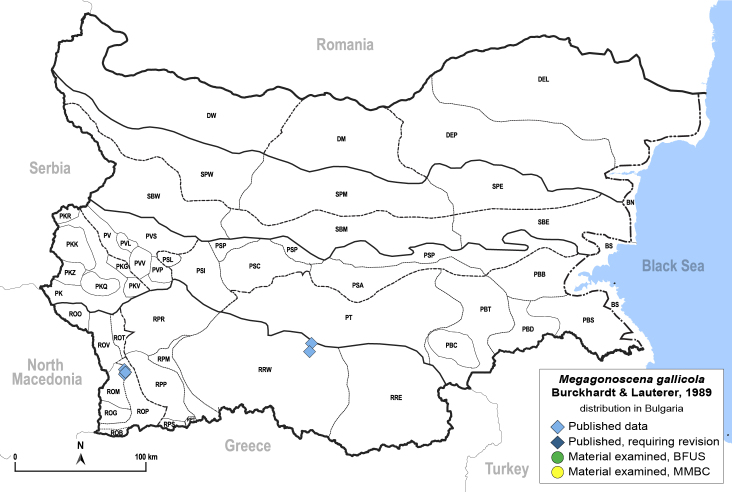
Distribution of *Megagonoscenagallicola* in Bulgaria.

**Figure 31a.**
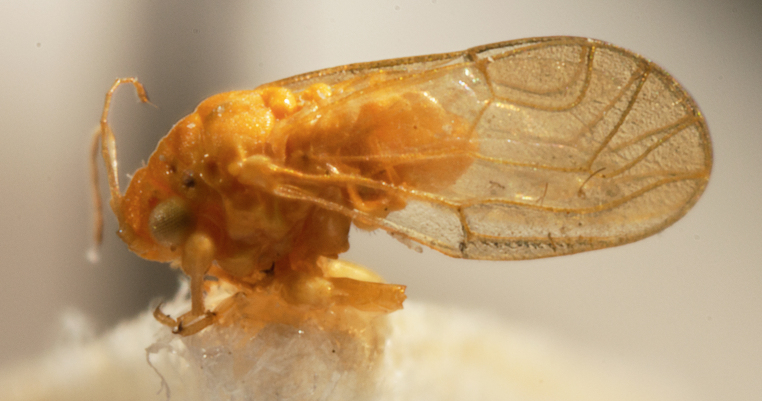
*Megagonoscenaviridis*, adult male;

**Figure 31b.**
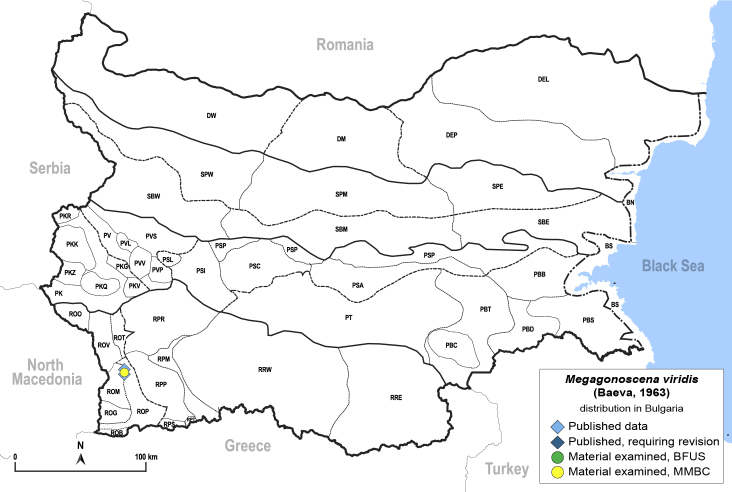
Distribution of *Megagonoscenaviridis* in Bulgaria.

**Figure 32a.**
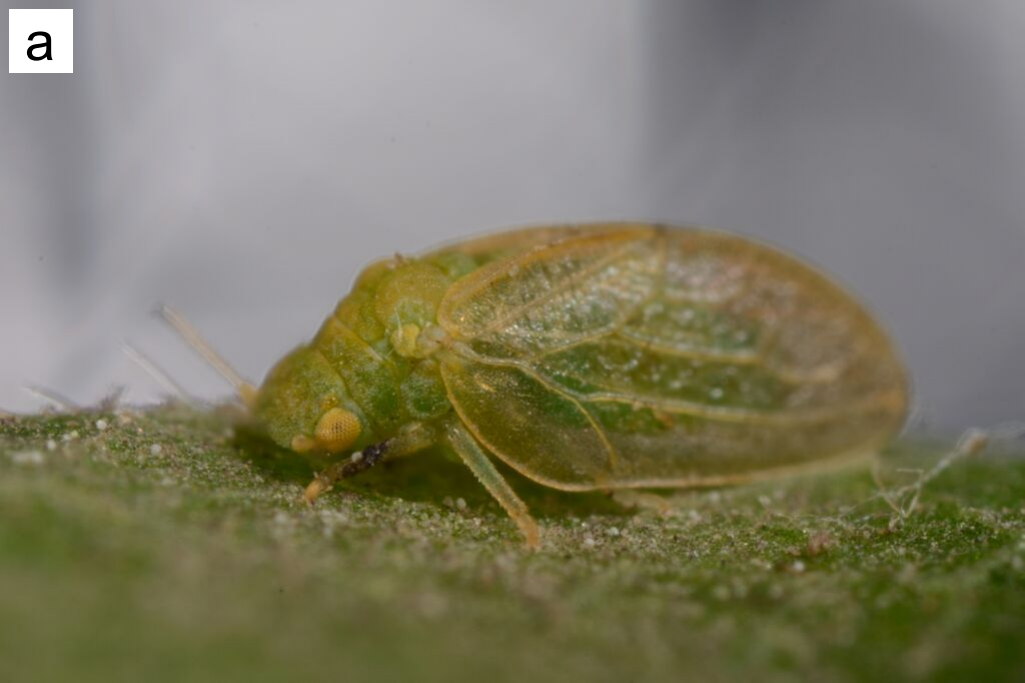
*Rhinocolaaceris*, adult male;

**Figure 32b.**
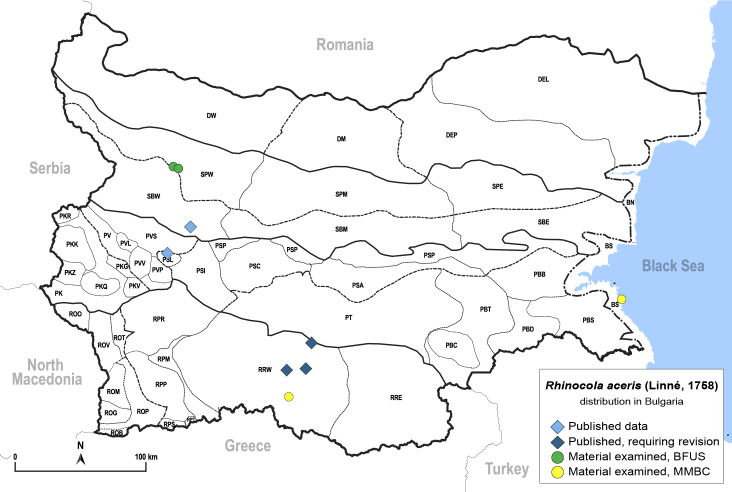
Distribution of *Rhinocolaaceris* in Bulgaria.

**Figure 33a.**
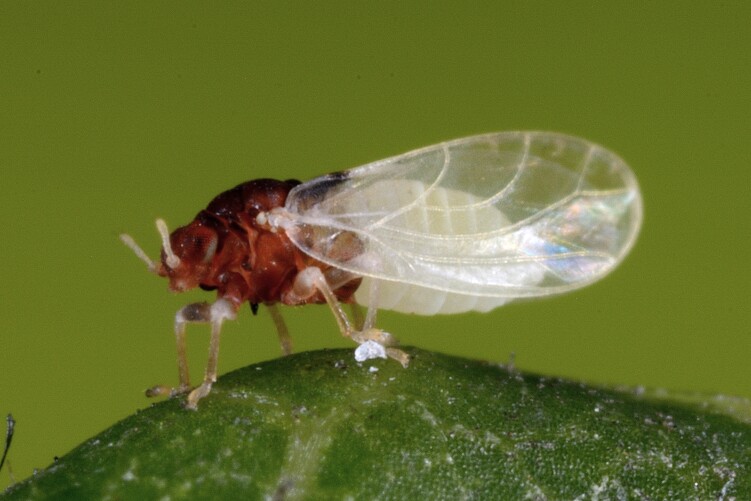
*Calophyarhois*, adult male;

**Figure 33b.**
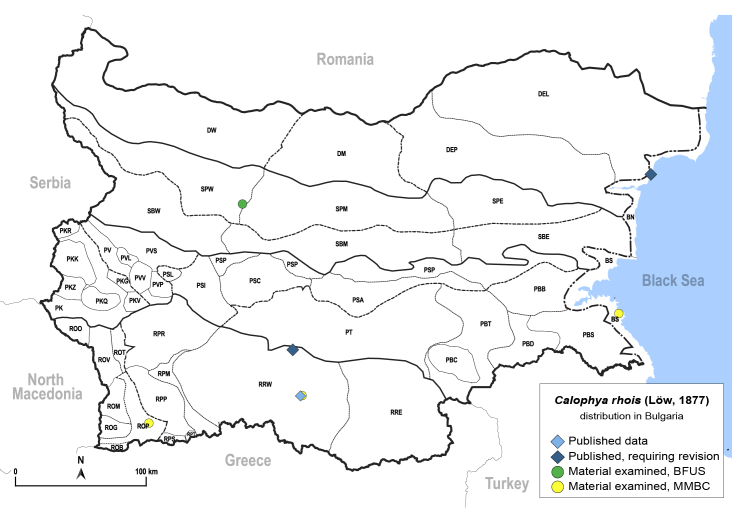
Distribution of *Calophyarhois* in Bulgaria.

**Figure 34a.**
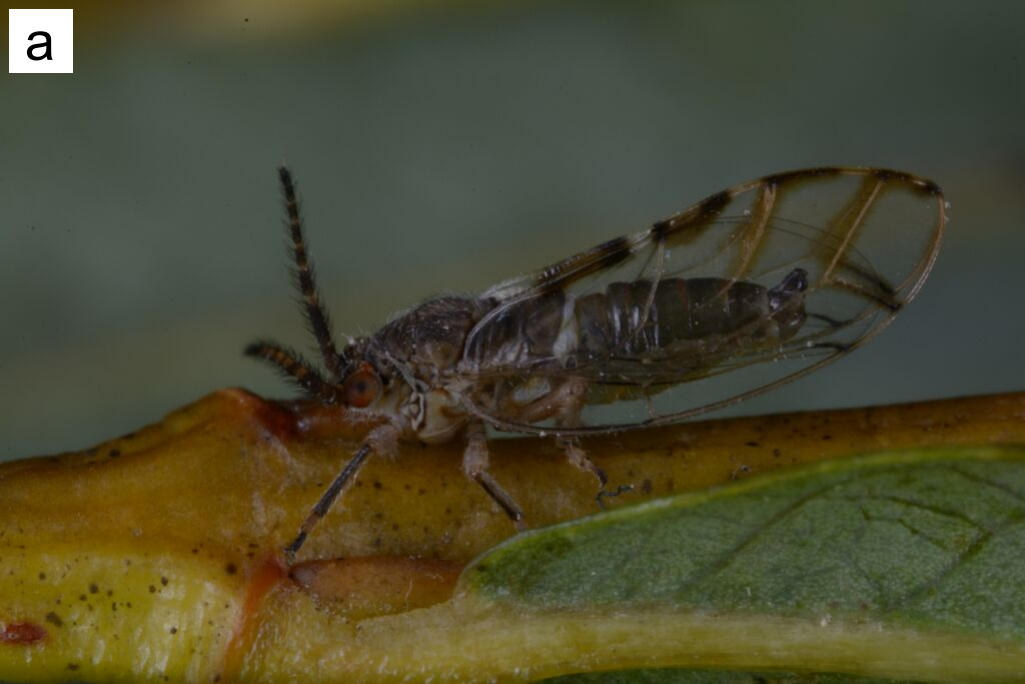
*Homotomaficus*, adult male;

**Figure 34b.**
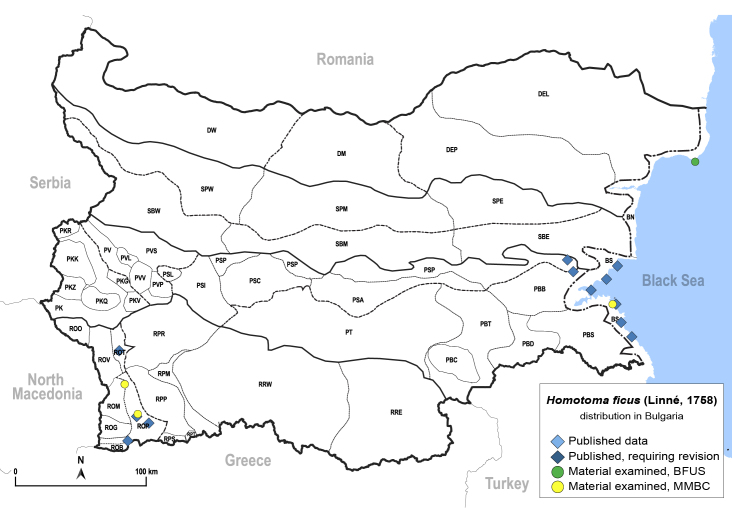
Distribution of *Homotomaficus* in Bulgaria.

**Figure 35a.**
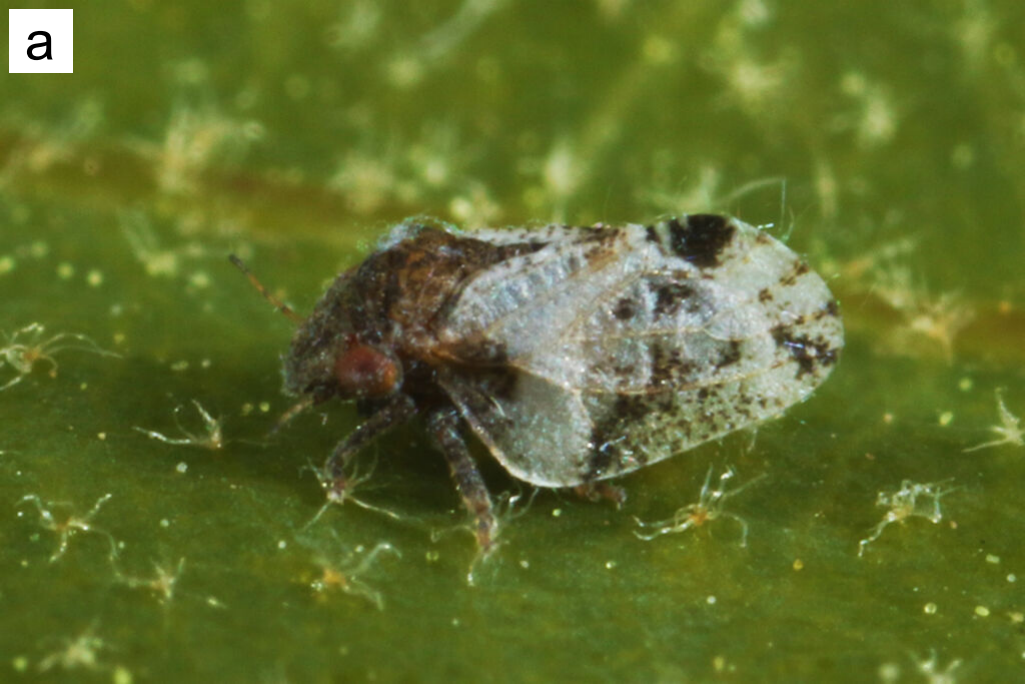
*Euphylluraphillyreae*, adult;

**Figure 35b.**
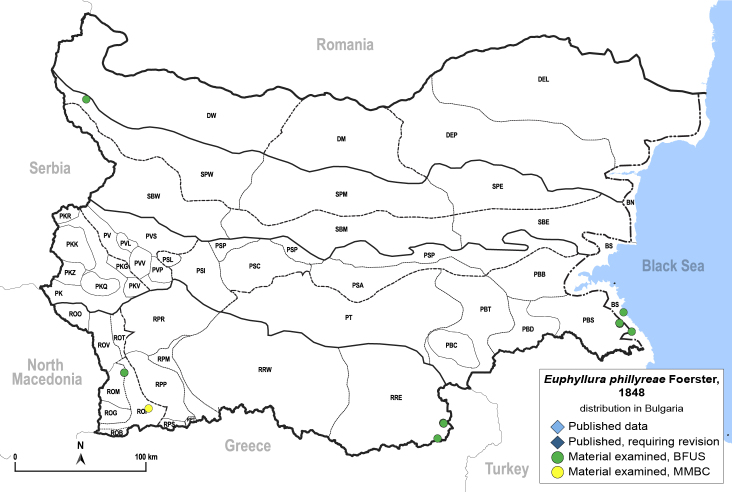
Distribution of *Euphylluraphillyreae* in Bulgaria.

**Figure 38a.**
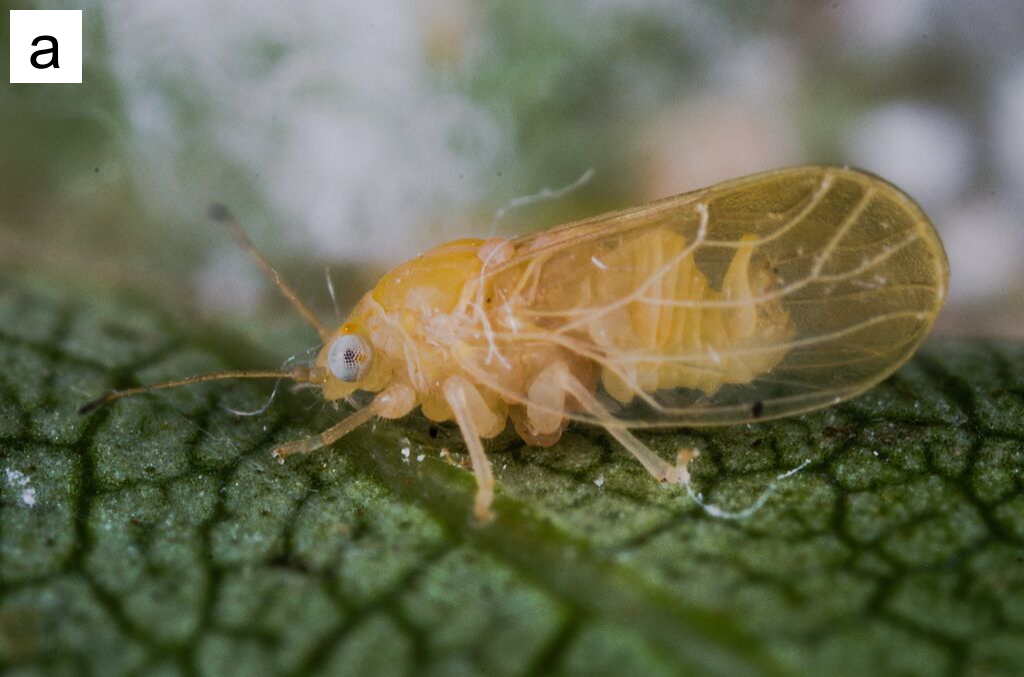
*Psyllopsisdobreanuae*, adult male;

**Figure 38b.**
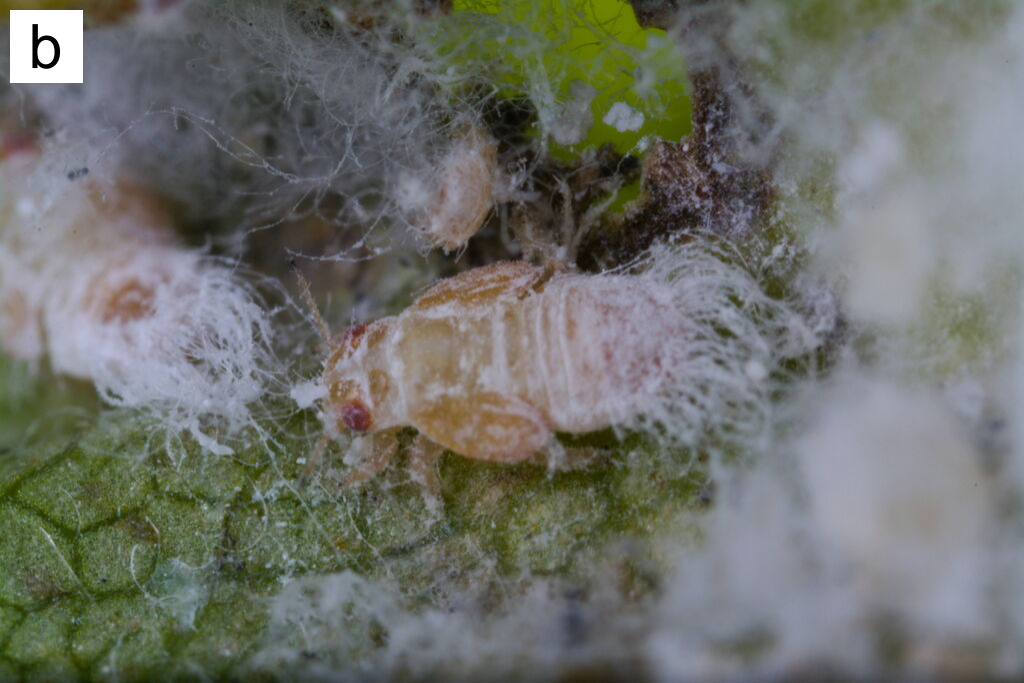
*Psyllopsisdobreanuae*, fifth instar immature;

**Figure 38c.**
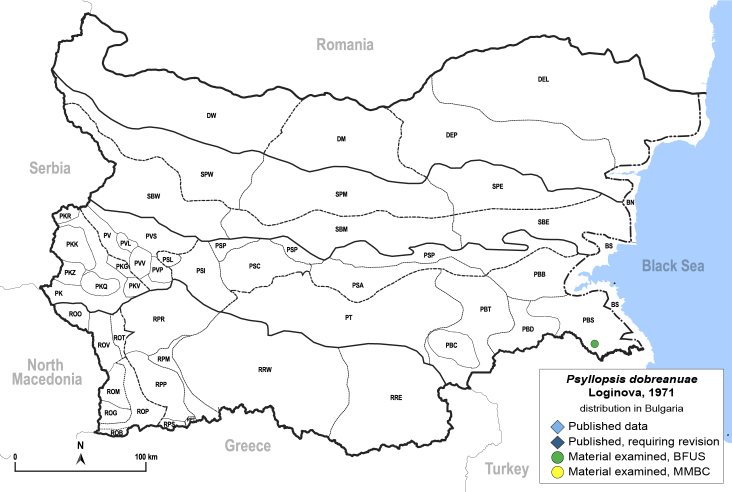
Distribution of *Psyllopsisdobreanuae* in Bulgaria.

**Figure 39a.**
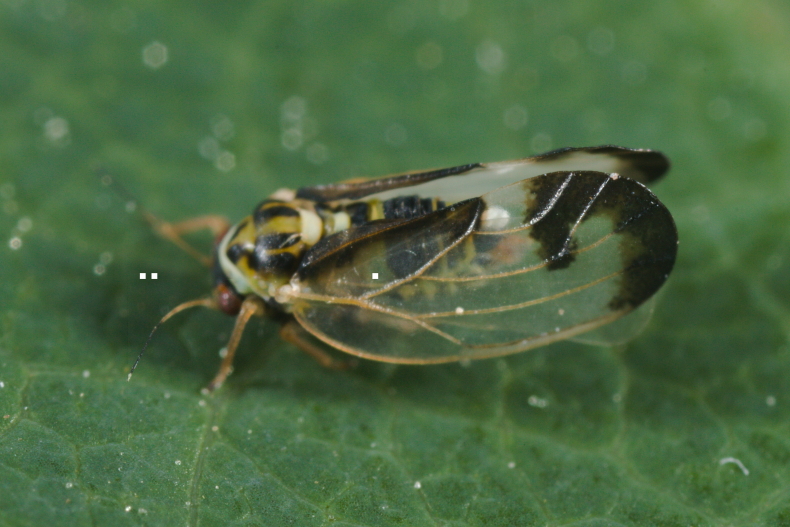
*Psyllopsisfraxini*, adult female;

**Figure 39b.**
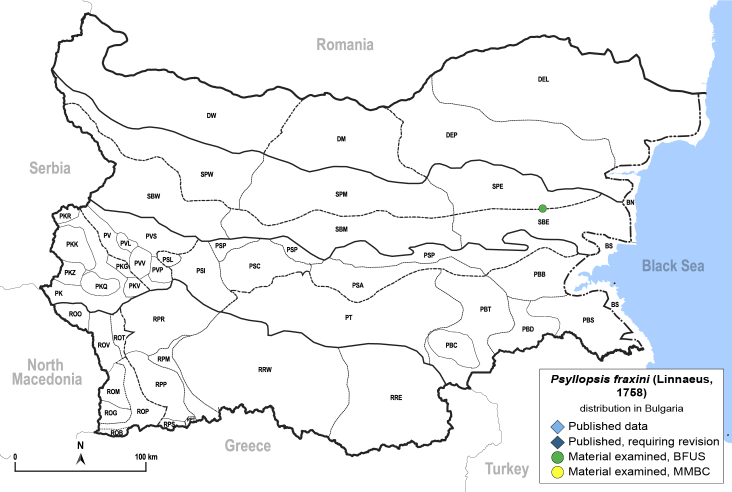
Distribution of *Psyllopsisfraxini* in Bulgaria.

**Figure 44a.**
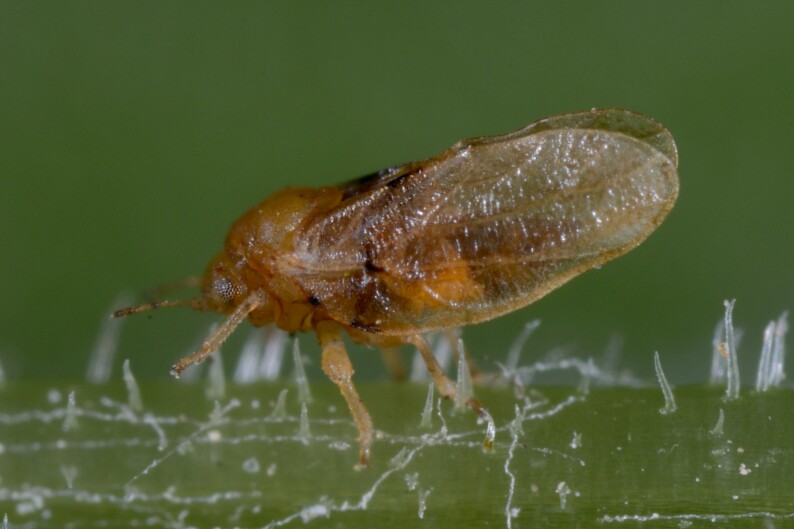
*Strophingiaericae*, adult female;

**Figure 44b.**
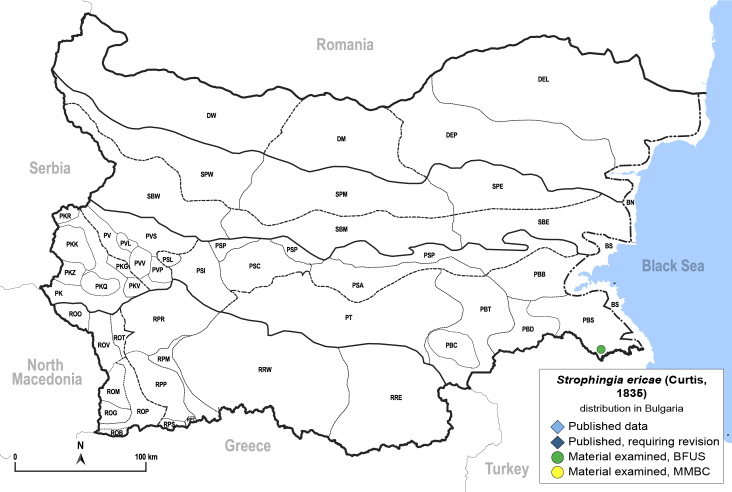
Distribution of *Strophingiaericae* in Bulgaria.

**Figure 46a.**
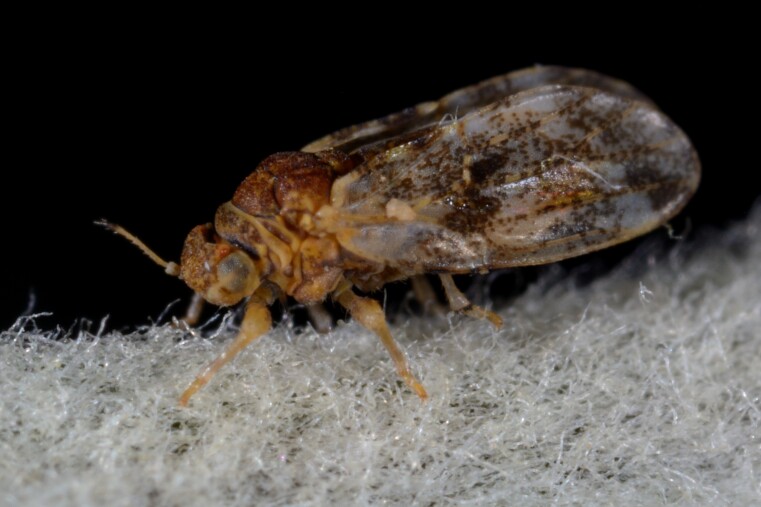
*Camaratoscenaspeciosa*, adult;

**Figure 46b.**
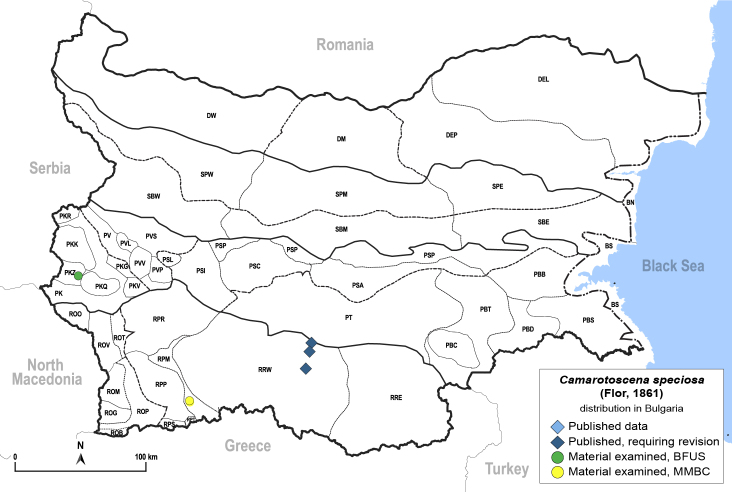
Distribution of *Camaratoscenaspeciosa* in Bulgaria.

**Figure 48a.**
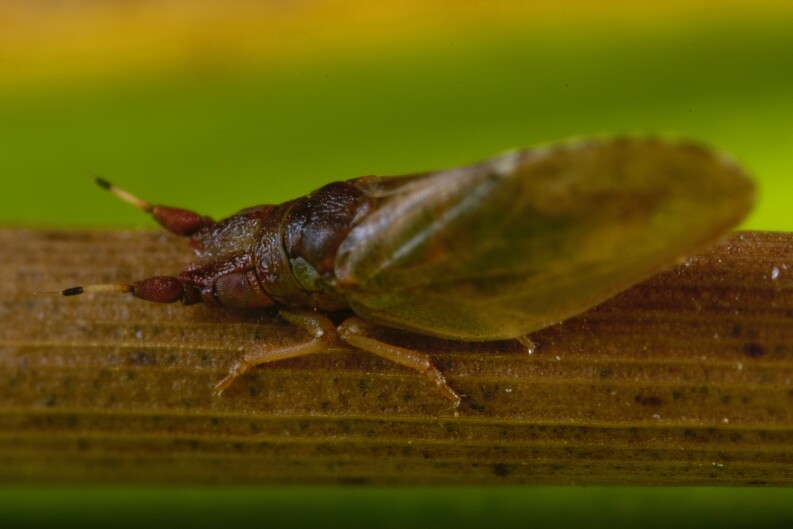
*Liviajunci*, adult female;

**Figure 48b.**
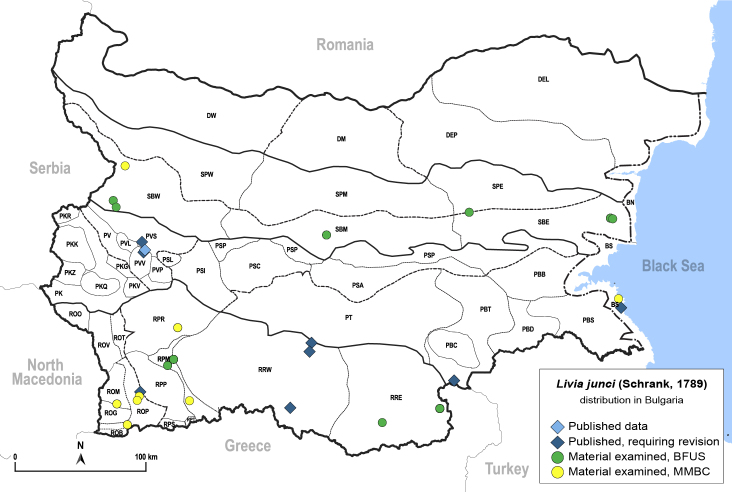
Distribution of *Liviajunci* in Bulgaria.

**Figure 50a.**
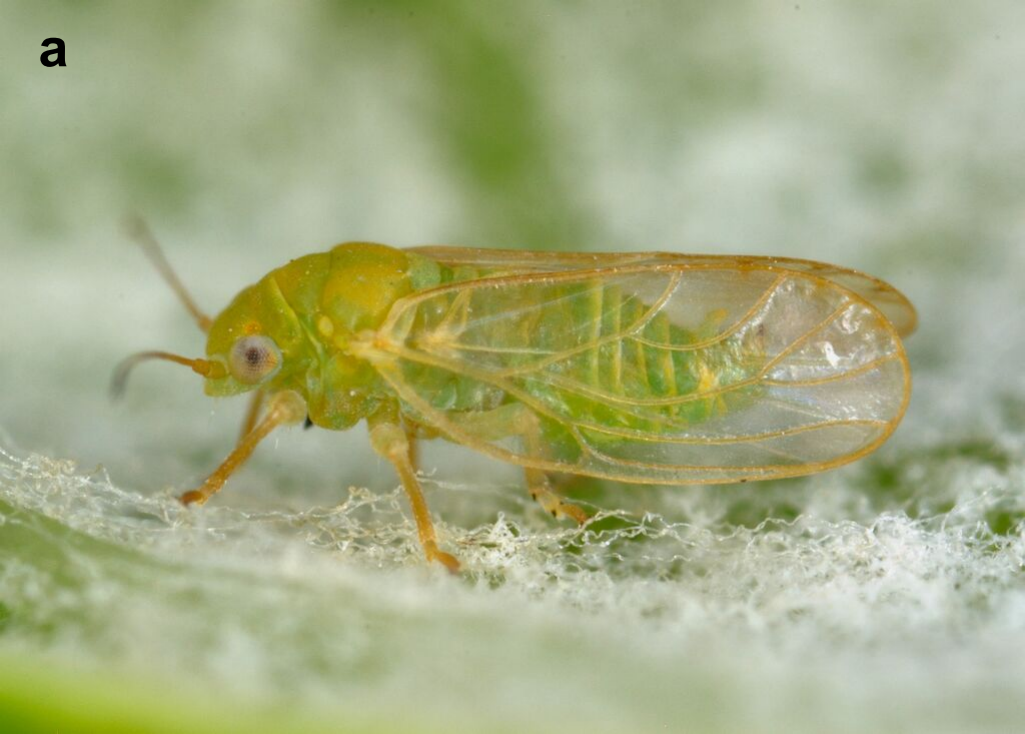
*Acizziajamatonica*, adult male;

**Figure 50b.**
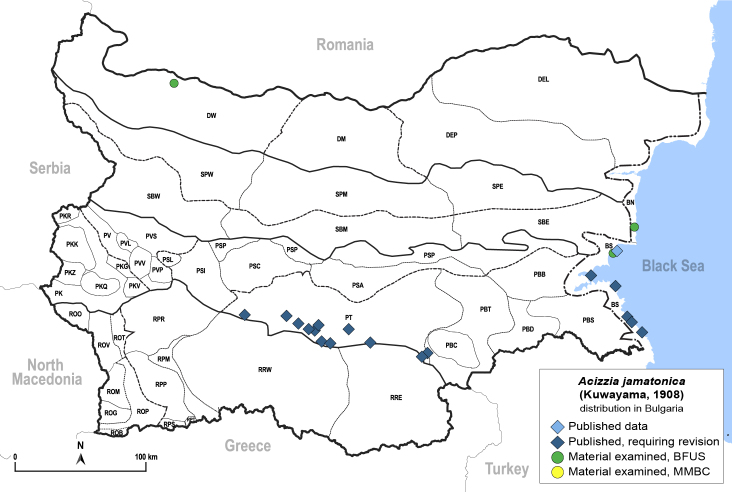
Distribution of *Acizziajamatonica* in Bulgaria.

**Figure 51a.**
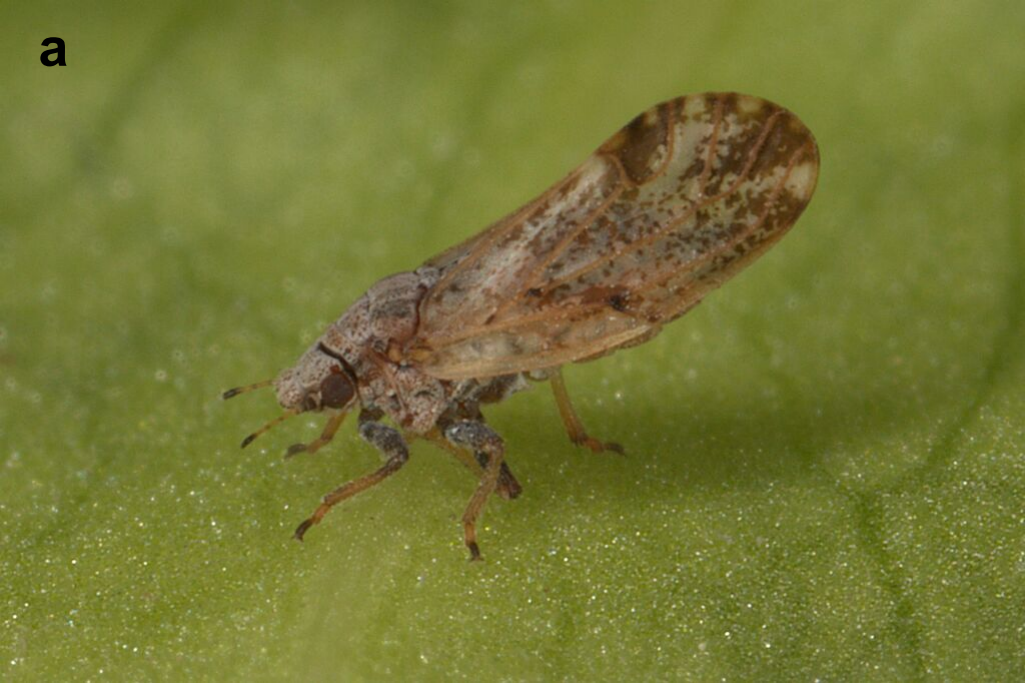
*Diaphorinalycii*, adult female;

**Figure 51b.**
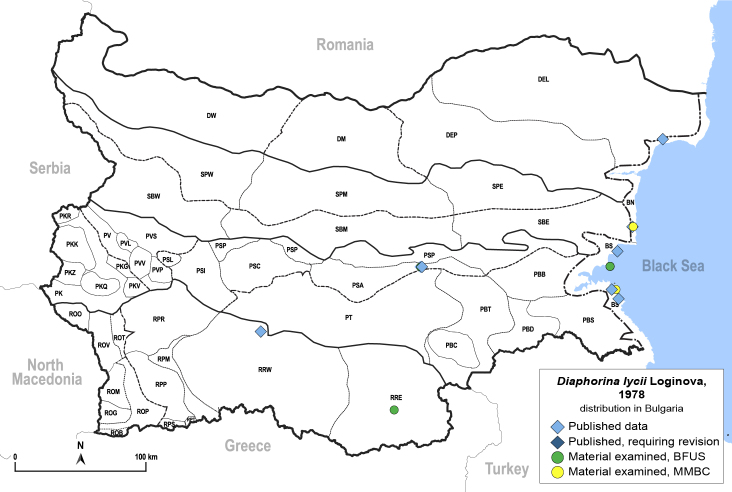
Distribution of *Diaphorinalycii* in Bulgaria.

**Figure 53a.**
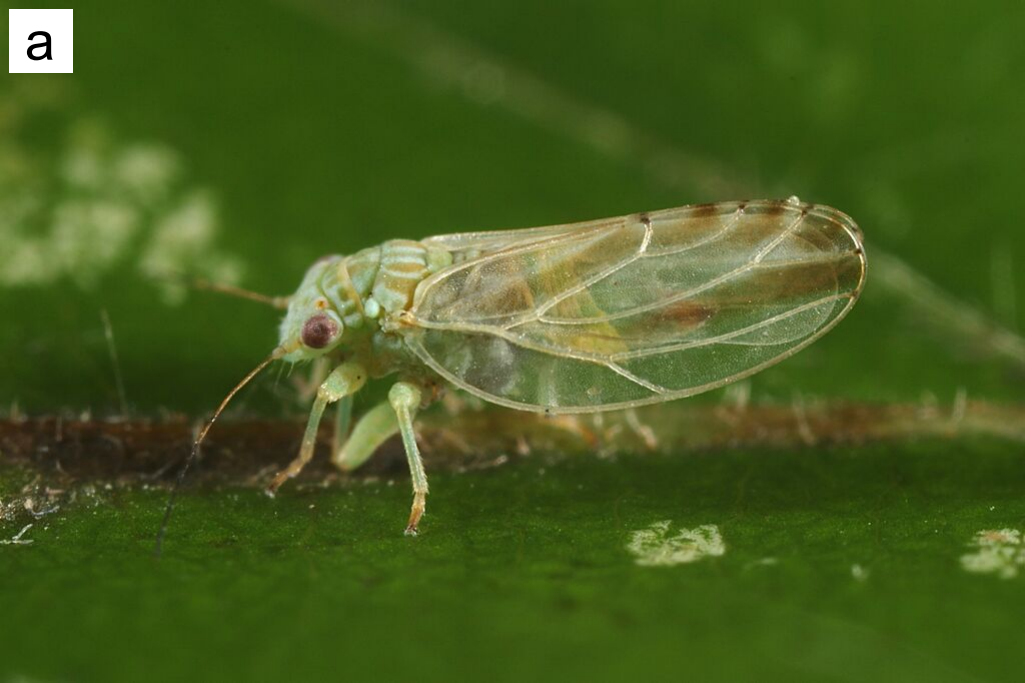
*Arytainamaculata*, adult female;

**Figure 53b.**
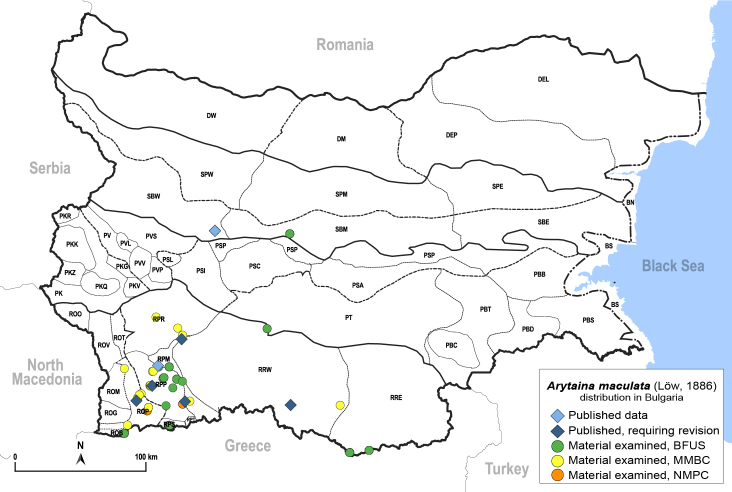
Distribution of *Arytainamaculata* in Bulgaria.

**Figure 54a.**
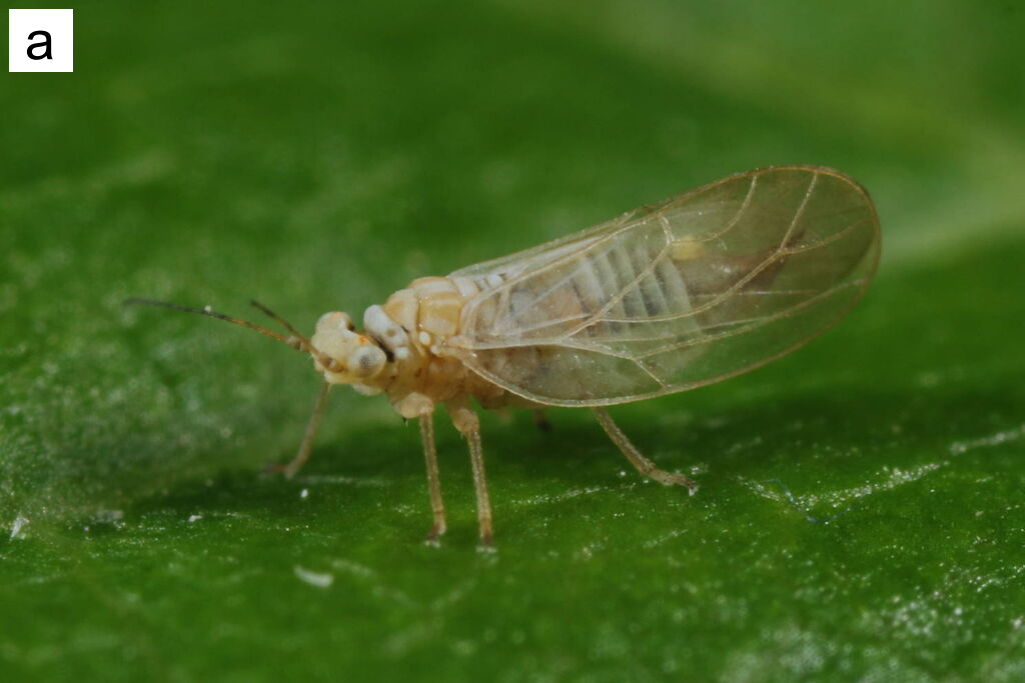
*Arytainillaspartiicola*, adult female;

**Figure 54b.**
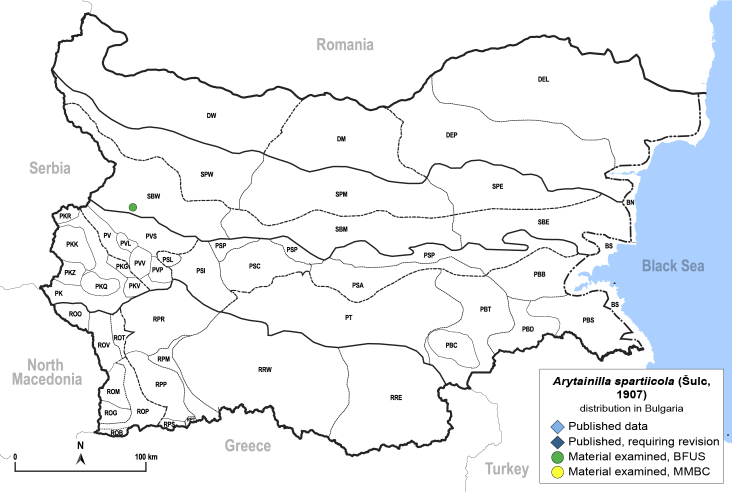
Distribution of *Arytainillaspartiicola* in Bulgaria.

**Figure 55a.**
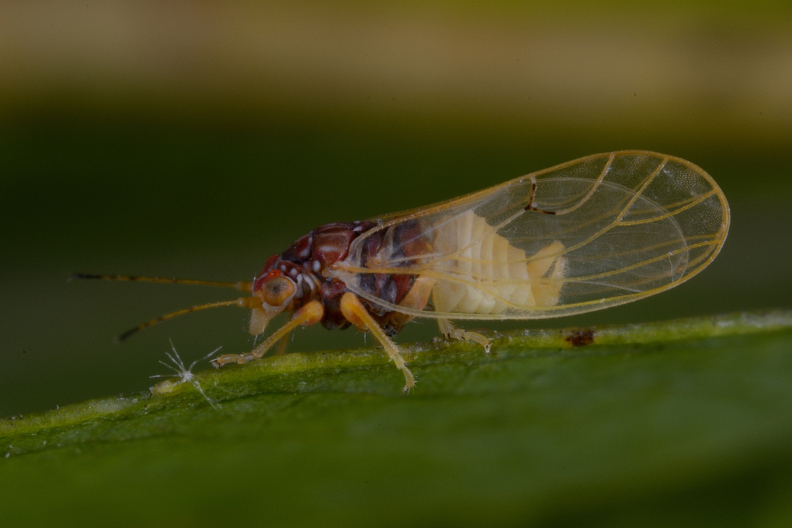
*Cacopsyllaabdominalis*, adult male;

**Figure 55b.**
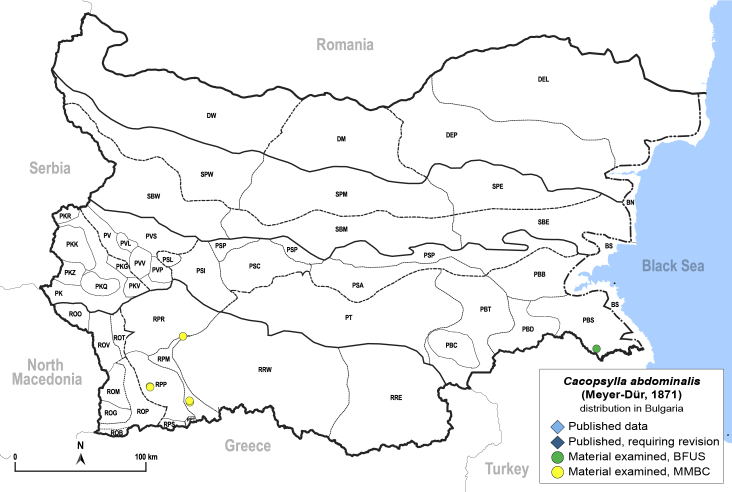
Distribution of *Cacopsyllaabdominalis* in Bulgaria.

**Figure 69a.**
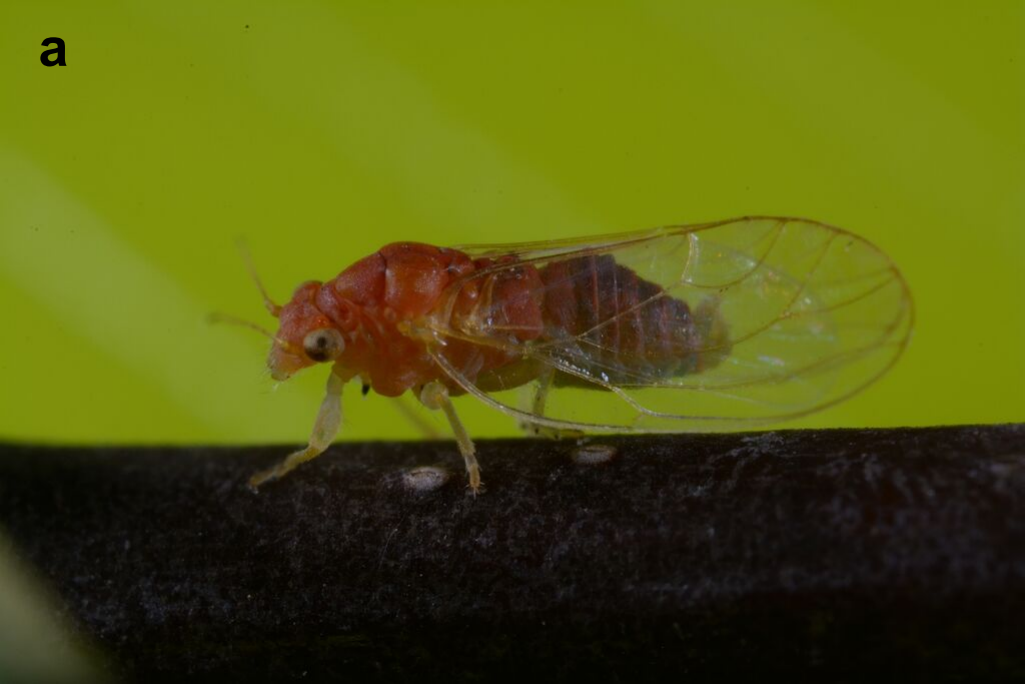
*Cacopsyllaperegrina*, adult male;

**Figure 69b.**
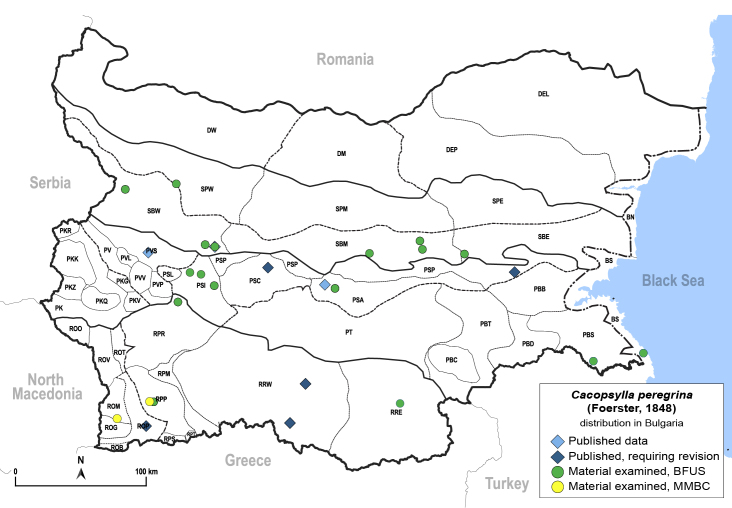
Distribution of *Cacopsyllaperegrina* in Bulgaria.

**Figure 71a.**
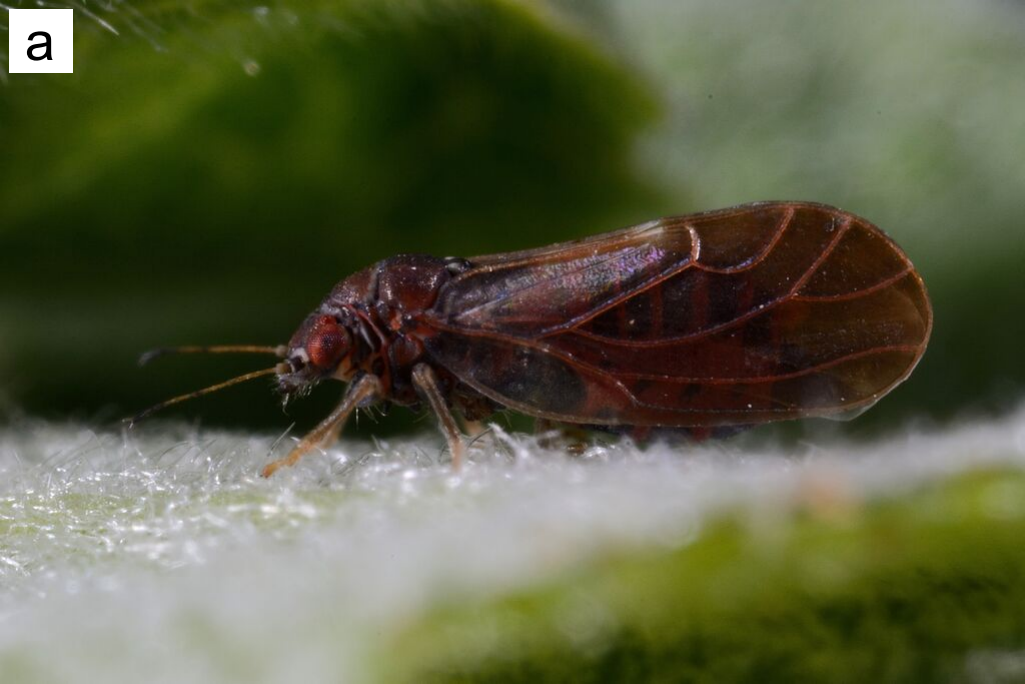
*Cacopsyllapruni*, adult female;

**Figure 71b.**
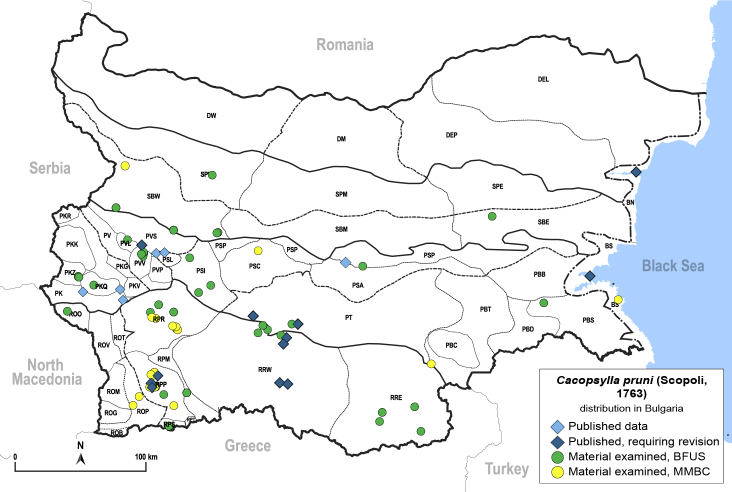
Distribution of *Cacopsyllapruni* in Bulgaria.

**Figure 72a.**
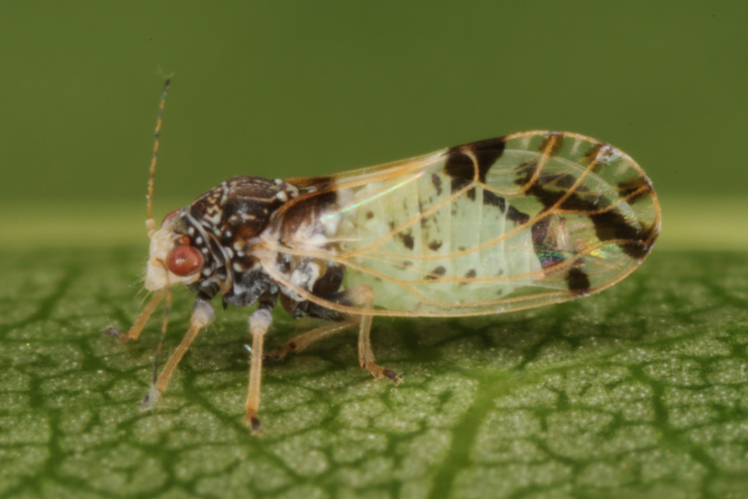
*Cacopsyllapulchella*, adult male;

**Figure 72b.**
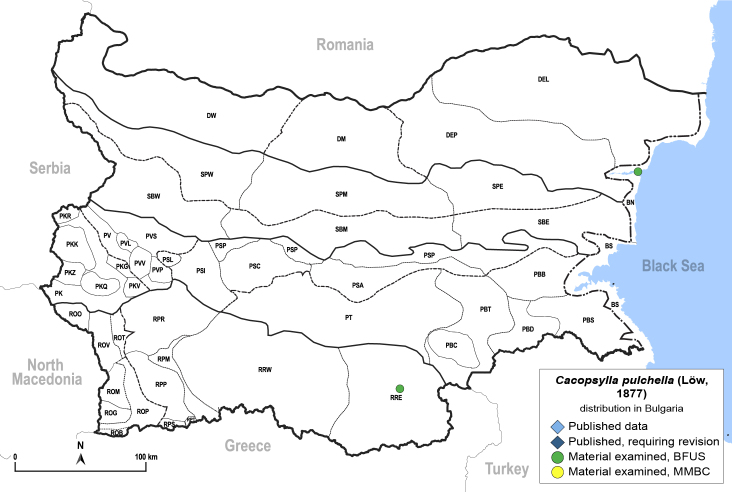
Distribution of *Cacopsyllapulchella* in Bulgaria.

**Figure 77a.**
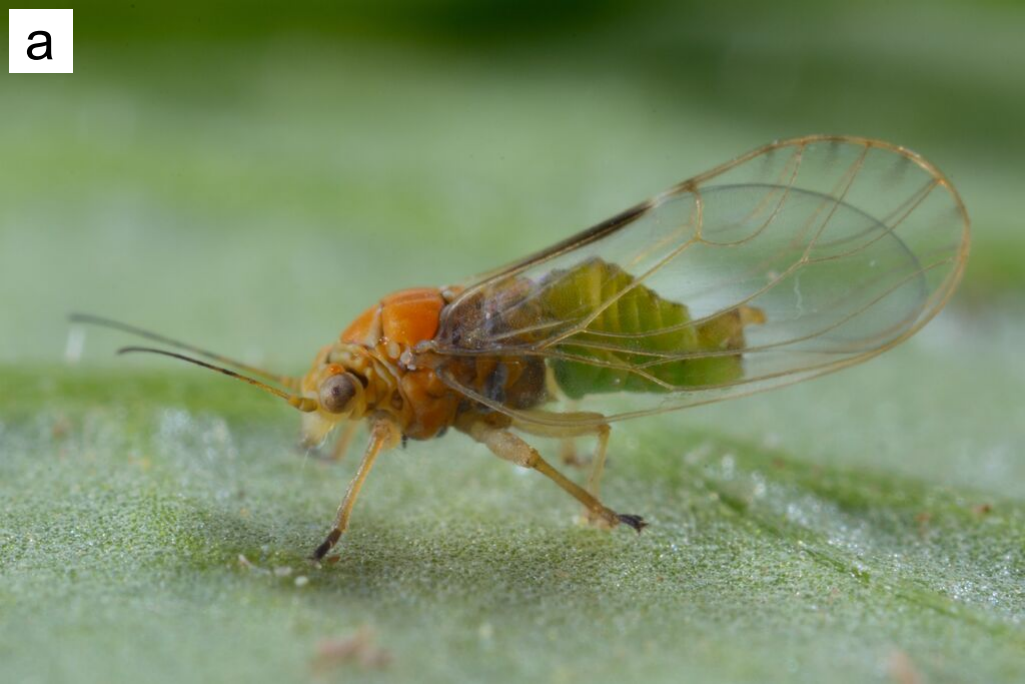
*Cacopsyllarhamnicola*, adult male

**Figure 77b.**
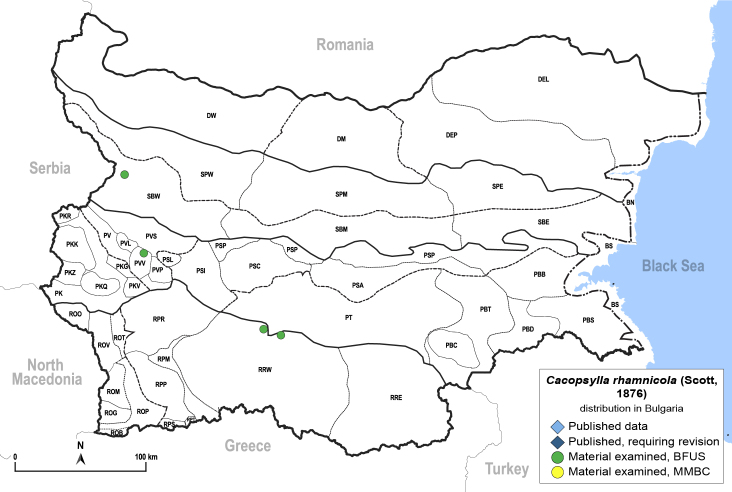
Distribution of *Cacopsyllarhamnicola* in Bulgaria.

**Figure 81a.**
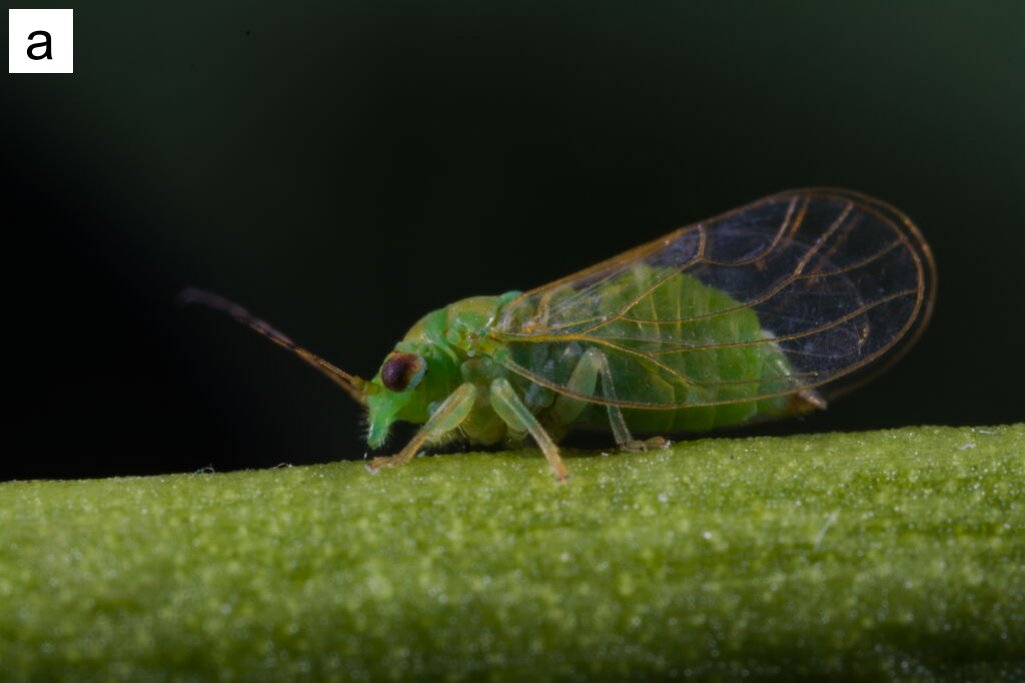
*Cacopsyllavisci*, adult female;

**Figure 81b.**
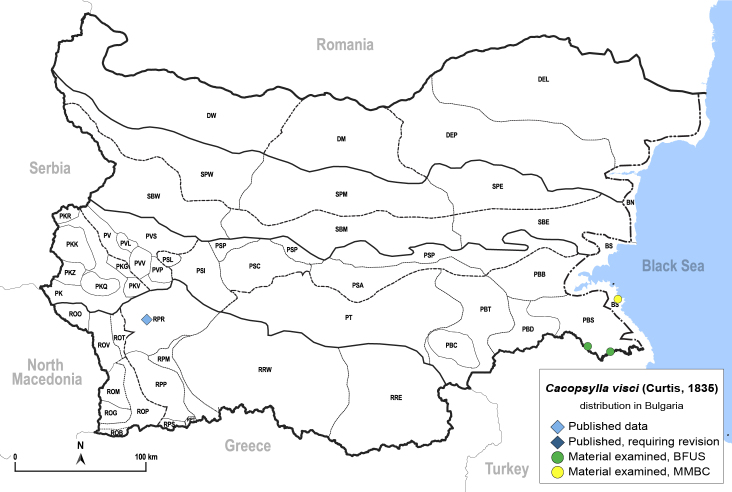
Distribution of *Cacopsyllavisci* in Bulgaria.

**Figure 82a.**
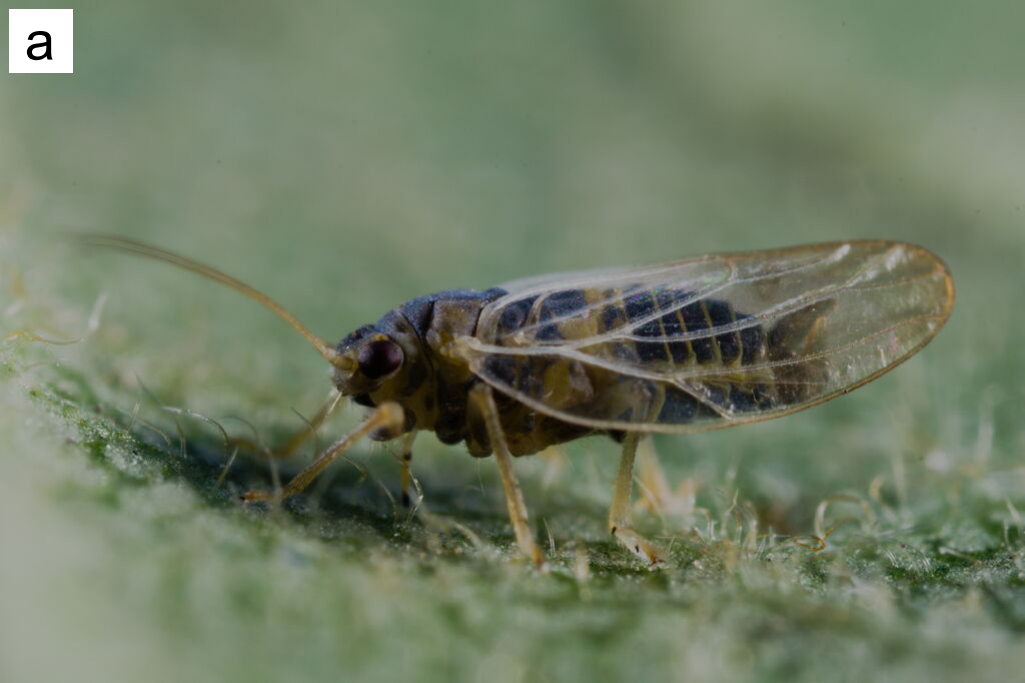
*Livillacognata*, adult male (dark specimen collected in September);

**Figure 82b.**
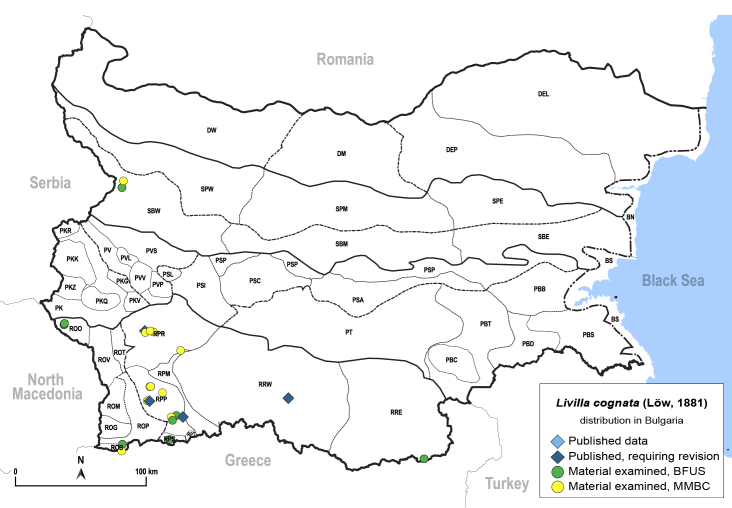
Distribution of *Livillacognata* in Bulgaria.

**Figure 83a.**
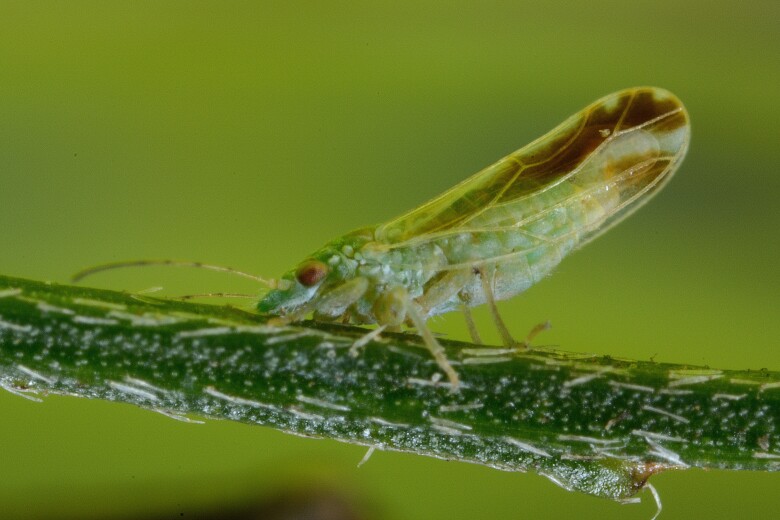
*Livillahorvathi*, adult male;

**Figure 83b.**
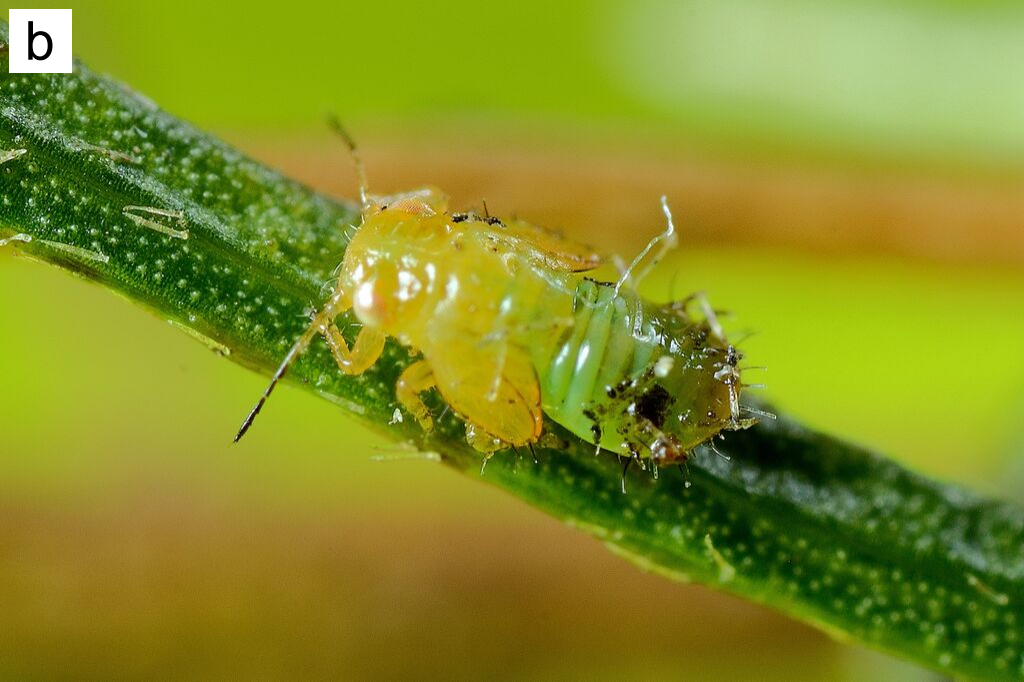
*Livillahorvathi*, immature;

**Figure 83c.**
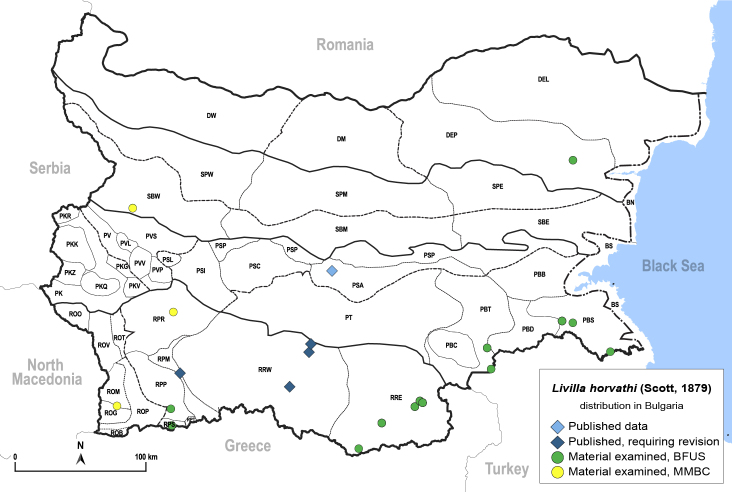
Distribution of *Livillahorvathi* in Bulgaria.

**Figure 84a.**
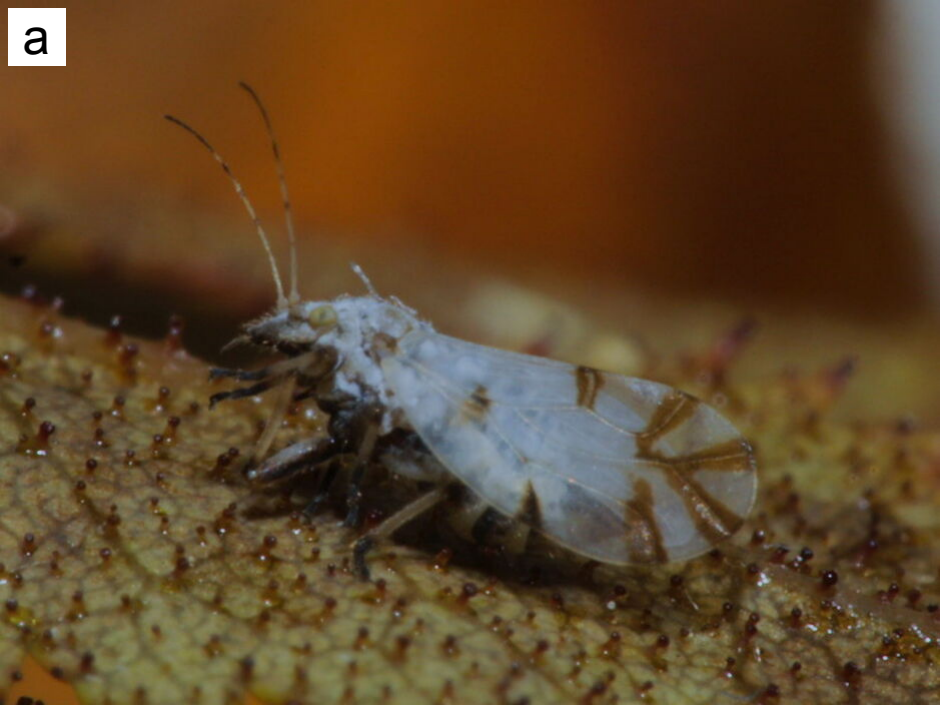
*Livillaradiata*, adult female;

**Figure 84b.**
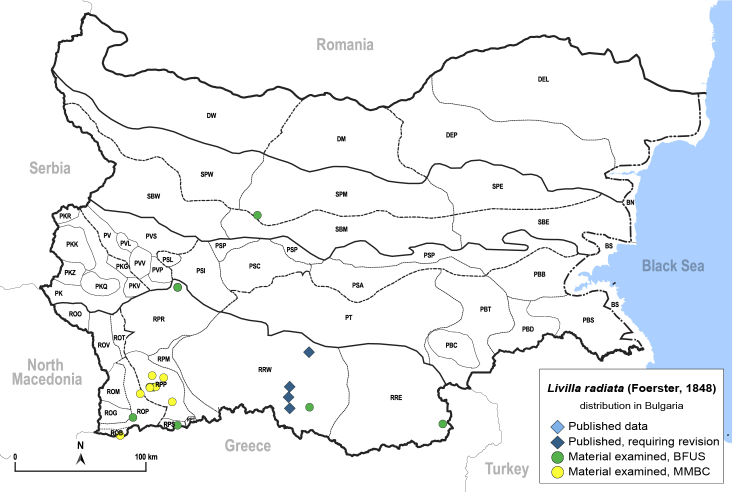
Distribution of *Livillaradiata* in Bulgaria.

**Figure 85a.**
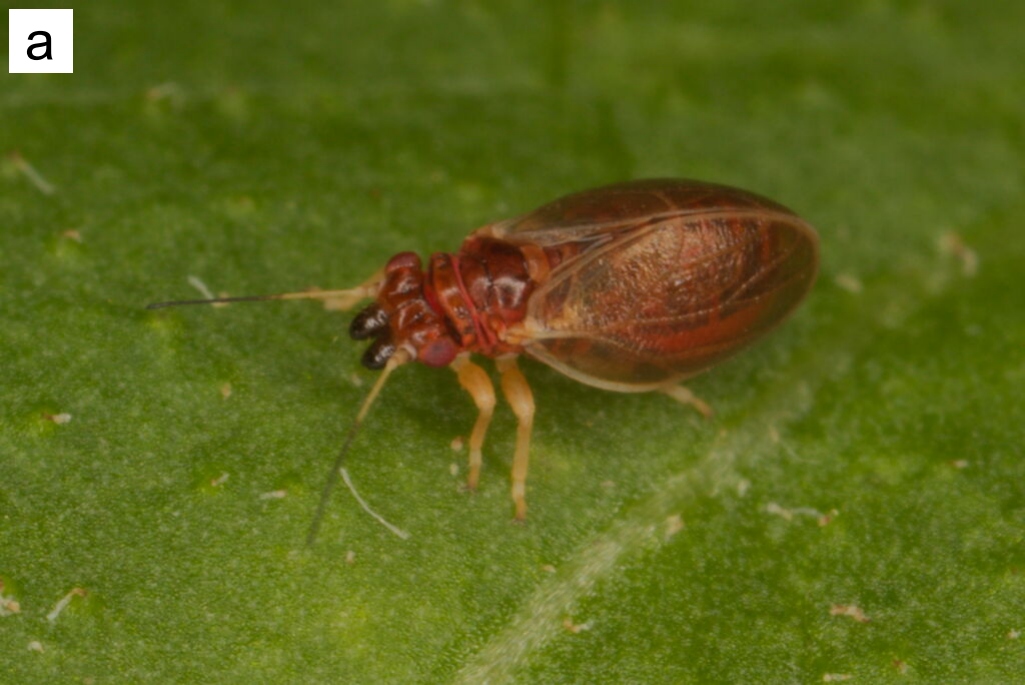
*Livillaulicis*, adult;

**Figure 85b.**
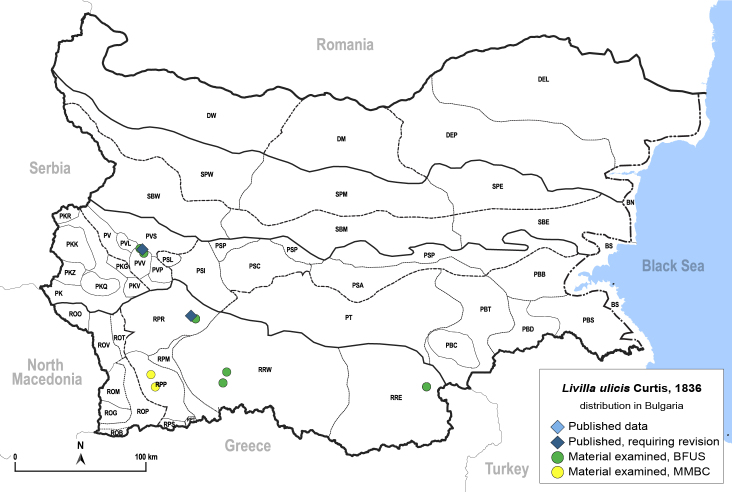
Distribution of *Livillaulicis* in Bulgaria.

**Figure 86a.**
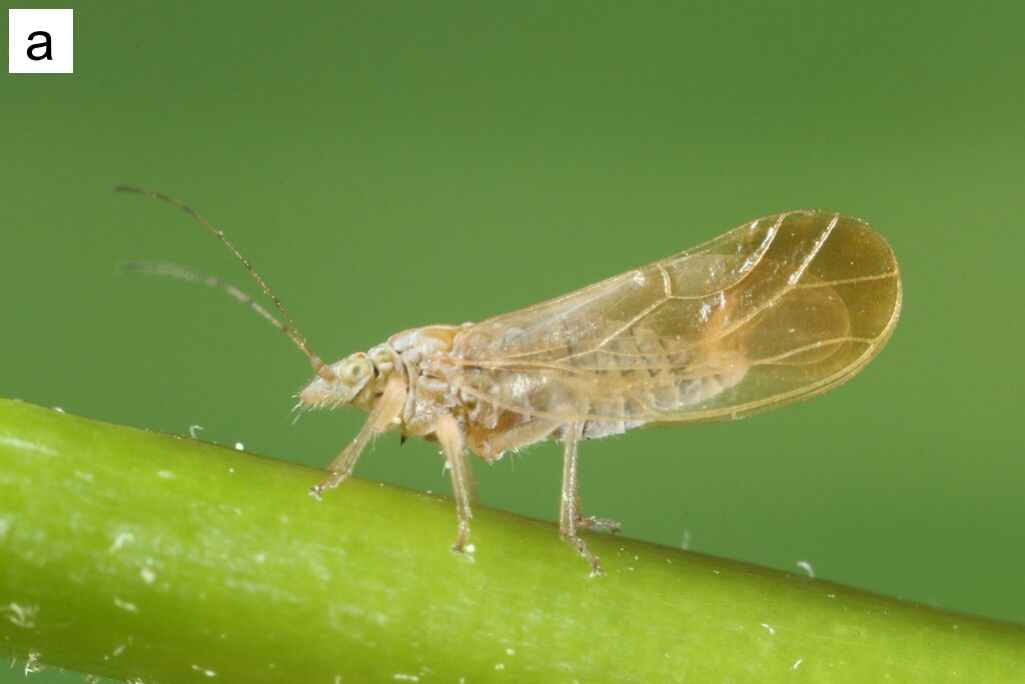
*Livillavariegata*, adult male;

**Figure 86b.**
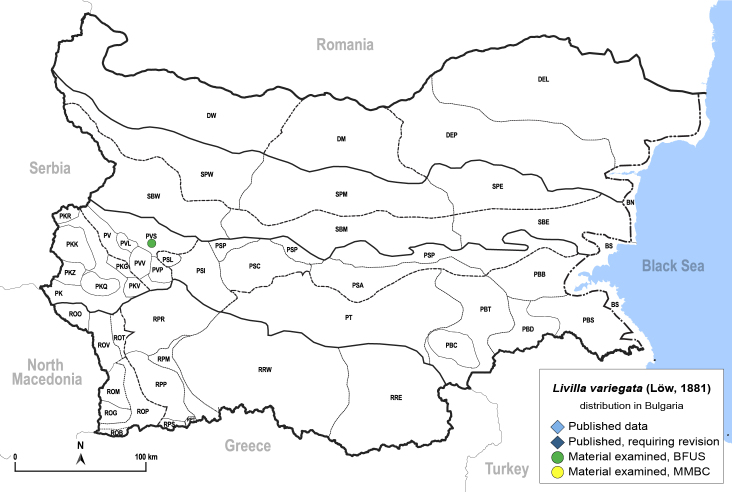
Distribution of *Livillavariegata* in Bulgaria.

**Figure 87a.**
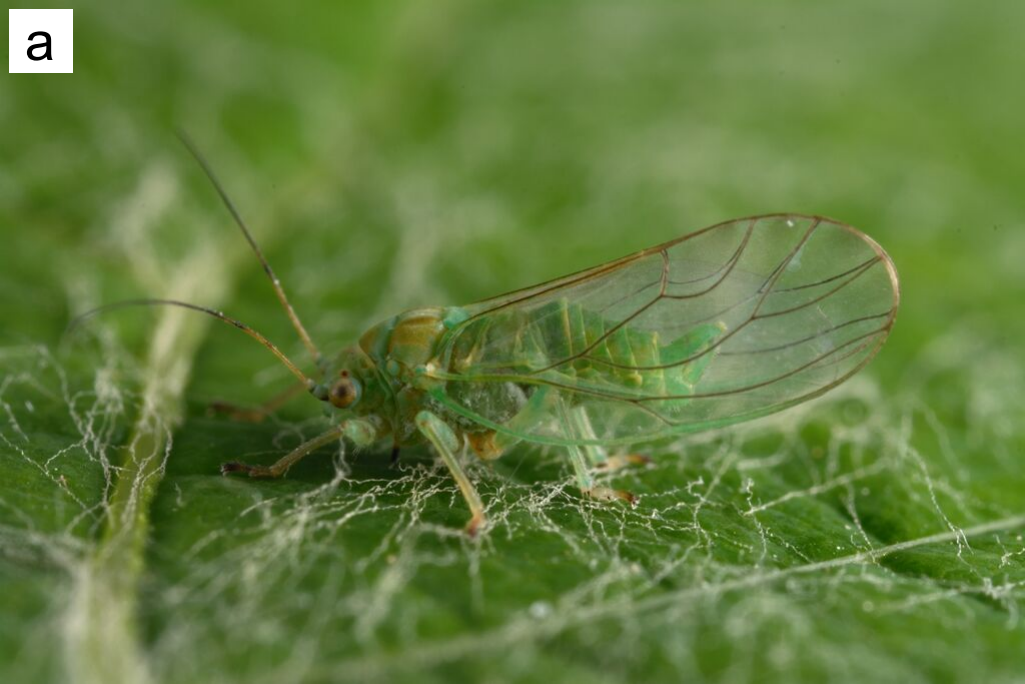
*Psyllaalni*, adult male.;

**Figure 87b.**
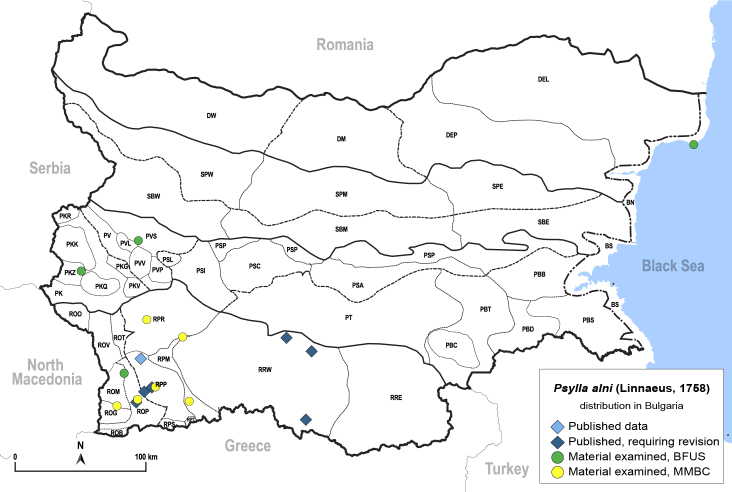
Distribution of *Psyllaalni* in Bulgaria.

**Figure 88a.**
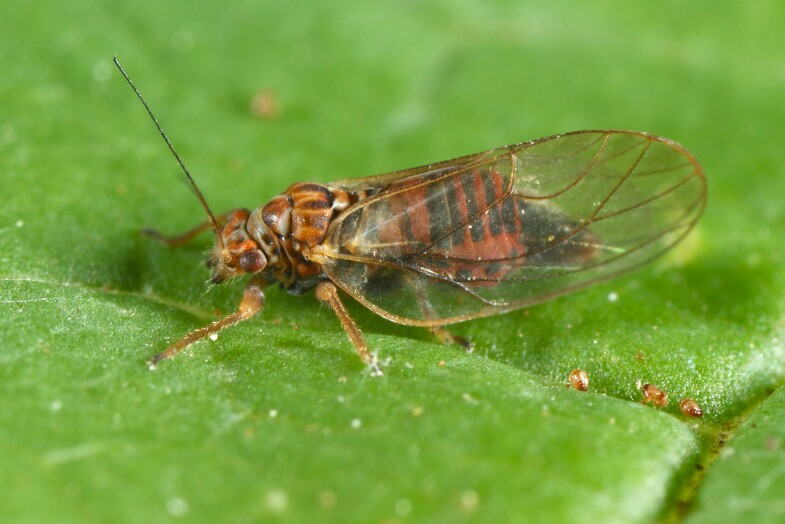
*Psyllaalpina*, adult female;

**Figure 88b.**
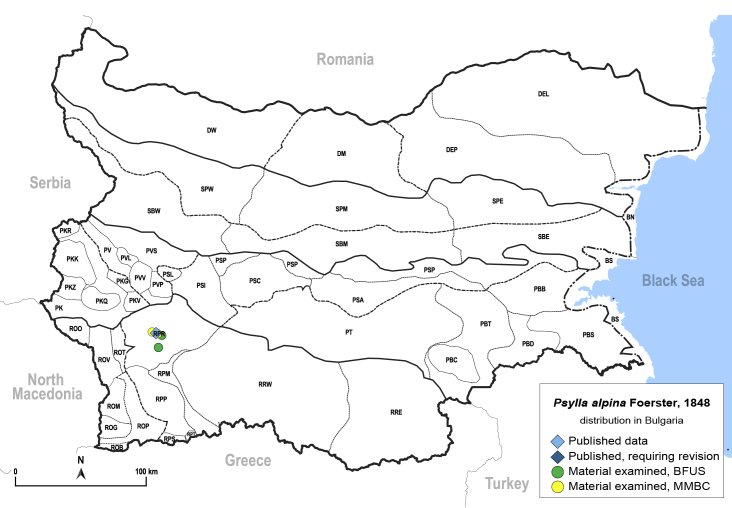
Distribution of *Psyllaalpina* in Bulgaria.

**Figure 90a.**
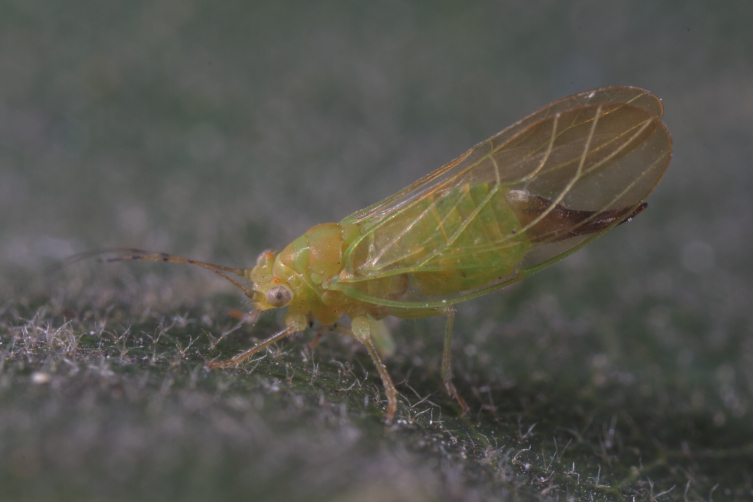
*Psyllafoersteri*, adult female;

**Figure 90b.**
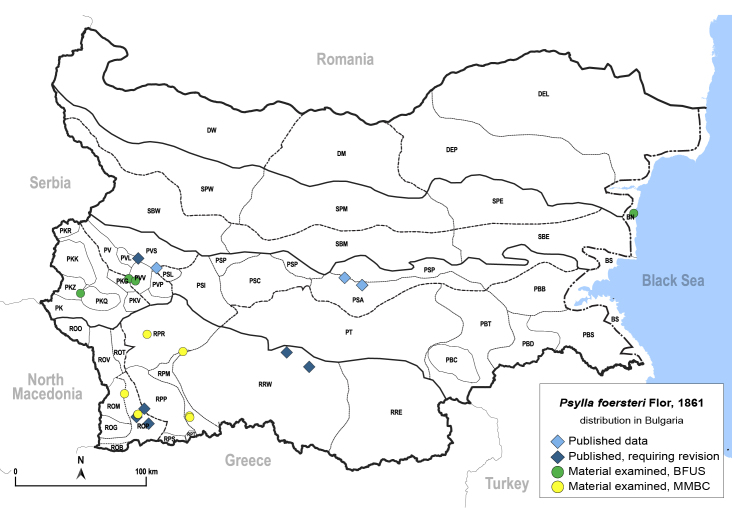
Distribution of *Psyllafoersteri* in Bulgaria.

**Figure 93a.**
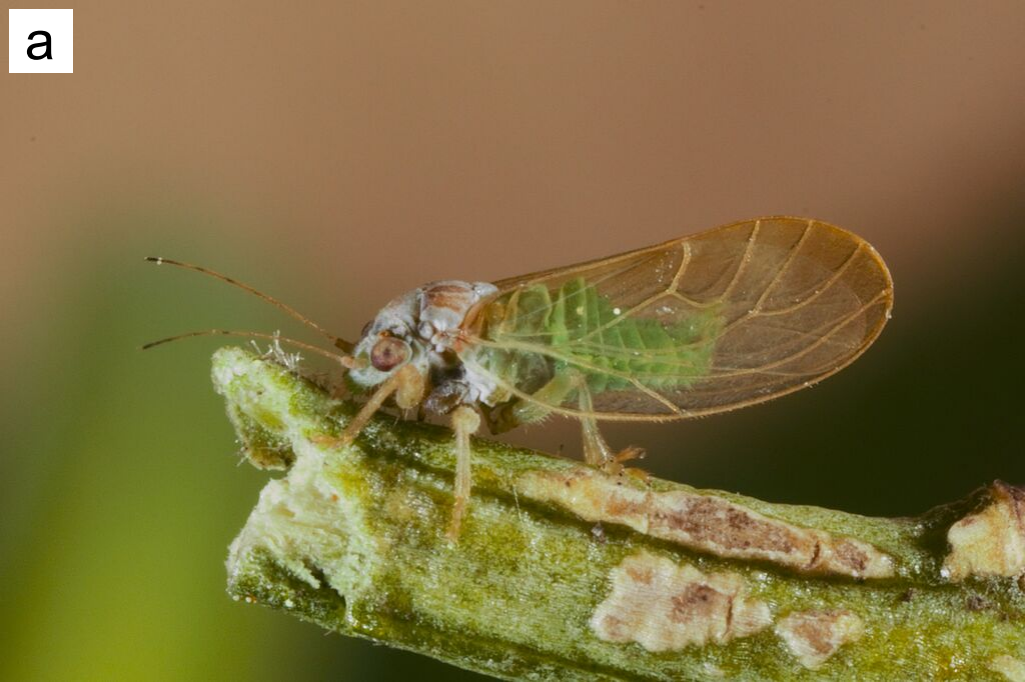
*Spanioneurabuxi*, adult male;

**Figure 93b.**
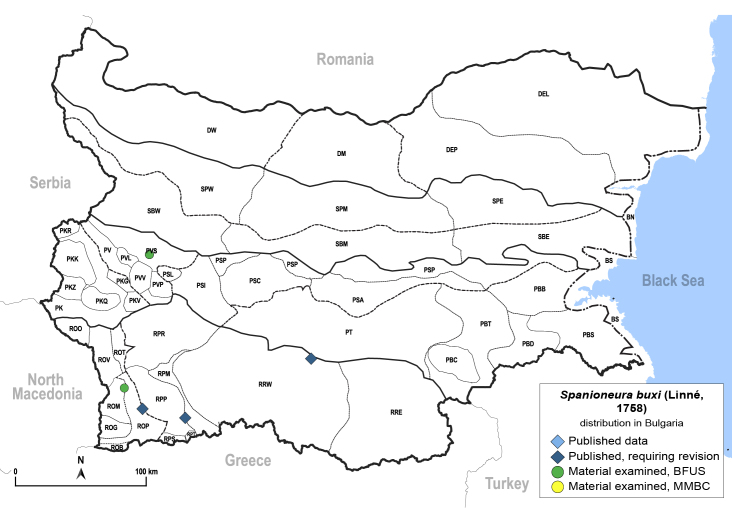
Distribution of *Spanioneurabuxi* in Bulgaria.

**Figure 95a.**
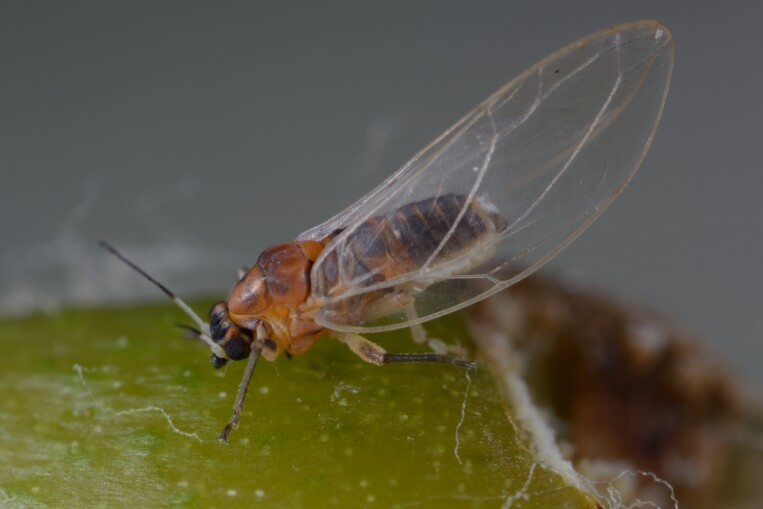
*Bactericeraalbiventris*, adult female;

**Figure 95b.**
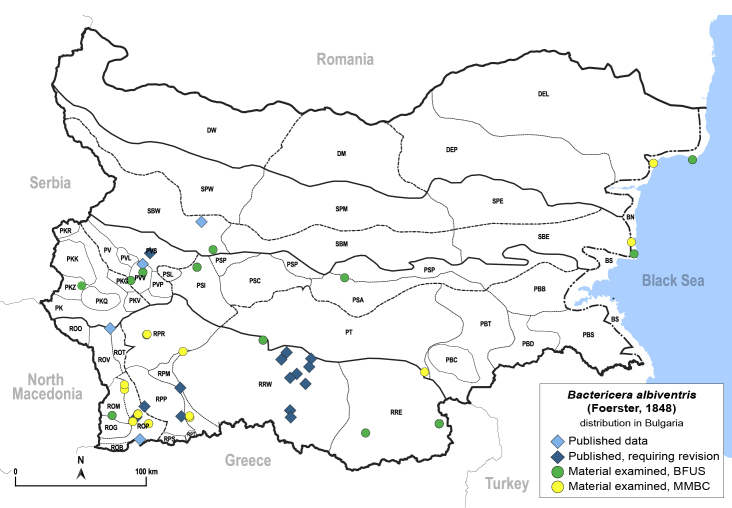
Distribution of *Bactericeraalbiventris* in Bulgaria.

**Figure 98a.**
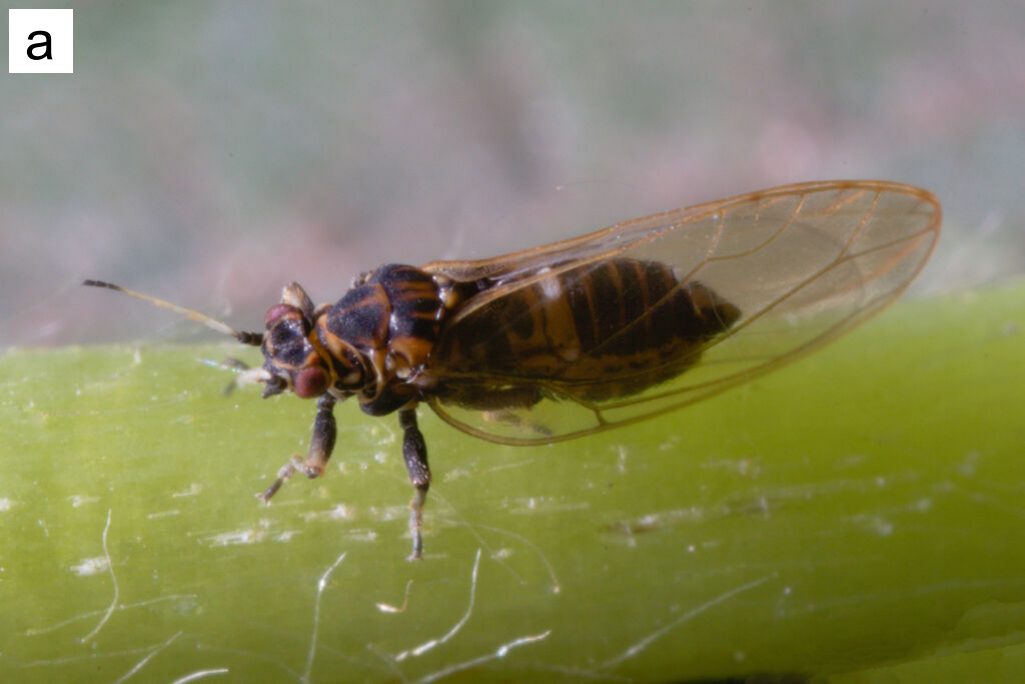
*Bactericerafemoralis*, adult female;

**Figure 98b.**
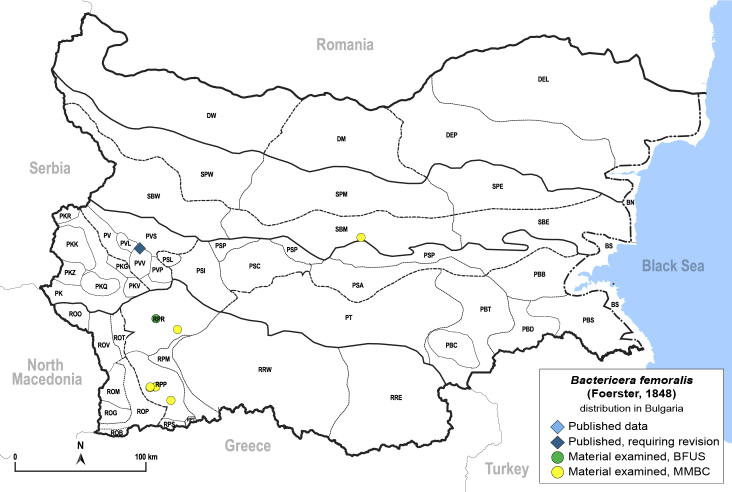
Distribution of *Bactericerafemoralis* in Bulgaria.

**Figure 100a.**
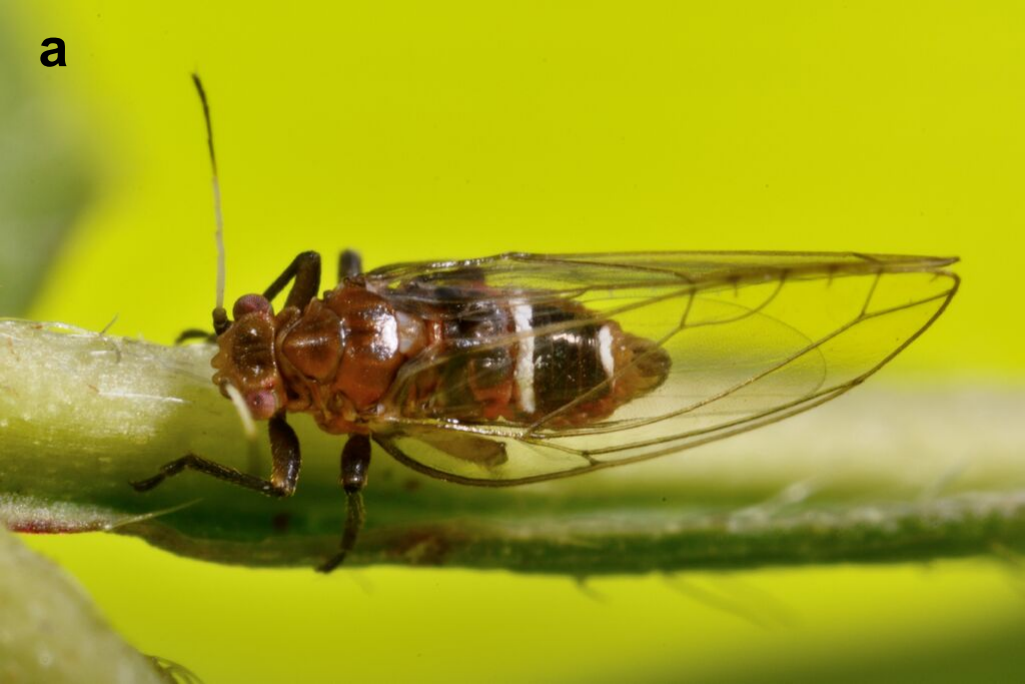
*Bactericeralyrata*, adult male;

**Figure 100b.**
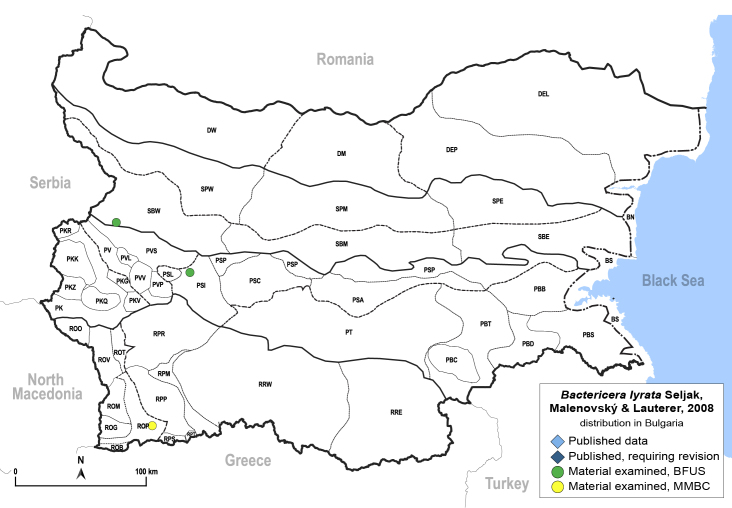
Distribution of *Bactericeralyrata* in Bulgaria.

**Figure 101a.**
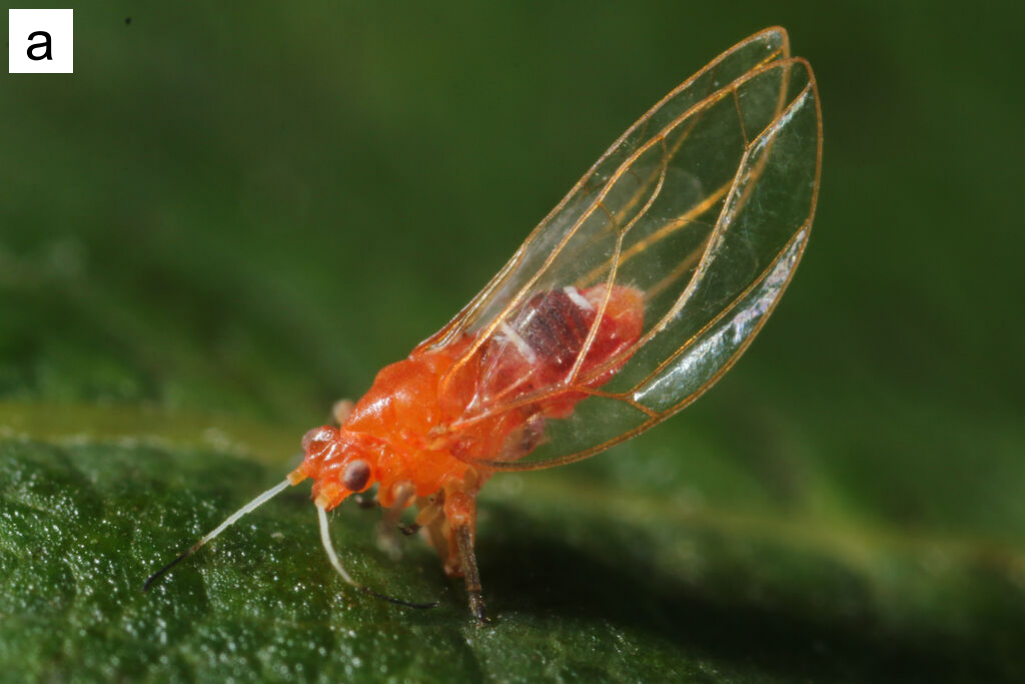
*Bactericeramodesta*, adult female;

**Figure 101b.**
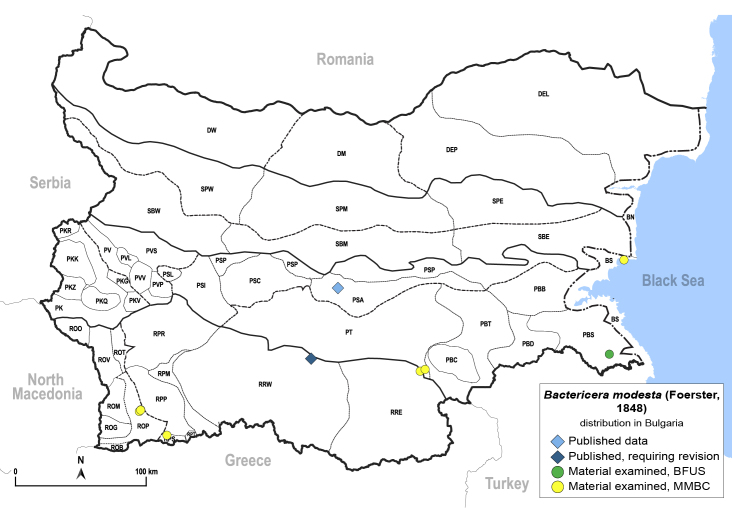
Distribution of *Bactericeramodesta* in Bulgaria.

**Figure 102a.**
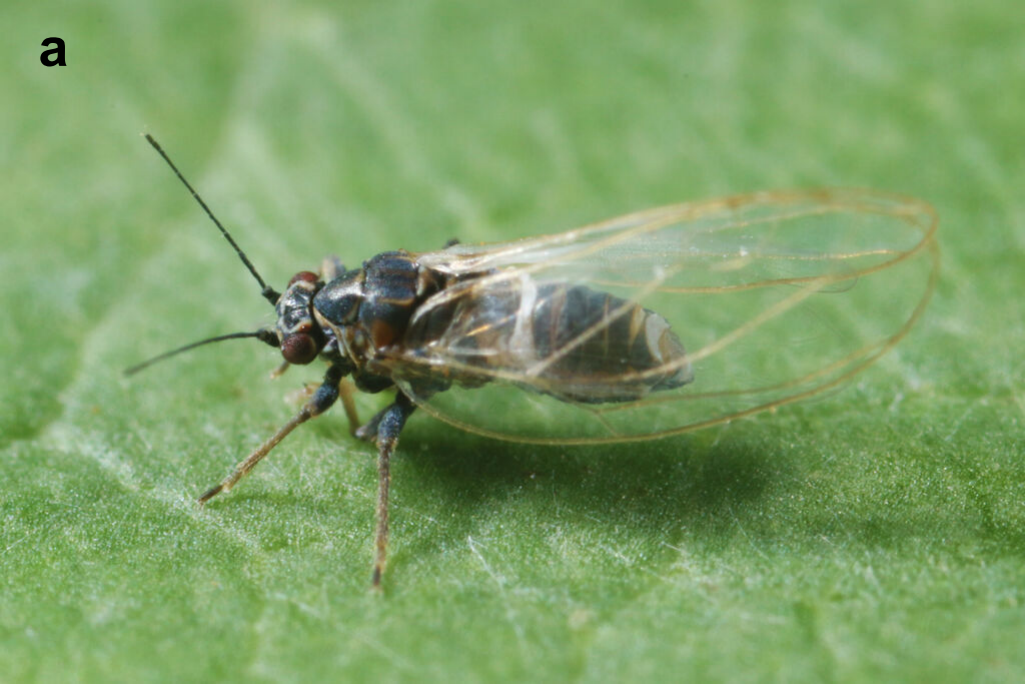
*Bactericeranigricornis*, adult female;

**Figure 102b.**
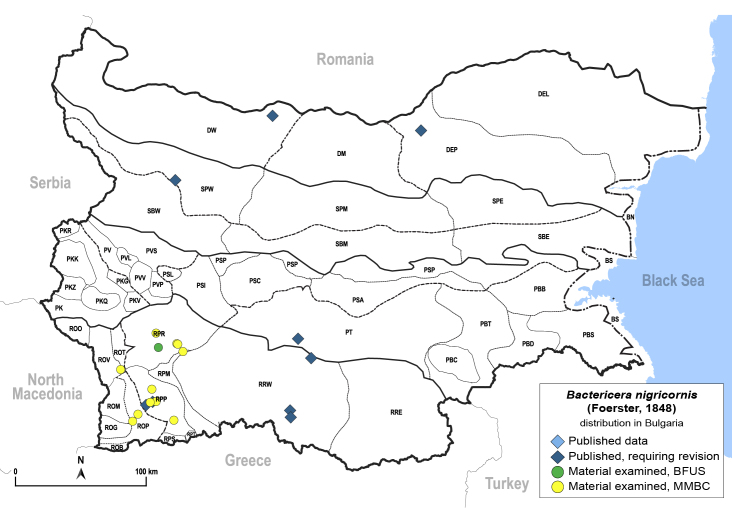
Distribution of *Bactericeranigricornis* in Bulgaria.

**Figure 103a.**
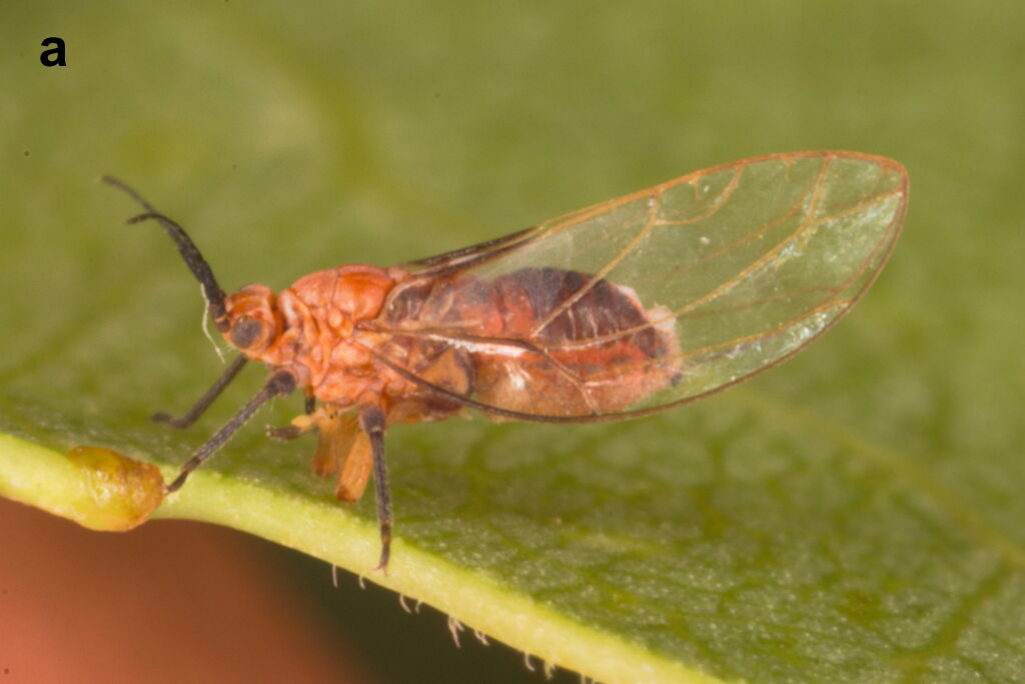
*Bactericeraperrisii*, adult female;

**Figure 103b.**
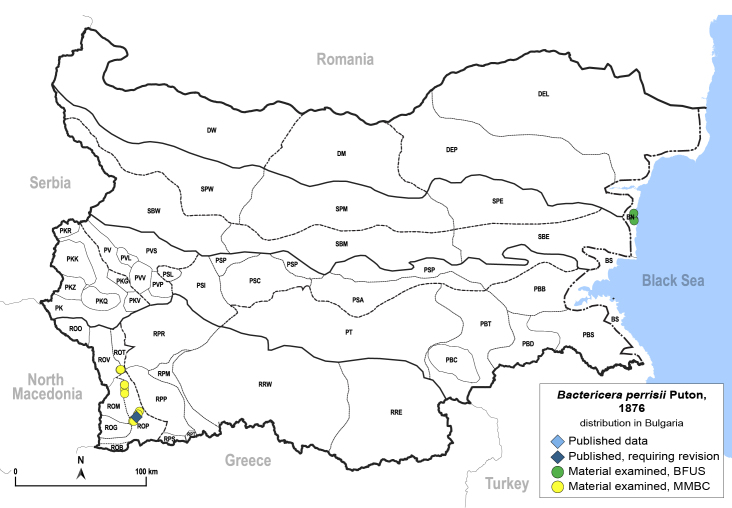
Distribution of *Bactericeraperrisii* in Bulgaria.

**Figure 104a.**
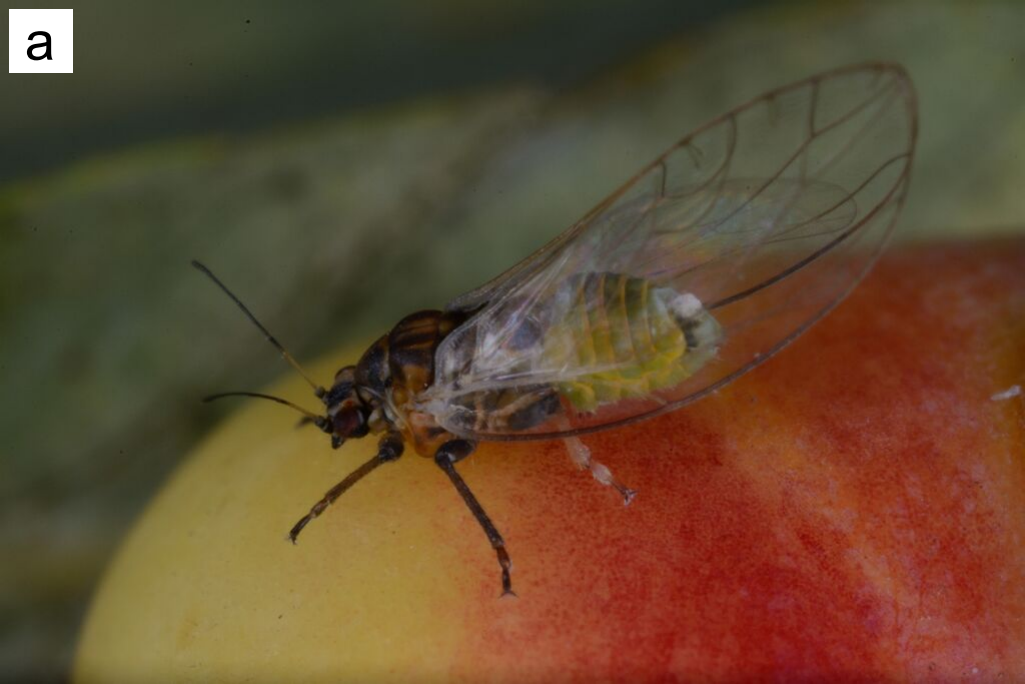
*Bactericerastriola*, adult female;

**Figure 104b.**
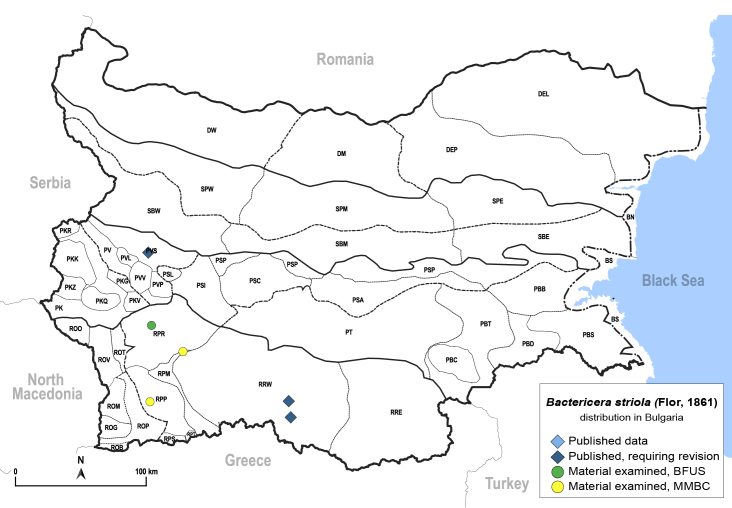
Distribution of *Bactericerastriola* in Bulgaria.

**Figure 105a.**
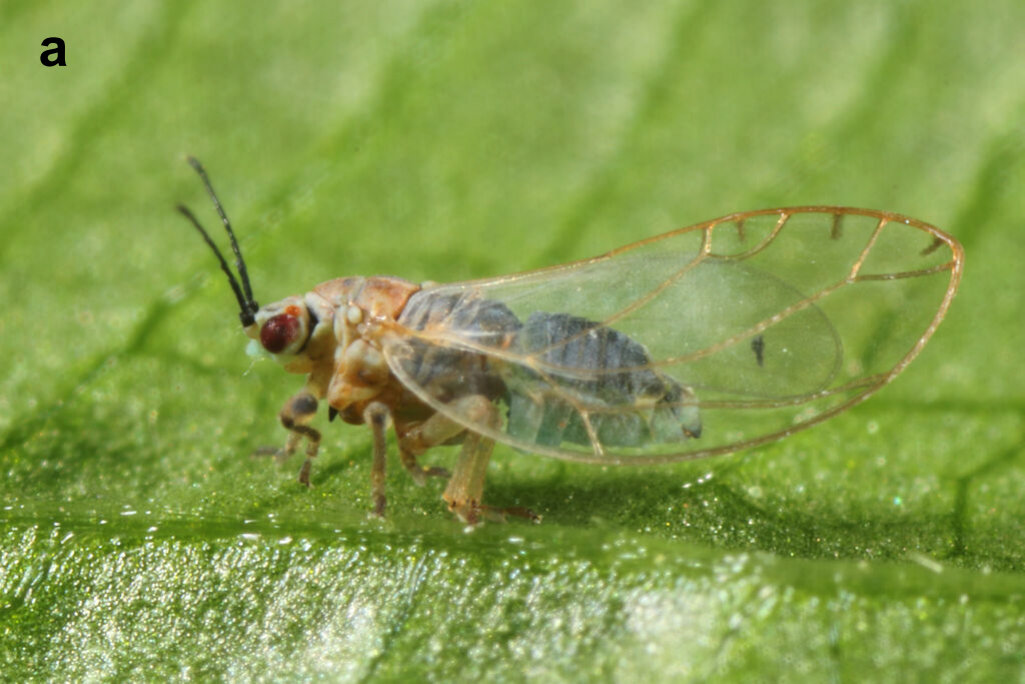
*Bactericeratrigonica*, adult female;

**Figure 105b.**
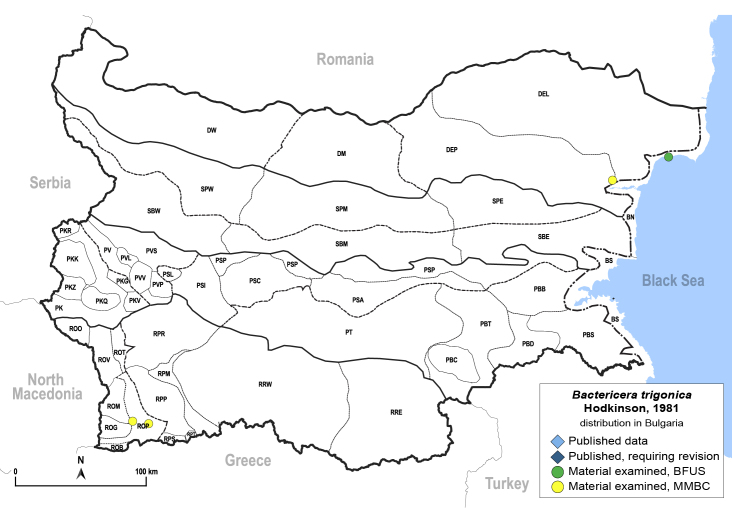
Distribution of *Bactericeratrigonica* in Bulgaria.

**Figure 107a.**
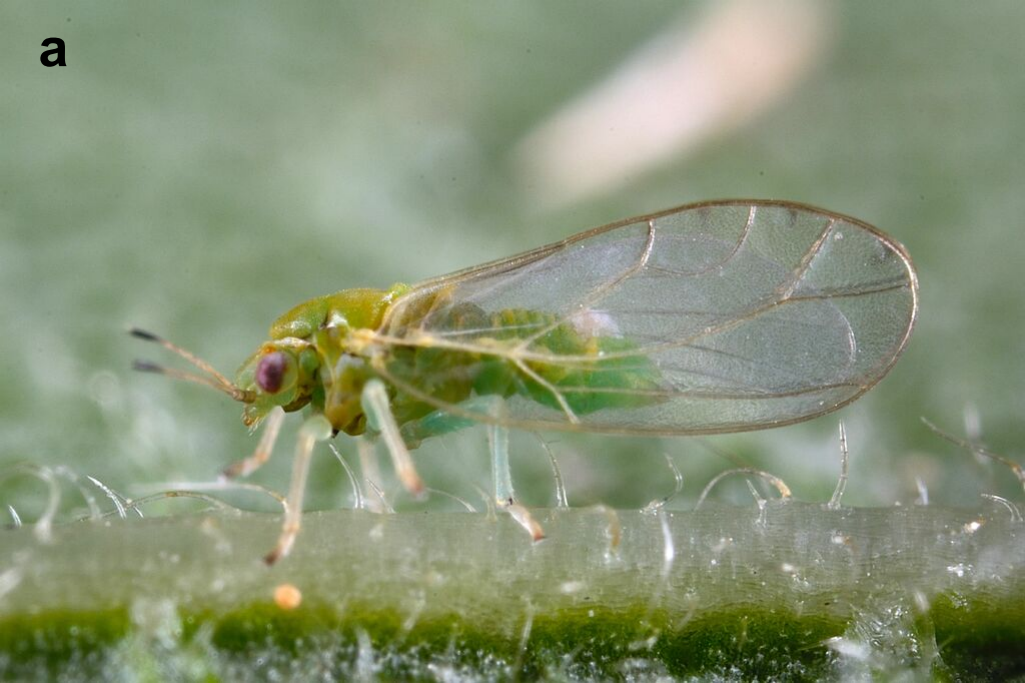
*Dyspersacirsii*, adult female;

**Figure 107b.**
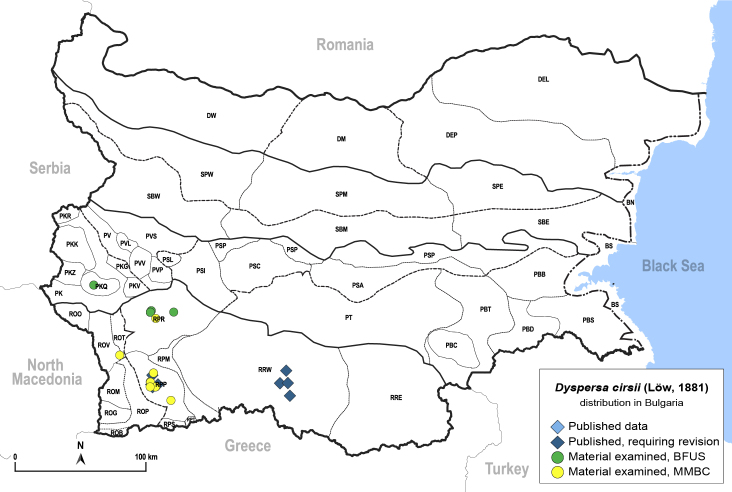
Distribution of *Dyspersacirsii* in Bulgaria.

**Figure 108a.**
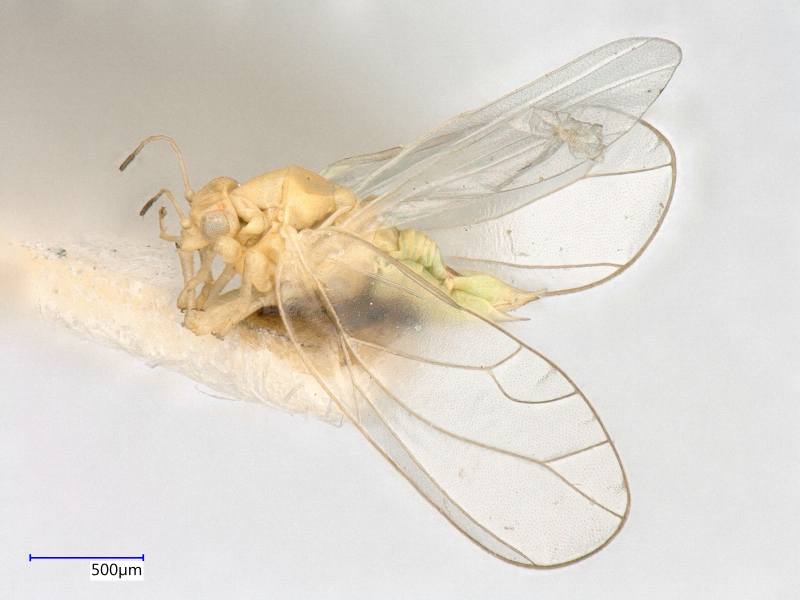
*Dyspersakantshavelii*, adult female;

**Figure 108b.**
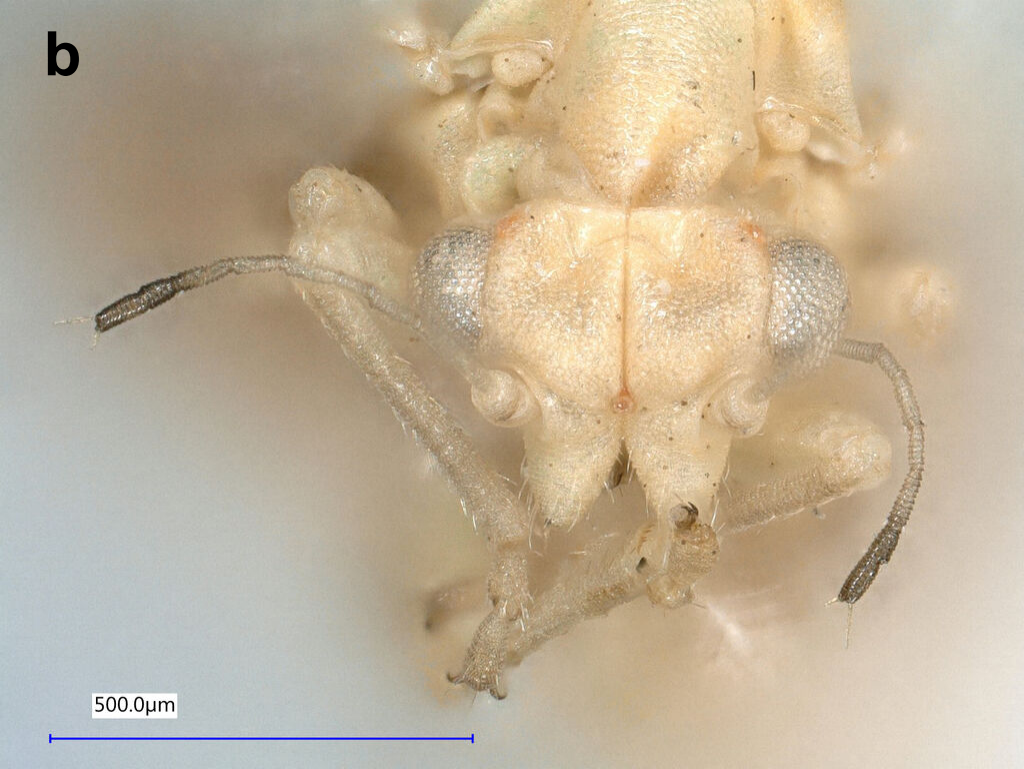
*Dyspersakantshavelii*, head, oblique frontal view;

**Figure 108c.**
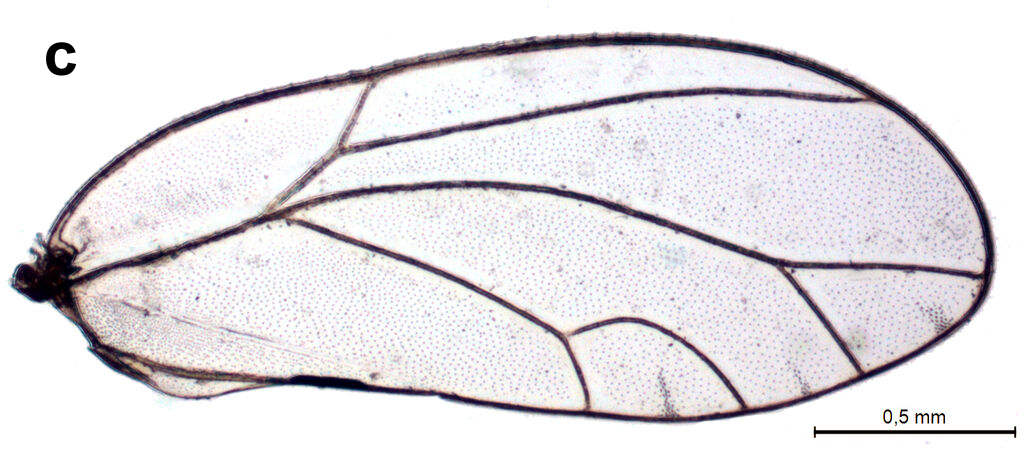
*Dyspersakantshavelii*, forewing;

**Figure 108d.**
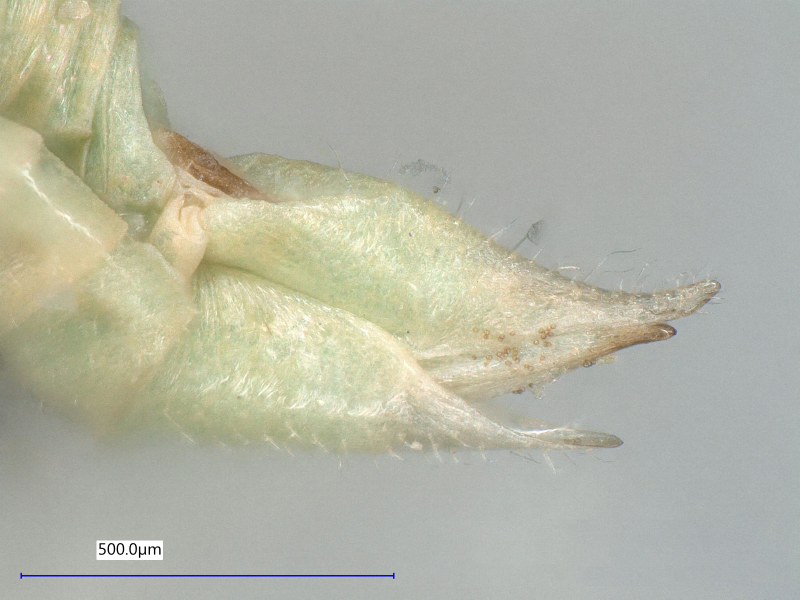
*Dyspersakantshavelii*, female terminalia, lateral view;

**Figure 108e.**
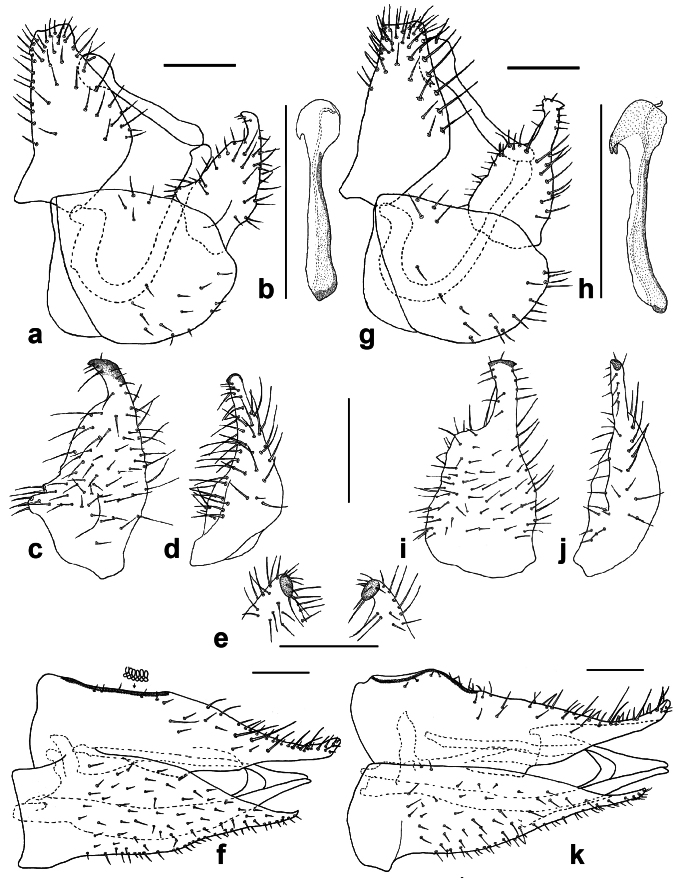
Comparison between male and female terminalia of *Dyspersakantshavelii* (specimen from Bulgaria) and *D.agrophila* (Löw, 1888) (specimen from the Czechia). ***D.kantshavelii***, **a** – male terminalia, lateral view; **b** – distal segment of aedeagus, lateral view; **c** – paramere, inner surface, lateral view; **d** – paramere, posterior view; **e** – paramere, dorsal view; **f** – female terminalia, lateral view; ***D.agrophila***, **g** – male terminalia, lateral view; **h** – distal segment of aedeagus, lateral view; **i** – paramere, inner surface, lateral view; **j** – paramere, posterior view; **k** – female terminalia, lateral view. Scale bars: 0.1 mm;

**Figure 108f.**
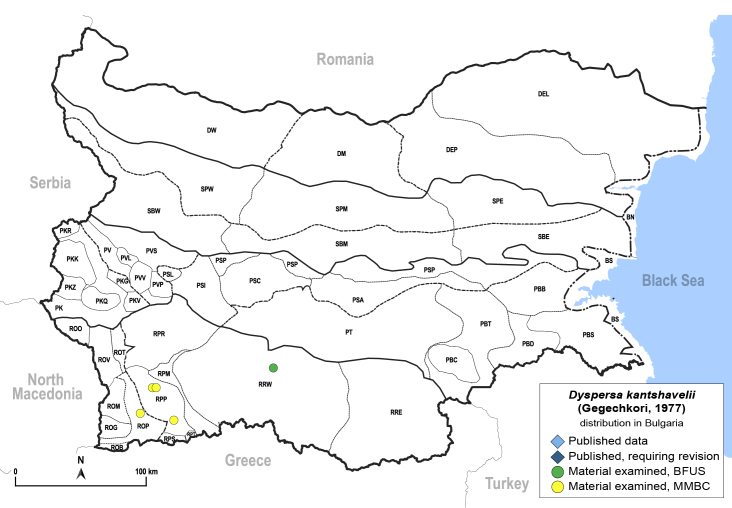
Distribution of *Dyspersakantshavelii* in Bulgaria.

**Figure 112a.**
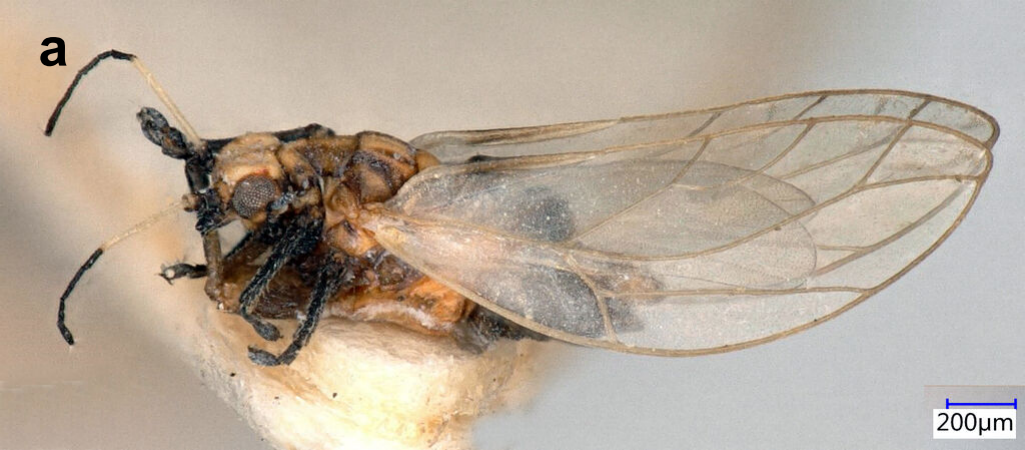
*Eryngiofagababugani*, adult female;

**Figure 112b.**
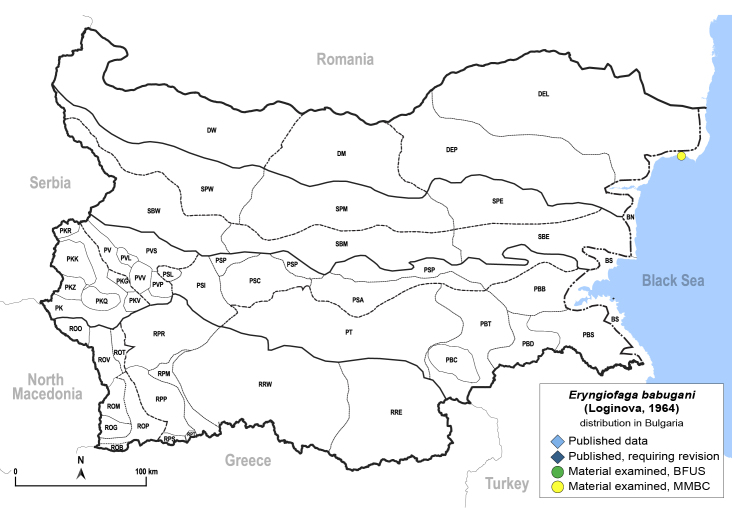
Distribution of *Eryngiofagababugani* in Bulgaria.

**Figure 113a.**
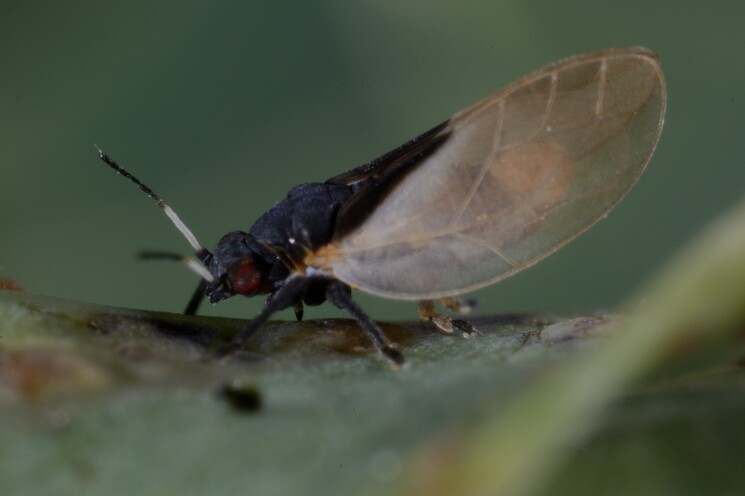
*Eryngiofagadlabolai*, adult female;

**Figure 113b.**
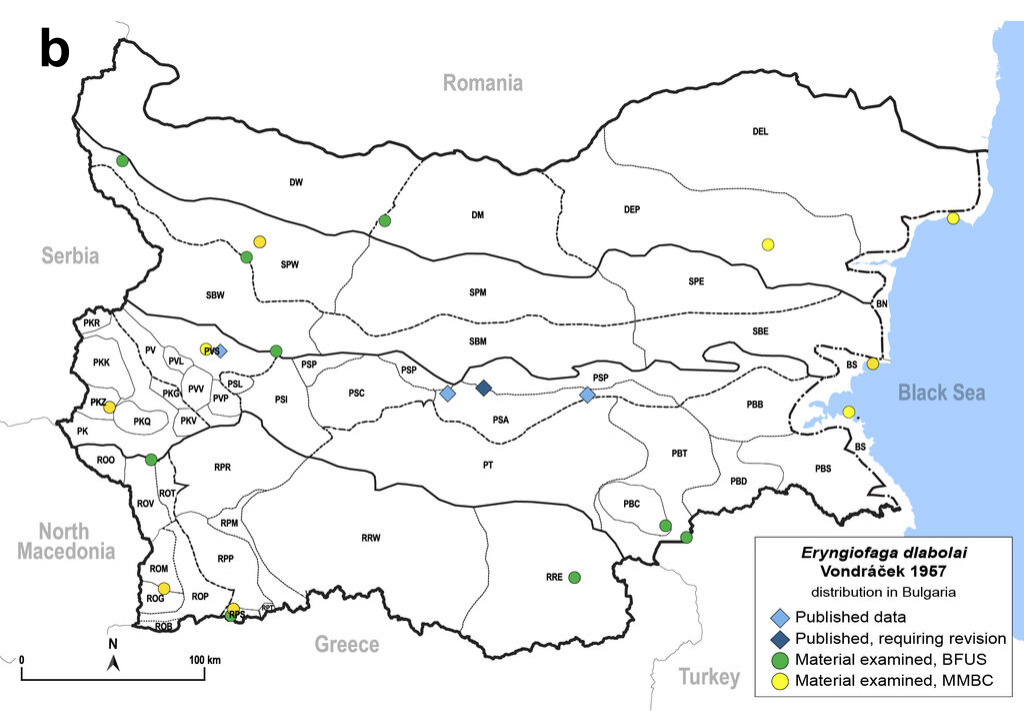
Distribution of *Eryngiofagadlabolai* in Bulgaria.

**Figure 114a.**
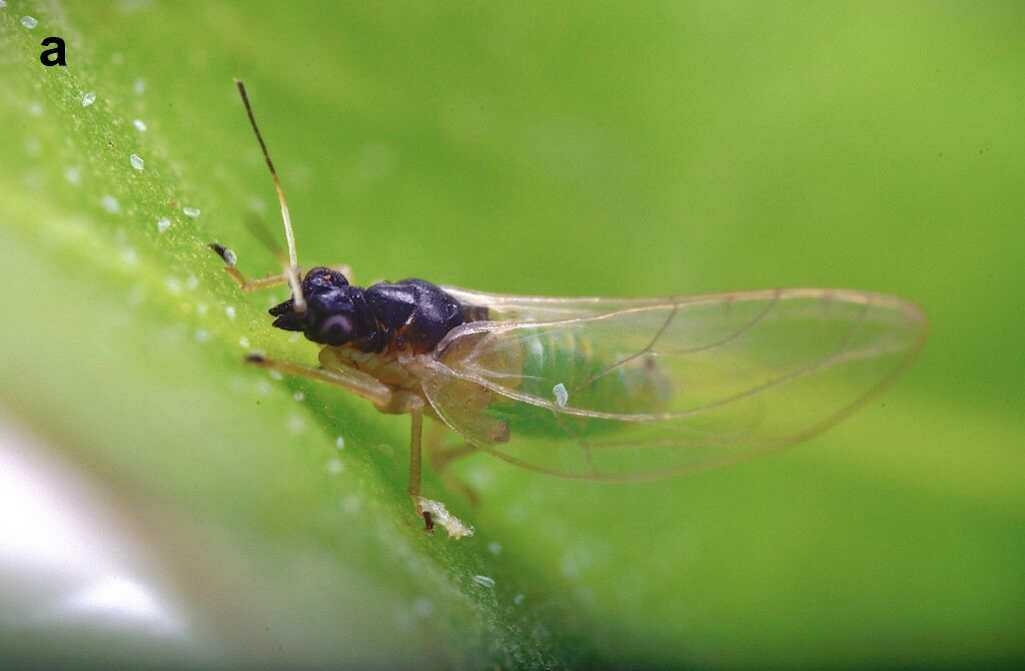
*Heterotriozachenopodii*, adult male;

**Figure 114b.**
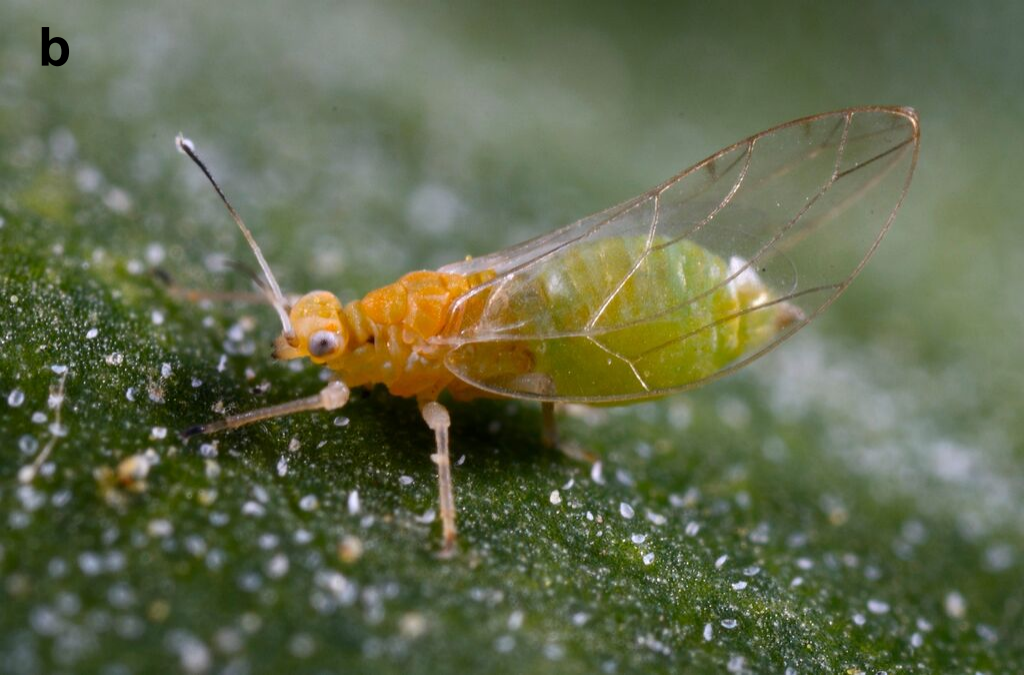
*Heterotriozachenopodii*, adult female;

**Figure 114c.**
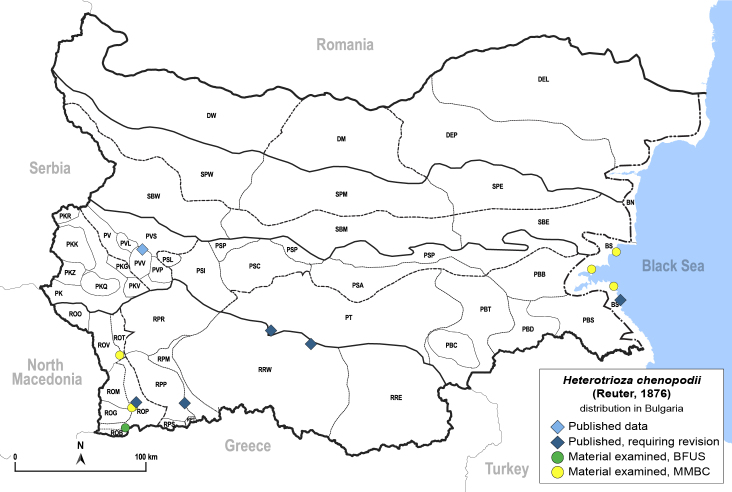
Distribution of *Heterotriozachenopodii* in Bulgaria.

**Figure 116a.**
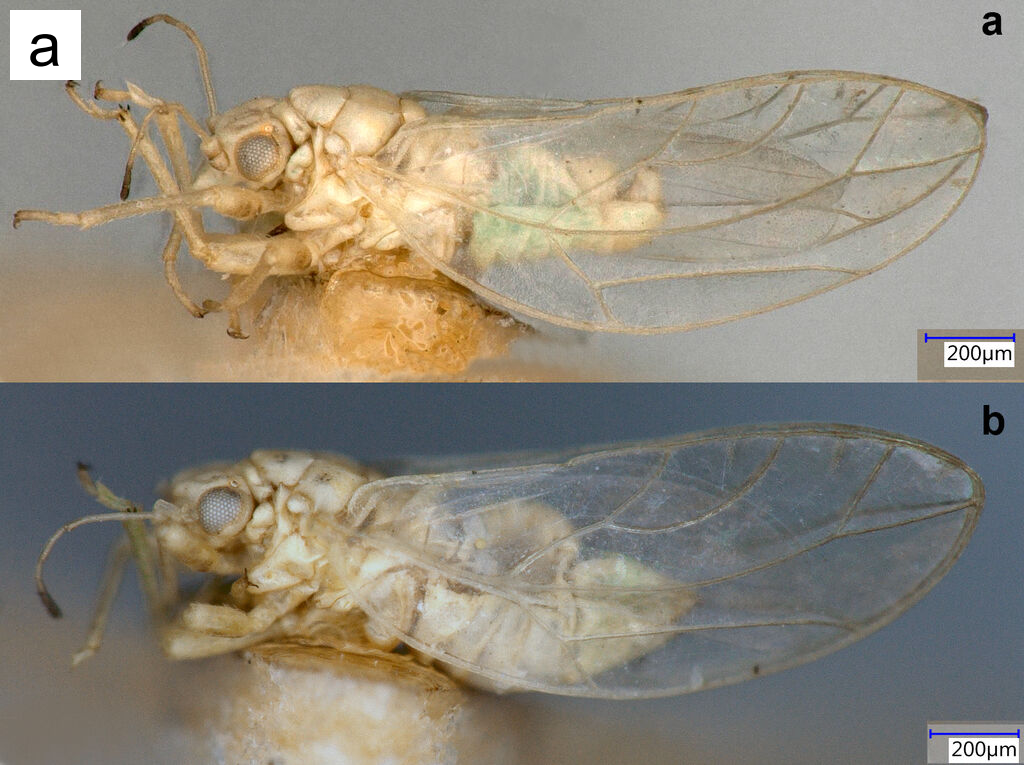
*Heteriotriozakochiae*: **a.** adult male; **b.** adult female;

**Figure 116b.**
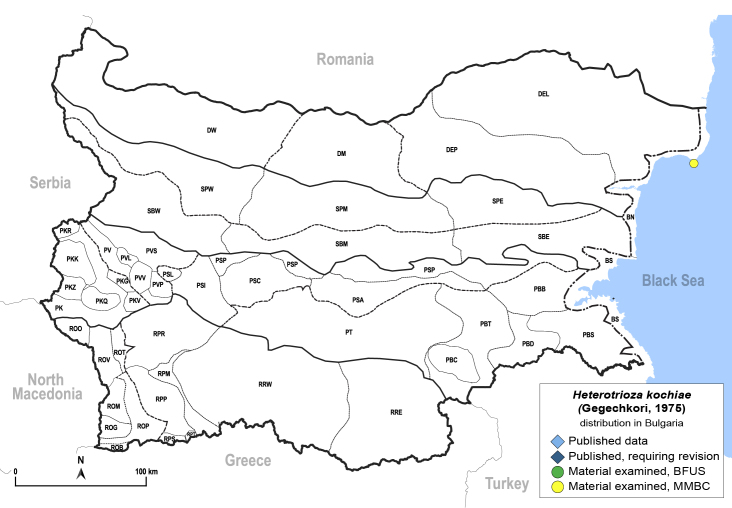
Distribution of *Heterotriozakochiae* in Bulgaria;

**Figure 116c.**
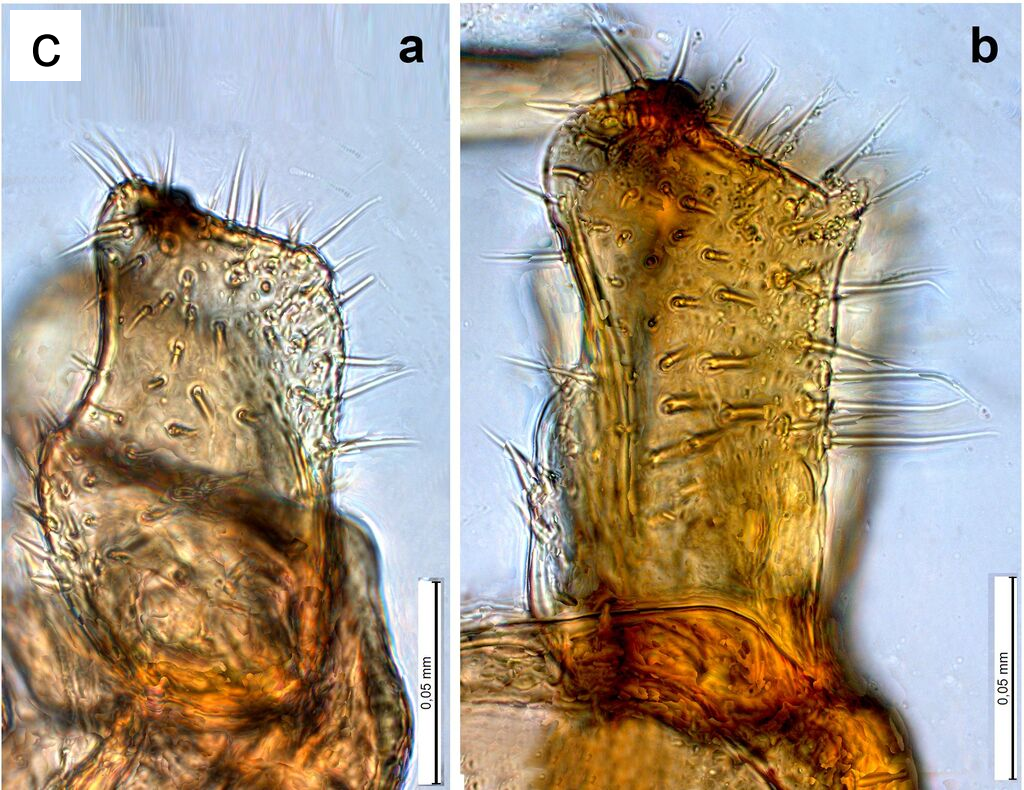
Comparison between male parameres of **a.** *Heteriotriozakochiae* from Bulgaria and **b.** *Heteriotriozaeurotiae* Loginova, 1960 from Kazakhstan;

**Figure 116d.**
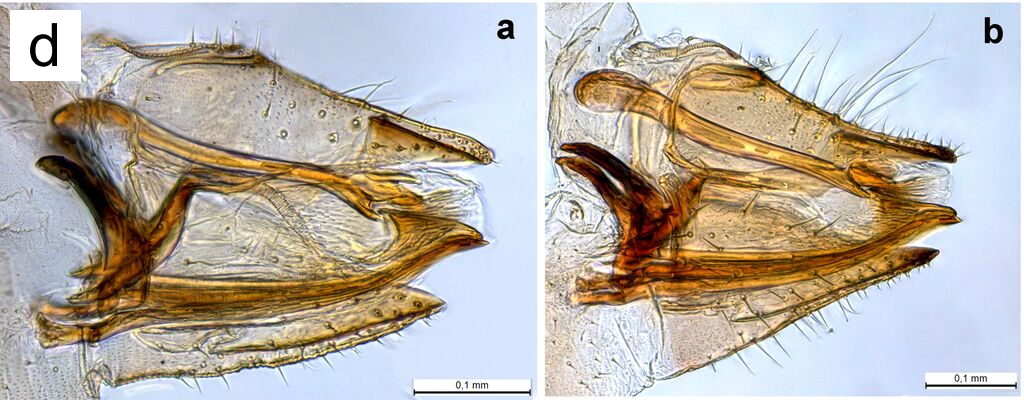
Comparison between female terminalia of **a.** *Heteriotriozakochiae* from Bulgaria and **b.** *Heteriotriozaeurotiae* Loginova, 1960 from Kazakhstan;

**Figure 116e.**
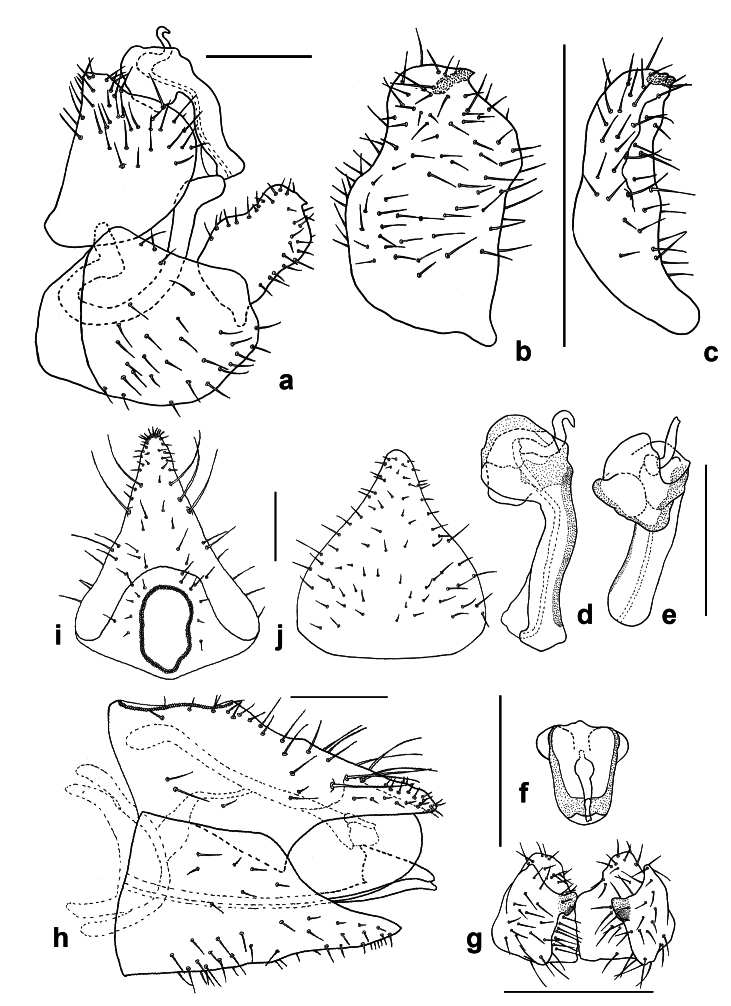
Male and female terminalia of *Heterotriozakochiae*. **a.** male terminalia in lateral view; **b.** paramere in lateral view; **c.** paramere in posterior view; **d.** distal segment of aedeagus in lateral view; **e.** distal segment of aedeagus in ventral view; **f.** distal segment of aedeagus in dorsal view; **g.** apices of parameres in dorsal view; **h.** female terminalia in lateral view; **i.** female proctiger in dorsal view; **j.** female subgenital plate in ventral view.

**Figure 117a.**
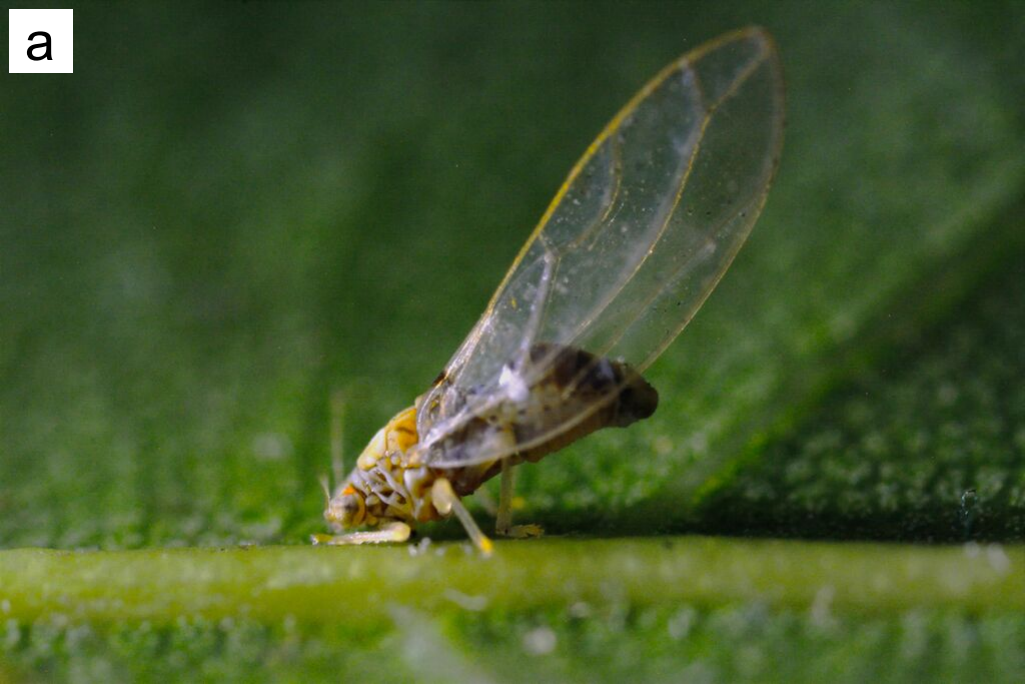
*Lauritriozaalacris*, adult male;

**Figure 117b.**
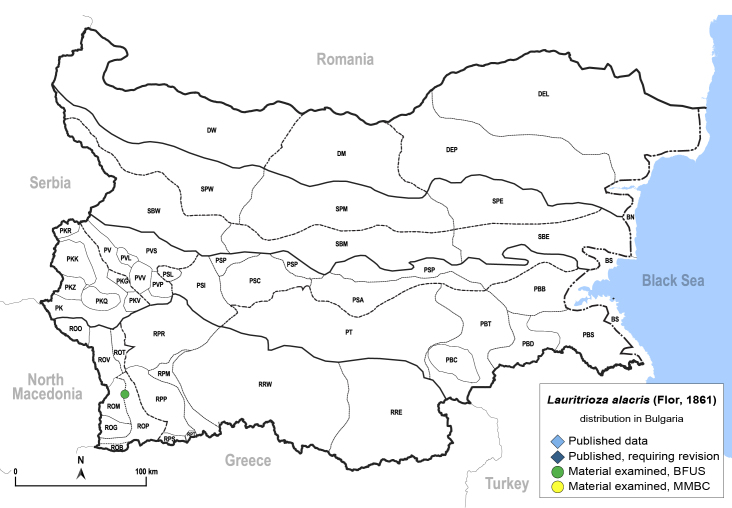
Distribution of *Lauritriozaalacris* in Bulgaria.

**Figure 118a.**
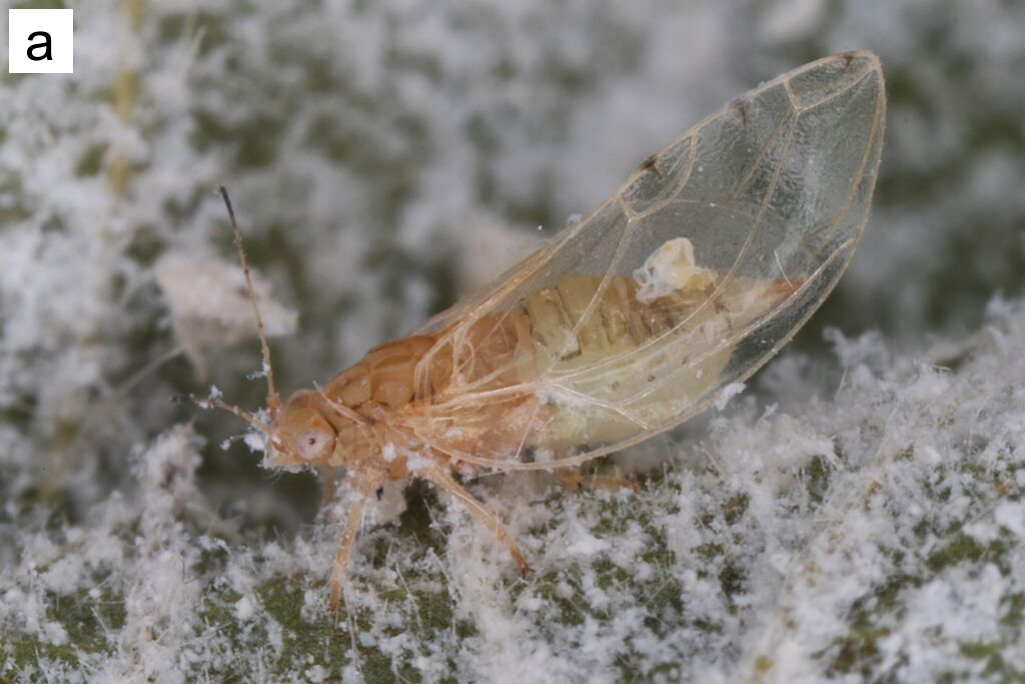
*Phylloplectatrisignata*, adult female;

**Figure 118b.**
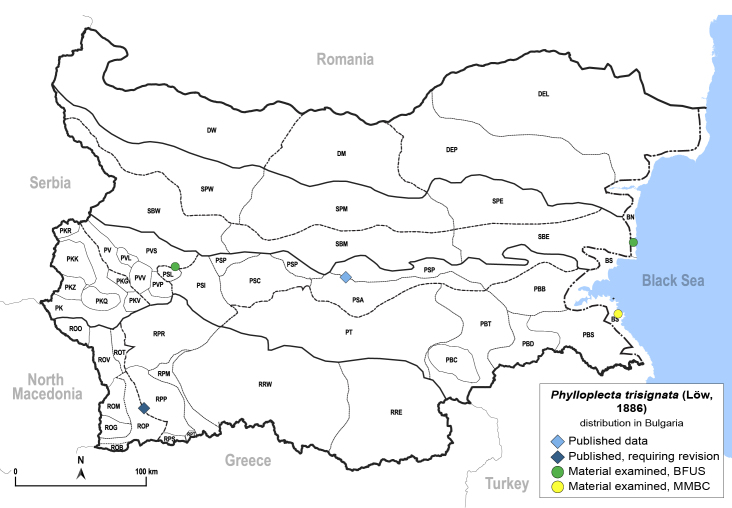
Distribution of *Phylloplectatrisignata* in Bulgaria.

**Figure 119a.**
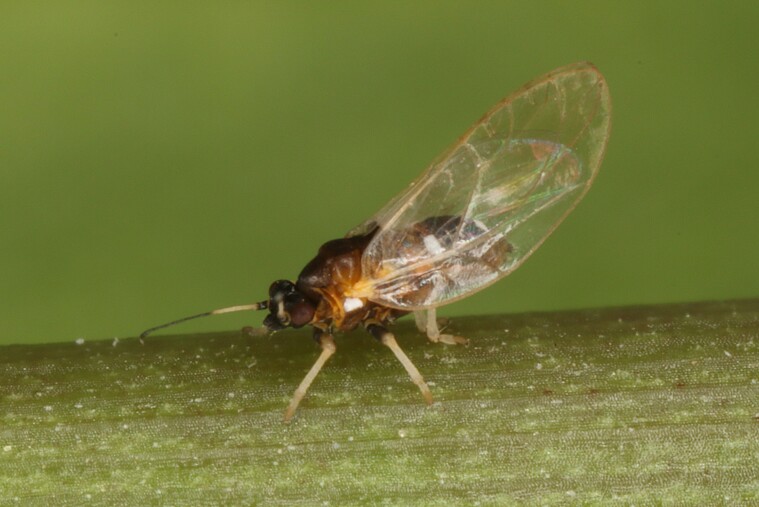
*Spaniozagalii*, adult female;

**Figure 119b.**
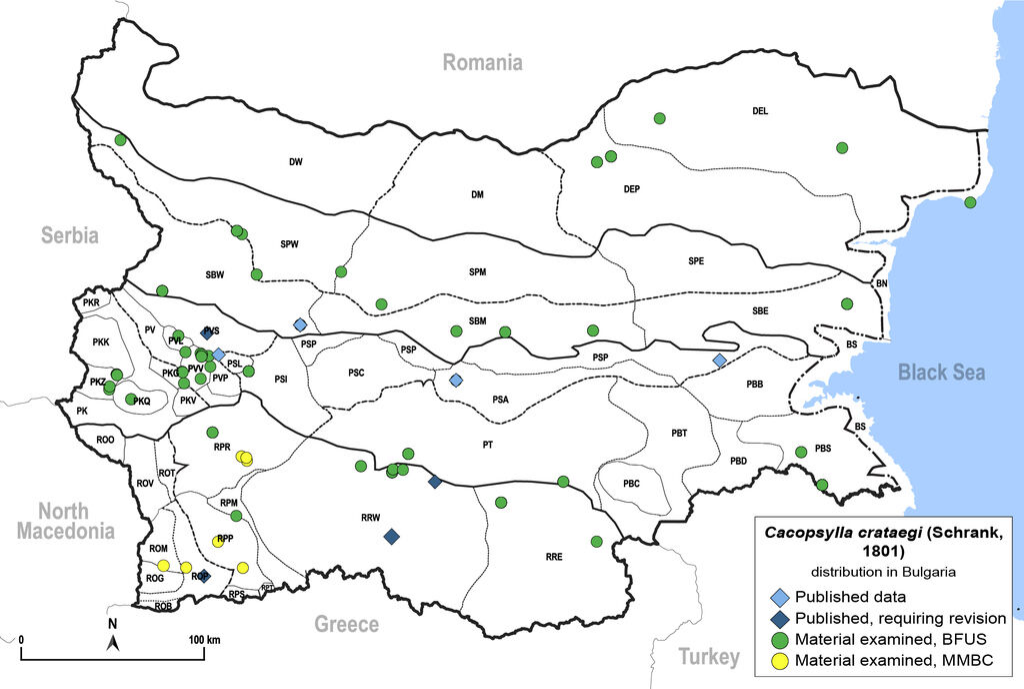
Distribution of *Spaniozagalii* in Bulgaria.

**Figure 120a.**
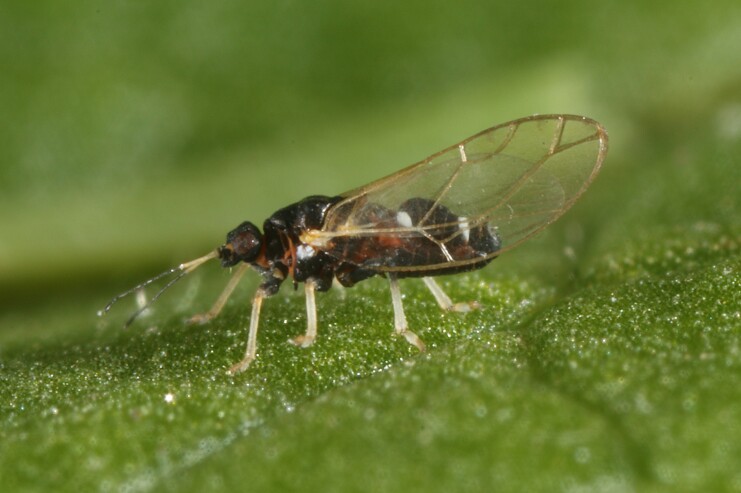
*Spaniozavelutina*, adult male;

**Figure 120b.**
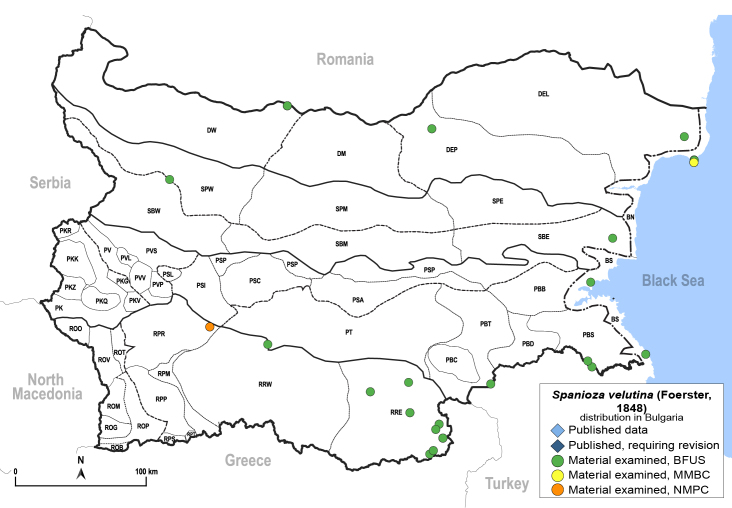
Distribution of *Spaniozavelutina* in Bulgaria.

**Figure 121a.**
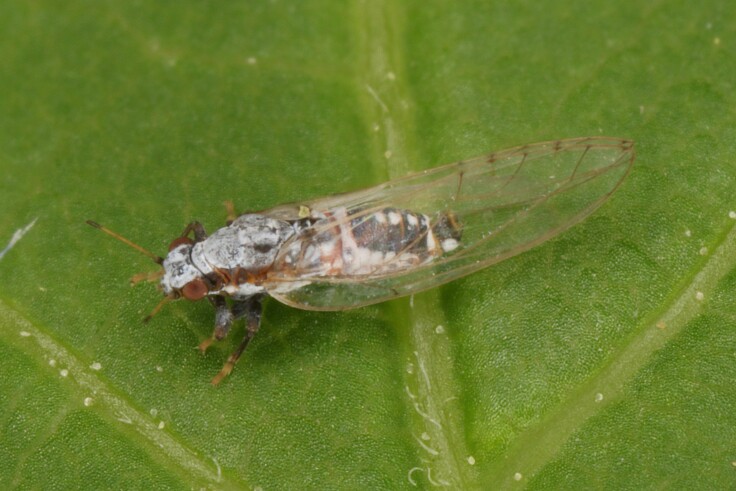
*Trichochermesrhamni*, adult male;

**Figure 121b.**
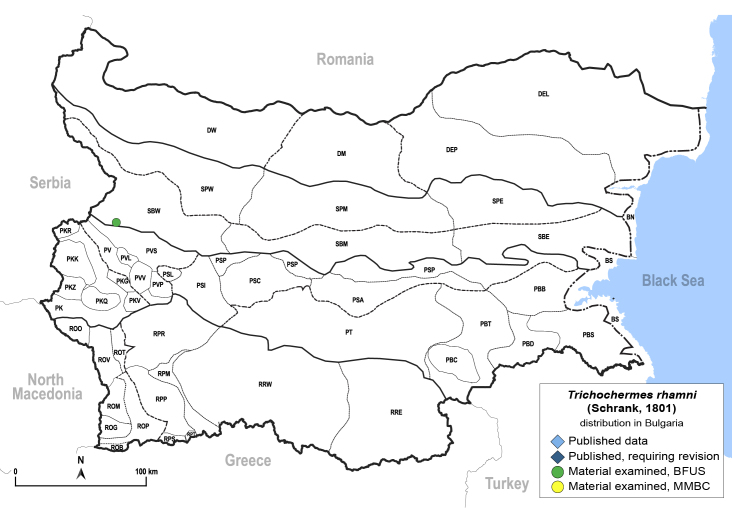
Distribution of *Trichochermesrhamni* in Bulgaria.

**Figure 126a.**
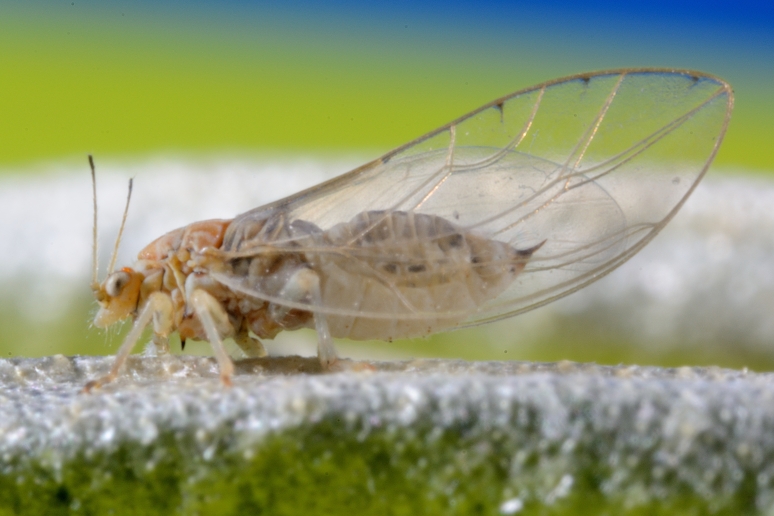
*Triozaneglecta*, adult female;

**Figure 126b.**
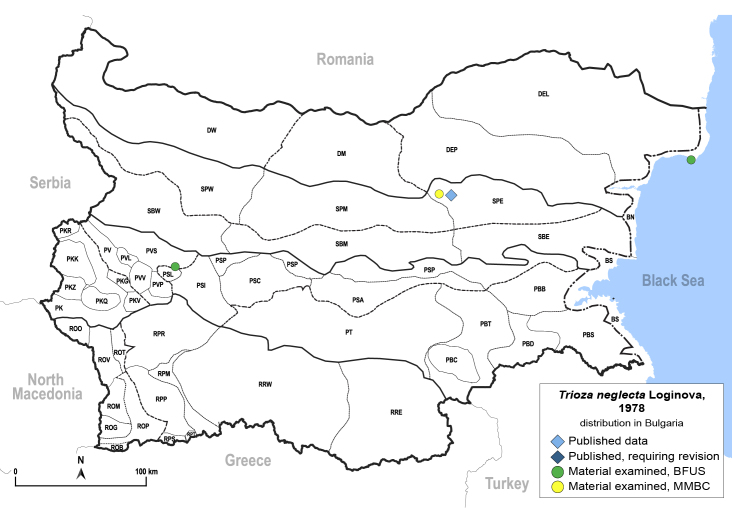
Distribution of *Triozaneglecta* in Bulgaria.

**Figure 127a.**
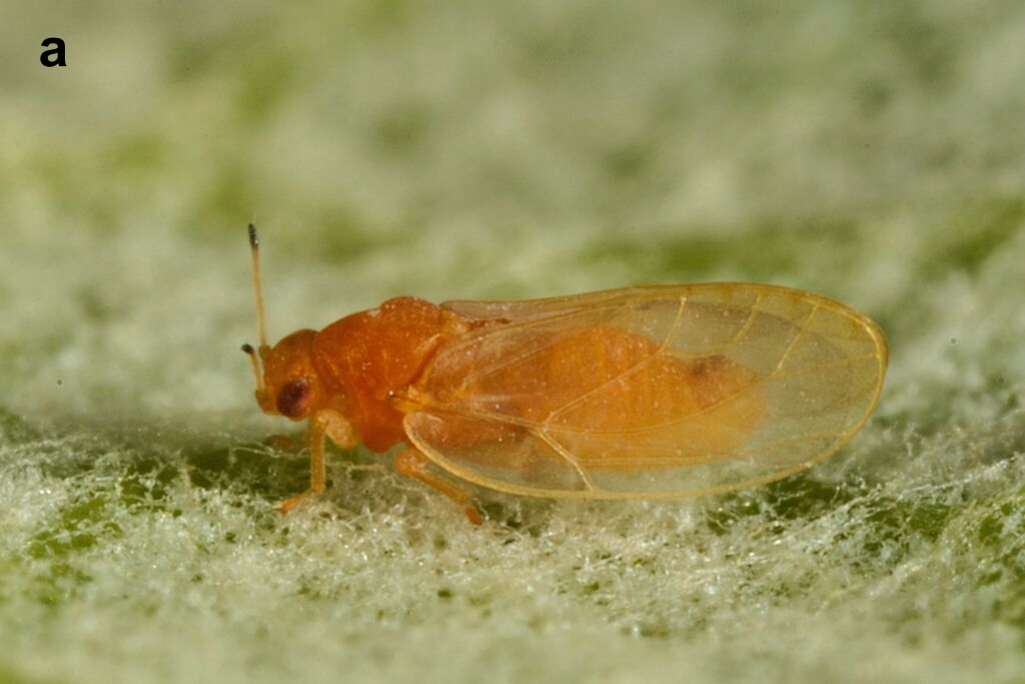
*Triozaproxima*. adult female;

**Figure 127b.**
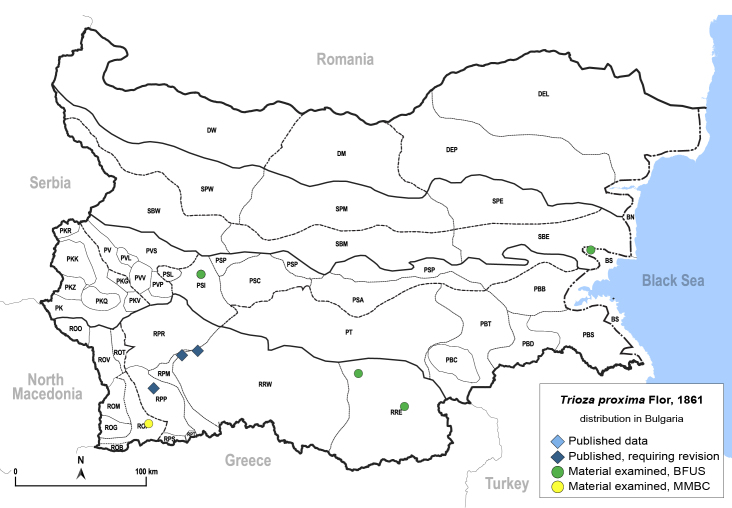
Distribution of *Triozaproxima* Flor, 1861 in Bulgaria.

**Figure 131a.**
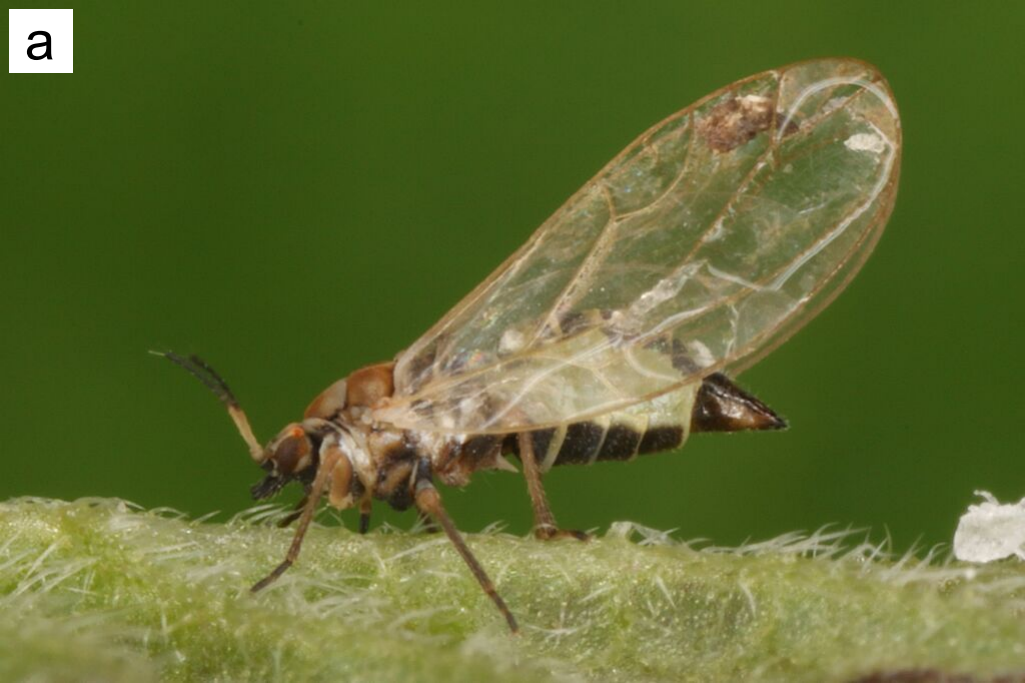
*Triozaurticae*, adult female;

**Figure 131b.**
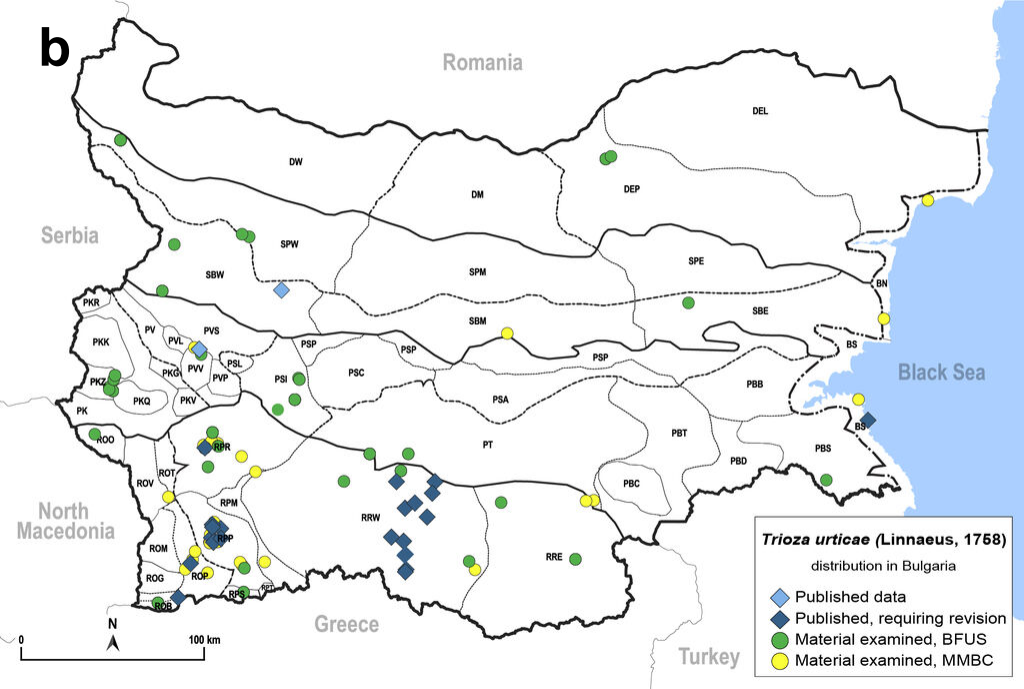
Distribution of *Triozaurticae* in Bulgaria.
