# Supplementary material for: Jumping plant lice (Hemiptera, Psylloidea) of Bulgaria – an annotated checklist
Source: Biodivers Data J. 2025 Mar 24;13:e147277. doi: 10.3897/BDJ.13.e147277 (PMC11959290; doi:10.3897/BDJ.13.e147277)
Supplement: Supplementary material 1 — Comparison of psyllid diversity in Bulgaria and other Balkan countries and Turkey [file bdj-13-e147277-s001.pdf]

Comparison of psyllid diversity in Bulgaria and other Balkan countries and Turkey

1. Burckhardt 1987; **2.** Dobreanu & Manolache 1962; **3.** Prodanović 2010; **4.** Drohojowska & Burckhardt 2014; **5.** Pintar 2023; **6.** Seljak 2020; **7.** recorded from North Macedonia: Lauterer & Burckhardt 2004 **8.** Burckhardt & Lauterer 1989; **9.** Bella & Rapisarda 2016; **10.** Karaca et *al*. 2015; **11.** Burckhardt et *al*. 2014; **12.** Lauterer et *al*. 2011; **13**. Ouvrard et *al*. 2016; **14.** Burckhardt & Hodkinson 1986; **15.** Burckhardt 1989; **16.** Prunar et al. 2023; **17.** Laz et al. 2024, **18.** Burckhardt 2005; **19.** Burckhardt & Lauterer 1982; **20.** Burckhardt & Lauterer 2006. See Ouvrard 2024 for full bibliographical data.

№	Species	Bulgaria	Greece[1]	Romania[2]	Serbia[3]	Turkey[4]	Croatia[5]	Slovenia[6]
1.	*Aphalara affinis*	X		X				
2.	*Aphalara avicularis*	X		X				X
3.	*Aphalara borealis*	X		X				
4.	*Aphalara calthae*			X			X	X
5.	*Aphalara exilis*	X	X					
6.	*Aphalara freji*	X		X	X			X
7.	*Aphalara grandicula*					X		
8.	*Aphalara longicaudata*							X
9.	*Aphalara maculipennis*	X			X	X		
10.	*Aphalara nigrimaculosa*	X						
11.	*Aphalara polygoni*	X	X	X				X
12.	*Aphalara purpurascens*			X				
13.	*Aphalara sauteri*							X
14.	*Aphalara ulicis*			X	X			
15.	*Colposcenia aliena*	X				X		
16.	*Colposcenia bidentata*	X				X		
17.	*Colposenia osmanica*	X				X		
18.	*Colposcenia traciana*	X	X				X	X
19.	*Craspedolepta araneosa*	X						
20.	*Craspedolepta armazhi*					X		
21.	*Craspedolepta artemisiae*	X		X				
22.	*Craspedolepta bulgarica*	X		X		X		X
23.	*Craspedolepta campestris*[7]							
24.	*Craspedolepta conspersa*	X			X			X
25.	*Craspedolepta flavipennis*			X			X	X
26.	*Craspedolepta innoxia*	X	X	X		X		
27.	*Craspedolepta latior*	X		X				
28.	*Craspedolepta malachitica*	X	X	X		X		X
29.	*Craspedolepta nebulosa*	X				X		X
30.	*Craspedolepta nervosa*	X	X	X	X		X	X
31.	*Craspedolepta omissa*	X	X			X		
32.	*Craspedolepta pontica*	X	X	X		X		
33.	*Craspedolepta setosa*					X		
34.	*Craspedolepta subpunctata*	X		X				
35.	*Craspedolepta sonchi*		X					
36.	*Crastina myricariae*					X		
37.	*Eumetoecus kochiae*	X						
38.	*Rhodochlanis bicolor*	X				X	X	X
39.	*Agonoscena cisti*		X			X	X	

Pramatarova M., Malenovský I., Gjonov I. Jumping plant lice (Hemiptera, Psylloidea) of Bulgaria – an annotated checklist

№	Species	Bulgaria	Greece[1]	Romania[2]	Serbia[3]	Turkey[4]	Croatia[5]	Slovenia[6]
40.	*Agonoscena pistaciae*	X	X			X		
41.	*Agonoscena succincta*						X	X
42.	*Agonoscena targionii*	X	X[8]			X	X	X
43.	*Lisronia varicicosta*		X[8]			X		
44.	*Megagonoscena gallicola*	X	X				X	X
45.	*Megagonoscena viridis*	X				X		
46.	*Rhinocola aceris*	X	X[8]	X	X	X	X	X
47.	*Ctenarytaina eucalypti*						X	
48.	*Glycaspis brimblecombei*		X[9]			X[10]	X	
49.	*Platyobria biemani*		X[11]					
50.	*Calophya rhois*	X	X	X	X	X	X	X
51.	*Homotoma ficus*	X	X	X	X	X	X	X
52.	*Euphyllura olivina*						X	X
53.	*Euphyllura phillyreae*	X	X			X	X	X
54.	*Euphyllura pakistanica*					X		
55.	*Euphyllura straminea*		X			X	X	
56.	*Psyllopsis discrepans*	X		X	X		X	X
57.	*Psyllopsis distinguenda*	X		X				X
58.	*Psyllopsis dobreanuae*	X		X				
59.	*Psyllopsis fraxini*	X		X	X		X	X
60.	*Psyllopsis fraxinicola*	X	X	X	X	X	X	X
61.	*Psyllopsis machinosus*	X		X	X	X		
62.	*Psyllopsis meliphila*	X		X	X		X	X
63.	*Psyllopsis securicola*					X		
64.	*Psyllopsis repens*				X	X		
65.	*Strophingia ericae*	X		X			X	X
66.	*Strophingia cinereae*	X	X					
67.	*Anomoterga unicolor*					X		
68.	*Aphorma lichenoides*	X	X			X		
69.	*Camaratoscena fulgidipennis*					X		
70.	*Camaratoscena hoberlandti*					X		
71.	*Camaratoscena lauta*					X		
72.	*Camarotoscena speciosa*	X	X	X	X	X	X	X
73.	*Camarotoscena subrubescens*	X				X	X	X
74.	*Livia crefeldensis*		X			?		
75.	*Livia junci*	X	X		X	X	X	X
76.	*Livia mediterranea*	X				X		
77.	*Acizzia acaciaebaileyanae*						X	
78.	*Acizzia jamatonica*	X	X[12]				X	
79.	*Acizzia uncatoides*		X[13]				X	
80.	*Diaphorina chobauti*		X				X	X
81.	*Diaphorina lycii*	X	X				X	
82.	*Diaphorina pusilla*		X					
83.	*Diaphorina putonii*		X			X	X	

№	Species	Bulgaria	Greece[1]	Romania[2]	Serbia[3]	Turkey[4]	Croatia[5]	Slovenia[6]
84.	*Arytaina genistae*	X	X	X			X	X
85.	*Arytaina maculata*	X	X					
86.	*Arytainilla cytisi*		X			X	X	
87.	*Arytainilla spartiophila*							X
88.	*Arytainilla spartiicola*	X						
89.	*Cacopsylla abdominalis*	X		X	X			
90.	*Cacopsylla affinis*	X	X	X	X	X		X
91.	*Cacopsylla albipes*	X			X	X		X
92.	*Cacopsylla ambigua*	X	X	X	X			X
93.	*Cacopsylla bidens*	X	X	X[14]	X	X	X	X
94.	*Cacopsylla breviantennata*	X		X	X	X	X	X
95.	*Cacopsylla brunneipennis*	X			X		X	X
96.	*Cacopsylla corcontum*	X			X			X
97.	*Cacopsylla crataegi*	X	X	X	X		X	X
98.	*Cacopsylla cretica*		X					
99.	*Cacopsylla elegantula*			X				X
100.	*Cacopsylla euxina*		X					
101.	*Cacopsylla fasciata*					X		
102.	*Cacopsylla fulguralis*						X	X
103.	*Cacopsylla incerta*		X			X		
104.	*Cacopsylla intermedia*						X	X
105.	*Cacopsylla iteophila*							X
106.	*Cacopsylla ledi*		X					
107.	*Cacopsylla mali*	X	X	X	X	X	X	X
108.	*Cacopsylla mariannae*					X		
109.	*Cacopsylla melanoneura*	X	X	X	X		X	X
110.	*Cacopsylla moscovita*					X		
111.	*Cacopsylla myrthi*					X	X	X
112.	*Cacopsylla myrtilli*	X						
113.	*Cacopsylla nigrita*	X		X	X			X
114.	*Cacopsylla notata*	X	X			X	X	
115.	*Cacopsylla parvipennis*							X
116.	*Cacopsylla peregrina*	X	X	X	X	X	X	X
117.	*Cacopsylla permixta*					X		
118.	*Cacopsylla picta*	X	X		X	X	X	X
119.	*Cacopsylla pruni*	X		X	X	X	X	X
120.	*Cacopsylla pulchella*	X	X			X	X	X
121.	*Cacopsylla pulchra*	X	X	X	X			X
122.	*Cacopsylla pyri*	X	X	X	X	X	X	X
123.	*Cacopsylla pyricola*	X	X		X		X	
124.	*Cacopsylla pyrisuga*	X	X	X	X	X	X	X
125.	*Cacopsylla rhamnicola*	X	X	X	X	X		X
126.	*Cacopsylla rhododendri*			X				
127.	*Cacopsylla saliceti*	X	X	X	X	X	X	X
128.	*Cacopsylla sorbi*	X			X			X
129.	*Cacopsylla suturalis*						X	

№	Species	Bulgaria	Greece[1]	Romania[2]	Serbia[3]	Turkey[4]	Croatia[5]	Slovenia[6]
130.	*Cacopsylla ulmi*	X		X	X			X
131.	*Cacopsylla viburni*							X
132.	*Cacopsylla visci*	X			X			X
133.	*Cacopsylla zetterstedti*		X			X		
134.	*Cyamophila astragalicola*					X		
135.	*Cyamophila glycyrrhizae*					X		
136.	*Cyamophila stoklosai*					X		
137.	*Livilla cognata*	X			X			
138.	*Livilla hodkinsoni*		X			X		
139.	*Livilla horvathi*	X	X	X		X		X
140.	*Livilla radiata*	X	X	X	X		X	X
141.	*Livilla smyrnensis*					X[15]		
142.	*Livilla spectabilis*		X			X	X	X
143.	*Livilla ulicis*	X		X				X
144.	*Livilla variegata*	X					X	X
145.	*Livilla vicina*							X
146.	*Livilla vittipennella*							X
147.	*Psylla alni*	X	X		X	X	X	X
148.	*Psylla alpina*	X		X				X
149.	*Psylla betulae*		X					
150.	*Psylla colorata*	X	X			X		X
151.	*Psylla foersteri*	X	X	X	X	X	X	X
152.	*Psylla fusca*	X		X				X
153.	*Psylla hartigii*	X		X			X	X
154.	*Spanioneura buxi*	X	X		X		X	X
155.	*Spanioneura caucasica*					X		
156.	*Spanioneura fonscolombii*	X		X[16]		X[17]	X	X
157.	*Spanioneura pechai*					X		
158.	*Spanioneura persica*					X		
159.	*Spanioneura turkiana*					X		
160.	*Bactericera acutipennis*	?	X					
161.	*Bactericera albiventris*	X	X	X	X	X	X	X
162.	*Bactericera bohemica*	X		X				X
163.	*Bactericera crithmi*					X	X	X
164.	*Bactericera curvatinervis*	X	X	X	X			X
165.	*Bactericera femoralis*	X		X		X		X
166.	*Bactericera harrisoni*	X		X				X
167.	*Bactericera kratochvili*							X
168.	*Bactericera lyrata*	X						X
169.	*Bactericera maura*						X	
170.	*Bactericera modesta*	X					X	X
171.	*Bactericera nigricornis*	X	X	X	X	X	X	X
172.	*Bactericera parastriola*							X
173.	*Bactericera perrisii*	X	X				X	X
174.	*Bactericera reuteri*	?						
175.	*Bactericera silvarnis*		X[18]					
176.	*Bactericera striola*	X		X				X

№	Species	Bulgaria	Greece[1]	Romania[2]	Serbia[3]	Turkey[4]	Croatia[5]	Slovenia[6]
177.	*Bactericera tremblayi*		X		X	X		
178.	*Bactericera trigonica*	X	X			X		X
179.	*Dyspersa abdominalis*	X						X
180.	*Dyspersa achilleae*					X		
181.	*Dyspersa apicalis*	?		X				X
182.	*Dyspersa carpathica*			X				
183.	*Dyspersa chrysanthemi*			X	X			X
184.	*Dyspersa cirsii*	X	X	X	X			X
185.	*Dyspersa flixiana*							X
186.	*Dyspersa kantshavelii*	X				X		
187.	*Dyspersa laserpitii*			X[19]	X			X
188.	*Dyspersa mesembrina*	X			X			
189.	*Dyspersa munda*	X		X				X
190.	*Dyspersa pallida*	X				X		X
191.	*Dyspersa senecionis*			X				X
192.	*Dyspersa schrankii*			X				X
193.	*Egeirotrioza populi*					X		
194.	*Egeirotrioza gegechkorii*		X					
195.	*Eryngiofaga babugani*	X						
196.	*Eryngiofaga dlabolai*	X						
197.	*Eryngiofaga mesomela*		X	X				
198.	*Eutrioza opima*					X		
199.	*Heterotrioza chenopodii*	X	X	X	X		X	X
200.	*Heterotrioza dichroa*	X	X	X				
201.	*Heterotrioza kochiae*	X						
202.	*Heterotrioza portulacoides*							X
203.	*Lauritrioza alacris*	X	X			X	X	X
204.	*Phylloplecta trisignata*	X	X			X	X	X
205.	*Spanioza drosopouli*		X[20]					
206.	*Spanioza galii*	X	X	X		X	X	X
207.	*Spanioza velutina*	X					X	X
208.	*Trichochermes marginepunctatus*						X	
209.	*Trichochermes rhamni*	X	X	X	X	X		X
210.	*Trichochermes walkeri*	X		X	X			X
211.	*Trioza centranthi*		X	X	X	X	X	X
212.	*Trioza cerastii*	X		X				X
213.	*Trioza dispar*	?			X			
214.	*Trioza flavipennis*	X	X	X			X	X
215.	*Trioza foersteri*		X	X	X	X		X
216.	*Trioza ilicina*					X	X	X
217.	*Trioza magnisetosa*					X		
218.	*Trioza megacerca*	X					X	X
219.	*Trioza neglecta*	X			X	X		
220.	*Trioza proxima*	X					X	X
221.	*Trioza remota*	X	X	X	X	X	X	X
222.	*Trioza rotundata*	X		X	X			X

№	Species	Bulgaria	Greece[1]	Romania[2]	Serbia[3]	Turkey[4]	Croatia[5]	Slovenia[6]
223.	*Trioza rumicis*	X		X				
224.	*Trioza saxifragae*							X
225.	*Trioza scottii*					X	X	X
226.	*Trioza soniae*						X	X
227.	*Trioza tatrensis*			X				
228.	*Trioza urticae*	X	X	X	X	X	X	X
	**Total:**	**130**	**89**	**86**	**63**	**101**	**86**	**126**
